# Development of VU6036864: A Triazolopyridine-Based High-Quality Antagonist Tool Compound of the M_5_ Muscarinic Acetylcholine Receptor

**DOI:** 10.1021/acs.jmedchem.4c01193

**Published:** 2024-08-06

**Authors:** Jinming Li, Douglas L. Orsi, Julie L. Engers, Madeline F. Long, Rory A. Capstick, Mallory A. Maurer, Christopher C. Presley, Paige N. Vinson, Alice L. Rodriguez, Allie Han, Hyekyung P. Cho, Sichen Chang, Megan Jackson, Michael Bubser, Anna L. Blobaum, Olivier Boutaud, Michael A. Nader, Colleen M. Niswender, P. Jeffrey Conn, Carrie K. Jones, Craig W. Lindsley, Changho Han

**Affiliations:** † Warren Center for Neuroscience Drug Discovery, Vanderbilt University, Nashville, Tennessee 37232, United States; ‡ Department of Pharmacology, Vanderbilt University, Nashville, Tennessee 37232, United States; § Center for the Neurobiology of Addiction Treatment, Wake Forest School of Medicine, Medical Center Boulevard Winston-Salem, Winston-Salem, North Carolina 27157, United States

## Abstract

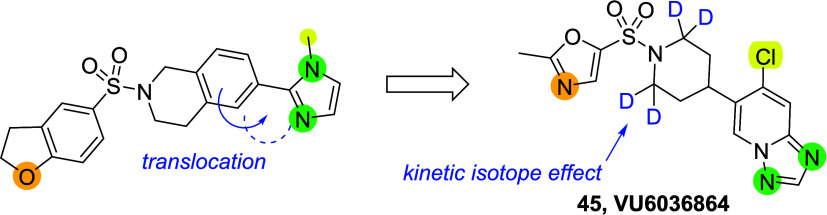

While the muscarinic acetylcholine receptor mAChR subtype 5 (M_5_) has been studied over decades, recent findings suggest that more in-depth research is required to elucidate a thorough understanding of its physiological function related to neurological and psychiatric disorders. Our efforts to identify potent, selective, and pharmaceutically favorable next-generation M_5_ antagonist tool compounds have led to the discovery of a novel triazolopyridine-based series. In particular, **VU6036864** (**45**) showed exquisite potency (human M_5_ IC_50_ = 20 nM), good subtype selectivity (>500 fold selectivity against human M_1–4_), desirable brain exposure (*K*_p_ = 0.68, *K*_p,uu_ = 0.65), and high oral bioavailability (%*F* > 100%). **VU6036864** (**45**) and its close analogues will support further studies of M_5_ as advanced antagonist tool compounds and play an important role in the emerging biology of M_5_.

## Introduction

Muscarinic acetylcholine receptors (mAChRs) are class A GPCRs composed of five subtypes, M_1_–M_5_.^1,2^ The interplay between the muscarinic ACh receptors (mAChRs) and their ligand, acetylcholine (ACh), is responsible for important functions including learning, memory, and involuntary muscle contraction in various organs including the brain.^1,2^ In general, expression levels of each subtype vary in different tissues and organs, and the physiological roles of each subtype vary accordingly. Of these subtypes, M_1_ and M_4_ are expressed in specific regions of the brain and play an important role in the central nervous system (CNS).^1,3^ Besides CNS-related functions, mAChRs have also been shown to play a role in tumor progression and metastasis through specific signaling pathways, and particular subtypes, such as M_3_ have emerged as cancer treatment options.^4,5^ Although less abundantly expressed in the CNS than the M_1_ and M_4_ receptors, M_5_ is highly expressed in dopamine neurons in the ventral tegmental area and the substantia nigra.^6,7^ Previously, several reports on the M_5_ receptor suggested that M_5_ may be an additional emerging therapeutic target.^8−15^ However, recent studies highlighted the complex nature of M_5_ biology and underscored the importance of further investigation into its role.^16,17^

In short, the physiological role of M_5_ remains unclear even decades after its initial identification and in-depth studies from multiple angles using a variety of tools are still required.

**Figure 1:**
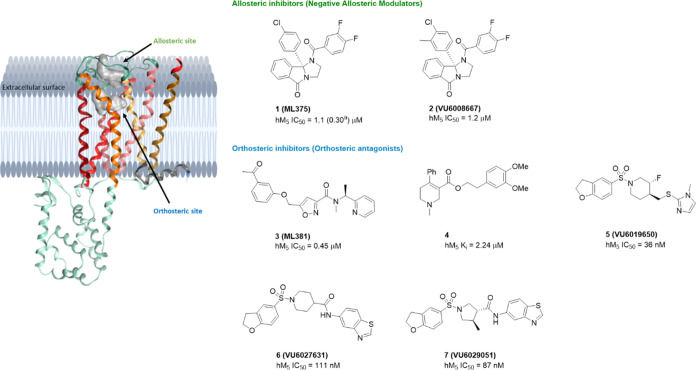
Crystal structure of M_5_ mAChR (PDB code: 6OL9)^9^ and reported allosteric and orthosteric M_5_ inhibitors.^11,18−21^^a^ IC_50_ value was obtained from a 96-well FlexStation II microplate reader.

As illustrated in Figure 1 with a crystal structure of M_5_, allosteric ligands that activate or inhibit M_5_ generally bind to the outer surface of the receptor above the orthosteric binding site, whereas orthosteric ligands have their binding sites within the transmembrane domain of the receptor.^9^ To date, two allosteric inhibitors **1** and **2** (or negative allosteric modulators) and five orthosteric inhibitors **3**–**7** (or orthosteric antagonists) have been reported as early generation inhibitor tools for M_5_ (Figure 1). Nonetheless, efforts to identify clinically acceptable compounds and better understand the physiological role of the M_5_ receptor remain unmet needs. Herein, we report the discovery effort that led to the identification of **VU6036864** (**45**) and its close analogues, which will be used to better understand M_5_ biology.

## Results and Discussion

### Design Rationale for A New Chemotype

**Scheme 1:**
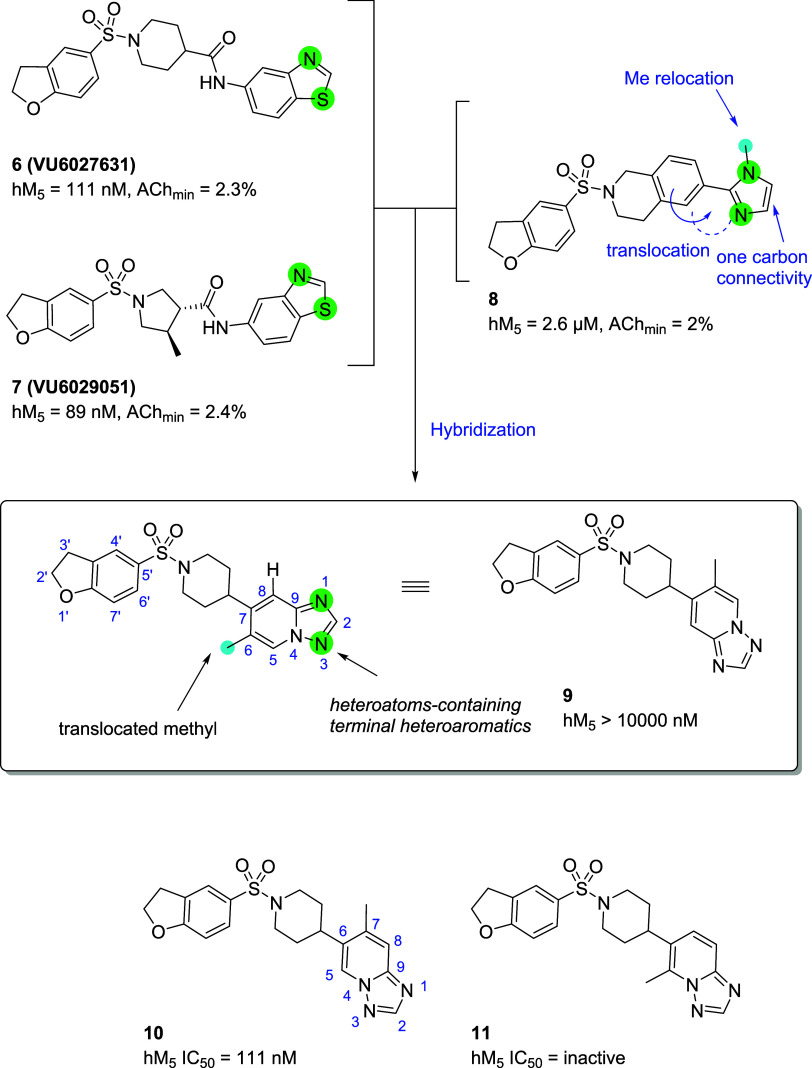
Rationalized Design of Triazolopyridine-Based M_5_ Antagonists

Our journey to find clinically amenable M_5_ antagonists, starting from a potent and selective M_5_ antagonist **VU6019650** (**5**), had reached a stage where the focus needed to be on optimizing brain exposure in addition to clearance. Although compounds within piperidine and pyrrolidine amide-based M_5_ antagonists (exemplified by **6** and **7**) showed good potencies with improvement in clearance profiles, their brain exposures were still suboptimal due to P-gp recognition.^20,21^ To overcome these challenges, we attempted to remove the hydrogen bond donor by methylating the amide NH as compounds lacking hydrogen bond donors tend not to be recognized by P-gp.^22^ However, methylation of the amide linkage was not tolerated and new chemotypes had to be devised through a scaffold hopping exercise.^20^ In this regard, compound **8**, found during the core modification exercises, became an excellent hybridization starting point (Scheme 1). While **8** lacked hydrogen bond donors, it was still moderately potent (hM_5_ IC_50_ = 2.6 μM, ACh_min_ = 2%).

Compound **9** was designed under the assumption that the phenyl ring translocated to imidazole in the tetrahydroisoquinoline core of **8** could mimic the benzo[*d*]thiazole ring of **6** or **7**, and that a methyl substituent in **8** could mimic the chiral methyl of **7** without interfering with the interaction of the triazolopyridine nitrogen atom if it were translocated to the 6-position of the triazolopyridine. As blocking the heteroatoms of the benzo[*d*]thiazole ring of **6** was observed to reduce potency in the past, we thought it was important to locate the substitution of the methyl group without disturbing the triazolopyridine nitrogen atom.^20^ In addition to compound **9**, triazolopyridine regioisomer **10** was synthesized in parallel, maintaining the same overall design logic.

Although the potency of **9** was very weak, we were pleased to see that compound **10** had improved potency compared to compound **8** (**9**; hM_5_ IC_50_ > 10,000 nM, ACh_min_ = 59%, **10**; hM_5_ IC_50_ = 111 nM, ACh_min_ = 2%). Compound **11** was also synthesized to test our hypothesis that the position of the methyl substituent is important. As expected, **11** was inactive, indicating that both nitrogen atoms of the triazolopyridine ring had to be exposed, similar to the cases in which the heteroatoms on benzo[*d*]thiazole rings of **6** and **7** had to be exposed to maintain efficacies. Between the triazolopyridine regioisomers **9** and **10**, the one on **10** was selected as an optimal new starting point based on the potency. With a new chemotype possessing excellent potency in hand, we sought to explore the SAR around compound **10** and profile selected analogues to find compounds with desirable clearance and brain exposure.

### Structure–Activity Relationship

Our initial investigations were focused on the sulfonamide substituent (Table 1). Attempts to increase the ring size from a 5,6-fused ring (**10**; 2,3-dihydrobezofuran) to a 6,6-fused ring system resulted in a loss of potency in general (**12**; hM_5_ IC_50_ = 187 nM, ACh_min_ = 2%, **13**; hM_5_ IC_50_ = 1520 nM, ACh_min_ = 3%, and **14**; hM_5_ IC_50_ = 547 nM, ACh_min_ = 3%). Based on this SAR trend, we hypothesized that a 5,6-fused ring might be an optimal size and replaced 2,3-dihydrobezofuran of **10** with alternative 5,6-fused rings such as benzimidazole. However, the benzimidazole was not tolerated as well (**15**; hM_5_ IC_50_ = 5200 nM, ACh_min_ = 5%).

**Table 1:**
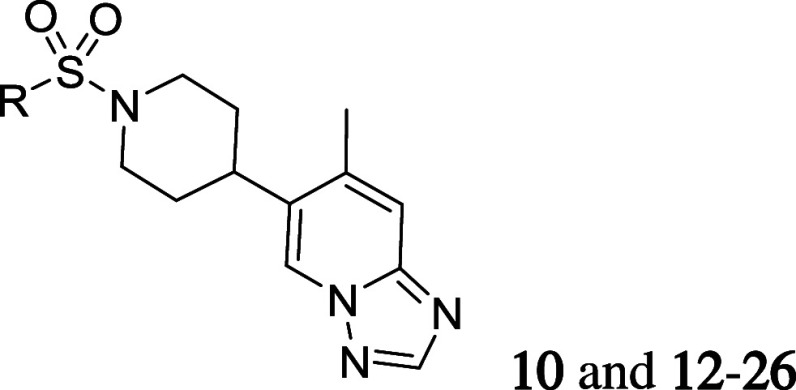

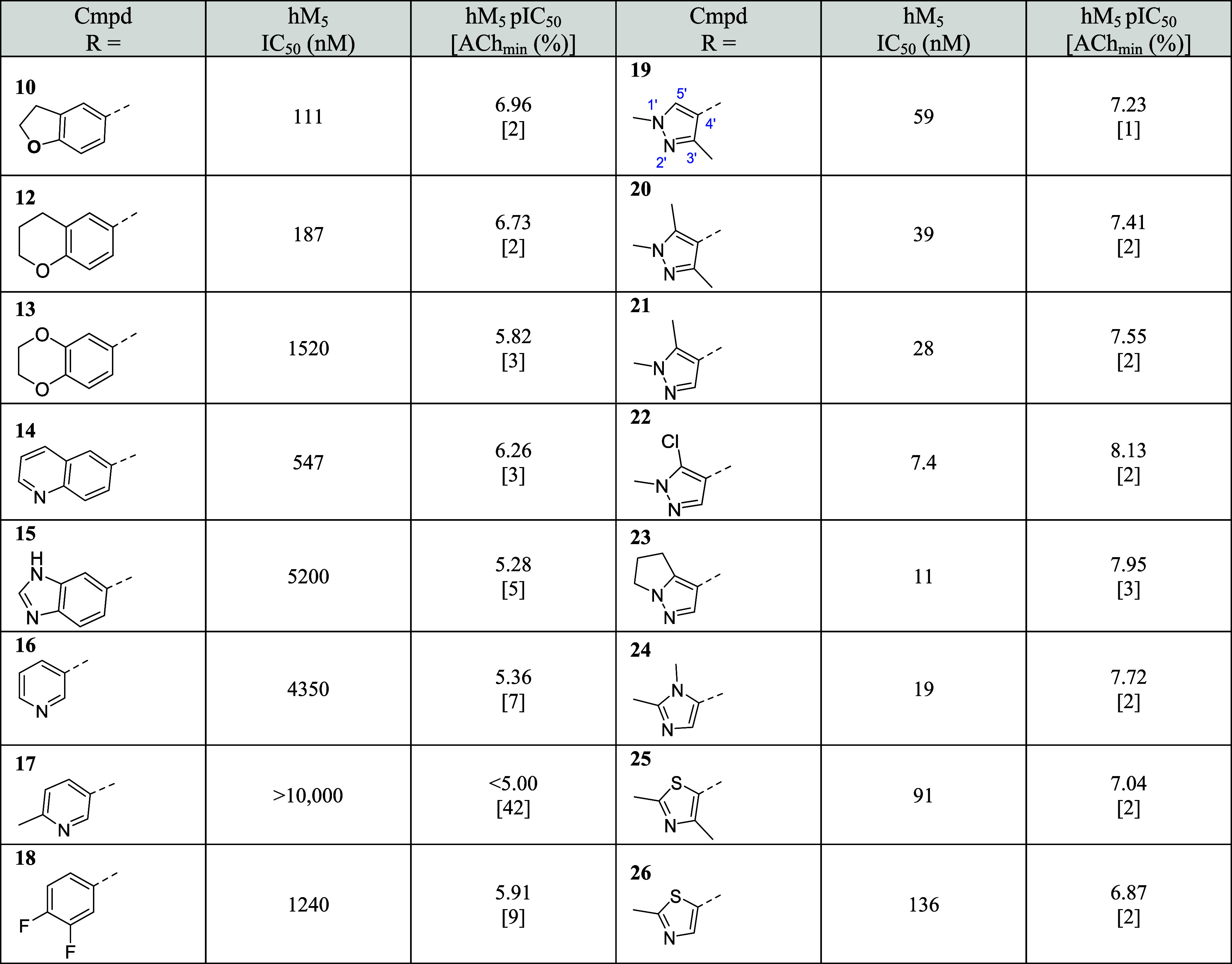
SAR of Sulfonylated Aryl Variants^a^

a Calcium mobilization assays in hM_5_ CHO cells. Refer to the Supporting Information (SI)Experimental Section for assay conditions. IC_50_ values for hM_5_ represent the means from a minimum of two independent experiments performed in triplicate. ACh_min_ (%) represents the remaining Ca response measured in the presence of an ACh EC_80_ concentration. Refer to the Molecular formula strings (CSV) file for additional details including mean ± SEM.

From our previous experience with piperidine and pyrrolidine amide-based M_5_ antagonists (exemplified by compounds **6** and **7**), we found that smaller-size rings provide a surprising potency boost or drop-off.^20,21^ Therefore, pyridine-based analogs (**16** and **17**) and 5-membered heterocycle-containing analogs were also explored (**19–26**).

Similar to piperidine amide-based M_5_ antagonists, 5-membered heterocycles like pyrazole were well tolerated. However, pyridine-based analogs showed much weaker efficacy compared to 5-membered heterocycle-based analogs (**16**; hM_5_ IC_50_ = 4350 nM, ACh_min_ = 7%, **17**; hM_5_ IC_50_ > 10,000 nM, ACh_min_ = 42%). While the 1′,3′-dimethyl-1*H*-pyrazole containing analog **19** was modestly more potent (**19**; hM_5_ IC_50_ = 59 nM, ACh_min_ = 1%) compared to compound **10** (hM_5_ IC_50_ = 111 nM, ACh_min_ = 2%), adding a 5′-position methyl substituent noticeably increased potency (**20**; hM_5_ IC_50_ = 39 nM, ACh_min_ = 2%). To determine whether the 3′-position substituent is essential for potency, compound **21** with 1′,5′-dimethyl substituents was also tested. Interestingly, **21** still retained potency without a 3′-position methyl substituent (**21**; hM_5_ IC_50_ = 28 nM, ACh_min_ = 2%). Therefore, we focused on a 5′-position substitution without any substituent on a 3′-position of pyrazole.

Attempts to replace the 5′-methyl of compound **21** with chlorine resulted in a significant potency increase (**22**; hM_5_ IC_50_ = 7.4 nM, ACh_min_ = 2%). This SAR trend could imply that an increase in hydrophobicity is favored, or that the chlorine atom is forming additional interactions, such as halogen bonding interactions. To test these possibilities and further understand the SAR trend, we prepared compound **23** with 5,5-fused bicycle moiety and found that **23** exhibited similar potency to **22**, confirming that the potency boost may be due to hydrophobicity rather than additional interactions (**23**; hM_5_ IC_50_ = 11 nM, ACh_min_ = 3%, **22**; hM_5_ IC_50_ = 7.4 nM, ACh_min_ = 2%).

5-Membered heterocycles with enhanced basicity compared to a pyrazole were also explored. We anticipated compounds with enhanced basicity might form stronger hydrogen bonding interactions with the receptor, resulting in an additional potency enhancement or a slight increase in aqueous solubility while maintaining efficacy. As expected, explored alternative 5-membered heterocycles such as imidazoles or thiazoles were tolerated as well. While 1,2-dimethyl-1*H*-imidazole was slightly more potent than 1,5-dimethyl-1*H*-pyrazole (**24**; hM_5_ IC_50_ = 19 nM, ACh_min_ = 2%, **21**; hM_5_ IC_50_ = 28 nM, ACh_min_ = 2%), the 2,4-dimethylthiazole moiety was slightly less potent compared to the 1,3-dimethyl-1*H*-pyrazole moiety (**25**; hM_5_ IC_50_ = 91 nM, ACh_min_ = 2%, **20**; hM_5_ IC_50_ = 39 nM, ACh_min_ = 2%). In addition, monomethyl thiazole analogue **26** was not as potent, reiterating the importance of space filling with hydrophobic moiety around the upper part of the ring for potency (**26**; hM_5_ IC_50_ = 136 nM, ACh_min_ = 2%).

Lastly, a disubstituted phenyl ring (compound **18**) was also explored to see whether two fluorine atoms (or substituents) could mimic the oxygen atom of 2,3-dihydrobenzofuran and the nitrogen atom of 5-membered azoles at the same time. While compound **18** was still moderately active, it was significantly weaker than 5-membered azole-based analogues (**18**; hM_5_ IC_50_ = 1240 nM, ACh_min_ = 9%). Therefore, our focus remained on the 5-membered azoles. However, it should be noted that adding hydrophobic substituents to the upper part of the ring could further enhance the efficacy.

Due to the high potency gained in **22**, our SAR investigation then moved to the triazolopyridine side while maintaining the 5-chloro-1-methyl-1*H*-pyrazole and piperidine (Table 2). Although the ideal methyl substituent position was confirmed with **10** and **11** (Scheme 1), a desmethyl analogue such as **27** was needed to demonstrate the substituent effect. As shown in Table 2, **27** was over 20-fold weaker compared to compound **22**, indicating the importance of substituent on the 7-position of the triazolopyridine ring (**27**; hM_5_ IC_50_ = 176 nM, ACh_min_ = 3%). While a chlorine atom, a bioisostere of methyl, was well tolerated (**28**, hM_5_ IC_50_ = 8.3 nM, ACh_min_ = 2%), a fluorine atom was about 2.5-fold weaker (**29**, hM_5_ IC_50_ = 19 nM, ACh_min_ = 1%). This SAR trend suggested that larger substituents might be preferred at the 7-position of the triazolopyridine ring. Therefore, analogs with larger hydrophobic substituents were prepared (compounds **30**-**32**). Although larger hydrophobic substituents were tolerated, their potencies were notably weaker compared to compounds **22** and **28**. (**30**; CF_3_; hM_5_ IC_50_ = 55 nM, ACh_min_ = 3%, **31**; Et; hM_5_ IC_50_ = 21 nM, ACh_min_ = 2%, **32**; cyclopropyl; hM_5_ IC_50_ = 42 nM, ACh_min_ = 2%). However, hydrophobic substituents were much more potent than hydrophilic substituents such as methoxy (**33**, hM_5_ IC_50_ = 518 nM, ACh_min_ = 2%).

**Table 2:**
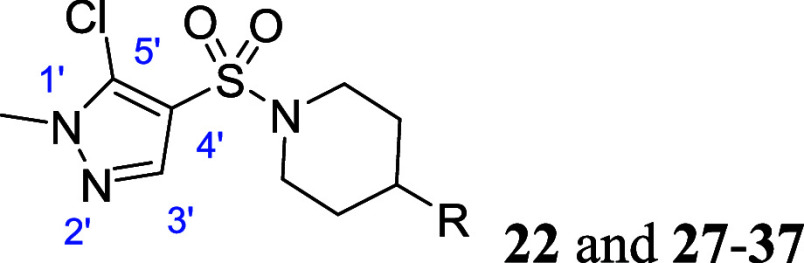

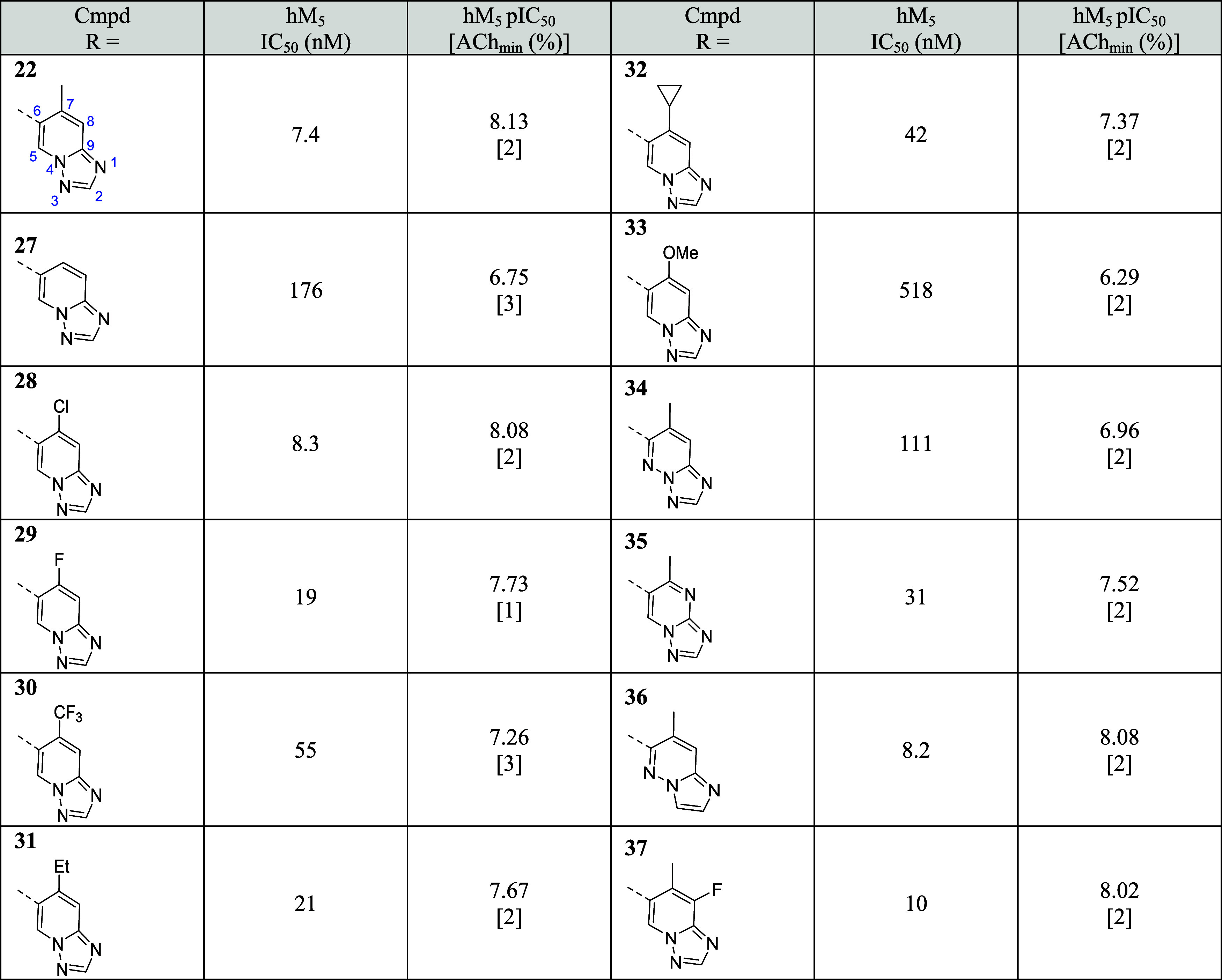
SAR of the Azolopyridine Variants^a^

a Calcium mobilization assays in hM_5_ CHO cells. Refer to the Supporting InformationExperimental Section for assay conditions. IC_50_ values for hM_5_ represent the means from a minimum of two independent experiments performed in triplicate. ACh_min_ (%) represents the remaining Ca response measured in the presence of ACh EC_80_ concentration. Refer to the Molecular formula strings (CSV) file for additional details including mean ± SEM.

This SAR trend was also maintained in aza-triazolopyridine-based analogues. Both compound **34** with 5-aza-triazolopyridine and compound **35** with 8-aza-triazolopyridine were less potent compared to compound **22**. (**34**; hM_5_ IC_50_ = 111 nM, ACh_min_ = 2%, **35**; hM_5_ IC_50_ = 31 nM, ACh_min_ = 2%, **22**; hM_5_ IC_50_ = 7.4 nM, ACh_min_ = 2%). Interestingly, the removal of a nitrogen atom from the 3-position of compound **34** resulted in a significant boost in potency (**36**; hM_5_ IC_50_ = 8.2 nM, ACh_min_ = 2%). Although the compound became more soluble, imidazo[1,2-*b*] pyridazine-based analogues generally suffered from poor subtype selectivity (*data not shown*). Therefore, triazolopyridines have remained as a lead series.

Lastly, since the hydrophobic nature was generally favored, we attempted to incorporate an additional fluorine atom into **22**, and the small fluorine atom on the 8-position of triazolopyridine was well tolerated (**37**; hM_5_ IC_50_ = 10 nM, ACh_min_ = 2%).

### Tier 1 DMPK Profile of Selected Compounds

Selected compounds from the initial SAR exploration were then advanced to the subsequent tier 1 DMPK profiling stage (Table 3 and *see*SI S3*for additional available DMPK profiling data for the remaining compounds*). In general, compounds within the triazolopyridine-based series showed favorable DMPK profiles. The molecular weights (MWs) of synthesized analogues were far below 500, cLogPs were below 2, and TPSAs were below 100. Compounds such as **19** and **26** remain low to moderate *in vitro* clearance even after the scaffold hopping from the pyrrolidine amide series (**19**; predicted human CL_hep_ = 5; predicted rat CL_hep_ = 14 mL/min/kg), (**26**; predicted human CL_hep_ = 7; predicted rat CL_hep_ = 27 mL/min/kg). In addition, the triazolopyridine series showed an excellent *f*_u_ in general (*f*_u_ > 0.1 in many cases). As expected, P-gp liability was not as severe due to the absence of hydrogen bond donors in general. Especially, compounds **26** and **28** were not P-gp substrates (**26**; ER = 3.46, **28**; ER = 1.78). CYP inhibition profiles were also generally acceptable, although some compounds showed marginal CYP inhibitory activity, particularly against CYP 2D6 and 3A4.

**Table 3:**
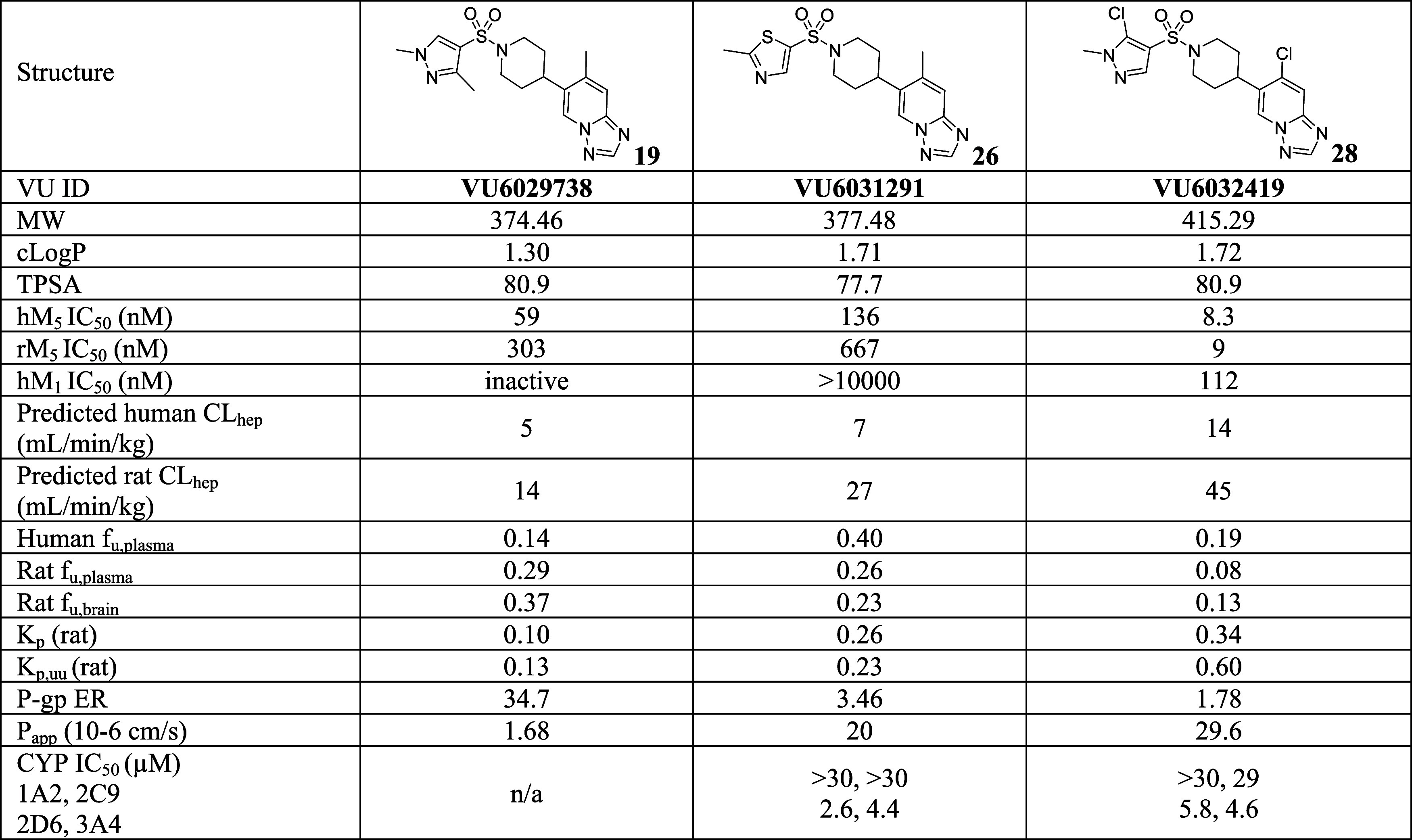
Tier 1 DMPK Profiles for Selected M_5_ Antagonists

**Scheme 2:**
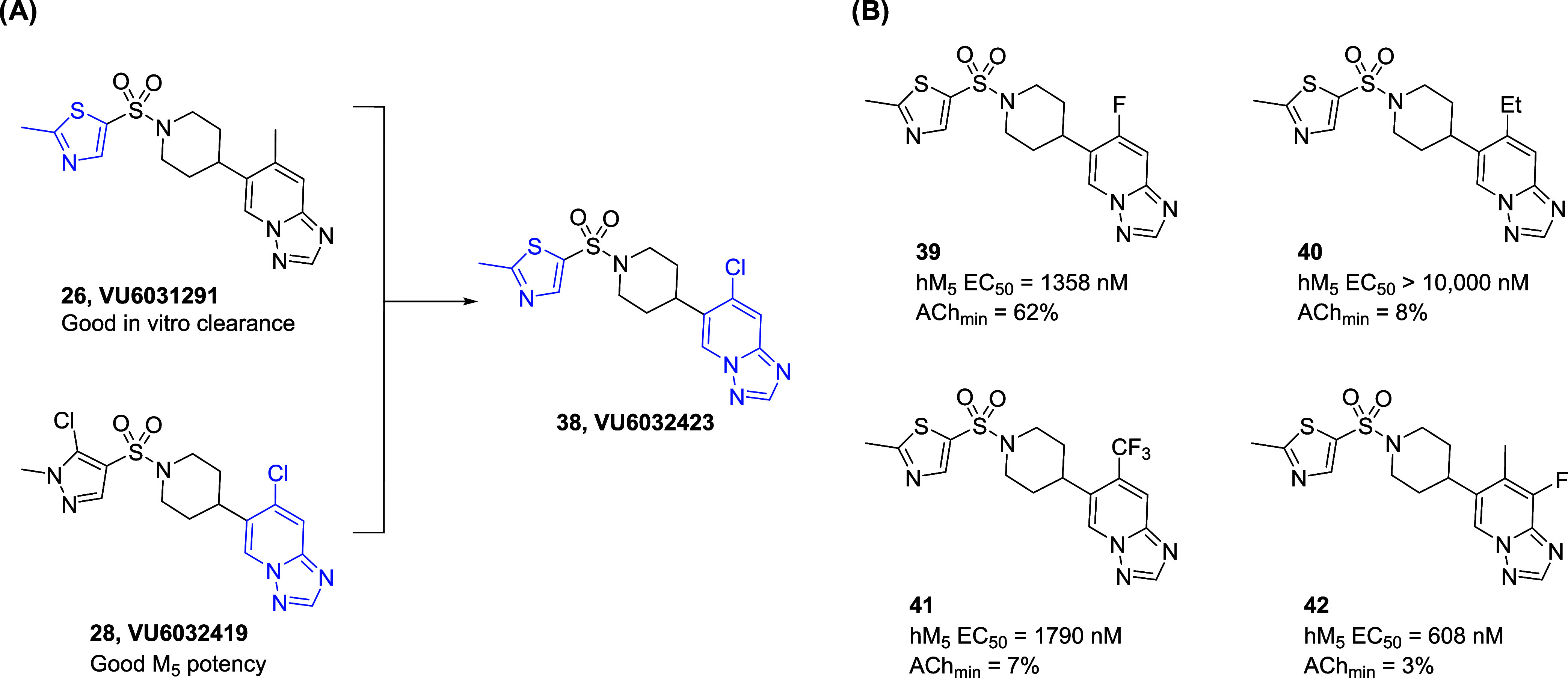
Design of **VU6032423** (**38**) and Its Close Analogues (A) **38** is designed as a mix and match of **26** and **28**. (B) Potencies of **39**-**42.**

Slight differences in DMPK profiles within structurally related subseries often provide an excellent mix-and-match opportunity. While compound **26** is considerably weaker compared to compound **28** (hM_5_ IC_50_ = 136 and 8.3 nM, respectively), its *in vitro* clearance profile was much more attractive (predicted human CL_hep_ = 7 and 14 mL/min/kg, respectively). Because both compounds **26** and **28** were not P-gp substrates, they were selected as mix-and-match candidates. (Scheme 2A).

**Figure 2:**
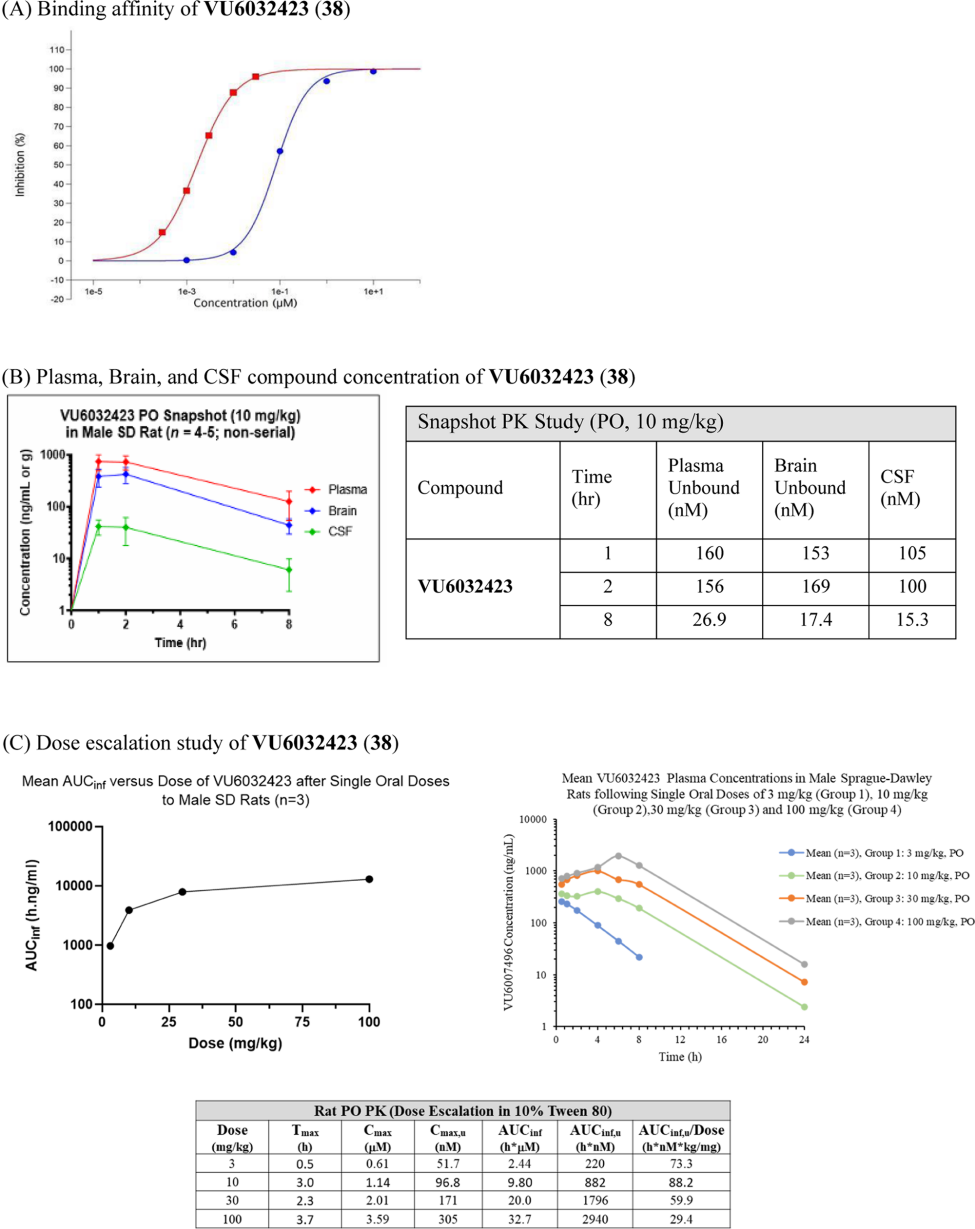
Tier 2 DMPK profiles of **VU6032423 (38)** (A) The binding affinity of **VU6032423** was measured against the human M_5_ receptor and results are presented as the percent inhibition of specific binding. Red box solid 4-DMAP (control): IC_50_ = 1.64 nM and *K_i_* = 1.02 nM blue circle solid **VU6032423**: IC_50_ = 50 nM and *K_i_* = 51 nM; Eurofins Panlabs Discovery Services study #: TW04–0006869, (B) Plasma, brain, and CSF compound concentration of **VU6032423** after 10 mg/kg PO dosing in 10% Tween 80 in water. Samples were obtained at 1, 2, and 8 h time points. The table shows a summary of the total and unbound concentrations in each compartment. (C) Dose escalation study result of **VU6032423**. Mean AUC_inf_ versus 3, 10, 30, and 100 mg/kg dosing of **VU6032423** in 10% Tween 80 in water.

Compound **38** was designed based on the 2-methylthiazole moiety from **26** and 7-chloro-[1,2,4]triazolo[1,5-*a*]pyridine from **28**. As shown in Table 4, compound **38** was quite potent (**38**, hM_5_ IC_50_ = 50 nM, ACh_min_ = 2%). Besides, the binding affinity and functional efficacy of **22** were closely aligned (51 nM and 50 nM, respectively, Figure 2A). Full displacement of the radioligand observed in this binding assay suggests an orthosteric mechanism of action similar to **5**;^11^ additionally, several compounds within the series were evaluated using kinetic binding and were consistent with a competitive interaction (*data not shown*). Unexpectedly, while *in vitro* clearance profile (**38**; predicted human CL_hep_ = 13; predicted rat CL_hep_ = 42 mL/min/kg) and P-gp efflux ratio (P-gp ER = 1.49) were still similar to compound **28**, the CYP inhibition profile was notably improved (**38**; CYP IC_50_ 1A2, 2C9, 2D6, and 3A4 = > 30, > 30, > 30, and 17.2 μM).

**Table 4:**
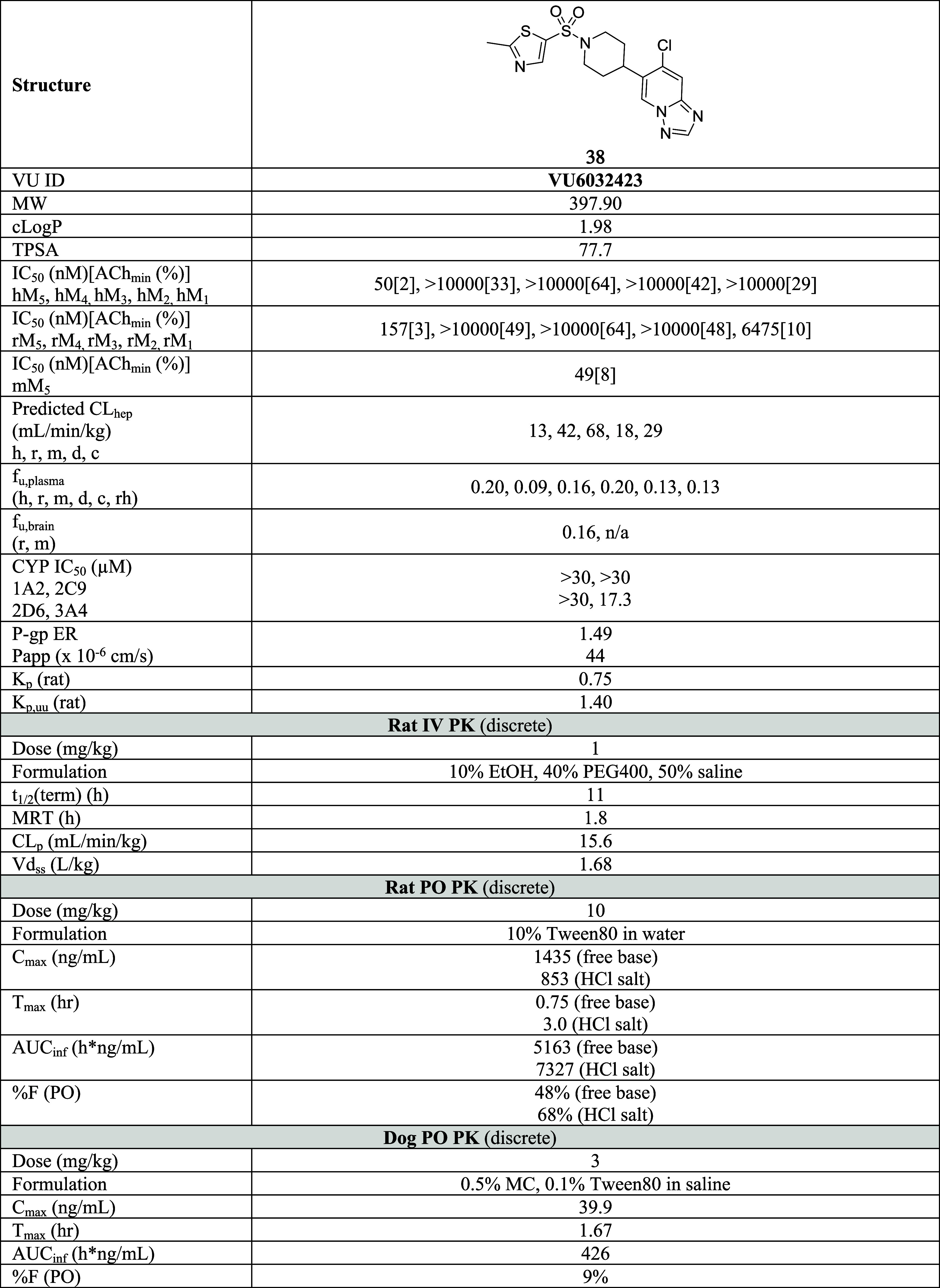
Profile of **VU6032423 (38)**

To further improve the *in vitro* clearance profiles, substituents on the triazolopyridine ring were explored (Scheme 2B). However, analogues with different substituents were not as potent (**39**-**42**). Therefore, compound **38** was selected as a first-generation lead compound within the newly discovered triazolopyridine series and advanced to the subsequent tier 2 DMPK profiling exercise (Table 4).

### Detailed Profile of **38**

The overall profile of compound **38** was promising (Table 4). Molecular weight, cLogP, and TPSA values were still in drug-like chemical space (MW = 397.9, cLogP = 1.98, and TPSA = 77.7). In addition, it was potent and subtype-selective (hM_5_ IC_50_ = 50 nM, ACh_min_ = 2%, > 200 fold-selective against human M_1–4_). While the overall receptor subtype selectivity trend was retained in rat (rM_1_ IC_50_ = 6475 nM and rM_2–4_ IC_50_ > 10,000 nM), moderate species differences in potency were noticed with rat M_5_, but not with mouse M_5_ (rM_5_ IC_50_ = 157 nM, ACh_min_ = 3%, mM_5_ IC_50_ = 49 nM, ACh_min_ = 8%). It is worth mentioning that although **38** selectively antagonized the M_5_ receptor in functional assays, **38** possessed weak binding affinities against M_2_ and M_4_ receptor subtypes and its K_i_s were 1.1 and 2.1 μM, respectively (See SI S4). Notably, compound **38** revealed a relatively clean ancillary pharmacology profile (see SI S10).

Predicted *in vitro* CL_hep_ values were low to moderate across species (human, rat, mouse, dog, and cyno = 13, 42, 68, 18, 29 mL/min/kg). In addition, both plasma and brain *f*_u_ values were excellent (>0.1 across all tested species). As mentioned, the CYP inhibition profile was good as well (**38**; CYP IC_50_ 1A2, 2C9, 2D6, and 3A4 = > 30, > 30, > 30, and 17.3 μM, respectively). Lastly, compound **38** was not a P-gp substrate with an efflux ratio of 1.49.

Rat *in vivo* DMPK profiles from IV and PO PK studies were also promising. The half-life was 11 h with a MRT of 1.8 h. CL_p_ was 15.6 mL/min/kg and V_ss_ was 1.68 L/kg. While %F with 10% Tween80 in water was 48%, oral bioavailability was significantly improved with the HCl salt (68%). In the latter case, *C*_max_ was 853 ng/mL with *T*_max_ of 3.0 h. AUC was 7327 h*ng/mL. However, dog PK was not as appealing. Oral bioavailability was 9% with 3 mg/kg dosing. *C*_max_ was 39.9 ng/mL with *T*_max_ of 1.67 h. AUC was 426 h*ng/mL.

**Figure 3:**
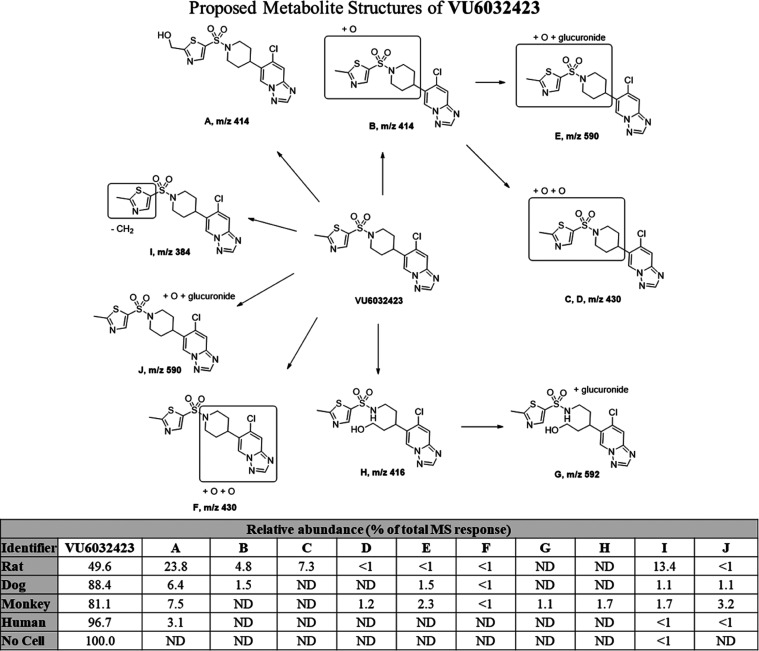
Multispecies metabolite identification of **VU6032423** (**38**). Metabolite A was the most abundant metabolite in human hepatocytes with a good preclinical safety species coverage. ND = Not Detected.

As shown in Figure 2B, a high concentration of compound was detected during the rat PK snapshot study. Both brain unbound concentration and CSF concentration were above the IC_50_ within 2 h time points. Exposure increased with dose and plateaued between 10 and 30 mg/kg, suggesting decreased absorption at higher doses (Figure 2C; 3, 10, 30, and 100 mg/kg). At 100 mg/kg dosing, *C*_max,unbound_ was 305 nM with AUC_unbound_ of 5227 h*nM. *T*_max_ was 3.67 h.

**38** was also subjected to multispecies metabolite identification (MetID) studies (Figure 3). It was fairly stable within 4 h of study time in all selected species, especially higher species (dog, monkey, and human). Because metabolite A (Met-A) was the most abundant metabolite in human hepatocytes with a good preclinical safety species coverage, we advanced it to further profiling (Table 5).

**Table 5:** Profile of **Met-A** (**43**)

VU ID	VU6043296
MW	397.90
cLogP	1.98
TPSA	77.7
hM_5_ IC_50_ (nM)/ACh_min_ (%)	385/2
rM_5_ IC_50_ (nM)/ACh_min_ (%)	2105/6
predicted human CL_hep_ (mL/min/kg)	9.4
predicted rat CL_hep_ (mL/min/kg)	16
human *f*_u,plasma_	0.17
rat *f*_u,plasma_	0.12
rat *f*_u,brain_	0.16
Rat IV PK (Cassette)	
*t*_1/2_ (term) (h)	1.47
MRT (h)	1.49
Cl__obs_ (mL/min/kg)	12
Vd_ss_ (L/kg)	1.1
AUC_inf_ (hr*ng/mL)	276
*K*_p_	0.05
*K*_p,uu_	0.06

While it was not as potent compared to the parent **38**, Met-A (**43**) was active against both human and rat M_5_ (hM_5_ IC_50_ = 385 nM, ACh_min_ = 2%, rM_5_ IC_50_ = 2105 nM, ACh_min_ = 6%). In addition, **43** turned out to be peripherally restricted (*K*_p_ = 0.05, *K*_p,uu_ = 0.06). Notably, the *in vitro* clearance profile of **43** was significantly improved compared to compound **38** (predicted human CL_hep_ = 9.4; predicted rat CL_hep_ = 16 mL/min/kg). Since the hydrophobic methyl is already decorated with a polar group (OH), CYP-mediated oxidation should be bypassed and result in low *in vitro* clearance. Likewise, modulating polarity around the metabolic soft spot can be a good approach to decrease metabolism.

Compound-mediated inhibition of the hERG channel is recognized as the primary source of cardiotoxicity.^23^ Because the thiazole moiety of **38** is slightly basic, compound **38** was then tested in the cardiac panel screen at 10 μM concentration (see SI S16). Unfortunately, compound **38** inhibited the hERG channel (62.8% at 10 μM and IC_50_ of 6.5 μM) and warranting follow-up studies.

### Further Optimization of **38**

**Scheme 3:**
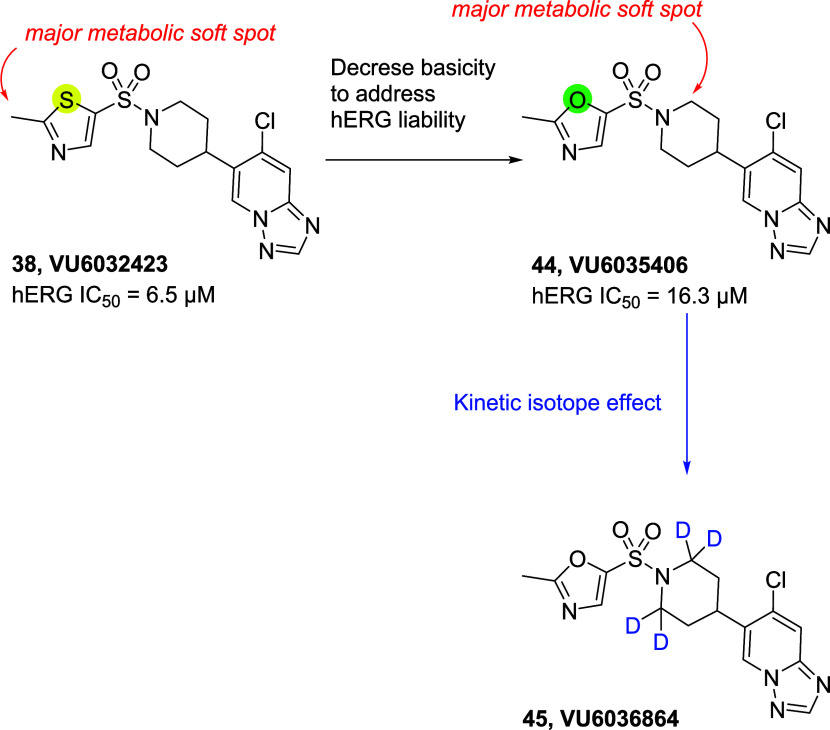
Basicity Modulation and Subsequent Deuterium Decoration of **VU6032423** (**38**)

To address the hERG issue, the molecule was fine-tuned starting from compound **38** (Scheme 3). Since a basic nitrogen atom within hydrophobic molecules tends to be recognized by hERG, the thiazole ring of **38** was replaced with an oxazole to reduce the basicity. This modification reduced hERG inhibition and unexpectedly enhanced potency, selectivity, and the DMPK profile (Table 6).

**Table 6:**
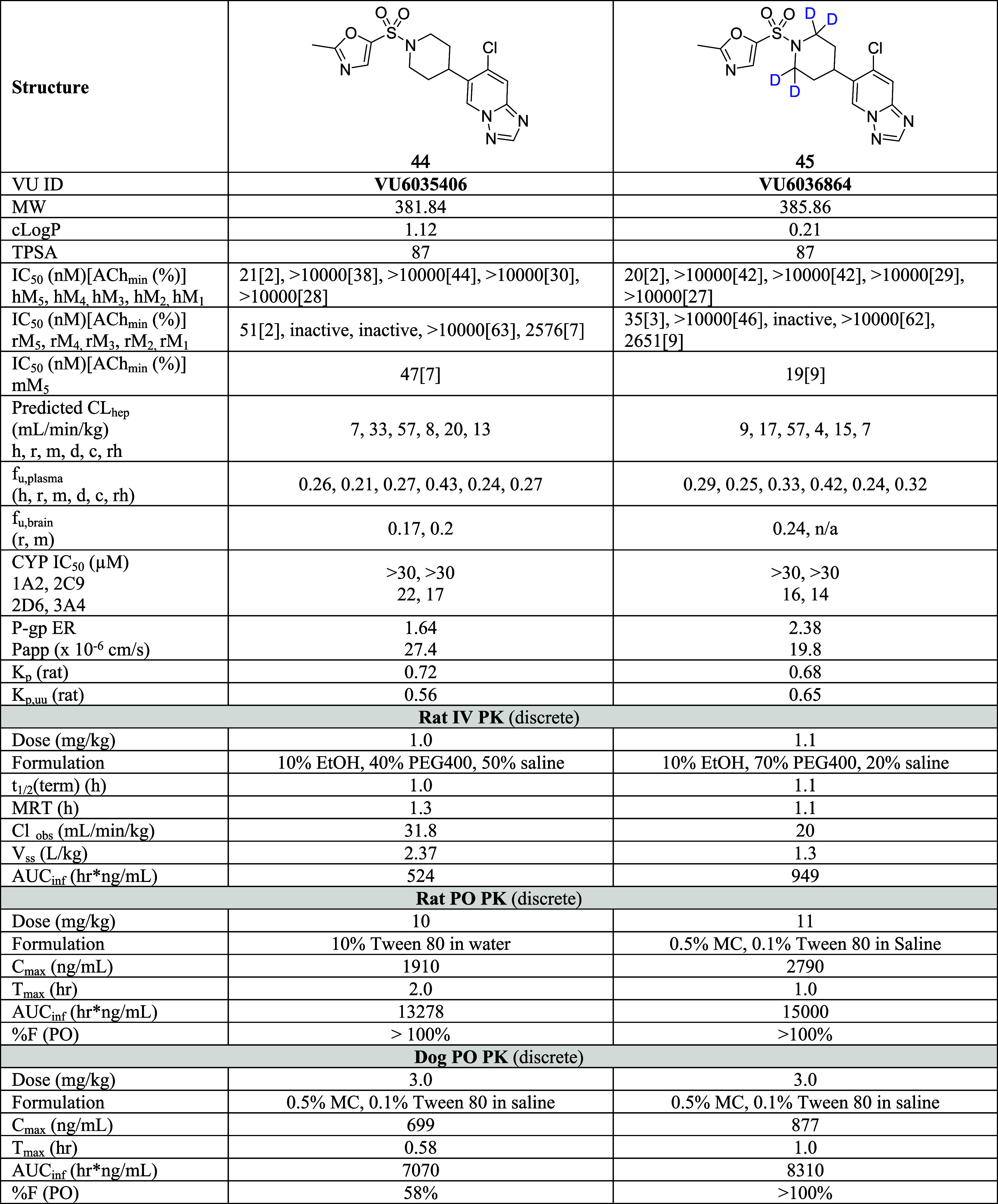
Profile Comparison of **VU6035406** (**44**) and **VU6036864** (**45**)

Compound **44** was slightly more potent and subtype-selective than **38** (hM_5_ IC_50_ = 21 nM, ACh_min_ = 2%, > 500 fold-selective against human M_1–4_) and species differences in antagonist potency were within 2-fold (rM_5_ IC_50_ = 51 nM, ACh_min_ = 2%, mM_5_ IC_50_ = 47 nM, ACh_min_ = 7%) while f_u_, CYP profiles, P-gp ER, and *K*_p_ remained comparable to **38** (Table 6 and SI S5). Compound **44** also possessed a good overall receptor subtype selectivity trend in rat (rM_1_ IC_50_ = 2576 nM rM_2_ IC_50_ > 10,000 nM, rM_3,4_ IC_50_ = inactive). In addition, *in vivo* DMPK profiles, including %F, were similar or slightly better than **38**.

Interestingly, while the majority of parameters were comparable (Table 6), the most noticeable difference between **38** and **44** was their major metabolic soft spot (Scheme 3 and SI S4). Because of slight differences in the methyl-attached 5-membered heterocycle, the metabolic soft spot was migrated from the methyl moiety to the piperidine ring (Scheme 3). With this soft spot analysis data, we tried to further improve the metabolic stability of the compound through the deuterium kinetic isotope effect. Due to the slower exchange rates of deuterium atoms, deuterated compounds were expected to have a lower metabolism rate and a longer half-life (Scheme 3). Not surprisingly, deuterium-incorporated compound **45** (**VU6036864**) retained very similar overall profiles compared to compound **44** including the receptor subtype selectivity trends in human and rat (Table 6). However, its metabolic stability and oral bioavailability were improved (**45**; human, rat, mouse, dog, cyno, and rhesus = 9, 17, 57, 4, 15, 7 mL/min/kg and %*F* > 100%). While mechanistic understanding leads to %*F* > 100% necessitate complex experiments that are beyond the scope of this manuscript, possible reasons can be found in the literature.^24,25^

All three compounds showed no time-dependent inhibition of CYP3A4 at a substrate concentration of 10 μM, and the microsomal metabolism was almost entirely dependent on CYP3A4. An apparent deuterium isotope effect was observed in the intrinsic clearance of **VU6035406** (**44**) vs **VU6036864** (**45**) (see SI S8).

Compounds **38**, **44**, and **45** were then progressed to an NHP PBL cassette study as well as the Eurofins Lead Profiling study. All three compounds showed high brain exposure in rhesus monkeys with excellent average *K*_p_ values of 1.28–1.40 (see SI S9). In addition, Eurofins Lead Profiling study results indicated that all three compounds exhibited clean ancillary pharmacology in general (see SI S10).

### Chemistry

**Scheme 4:**
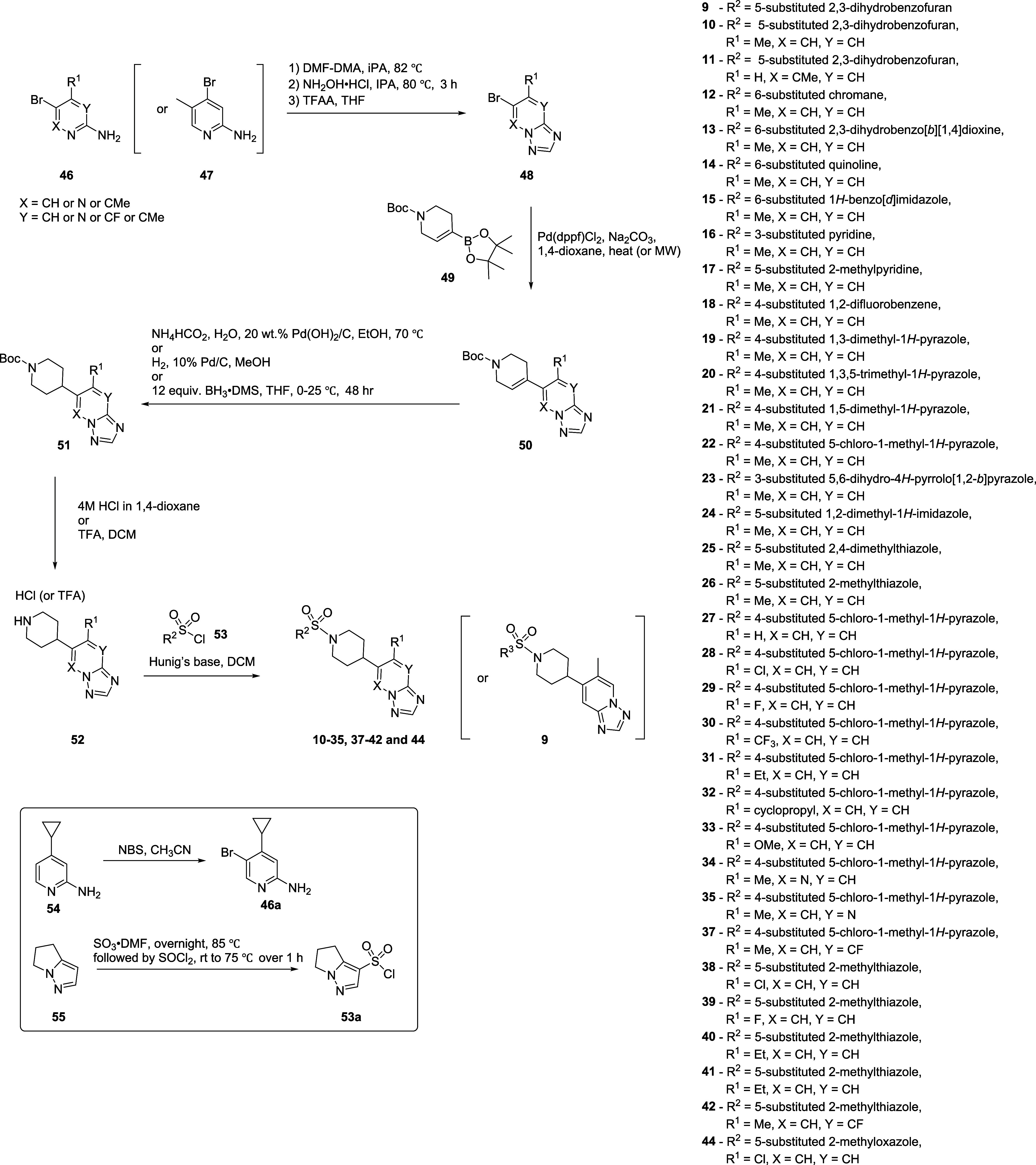
Synthesis of Azolopyridine-based M_5_ Antagonists **9**–**35**, **37**–**42**, and **44**

The general synthetic route for triazolopyridine-based M_5_ antagonists is outlined in Scheme 4. Most of the final compounds were synthesized in 7 linear steps starting from substituted 2-aminopyridine **46**. Substituted 2-aminopyridine **46** (or **47** for the triazolopyridine regioisomer **9**) was cyclized with DMF-DMA and hydroxylamine hydrochloride, followed by TFAA to form decorated triazolopyridines **48**. **48** was then coupled with pinacolborane **49** via Suzuki-Miyaura coupling. Final compounds **10**-**35**, **37**-**42**, and **44** were then assembled by olefin reduction of **50**, Boc-deprotection of **51**, followed by sulfonamide formation reactions with corresponding sulfonyl chlorides **53**.

While 2-aminopyridine starting material **46a** was obtained by reacting halogen lacking 2-aminopyridine **54** and NBS, not readily available sulfonyl chloride **53a** was prepared from compound **55** via SO_3_·DMF followed by SOCl_2_ treatments.

Although most of the steps were straightforward and high-yielding, the most challenging step was the reduction of intermediate **50**. Depending on the substituents and/or aza-decoration status of the triazolopyridine ring, different reaction patterns were observed. Slow/partial reduction, over-reduction, and dehalogenation were the most common issues. Therefore, different reduction conditions were employed to address those issues. In brief, slow reduction issues were addressed with a transfer hydrogen condition with ammonium formate. Over-reduction issues from the transfer hydrogenation reaction were addressed by switching to regular hydrogenation with H_2_. Halogen (especially Cl atom) containing intermediates were reduced using BH_3_·DMS due to dehalogenation under Pd-catalyzed reduction conditions.

**Scheme 5:**
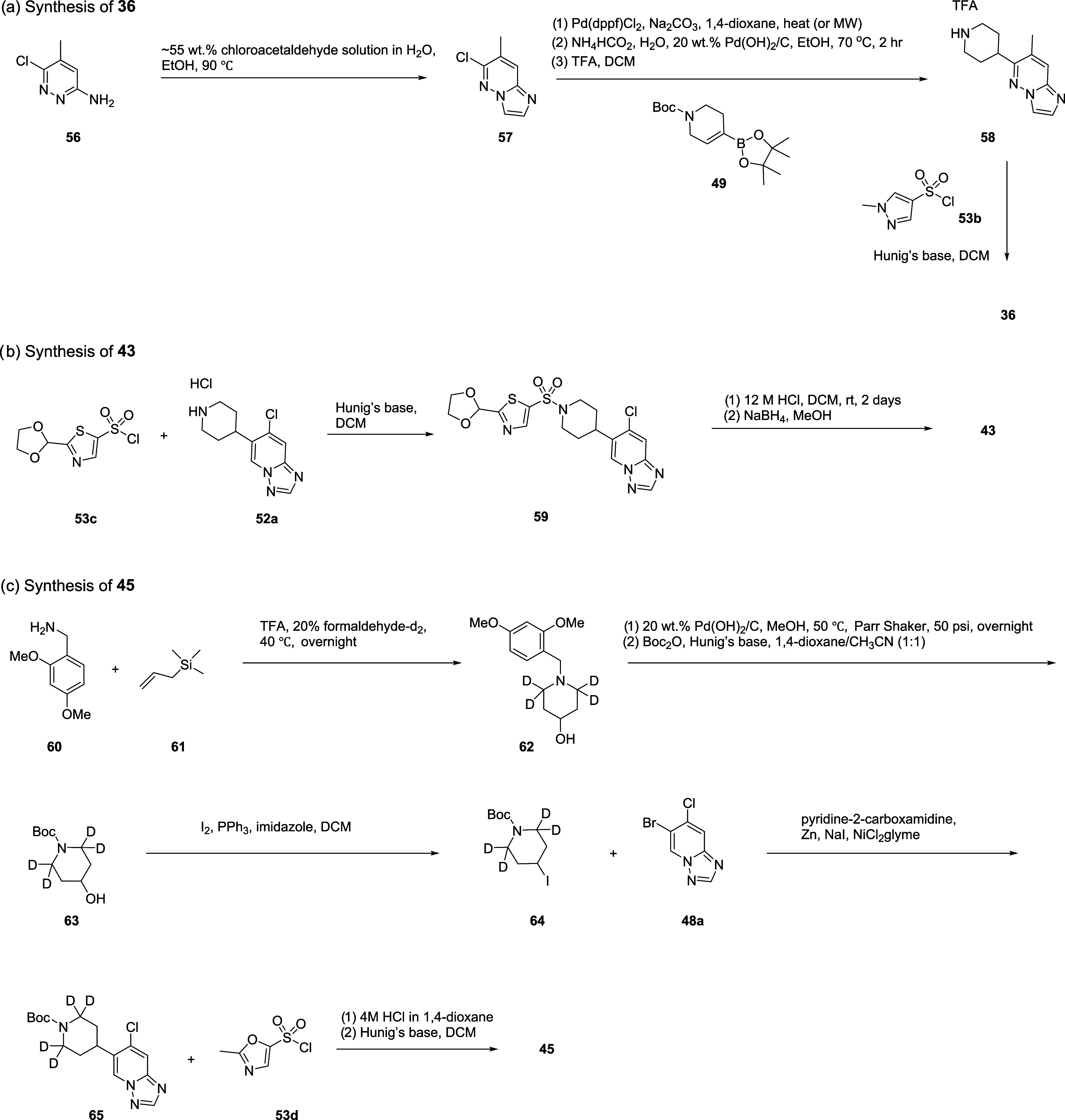
Synthesis of **36**, **43**, and **45**

As shown in Scheme 5, the imidazo[1,2-*b*]pyridazine ring of compound **36** was synthesized by reacting chloroacetaldehyde with chlorinated 2-aminopyridine **56**. Intermediate **57** in hand, the final compound **36** was synthesized via Suzuki-Miyaura coupling with **49**, olefin reduction, and Boc-deprotection, followed by sulfonamide formation reaction with **53b**.

In addition, Met-A (**43**) was synthesized using commercially available sulfonyl chloride **53c** and intermediate **52a**. Acetal deprotection under an acidic condition followed by a reduction using NaBH_4_ provided Met-A (**43**) from intermediate **59**.

Lastly, **45** (**VU6036864**) was prepared via a slightly modified synthetic route (Scheme 5). This updated route allowed us to keep the total number of steps at seven while avoiding the problematic reduction step. The tetradeuterated piperidine ring of **45** was formed starting from formaldehyde-*d*_2_, DMB amine **60**, and allyltrimethylsilane **61**. These synthons formed DMB-protected deuterated piperidin-4-ol **62** in one step. DMB protecting group was then replaced with Boc protecting group in 2 steps. The hydroxyl group of intermediate **63** was then converted to iodide via Appel reaction. Iodinated **64** was then cross-coupled with triazolopyridine **48a** via Negishi coupling. **45** (**VU6036864**) was then obtained after Boc-deprotection and sulfonamide formation reaction sequence with **53d**.

## Conclusions

In summary, a series of high-quality M_5_ orthosteric antagonists, including **45 (VU6036864)**, were discovered. This new class of high-quality tool compounds comprises a novel triazolopyridine moiety connected directly to a piperidine core. **45 (VU6036864)** and its close analogues exhibited good potency, subtype selectivity, and oral DMPK properties. Taken together, our study showcases advanced M_5_ orthosteric antagonist tool compounds that can be used for further M_5_-related studies. Follow-up studies using those tools are underway and will be reported in specialized journals. In the meantime, we hope that reported tools will provide various options for use in the field and ultimately contribute to the field of M_5_.

## Experimental Section

### General Chemistry

All reactions were carried out employing standard chemical techniques under an inert atmosphere. Solvents used for extraction, washing, and chromatography were HPLC grade. All reagents were purchased from commercial sources and were used without further purification. All microwave reactions were carried out in sealed tubes in a Biotage Initiator microwave synthesis reactor. Temperature control was automated via IR sensor and all indicated temperatures correspond to the maximal temperature reached during each experiment. Analytical HPLC was performed on an Agilent 1200 LCMS with UV detection at 215 and 254 nm along with ELSD detection and electrospray ionization, with all final compounds showing >95% purity and a parent mass ion consistent with the desired structure. Low resolution mass spectra were obtained on an Agilent 6120 or 6150 with ESI source. All NMR spectra were recorded on a 400 MHz Brüker AV-400 instrument. ^1^H chemical shifts are reported as δ values in ppm relative to the residual solvent peak (CDCl_3_ = 7.26). Data are reported as follows: chemical shift, multiplicity (br. = broad, s = singlet, d = doublet, t = triplet, q = quartet, dd = doublet of doublets, m = multiplet), coupling constant (Hz), and integration. ^13^C chemical shifts are reported as δ values in ppm relative to the residual solvent peak (CDCl_3_ = 77.16). High resolution mass spectra were obtained on an Agilent 6540 UHD Q-TOF with ESI source. Automated flash column chromatography was performed on a Teledyne ISCO Combiflash Rf system. For compounds that were purified on a Gilson preparative reversed-phase HPLC, the system comprised of a 333 aqueous pump with solvent-selection valve, 334 organic pump, GX-271 or GX-281 liquid hander, two column switching valves, and a 155 UV detector. UV wavelength for fraction collection was user-defined, with absorbance at 254 nm always monitored. Method: Phenomenex Axia-packed Luna C18, 30 × 50 mm, 5 μm column. Mobile phase: CH_3_CN in H_2_O (0.1% TFA). Gradient conditions: 0.75 min equilibration, followed by user-defined gradient (starting organic percentage, ending organic percentage, duration), hold at 95% CH_3_CN in H_2_O (0.1% TFA) for 1 min, 50 mL/min, 23 °C. Melting points were recorded on an OptiMelt automated melting point system by Stanford Research Systems. cLogP, MW, and TPSA were calculated using PerkinElmer ChemDraw professional version 20.1.0.110.

All final compounds were purified to 95% as determined by analytical LCMS (214 nm, 254 nm, and ELSD), ^1^H and/or ^13^C NMR, and high-resolution MS. **53a** was prepared according to previously reported protocols using SO_3_·DMF and SOCl_2_.^20,21^ Syntheses and/or characterization of selected intermediates as well as remaining final compounds are in the Supporting Information (SI).

### General Procedure: Triazolopyridine Synthesis

**Step 1:** Substituted 2-aminopyridine (1 equiv) was added to a round-bottom flask. iPA and DMF·DMA (1.3 equiv) were then added. The resulting mixture was heated to 82 °C for 3 h, after which time the reaction was cooled to 50 °C. NH_2_OH·HCl (1.3 equiv) was added in one portion, and the reaction was stirred at 50 °C for 2 h, after which time the reaction was cooled to room temperature and concentrated under reduced pressure. This hydroxy-formamidine crude mixture was directly used without further purification.

**Step 2**: THF (55 mL) was added to the crude mixture of hydroxy-formamidine. The resulting mixture was cooled to 0 °C. Trifluoroacetic anhydride (8 mL, 57.8 mmol, 3 equiv) was then added by syringe, and the reaction was stirred at room temperature overnight, after which time the reaction was quenched with 1 N NaOH, and then extracted with CHCl_3_/iPA solution (3:1). The combined organic extracts were concentrated and dried over Na_2_SO_4_, and solvents were filtered and concentrated. The crude residue was then purified by column chromatography (0–100% EtOAc in hexanes).

### General Procedure: Suzuki–Miyaura Cross-Coupling

Halogenated triazolo[1,5-*a*]pyridine (1 equiv), *N*-Boc-1,2,3,6-tetrahydropyridine-4-boronic acid pinacol ester (0.95 equiv), Na_2_CO_3_ (2 equiv), and Pd(dppf)Cl_2_·DCM (0.05 equiv) were added to a microwave vial, which was sealed and placed under inert atmosphere. 1,4-dioxane and H_2_O (1:1) were added via syringe, and the reaction mixture was purged with nitrogen. The resulting reaction mixture was then heated with microwave irradiation at 140 °C for 15 min, after which time the reaction mixture was filtered through Celite with EtOAc. The aqueous layer was extracted with EtOAc. The combined organic layers were dried over Na_2_SO_4_ and concentrated under reduced pressure. The reaction was purified by column chromatography (0–100% EtOAc in hexanes).

### General Procedure: Olefin Reduction—Transfer Hydrogenation

Olefin intermediate (1 equiv), Pd(OH)_2_/C (0.1 equiv), and ammonium formate (18 equiv) were added followed by EtOH. The mixture was placed under an H_2_ atmosphere. The mixture was then heated at 70 °C for 2 h, after which time the reaction was allowed to cool to room temperature, and the resulting mixture was filtered through Celite and thoroughly washed with MeOH. The filtrate was concentrated and then taken up in CH_2_Cl_2_ and H_2_O (1:1), and the aqueous layer was extracted with CH_2_Cl_2_. The combined organic layers were dried with Na_2_SO_4_, filtered, and concentrated under reduced pressure. The crude material was purified by column chromatography (0–40% of 10% MeOH with 1% NH_4_OH in CH_2_Cl_2_).

### General Procedure: Olefin Reduction—Hydrogenation

Olefin intermediate (1 equiv) was dissolved in MeOH and purged with N_2_. 10% Pd/C (0.1 equiv) was then added. The reaction mixture was stirred under H_2_ atmosphere (1 atm or Parr Shaker) overnight. The reaction mixture was then filtered through a pad of Celite which was rinsed thoroughly with MeOH and CH_2_Cl_2_. The filtrate was concentrated and purified using column chromatography (0–60% EtOAc in hexanes).

### General Procedure: Olefin Reduction—Borane Reduction

Olefin intermediate (1 equiv) was added to a round-bottomed flask, sealed with a rubber septum, and placed under an N_2_ atmosphere. THF was added, and the mixture was cooled to 0 °C, and 2 M BH_3_·DMS in THF (6 equiv) was slowly added via a syringe. After 5 min at 0 °C, the reaction was warmed to room temperature and allowed to stir overnight. (If residual starting material remains: an additional 2 M BH_3_·DMS in THF (6 equiv) was slowly added, and the reaction was stirred overnight.) The reaction was then cooled to 0 °C and quenched with 3 N NaOH (20.0 mL). The mixture was stirred at 60 °C for 3 h, after which time the reaction was concentrated under reduced pressure to remove THF, and the aqueous layer was extracted with EtOAc. The combined organic layers were washed with brine, and then dried over Na_2_SO_4_ and concentrated under reduced pressure. The crude residue was purified by column chromatography (10–100% EtOAc in hexanes).

### General Procedure: Boc Deprotection

Boc-protected intermediate (1 equiv) was dissolved in 1,4-dioxane and MeOH (5:1), and 4 M HCl in 1,4-dioxane (15 equiv) was added dropwise. The resulting mixture was stirred at room temperature for 4 h, after which time solvents were concentrated under reduced pressure. The resulting solid was used for the next step without further purification.

### General Procedure: Sulfonamide Formation

Sulfonyl chloride (1 equiv) and piperidine salt (1.2 equiv) were dissolved in CH_2_Cl_2_. To this reaction mixture, *N*,*N*-diisopropylethylamine (3 equiv) was added and stirred at room temperature for 1 h, after which time the reaction mixture was quenched with H_2_O and extracted with CH_2_Cl_2_. The combined extracts were dried over Na_2_SO_4_, filtered, and concentrated to dryness. The crude residue was then purified by column chromatography (0–20% MeOH in CH_2_Cl_2_).

#### 5-((4-(7-Chloro-[1,2,4]triazolo[1,5-*a*]pyridin-6-yl)piperidin-1-yl)sulfonyl)-2-methylthiazole (**38**; VU6032423)

**Step 1 and 2:** 5-Bromo-4-chloro-2-aminopyridine (4.00 g, 19.3 mmol, 1 equiv) was added to a round-bottom flask. iPA (64.3 mL) and DMF·DMA (3.33 mL, 25.1 mmol, 1.3 equiv) were added, and the resulting mixture was heated to 82 °C for 3 h, after which time the reaction was cooled to 50 °C. NH_2_OH·HCl (1.74 g, 25.1 mmol, 1.3 equiv) was added in one portion, and the reaction was stirred at 50 °C for 2 h, after which time the reaction was cooled to room temperature and concentrated under reduced pressure and directly used without purification.

**Step 3:** The crude mixture was redissolved in THF (55 mL), and the resulting mixture was cooled to 0 °C. TFAA (8.04 mL, 57.8 mmol, 3 equiv) was then added, and the reaction was stirred at room temperature overnight, after which time the reaction was quenched with 1 N NaOH (55 mL), and then extracted with CHCl_3_:iPA solution (3:1). The combined organic extracts were concentrated and dried over Na_2_SO_4_, and the solvents were filtered and concentrated. The crude residue was then purified by column chromatography (0–100% EtOAc in hexanes) to provide 6-bromo-7-chloro-[1,2,4]triazolo[1,5-*a*]pyridine. (3.93 g, 87% over 2 steps). ^1^H NMR (400 MHz, MeOD) δ 9.51 (s, 1 H), 8.80 (s, 1 H), 8.25 (s, 1 H); ES-MS [M + H]^+^ = 232.2.

**Step 4**: 6-Bromo-7-chloro-[1,2,4]triazolo[1,5-*a*]pyridine (3.99 g, 17.2 mmol, 1 equiv), *N*-Boc-1,2,3,6-tetrahydropyridine-4-boronic acid pinacol ester (4.78 g, 15.5 mmol, 0.95 equiv), Na_2_CO_3_ (3.71 g, 34.3 mmol, 2 equiv), and Pd(dppf)Cl_2_·DCM (0.703 g, 0.86 mmol, 0.05 equiv) were added to a microwave vial, which was sealed and placed under inert atmosphere. 1,4-dioxane (6.2 mL) and H_2_O (6.2 mL) were added via syringe, and the reaction mixture was purged with nitrogen. The resulting reaction mixture was then heated with microwave irradiation at 140 °C for 15 min, after which time the reaction mixture was filtered through Celite with EtOAc. The aqueous layer was extracted with EtOAc. The combined organic layer was dried over Na_2_SO_4_ and concentrated under reduced pressure. The reaction was purified by column chromatography (0–100% EtOAc in hexanes) to provide *tert*-butyl 4-(7-chloro-[1,2,4]triazolo[1,5-*a*]pyridin-6-yl)-3,6-dihydropyridine-1(2*H*)-carboxylate (4.075 g, 70%). ^1^H NMR (400 MHz, CDCl_3_) δ 8.42 (d, *J* = 0.7 Hz, 1 H), 8.32 (s, 1 H), 7.80 (d, *J* = 0.7 Hz, 1 H), 5.83 (bs, 1 H), 4.10 (q, *J* = 2.9 Hz, 2 H), 3.66 (t, *J* = 5.6 Hz, 2 H), 2.47 (bs, 2 H), 1.51 (s, 9 H); ES-MS [M + H]^+^ = 335.2.

**Step 5:***tert*-Butyl 4-(7-chloro-[1,2,4]triazolo[1,5-*a*]pyridin-6-yl)-3,6-dihydro-2*H*-pyridine-1-carboxylate (731 mg, 2.18 mmol, 1 equiv) was added to a round bottomed flask, sealed with a rubber septum, and placed under an N_2_ atmosphere. THF (22 mL) was added, and the mixture was cooled to 0 °C, and 2 M BH_3_·DMS in THF (6.55 mL, 13.1 mmol, 6 equiv) was slowly added via syringe. After 5 min at 0 °C, the reaction was warmed to room temperature and allowed to stir overnight, after which time an additional 2 M BH_3_·DMS in THF (6.55 mL, 13.1 mmol, 6 equiv) was slowly added via syringe, and the reaction was stirred overnight, after which time the reaction was cooled to 0 °C and quenched with 3 N NaOH (20 mL). The mixture was stirred at 60 °C for 3 h, after which time the reaction was concentrated under reduced pressure to remove THF, and the aqueous layer was extracted with EtOAc (3 × 30 mL). The combined organic layers were washed with brine, and then dried over Na_2_SO_4_ and concentrated under reduced pressure. The crude residue was purified by column chromatography (10–100% EtOAc in hexanes) to provide *tert*-butyl 4-(7-chloro-[1,2,4]triazolo[1,5-*a*]pyridin-6-yl)piperidine-1-carboxylate (346 mg, 47%). ^1^H NMR (400 MHz, CDCl_3_) δ 8.42 (d, *J* = 0.8 Hz, 1 H), 8.34 (s, 1 H), 7.86 (s, 1 H), 4.31 (bs, 2 H), 3.13 (tt, *J* = 12.1, 3.2 Hz, 1 H), 2.88 (t, *J* = 12.9 Hz, 2 H), 2.03–2.00 (m, 2 H), 1.58 (td, *J* = 12.6, 4.2 Hz, 2 H), 1.49 (s, 9H); ES-MS [M + H]^+^ = 337.3. * Residual starting material was further purified using Prep SFC. See SI for Prep SFC condition.

**Step 6:***tert*-Butyl 4-(7-chloro-[1,2,4]triazolo[1,5-*a*]pyridin-6-yl)piperidine-1-carboxylate (343 mg, 1.02 mmol, 1 equiv) was added to a vial. 4N HCl in 1,4-dioxane (8 mL, 32 mmol, 32 equiv) was added via syringe. The mixture was stirred at room temperature for 1 h, after which time the mixture was concentrated to dryness to provide 7-chloro-6-(piperidin-4-yl)-[1,2,4]triazolo[1,5-*a*]pyridine hydrochloride, which was directly used without further purification (278 mg, 99%). ES-MS [M + H]^+^ = 237.4.

**Step 7:** 2-Methylthiazole-5-sulfonyl chloride (34.7 mg, 0.18 mmol, 1.2 equiv) and 7-chloro-6-(4-piperidyl)-[1,2,4]triazolo[1,5-*a*]pyridine;hydrochloride (40 mg, 0.15 mmol, 1 equiv) were added to a vial. CH_2_Cl_2_ (1 mL) and *N*,*N*-diisopropylethylamine (80 μL, 0.44 mmol, 3 equiv) were added, and the resulting mixture was stirred at 25 °C for 30 min, after which time H_2_O (1 mL) was added to quench the reaction. The reaction mixture was passed through a phase separator. The combined organic layer was concentrated under reduced pressure. The crude residue was purified by column chromatography (0–10% MeOH in CH_2_Cl_2_) to provide the title compound (**38; VU6032423**) (47.6 mg, 81%) as a white solid. ^1^H NMR (400 MHz, CDCl_3_) δ 8.43 (s, 1H), 8.33 (s, 1H), 8.04 (s, 1H), 7.82 (s, 1H), 4.03 (d, *J* = 11.4 Hz, 2H), 2.98 (tt, *J* = 12.3, 2.9 Hz, 1H), 2.81 (s, 3H), 2.59 (td, *J* = 12.1, 2.5 Hz, 2H), 2.15 (d, *J* = 13.3 Hz, 2H), 1.84 (qd, *J* = 13.3, 12.8, 4.2 Hz, 2H); ^13^C NMR (101 MHz, DMSO) δ 172.6, 154.8, 148.9, 146.6, 136.2, 130.8, 128.9, 127.4, 115.7, 46.3 (2), 35.7, 30.3 (2), 19.4; ES-MS [M + H]^+^ = 398.0; MP: 230.2–232.1 °C; HRMS (TOF, ES^+^): [M + H]^+^ calcd for C_15_H_16_ClN_5_O_2_S_2_, 398.0507; found, 398.0504.

#### 5-((4-(7-Chloro-[1,2,4]triazolo[1,5-*a*]pyridin-6-yl)piperidin-1-yl)sulfonyl)-2-methyloxazole (**44**; VU6035406)

2-Methyloxazole-5-sulfonyl chloride (700 mg, 3.85 mmol, 1.0 equiv) and 7-chloro-6-(4-piperidyl)-[1,2,4]triazolo[1,5-*a*]pyridine;hydrochloride (1158 mg, 4.24 mmol, 1.1 equiv) were added to a round-bottom flask. CH_2_Cl_2_ (30 mL) and *N*,*N*-diisopropylethylamine (2.7 mL, 15.5 mmol, 4 equiv) were added, and the resulting mixture was stirred at 25 °C overnight. After which time the reaction mixture was quenched with sat. aq. NaHCO_3_ and extracted with CH_2_Cl_2_. The combined extracts were dried over Na_2_SO_4_, filtered, and concentrated under reduced pressure. The crude residue was purified by column chromatography (0–10% MeOH in CH_2_Cl_2_) to provide the title compound (1238 mg, 84%) as a white solid. ^1^H NMR (400 MHz, CDCl_3_) δ 8.43 (s, 1H), 8.33 (s, 1H), 7.83 (s, 1H), 7.52 (s, 1H), 4.08 (d, *J* = 12.2 Hz, 2H), 3.05 (tt, *J* = 12.1, 3.0 Hz, 1H), 2.80 (td, *J* = 12.4, 2.5 Hz, 2H), 2.59 (s, 3H), 2.15 (d, *J* = 13.1 Hz, 2H), 1.80 (qd, *J* = 12.6, 4.1 Hz, 2H); ^13^C NMR (101 MHz, CDCl_3_) δ 165.1, 155.0, 149.5, 145.0, 137.0, 133.0, 129.2, 126.1, 116.7, 46.5 (2), 36.6, 31.4 (2), 14.5; ES-MS [M + H]^+^ = 382; Melting point: 185.1–188.8 °C. HRMS (TOF, ES^+^): [M + H]^+^ calcd for C_15_H_16_ClN_5_O_3_S, 382.0735; found, 382.0728.

#### 5-((4-(7-chloro-[1,2,4]triazolo[1,5-*a*]pyridin-6-yl)piperidin-1-yl-2,2,6,6-*d*_4_)sulfonyl)-2-methyloxazole (**45**; VU6036864)

**Step 1:** Formaldehyde-*d*_2_ (20% in D_2_O) (2.2 mL, 13.8 mmol, 2.3 equiv) was mixed with 2,4-dimethoxybenzylamine (0.898 mL, 5.98 mmol, 1 equiv). To this mixture, trifluoroacetic acid (0.458 mL, 5.98 mmol, 1 equiv) was added. The resulting mixture was sonicated for 10 min and then stirred at rt for 1 h. To the resulting mixture was added allyltrimethylsilane (1.05 mL, 6.58 mmol, 1.1 equiv). This reaction mixture was stirred at 40 °C overnight. The reaction mixture was then diluted with H_2_O (10 mL) and CH_2_Cl_2_ (10 mL). Then, K_2_CO_3_ (413.3 mg, 2.99 mmol) was added and stirred for 10 min at rt. After this time, the reaction mixture was extracted with CH_2_Cl_2_, the layers were combined, dried over anhydrous Na_2_SO_4_, filtered, and evaporated. The residue was purified by silica gel chromatography using a gradient of 0–20% MeOH in CH_2_Cl_2_ as eluent to yield 1-(2,4-dimethoxybenzyl)piperidin-2,2,6,6-*d*_4_-4-ol as an oil. ^1^H NMR (400 MHz, CDCl_3_) δ 7.21 (d, *J* = 8.0 Hz, 1H), 6.48–6.43 (m, 2H), 3.80 (s, 3H), 3.78 (s, 3H), 3.65 (tt, *J* = 9.0, 4.3 Hz, 1H), 3.50 (s, 2H), 1.85 (dd, *J* = 13.0, 4.3 Hz, 2H), 1.75 (br s, 1H), 1.57 (dd, *J* = 13.0, 9.1 Hz, 2H); ES-MS [M + H]^+^ = 256.1.

**Step 2:** To a solution of 2,2,6,6-tetradeuterio-1-[(2,4-dimethoxyphenyl)methyl]piperidin-4-ol (3394.7 mg, 13.29 mmol, 1 equiv) in MeOH (40 mL) was added 20 wt % Pd(OH)_2_/C (933.5 mg, 1.33 mmol, 0.1 equiv). The reaction was charged H_2_ and stirred at 50 psi 50 °C overnight in Parr Shaker. After which time, the reaction mixture was filtered and concentrated under reduced pressure to provide piperidin-2,2,6,6-*d*_4_-4-ol (1398.2 mg, quantitative).

**Step 3:** To a solution of piperidin-2,2,6,6-*d*_4_-4-ol (1398.2 mg, 13.3 mmol, 1 equiv) in 1,4-dioxane (40 mL) and CH_3_CN (40 mL), Boc_2_O (4.6 mL, 20 mmol, 1.5 equiv) was added and stirred at rt for 3 h. The reaction mixture was then quenched with sat. aq. NaHCO_3_ (10 mL). The aqueous layer was extracted with CH_2_Cl_2_ (3 × 100 mL) after 1,4-dioxane and CH_3_CN were removed in vacuo. Combined organic layers were dried over Na_2_SO_4_ and filtered. Solvents were then concentrated. Crude was purified by silica gel chromatography using a gradient of 0–20% MeOH in CH_2_Cl_2_ as eluent to yield *tert*-Butyl 4-hydroxypiperidine-1-carboxylate-2,2,6,6-*d*_4_ (1658.9 mg, 60%); ^1^H NMR (400 MHz, CDCl_3_) δ 3.83 (tt, *J* = 8.1, 3.9 Hz, 1H), 1.83 (dd, *J* = 13.2, 4.0 Hz, 2H), 1.61 (br s, 1H), 1.47–1.41 (m, 2H), 1.45 (s, 9H); ES-MS [M + H-*t*Bu]^+^ = 150.

**Step 4:** To a solution of *N*-Boc-4-hydroxypiperdine (1474 mg, 7.2 mmol, 1 equiv) in CH_2_Cl_2_ (30 mL) was added PPh_3_ (2449 mg, 9.3 mmol, 1.3 equiv) and imidazole (733 mg, 10.8 mmol, 1.5 equiv). The resulting slurry was then cooled to 0 °C in an ice bath and I_2_ (2187 mg, 8.6 mmol, 1.2 equiv) was added in small portions. The solution was stirred for 18 h at ambient temperature. Afterward, the solution was diluted with H_2_O (20 mL) and extracted with diethyl ether (3 × 50 mL). The organic layer was washed with brine and dried over Na_2_SO_4_. Concentration in vacuo followed by trituration with CH_2_Cl_2_ and hexanes/EtOAc (8/2) mixture removed the excess PPh_3_ and TPPO. The filtrate was concentrated in vacuo and purified by silica gel chromatography using a gradient of 0–100% EtOAc in hexanes as eluent to provide *tert*-butyl 4-iodopiperidine-1-carboxylate-2,2,6,6-*d*_4_ as a colorless oil (1971 mg, 87%). ^1^H NMR (400 MHz, CDCl_3_) δ 4.45 (p, *J* = 6.0 Hz, 1H), 2.01 (d, *J* = 6.0 Hz, 4H), 1.45 (s, 9H); ^13^C NMR (101 MHz, CDCl_3_) δ 154.8, 79.9, 43.5 (m, 2), 37.3 (2), 28.6 (3), 27.9; ES-MS [M + H-*t*Bu]^+^ = 259.9.

**Step 5**: To a solution of activated Zn (1499 mg, 23 mmol, 3.6 equiv) in DMA (15 mL), was added 6-bromo-7-chloro-[1,2,4]triazolo[1,5-*a*]pyridine (1480 mg, 6.4 mmol, 1 equiv), *tert*-Butyl 4-iodopiperidine-1-carboxylate-2,2,6,6-*d*_4_ (2107 mg, 6.7 mmol,1.05 equiv), and pyridine-2-carboxamidine (154 mg, 1.27 mmol, 0.2 equiv), NiCl_2_glyme (282 mg, 1.27 mmol, 0.2 equiv) and NaI (961 mg, 6.37 mmol, 1 equiv). The mixture was then stirred at rt for 4 h under N_2_. The reaction mixture was quenched with H_2_O (10 mL) and extracted with DCM (3 × 30 mL). Organic layers were combined, dried over Na_2_SO_4_, filtered, and concentrated in vacuo. The residue was purified by silica gel chromatography using a gradient of 0–100% EtOAc in hexanes as eluent to give *tert*-butyl 4-(7-chloro-[1,2,4]triazolo[1,5-*a*]pyridin-6-yl)piperidine-1-carboxylate-2,2,6,6-*d*_4_ (596.6 mg, 27%); ^1^H NMR (400 MHz, CDCl_3_) δ 8.42 (s, 1H), 8.33 (s, 1H), 7.86 (s, 1H), 3.13 (tt, *J* = 12.2, 3.2 Hz, 1H), 2.00 (dd, *J* = 13.3, 3.3 Hz, 2H), 1.55 (t, *J* = 12.6 Hz, 2H), 1.49 (s, 9H); ES-MS [M + H]^+^ = 341.2.

**Step 6:** To a solution of *tert*-Butyl 4-(7-chloro-[1,2,4]triazolo[1,5-*a*]pyridin-6-yl)piperidine-1-carboxylate-2,2,6,6-*d*_4_ (1590 mg, 4.67 mmol, 1 equiv) in 1,4-dioxane (30 mL) and MeOH (10 mL) was added 4 N HCl in 1,4-dioxane (23.3 mL, 93.3 mmol, 20 equiv) was added. The mixture was stirred at rt for 4 h, after which time the mixture was concentrated to dryness to provide 7-chloro-6-(piperidin-4-yl-2,2,6,6-*d*_4_)-[1,2,4]triazolo[1,5-*a*]pyridine hydrochloride, which was directly used without further purification (quantitative). ES-MS [M + H]^+^ = 241.1.

**Step 7:** To a solution of 2-methyloxazole-5-sulfonyl chloride (1016.5 mg, 5.6 mmol, 1.2 equiv) in CH_2_Cl_2_ (50 mL), 7-chloro-6-(piperidin-4-yl-2,2,6,6-*d*_4_)-[1,2,4]triazolo[1,5-*a*]pyridine hydrochloride (1293 mg, 4.66 mmol, 1 equiv) and *N*,*N*-diisopropylethylamine (4.88 mL, 28 mmol, 6 equiv) were added and stirred at rt overnight. Upon completion, the reaction mixture was quenched with sat. aq. NaHCO_3_ (10 mL) and extracted with CH_2_Cl_2_ (3 × 50 mL). The combined extracts were dried over Na_2_SO_4_, filtered, and concentrated to dryness. The crude was then purified by flash column chromatography eluting 0–10% MeOH in CH_2_Cl_2_ to give 5-((4-(7-chloro-[1,2,4]triazolo[1,5-*a*]pyridin-6-yl)piperidin-1-yl-2,2,6,6-*d*_4_)sulfonyl)-2-methyloxazole (1200 mg, 66%). ^1^H NMR (400 MHz, CDCl_3_) δ 8.43 (s, 1H), 8.34 (s, 1H), 7.84 (s, 1H), 7.52 (s, 1H), 3.05 (tt, *J* = 12.4, 3.3 Hz, 1H), 2.59 (s, 3H), 2.17–2.09 (m, 2H), 1.78 (t, *J* = 12.9 Hz, 2H); ^13^C NMR (101 MHz, DMSO) δ 165.2, 154.8, 148.9, 143.9, 136.2, 132.7, 129.0, 127.4, 115.7, 45.3 (m, 2), 35.5, 30.2 (2), 14.0; ES-MS [M + H]^+^ = 386; MP: 184.3–186.8 °C, HRMS (TOF, ES^+^): [M + H]^+^ calcd for C_15_H_12_D_4_ClN_5_O_3_S, 386.0986; found, 386.0986.

#### 7-(1-((2,3-Dihydrobenzofuran-5-yl)sulfonyl)piperidin-4-yl)-6-methyl-[1,2,4]triazolo[1,5-*a*]pyridine (**9**)

Compound **9** was synthesized according to exemplary synthetic procedure (23.8 mg). ^1^H NMR (400 MHz, CDCl_3_) δ 8.34 (s, 1H), 8.25 (s, 1H), 7.65–7.55 (m, 2H), 7.53 (s, 1H), 6.89 (d, *J* = 8.3 Hz, 1H), 4.70 (t, *J* = 8.8 Hz, 2H), 4.01–3.93 (m, 2H), 3.30 (t, *J* = 8.8 Hz, 2H), 2.63 (tt, *J* = 11.3, 4.0 Hz, 1H), 2.41 (td, *J* = 11.7, 3.2 Hz, 2H), 2.31 (d, *J* = 1.0 Hz, 3H), 1.94–1.78 (m, 4H); ^13^C NMR (101 MHz, CDCl_3_) δ 164.1, 153.9, 150.0, 147.6, 129.4, 128.5, 127.6, 126.8, 125.0, 122.8, 112.5, 109.7, 72.4, 46.8 (2), 37.7, 31.7 (2), 29.2, 16.5; HRMS (TOF, ES^+^): [M + H]^+^ calcd for C_20_H_22_N_4_O_3_S, 399.1485; found, 399.1483.

#### 6-(1-((2,3-Dihydrobenzofuran-5-yl)sulfonyl)piperidin-4-yl)-7-methyl-[1,2,4]triazolo[1,5-*a*]pyridine (**10**)

Compound **10** was synthesized according to exemplary synthetic procedure (10.1 mg). ^1^H NMR (400 MHz, CDCl_3_) δ 8.39 (s, 1H), 8.34 (s, 1H), 7.68 (s, 1H), 7.62 (s, 1H), 7.59 (dd, *J* = 8.4, 2.1 Hz, 1H), 6.90 (d, *J* = 8.3 Hz, 1H), 4.71 (t, *J* = 8.9 Hz, 2H), 3.98 (d, *J* = 11.7 Hz, 2H), 3.30 (t, *J* = 8.8 Hz, 2H), 2.66 (tt, *J* = 12.1, 3.3 Hz, 1H), 2.45–2.37 (m, 2H), 2.43 (s, 3H) 1.97 (d, *J* = 13.8 Hz,, 2H), 1.83 (qd, *J* = 13.3, 12.7, 4.0 Hz, 2H); HRMS (TOF, ES^+^): [M + H]^+^ calcd for C_20_H_22_N_4_O_3_S, 399.1485; found, 399.1480.

#### 6-(1-((2,3-Dihydrobenzofuran-5-yl)sulfonyl)piperidin-4-yl)-5-methyl-[1,2,4]triazolo[1,5-*a*]pyridine (**11**)

Compound **11** was synthesized according to exemplary synthetic procedure (12.7 mg). ^1^H NMR (400 MHz, CDCl_3_) δ 8.32 (s, 1H), 7.70–7.57 (m, 3H), 7.43 (d, *J* = 9.3 Hz, 1H), 6.90 (d, *J* = 8.3 Hz, 1H), 4.71 (t, *J* = 8.8 Hz, 2H), 3.97 (dp, *J* = 11.4, 2.1 Hz, 2H), 3.31 (t, *J* = 8.8 Hz, 2H), 2.80–2.72 (m, 1H) 2.76 (s, 3H), 2.40 (td, *J* = 11.9, 2.7 Hz, 2H), 1.95 (qd, *J* = 12.9, 12.2, 4.3 Hz, 2H), 1.87–1.79 (m, 2H); HRMS (TOF, ES^+^): [M + H]^+^ calcd for C_20_H_22_N_4_O_3_S, 399.1485; found, 399.1476.

#### 6-(1-(Chroman-6-ylsulfonyl)piperidin-4-yl)-7-methyl-[1,2,4]triazolo[1,5-*a*]pyridine (**12**)

Compound **12** was synthesized according to exemplary synthetic procedure (12 mg). ^1^H NMR (400 MHz, CDCl_3_) δ 8.34 (s, 1H), 8.23 (s, 1H), 7.52–7.45 (m, 3H), 6.92–6.86 (m, 1H), 4.29–4.22 (m, 2H), 4.00–3.90 (m, 2H), 2.84 (t, *J* = 6.4 Hz, 2H), 2.61 (tt, *J* = 12.1, 3.4 Hz, 1H), 2.44–2.34 (m, 5H), 2.09–2.00 (m, 2H), 1.98–1.91 (m, 2H), 1.86–1.73 (m, 2H); ^13^C NMR (101 MHz, CDCl_3_) δ 158.9, 153.9, 149.3, 140.0, 131.2, 130.0, 127.4, 126.8, 124.8, 123.0, 117.5, 116.3, 67.1, 46.9 (2), 36.1, 32.0 (2), 25.0, 21.8, 19.8; HRMS (TOF, ES^+^): [M + H]^+^ calcd for C_21_H_24_N_4_O_3_S, 413.1642; found, 413.1636.

#### 6-(1-((2,3-Dihydrobenzo[*b*][1,4]dioxin-6-yl)sulfonyl)piperidin-4-yl)-7-methyl-[1,2,4]triazolo[1,5-*a*]pyridine (**13**)

Compound **13** was synthesized according to exemplary synthetic procedure (9.6 mg). ^1^H NMR (400 MHz, CDCl_3_) δ 8.34 (s, 1H), 8.25 (s, 1H), 7.51 (s, 1H), 7.32 (d, *J* = 2.1 Hz, 1H), 7.29 (dd, *J* = 8.4, 2.2 Hz, 1H), 7.00 (d, *J* = 8.5 Hz, 1H), 4.37–4.28 (m, 4H), 3.97 (d, *J* = 11.7 Hz, 2H), 2.63 (tt, *J* = 12.1, 3.3 Hz, 1H), 2.43 (td, *J* = 12.0, 2.5 Hz, 2H), 2.39 (s, 3H), 1.96 (d, *J* = 13.0 Hz, 2H), 1.80 (qd, *J* = 12.6, 3.9 Hz, 2H); HRMS (TOF, ES^+^): [M + H]^+^ calcd for C_20_H_22_N_4_O_4_S, 415.1435; found, 415.1432.

#### 6-((4-(7-Methyl-[1,2,4]triazolo[1,5-*a*]pyridin-6-yl)piperidin-1-yl)sulfonyl)quinoline (**14**)

Compound **14** was synthesized according to exemplary synthetic procedure (8.9 mg). ^1^H NMR (400 MHz, CDCl_3_) δ 9.10 (d, *J* = 2.5 Hz, 1H), 8.41–8.24 (m, 5H), 8.04 (dd, *J* = 8.8, 2.1 Hz, 1H), 7.59 (dd, *J* = 8.3, 4.3 Hz, 1H), 7.51 (s, 1H), 4.11 (d, *J* = 11.7 Hz, 2H), 2.61 (tt, *J* = 12.2, 3.3 Hz, 1H), 2.49 (td, *J* = 11.9, 1.9 Hz, 2H), 2.35 (s, 3H), 1.99 (d, *J* = 12.2 Hz, 2H), 1.85 (qd, *J* = 13.1, 12.6, 4.0 Hz, 2H); HRMS (TOF, ES^+^): [M + H]^+^ calcd for C_21_H_21_N_5_O_2_S, 408.1489; found, 408.1483.

#### 6-(1-((1H-Benzo[*d*]imidazol-6-yl)sulfonyl)piperidin-4-yl)-7-methyl-[1,2,4]triazolo[1,5-*a*]pyridine (**15**)

Compound **15** was synthesized according to exemplary synthetic procedure (7.3 mg). ^1^H NMR (400 MHz, MeOD) δ 8.56 (s, 1H), 8.43 (s, 1H), 8.28 (s, 1H), 8.13 (d, *J* = 1.2 Hz, 1H), 7.84 (d, *J* = 8.5 Hz, 1H), 7.75 (dd, *J* = 8.5, 1.7 Hz, 1H), 7.51 (s, 1H), 3.99 (d, *J* = 11.7 Hz, 2H), 2.74 (tt, *J* = 12.0, 3.2 Hz, 1H), 2.46 (td, *J* = 12.0, 2.5 Hz, 2H), 2.40 (s, 3H), 1.98 (d, *J* = 12.4 Hz, 2H), 1.83 (qd, *J* = 12.6, 3.9 Hz, 2H); HRMS (TOF, ES^+^): [M + H]^+^ calcd for C_19_H_20_N_6_O_2_S, 397.1441; found, 397.1436.

#### 7-Methyl-6-(1-(pyridin-3-ylsulfonyl)piperidin-4-yl)-[1,2,4]triazolo[1,5-*a*]pyridine (**16**)

Compound **16** was synthesized according to exemplary synthetic procedure (5 mg). ^1^H NMR (400 MHz, CDCl_3_) δ 9.03 (dd, *J* = 2.4, 0.8 Hz, 1H), 8.86 (dd, *J* = 4.9, 1.6 Hz, 1H), 8.35 (s, 1H), 8.24 (s, 1H), 8.09 (ddd, *J* = 8.0, 2.3, 1.6 Hz, 1H), 7.55–7.49 (m, 2H), 4.05 (dt, *J* = 11.6, 2.3 Hz, 2H), 2.69–2.60 (m, 1H), 2.47 (td, *J* = 12.1, 2.4 Hz, 2H), 2.38 (d, *J* = 1.0 Hz, 3H), 2.03–1.95 (m, 2H), 1.87–1.78 (m, 2H); ^13^C NMR (101 MHz, CDCl_3_) δ 154.1, 153.7, 149.5, 148.6, 139.8, 135.4, 133.1, 130.7, 124.8, 123.9, 116.5, 46.8 (2), 36.0, 32.0 (2), 19.8; HRMS (TOF, ES^+^): [M + H]^+^ calcd for C_17_H_19_N_5_O_2_S, 358.1332; found, 358.1327.

#### 7-Methyl-6-(1-((6-methylpyridin-3-yl)sulfonyl)piperidin-4-yl)-[1,2,4]triazolo[1,5-*a*]pyridine (**17**)

Compound **17** was synthesized according to exemplary synthetic procedure (9.5 mg). ^1^H NMR (400 MHz, CDCl_3_) δ 8.89 (dd, *J* = 2.4, 0.8 Hz, 1H), 8.34 (s, 1H), 8.25 (s, 1H), 7.96 (dd, *J* = 8.1, 2.4 Hz, 1H), 7.50 (s, 1H), 7.35 (d, *J* = 8.1 Hz, 1H), 4.07–3.98 (m, 2H), 2.67 (s, 3H), 2.62 (dt, *J* = 12.1, 3.3 Hz, 1H), 2.45 (td, *J* = 12.0, 2.4 Hz, 2H), 2.38 (d, *J* = 1.0 Hz, 3H), 2.02–1.93 (m, 2H), 1.87–1.75 (m, 2H); ^13^C NMR (101 MHz, CDCl_3_) δ 163.7, 154.1, 149.4, 148.1, 139.8, 135.7, 130.8, 130.1, 124.8, 123.5, 116.4, 46.8 (2), 36.0, 32.0 (2), 24.9, 19.8; HRMS (TOF, ES^+^): [M + H]^+^ calcd for C_18_H_21_N_5_O_2_S, 372.1489; found, 372.1482.

#### 6-(1-((3,4-Difluorophenyl)sulfonyl)piperidin-4-yl)-7-methyl-[1,2,4]triazolo[1,5-*a*]pyridine (**18**)

Compound **18** was synthesized according to exemplary synthetic procedure (10.6 mg). ^1^H NMR (400 MHz, CDCl_3_) δ 8.35 (s, 1H), 8.25 (s, 1H), 7.64 (ddd, *J* = 9.3, 7.2, 2.2 Hz, 1H), 7.58 (dddd, *J* = 8.6, 3.9, 2.2, 1.4 Hz, 1H), 7.51 (s, 1H), 7.37 (ddd, *J* = 9.6, 8.6, 7.3 Hz, 1H), 4.04–3.95 (m, 2H), 2.64 (tt, *J* = 12.1, 3.3 Hz, 1H), 2.44 (td, *J* = 12.0, 2.4 Hz, 2H), 2.39 (d, *J* = 0.9 Hz, 3H), 2.03–1.95 (m, 2H), 1.88–1.76 (m, 2H); HRMS (TOF, ES^+^): [M + H]^+^ calcd for C_18_H_18_F_2_N_4_O_2_S, 393.1191; found, 393.1185.

#### 6-(1-((1,3-Dimethyl-1*H*-pyrazol-4-yl)sulfonyl)piperidin-4-yl)-7-methyl-[1,2,4]triazolo[1,5-*a*]pyridine (**19**)

Compound **19** was synthesized according to exemplary synthetic procedure (47.9 mg). ^1^H NMR (400 MHz, CDCl_3_) δ 8.36 (s, 1H), 8.25 (s, 1H), 7.71 (s, 1H), 7.52 (s, 1H), 3.96 (d, *J* = 11.5 Hz, 2H), 3.89 (s, 3H), 2.68 (tt, *J* = 12.1, 3.3 Hz, 1H), 2.50 (td, *J* = 12.0, 2.5 Hz, 2H), 2.44 (s, 3H), 2.42 (s, 3H), 2.00 (d, *J* = 14.1 Hz, 2H), 1.83 (qd, *J* = 13.4, 12.7, 4.0 Hz, 2H); HRMS (TOF, ES^+^): [M + H]^+^ calcd for C_17_H_22_N_6_O_2_S, 375.1598; found, 375.1593.

#### 7-Methyl-6-(1-((1,3,5-trimethyl-1*H*-pyrazol-4-yl)sulfonyl)piperidin-4-yl)-[1,2,4]triazolo[1,5-*a*]pyridine (**20**)

Compound **20** was synthesized according to exemplary synthetic procedure (11.8 mg). ^1^H NMR (400 MHz, CDCl_3_) δ 8.36 (s, 1H), 8.27 (s, 1H), 7.54 (s, 1H), 3.94 (d, *J* = 11.5 Hz, 2H), 3.78 (s, 3H), 2.70 (tt, *J* = 12.1, 3.3 Hz, 1H), 2.55 (td, *J* = 12.0, 2.5 Hz, 2H), 2.49 (s, 3H), 2.43 (s, 3H), 2.41 (s, 3H), 2.02–1.95 (m, 2H), 1.80 (qd, *J* = 12.8, 3.9 Hz, 2H); HRMS (TOF, ES^+^): [M + H]^+^ calcd for C_18_H_24_N_6_O_2_S, 389.1754; found, 389.1751.

#### 6-(1-((1,5-Dimethyl-1*H*-pyrazol-4-yl)sulfonyl)piperidin-4-yl)-7-methyl-[1,2,4]triazolo[1,5-*a*]pyridine (**21**)

Compound **21** was synthesized according to exemplary synthetic procedure (15.7 mg). ^1^H NMR (400 MHz, CDCl_3_) δ 8.37 (s, 1H), 8.26 (s, 1H), 7.69 (s, 1H), 7.53 (s, 1H), 3.95 (d, *J* = 11.4 Hz, 2H), 3.86 (s, 3H), 2.66 (tt, *J* = 12.0, 3.3 Hz, 1H), 2.52 (s, 3H), 2.49–2.40 (m, 5H), 1.99 (d, *J* = 14.1 Hz, 2H), 1.83 (qd, *J* = 12.7, 3.9 Hz, 2H); HRMS (TOF, ES^+^): [M + H]^+^ calcd for C_17_H_22_N_6_O_2_S, 375.1598; found, 375.1591.

#### 6-(1-((5-Chloro-1-methyl-1*H*-pyrazol-4-yl)sulfonyl)piperidin-4-yl)-7-methyl-[1,2,4]triazolo[1,5-*a*]pyridine (**22**)

Compound **22** was synthesized according to exemplary synthetic procedure (11.5 mg). ^1^H NMR (400 MHz, CDCl_3_) δ 8.36 (s, 1H), 8.26 (s, 1H), 7.80 (s, 1H), 7.52 (s, 1H), 4.03 (d, *J* = 11.8 Hz, 2H), 3.93 (s, 3H), 2.70 (tt, *J* = 12.2, 3.3 Hz, 1H), 2.58 (td, *J* = 12.1, 11.4, 1.8 Hz, 2H), 2.42 (s, 3H), 2.00 (d, *J* = 13.4 Hz, 2H), 1.82 (qd, *J* = 12.6, 4.0 Hz, 2H); HRMS (TOF, ES^+^): [M + H]^+^ calcd for C_16_H_19_ClN_6_O_2_S, 395.1051; found, 395.1043.

#### 6-(1-((5,6-Dihydro-4*H*-pyrrolo[1,2-*b*]pyrazol-3-yl)sulfonyl)piperidin-4-yl)-7-methyl-[1,2,4]triazolo[1,5-*a*]pyridine (**23**)

Compound **23** was synthesized according to exemplary synthetic procedure (18 mg). ^1^H NMR (400 MHz, CDCl_3_) δ 8.37 (s, 1H), 8.25 (s, 1H), 7.74 (s, 1H), 7.52 (s, 1H), 4.24 (t, *J* = 7.4 Hz, 2H), 3.96 (d, *J* = 11.4 Hz, 2H), 3.12 (t, *J* = 7.5 Hz, 2H), 2.75–2.61 (m, 3H), 2.49–2.39 (m, 5H), 2.00 (d, *J* = 11.6 Hz, 2H), 1.85 (qd, *J* = 13.3, 12.7, 4.0 Hz, 2H); HRMS (TOF, ES^+^): [M + H]^+^ calcd for C_18_H_22_N_6_O_2_S, 387.1598; found, 387.1594.

#### 6-(1-((1,2-Dimethyl-1*H*-imidazol-5-yl)sulfonyl)piperidin-4-yl)-7-methyl-[1,2,4]triazolo[1,5-*a*]pyridine (**24**)

Compound **24** was synthesized according to exemplary synthetic procedure (20.8 mg). ^1^H NMR (400 MHz, CDCl_3_) δ 8.36 (s, 1H), 8.25 (s, 1H), 7.53 (s, 1H), 7.51 (s, 1H), 3.97 (d, *J* = 12.0 Hz, 2H), 3.74 (s, 3H), 2.80–2.69 (m, 3H), 2.46 (s, 3H), 2.44 (s, 3H), 2.01 (d, *J* = 12.7 Hz, 2H), 1.77 (qd, *J* = 13.4, 12.8, 4.1 Hz, 2H); HRMS (TOF, ES^+^): [M + H]^+^ calcd for C_17_H_22_N_6_O_2_S, 375.1598; found, 375.1596.

#### 2,4-Dimethyl-5-((4-(7-methyl-[1,2,4]triazolo[1,5-*a*]pyridin-6-yl)piperidin-1-yl)sulfonyl)thiazole (**25**)

Compound **25** was synthesized according to exemplary synthetic procedure (45.5 mg). ^1^H NMR (400 MHz, CDCl_3_) δ 8.37 (s, 1H), 8.27 (s, 1H), 7.55 (s, 1H), 4.03 (d, *J* = 11.6 Hz, 2H), 2.77–2.60 (m, 9H), 2.43 (s, 3H), 2.02 (d, *J* = 13.1 Hz, 2H), 1.84 (qd, *J* = 12.8, 4.1 Hz, 2H); HRMS (TOF, ES^+^): [M + H]^+^ calcd for C_17_H_21_N_5_O_2_S_2_, 392.1209; found, 392.1202.

#### 2-Methyl-5-((4-(7-methyl-[1,2,4]triazolo[1,5-*a*]pyridin-6-yl)piperidin-1-yl)sulfonyl)thiazole (**26**)

Compound **26** was synthesized according to exemplary synthetic procedure (12.6 mg). ^1^H NMR (400 MHz, CDCl_3_) δ 8.37 (s, 1H), 8.27 (s, 1H), 8.03 (s, 1H), 7.55 (s, 1H), 4.02 (d, *J* = 11.6 Hz, 2H), 2.80 (s, 3H), 2.69 (tt, *J* = 12.2, 3.2 Hz, 1H), 2.56 (td, *J* = 12.0, 2.6 Hz, 2H), 2.42 (d, *J* = 1.0 Hz, 3H), 2.03 (d, *J* = 13.0 Hz, 2H), 1.86 (qd, *J* = 12.6, 3.9 Hz, 2H); HRMS (TOF, ES^+^): [M + H]^+^ calcd for C_16_H_19_N_5_O_2_S_2_, 378.1053; found, 378.1045.

#### 6-(1-((5-Chloro-1-methyl-1*H*-pyrazol-4-yl)sulfonyl)piperidin-4-yl)-[1,2,4]triazolo[1,5-*a*]pyridine (**27**)

Compound **27** was synthesized according to exemplary synthetic procedure (12.8 mg). ^1^H NMR (400 MHz, CDCl_3_) δ 8.41 (s, 1H), 8.34 (s, 1H), 7.79 (s, 1H), 7.77 (d, *J* = 9.2 Hz, 1H), 7.42 (dd, *J* = 9.2, 1.8 Hz, 1H), 4.02 (d, *J* = 11.8 Hz, 2H), 3.92 (s, 3H), 2.69–2.53 (m, 3H), 2.02 (d, *J* = 12.0 Hz, 2H), 1.88 (qd, *J* = 13.4, 12.8, 4.1 Hz, 2H); HRMS (TOF, ES^+^): [M + H]^+^ calcd for C_15_H_17_ClN_6_O_2_S, 381.0895; found, 381.0891.

#### 7-Chloro-6-(1-((5-chloro-1-methyl-1*H*-pyrazol-4-yl)sulfonyl)piperidin-4-yl)-[1,2,4]triazolo[1,5-*a*]pyridine (**28**)

Compound **28** was synthesized according to exemplary synthetic procedure (7.1 mg). ^1^H NMR (400 MHz, CDCl_3_) δ 8.44 (s, 1H), 8.35 (s, 1H), 7.87 (s, 1H), 7.80 (s, 1H), 4.05 (d, *J* = 11.8 Hz, 2H), 3.93 (s, 3H), 2.99 (tt, *J* = 12.3, 3.3 Hz, 1H), 2.61 (td, *J* = 12.2, 2.5 Hz, 2H), 2.13 (d, *J* = 12.7 Hz, 2H), 1.81 (qd, *J* = 12.5, 4.0 Hz, 2H); HRMS (TOF, ES^+^): [M + H]^+^ calcd for C_15_H_16_Cl_2_N_6_O_2_S, 415.0505; found, 415.0501.

#### 6-(1-((5-Chloro-1-methyl-1*H*-pyrazol-4-yl)sulfonyl)piperidin-4-yl)-7-fluoro-[1,2,4]triazolo[1,5-*a*]pyridine (**29**)

Compound **29** was synthesized according to exemplary synthetic procedure (14.5 mg). ^1^H NMR (400 MHz, CDCl_3_) δ 8.40 (d, *J* = 6.5 Hz, 1H), 8.30 (s, 1H), 7.80 (s, 1H), 7.39 (d, *J* = 9.9 Hz, 1H), 4.07–3.99 (m, 2H), 3.93 (s, 3H), 2.86 (tt, *J* = 12.3, 3.4 Hz, 1H), 2.60 (td, *J* = 12.2, 2.4 Hz, 2H), 2.08 (d, *J* = 13.0 Hz, 2H), 1.87 (qd, *J* = 12.6, 4.1 Hz, 2H); HRMS (TOF, ES^+^): [M + H]^+^ calcd for C_15_H_16_ClFN_6_O_2_S, 399.0801; found, 399.0794.

#### 6-(1-((5-Chloro-1-methyl-1*H*-pyrazol-4-yl)sulfonyl)piperidin-4-yl)-7-(trifluoromethyl)-[1,2,4]triazolo[1,5-*a*]pyridine (**30**)

Compound **30** was synthesized according to exemplary synthetic procedure (5.8 mg). ^1^H NMR (400 MHz, CDCl_3_) δ 8.65 (s, 1H), 8.46 (s, 1H), 8.12 (s, 1H), 7.80 (s, 1H), 4.04 (d, *J* = 11.5 Hz, 2H), 3.95 (s, 3H), 2.92 (t, *J* = 12.2 Hz, 1H), 2.55 (td, *J* = 12.2, 2.3 Hz, 2H), 2.07 (d, *J* = 13.0 Hz, 2H), 1.89 (qd, *J* = 12.6, 3.9 Hz, 2H); HRMS (TOF, ES^+^): [M + H]^+^ calcd for C_16_H_16_ClF_3_N_6_O_2_S, 449.0769; found, 449.0763.

#### 6-(1-((5-Chloro-1-methyl-1*H*-pyrazol-4-yl)sulfonyl)piperidin-4-yl)-7-ethyl-[1,2,4]triazolo[1,5-*a*]pyridine (**31**)

Compound **31** was synthesized according to exemplary synthetic procedure (7.9 mg). ^1^H NMR (400 MHz, CDCl_3_) δ 8.41 (s, 1H), 8.33 (s, 1H), 7.81 (s, 1H), 7.65 (s, 1H), 4.04 (d, *J* = 11.8 Hz, 2H), 3.94 (s, 3H), 2.75 (q, *J* = 7.3 Hz, 3H), 2.58 (td, *J* = 12.0, 2.5 Hz, 2H), 2.00 (d, *J* = 14.1 Hz, 2H), 1.86 (qd, *J* = 13.4, 12.7, 4.0 Hz, 2H), 1.31 (t, *J* = 7.4 Hz, 3H); HRMS (TOF, ES^+^): [M + H]^+^ calcd for C_17_H_21_ClN_6_O_2_S, 409.1208; found, 409.1202.

#### 6-(1-((5-Chloro-1-methyl-1*H*-pyrazol-4-yl)sulfonyl)piperidin-4-yl)-7-cyclopropyl-[1,2,4]triazolo[1,5-*a*]pyridine (**32**)

Compound **32** was synthesized according to exemplary synthetic procedure (10.8 mg). ^1^H NMR (400 MHz, CDCl_3_) δ 8.38 (s, 1H), 8.27 (s, 1H), 7.81 (s, 1H), 7.34 (s, 1H), 4.05 (d, *J* = 11.8 Hz, 2H), 3.93 (s, 3H), 3.09 (tt, *J* = 12.2, 3.4 Hz, 1H), 2.59 (td, *J* = 12.2, 2.5 Hz, 2H), 2.08 (d, *J* = 14.0 Hz, 2H), 1.96–1.78 (m, 3H), 1.11–1.04 (m, 2H), 0.83–0.76 (m, 2H); HRMS (TOF, ES^+^): [M + H]^+^ calcd for C_18_H_21_ClN_6_O_2_S, 421.1208; found, 421.1205.

#### 6-(1-((5-Chloro-1-methyl-1*H*-pyrazol-4-yl)sulfonyl)piperidin-4-yl)-7-methoxy-[1,2,4]triazolo[1,5-*a*]pyridine (**33**)

Compound **33** was synthesized according to exemplary synthetic procedure (7.5 mg). ^1^H NMR (400 MHz, CDCl_3_) δ 8.23 (s, 1H), 8.18 (s, 1H), 7.80 (s, 1H), 6.98 (s, 1H), 4.00 (dp, *J* = 11.7, 1.9 Hz, 2H), 3.93 (s, 3H), 3.92 (s, 3H), 2.87 (tt, *J* = 12.3, 3.3 Hz, 1H), 2.58 (td, *J* = 12.1, 2.4 Hz, 2H), 2.03 (dt, *J* = 12.6, 2.5 Hz, 2H), 1.87–1.73 (m, 2H); ^13^C NMR (101 MHz, CDCl_3_) δ 159.5, 154.2, 151.0, 140.4, 129.0, 125.3, 124.5, 115.3, 94.1, 56.2, 46.7 (2), 37.3, 33.9, 31.0 (2); HRMS (TOF, ES^+^): [M + H]^+^ calcd for C_16_H_19_ClN_6_O_3_S, 411.1001; found, 411.1002.

#### 6-(1-((5-Chloro-1-methyl-1*H*-pyrazol-4-yl)sulfonyl)piperidin-4-yl)-7-methyl-[1,2,4]triazolo[1,5-*b*]pyridazine (**34**)

Compound **34** was synthesized according to exemplary synthetic procedure (11.2 mg). ^1^H NMR (400 MHz, CDCl_3_) δ 8.42 (s, 1H), 7.91 (d, *J* = 1.1 Hz, 1H), 7.81 (s, 1H), 4.02 (d, *J* = 12.2 Hz, 2H), 3.94 (s, 3H), 2.94 (tt, *J* = 11.2, 3.5 Hz, 1H), 2.67 (td, *J* = 12.1, 2.7 Hz, 2H), 2.49 (d, *J* = 1.1 Hz, 3H), 2.24–2.10 (m, 2H), 2.01 (d, *J* = 12.6 Hz, 2H); HRMS (TOF, ES^+^): [M + H]^+^ calcd for C_15_H_18_ClN_7_O_2_S, 396.1004; found, 396.0997.

#### 6-(1-((5-Chloro-1-methyl-1*H*-pyrazol-4-yl)sulfonyl)piperidin-4-yl)-5-methyl-[1,2,4]triazolo[1,5-*a*]pyrimidine (**35**)

Compound **35** was synthesized according to exemplary synthetic procedure (3.3 mg). ^1^H NMR (400 MHz, CDCl_3_) δ 8.57 (s, 1H), 8.41 (s, 1H), 7.81 (s, 1H), 4.06 (d, *J* = 11.8 Hz, 2H), 3.93 (s, 3H), 2.77 (tt, *J* = 12.0, 3.2 Hz, 1H), 2.69 (s, 3H), 2.60 (td, *J* = 12.2, 2.5 Hz, 2H), 2.05 (d, *J* = 13.5 Hz, 2H), 1.83 (qd, *J* = 12.7, 4.0 Hz, 2H); HRMS (TOF, ES^+^): [M + H]^+^ calcd for C_15_H_18_ClN_7_O_2_S, 396.1004; found, 396.0997.

#### 6-(1-((5-Chloro-1-methyl-1*H*-pyrazol-4-yl)sulfonyl)piperidin-4-yl)-7-methylimidazo[1,2-*b*]pyridazine (**36**)

Compound **36** was synthesized according to exemplary synthetic procedure (6.9 mg). ^1^H NMR (400 MHz, CDCl_3_) δ 7.83 (t, *J* = 1.0 Hz, 1H), 7.81 (s, 1H), 7.65 (d, *J* = 1.3 Hz, 1H), 7.64 (t, *J* = 1.0 Hz, 2H), 4.04–3.94 (m, 2H), 3.93 (s, 3H), 2.84 (tt, *J* = 11.4, 3.6 Hz, 1H), 2.60 (td, *J* = 12.0, 2.7 Hz, 2H), 2.36 (d, *J* = 1.1 Hz, 1H), 2.17–2.02 (m, 2H), 2.02–1.91 (m, 2H); ^13^C NMR (101 MHz, CDCl_3_) δ 156.3, 140.4, 138.8, 133.4, 129.0, 126.4, 125.0, 116.1, 115.4, 46.4 (2), 38.3, 37.3, 30.2 (2), 18.8; HRMS (TOF, ES^+^): [M + H]^+^ calcd for C_16_H_19_ClN_6_O_2_S, 395.1051; found, 395.1052.

#### 6-(1-((5-Chloro-1-methyl-1*H*-pyrazol-4-yl)sulfonyl)piperidin-4-yl)-8-fluoro-7-methyl-[1,2,4]triazolo[1,5-*a*]pyridine (**37**)

Compound **37** was synthesized according to exemplary synthetic procedure (25.5 mg). ^1^H NMR (400 MHz, CDCl_3_) δ 8.28 (s, 1H), 8.25 (s, 1H), 7.80 (s, 1H), 4.04 (d, *J* = 11.7 Hz, 2H), 3.93 (s, 3H), 2.71 (tt, *J* = 12.1, 3.3 Hz, 1H), 2.59 (td, *J* = 12.1, 2.5 Hz, 2H), 2.35 (d, *J* = 2.9 Hz, 3H), 2.01 (d, *J* = 13.6 Hz, 2H), 1.83 (qd, *J* = 13.4, 12.8, 4.1 Hz, 2H); HRMS (TOF, ES^+^): [M + H]^+^ calcd for C_16_H_18_ClFN_6_O_2_S, 413.0957; found, 413.0951.

#### 5-((4-(7-Fluoro-[1,2,4]triazolo[1,5-*a*]pyridin-6-yl)piperidin-1-yl)sulfonyl)-2-methylthiazole (**39**)

Compound **39** was synthesized according to exemplary synthetic procedure (7.2 mg). ^1^H NMR (400 MHz, CDCl_3_) δ 8.40 (d, *J* = 6.6 Hz, 1H), 8.30 (s, 1H), 8.03 (s, 1H), 7.39 (d, *J* = 9.9 Hz, 1H), 4.01 (d, *J* = 11.6 Hz, 2H), 2.85 (ddd, *J* = 16.0, 12.6, 3.6 Hz, 1H), 2.80 (s, 3H), 2.58 (td, *J* = 12.2, 2.6 Hz, 2H), 2.09 (d, *J* = 14.1 Hz, 2H), 1.90 (qd, *J* = 12.6, 4.1 Hz, 2H); HRMS (TOF, ES^+^): [M + H]^+^ calcd for C_15_H_16_FN_5_O_2_S_2_, 382.0802; found, 382.0795.

#### 5-((4-(7-Ethyl-[1,2,4]triazolo[1,5-*a*]pyridin-6-yl)piperidin-1-yl)sulfonyl)-2-methylthiazole (**40**)

Compound **40** was synthesized according to exemplary synthetic procedure (6.8 mg). ^1^H NMR (400 MHz, CDCl_3_) δ 8.39 (s, 1H), 8.27 (s, 1H), 8.04 (s, 1H), 7.58–7.54 (m, 1H), 4.02 (ddt, *J* = 11.9, 4.2, 1.9 Hz, 2H), 2.81 (s, 3H), 2.77–2.67 (m, 3H), 2.56 (td, *J* = 12.1, 2.6 Hz, 2H), 2.02 (ddd, *J* = 12.6, 4.0, 1.9 Hz, 2H), 1.88 (qd, *J* = 12.5, 3.9 Hz, 2H), 1.30 (t, *J* = 7.4 Hz, 3H); HRMS (TOF, ES^+^): [M + H]^+^ calcd for C_17_H_21_N_5_O_2_S_2_, 392.1209; found, 392.1205.

#### 2-Methyl-5-((4-(7-(trifluoromethyl)-[1,2,4]triazolo[1,5-*a*]pyridin-6-yl)piperidin-1-yl)sulfonyl)thiazole (**41**)

Compound **41** was synthesized according to exemplary synthetic procedure (4.8 mg). ^1^H NMR (400 MHz, CDCl_3_) δ 8.65 (s, 1H), 8.46 (s, 1H), 8.13 (s, 1H), 8.04 (s, 1H), 4.02 (d, *J* = 11.8 Hz, 2H), 2.96–2.86 (m, 1H), 2.82 (s, 3H), 2.54 (td, *J* = 12.1, 2.6 Hz, 2H), 2.09 (d, *J* = 13.2 Hz, 2H), 1.92 (qd, *J* = 12.5, 3.7 Hz, 2H); HRMS (TOF, ES^+^): [M + H]^+^ calcd for C_16_H_16_F_3_N_5_O_2_S_2_, 432.0770; found, 432.0766.

#### 5-((4-(8-Fluoro-7-methyl-[1,2,4]triazolo[1,5-*a*]pyridin-6-yl)piperidin-1-yl)sulfonyl)-2-methylthiazole (**42**)

Compound **42** was synthesized according to exemplary synthetic procedure (15.5 mg). ^1^H NMR (400 MHz, CDCl_3_) δ 8.29 (s, 1H), 8.25 (s, 1H), 8.04 (s, 1H), 4.03 (d, *J* = 11.7 Hz, 2H), 2.81 (s, 3H), 2.70 (tt, *J* = 12.2, 3.2 Hz, 1H), 2.57 (td, *J* = 12.0, 2.5 Hz, 2H), 2.35 (d, *J* = 2.9 Hz, 3H), 2.04 (d, *J* = 13.8 Hz, 2H), 1.86 (qd, *J* = 13.1, 12.6, 4.0 Hz, 2H); HRMS (TOF, ES^+^): [M + H]^+^ calcd for C_16_H_18_FN_5_O_2_S_2_, 396.0959; found, 396.0956.

#### (5-((4-(7-Chloro-[1,2,4]triazolo[1,5-*a*]pyridin-6-yl)piperidin-1-yl)sulfonyl)thiazol-2-yl)methanol (**43**)

Compound **43** was synthesized according to Scheme 5 (3.6 mg). ^1^H NMR (400 MHz, CDCl_3_) δ 8.43 (s, 1H), 8.33 (s, 1H), 8.14 (s, 1H), 7.82 (s, 1H), 5.05 (d, *J* = 5.9 Hz, 2H), 4.05 (d, *J* = 11.5 Hz, 2H), 2.99 (tt, *J* = 12.2, 3.1 Hz, 1H), 2.72 (t, *J* = 5.9 Hz, 1H), 2.62 (td, *J* = 12.0, 2.4 Hz, 2H), 2.14 (d, *J* = 12.9 Hz, 2H), 1.83 (qd, *J* = 12.6, 4.1 Hz, 2H); HRMS (TOF, ES^+^): [M + H]^+^ calcd for C_15_H_16_ClN_5_O_3_S_2_, 414.0456; found, 414.0451.

## Supporting Information Available

General chemistry, experimental information, and syntheses of all other compounds; in vitro and in vivo pharmacology and DMPK methods (PDF)
Molecular formula strings (CSV)

## Abbreviations

BH_3_·DMS: Borane dimethyl sulfide complex
CSF: cerebrospinal fluid
CYP: cytochrome P450
DCM: dichloromethane
DMB: dimethoxybenzyl
DMPK: drug metabolism pharmacokinetics
DMF·DMA: *N*,*N*-dimethylformamide dimethyl acetal
*f*_u_: fraction unbound
hERG: human ether-a-go-go-related gene
iPA: isopropanol
MP: melting point
MW: molecular weight
*MW*: microwave
NH_2_OH·HCl: hydroxylamine hydrochloride
P-gp ER: p-glycoprotein efflux ratio
SAR: structure–activity relationship
SI: supporting Information
TFAA: trifluoroacetic anhydride
TPPO: triphenylphosphine oxide
TPSA: total polar surface area
