# Supplementary material for: Development of VU6036864: A Triazolopyridine-Based High-Quality Antagonist Tool Compound of the M5 Muscarinic Acetylcholine Receptor
Source: J Med Chem. 2024 Aug 6;67(16):14394–413. doi: 10.1021/acs.jmedchem.4c01193 (PMC11345818; doi:10.1021/acs.jmedchem.4c01193)
Supplement: Supplementary file 1 — jm4c01193_si_001.pdf [file jm4c01193_si_001.pdf]

Supporting Information

Development of VU6036864: a triazolopyridine-based high-quality antagonist tool compound of the M_5 muscarinic acetylcholine receptor

Jinming Li, Douglas L. Orsi, Julie L. Engers, Madeline F. Long, Rory A. Capstick, Mallory A. Maurer, Christopher C. Presley, Paige N. Vinson, Alice L. Rodriguez, Allie Han, Hyekyung P. Cho, Sichen Chang, Megan Jackson, Michael Bubser, Anna L. Blobaum, Olivier Boutaud, Michael A. Nader, Colleen M. Niswender, P. Jeffrey Conn, Carrie K. Jones, Craig W. Lindsley*, and Changho Han*

Corresponding Author

Craig W. Lindsley- Warren Center for Neuroscience Drug Discovery, and Vanderbilt Institute of Chemical Biology, Vanderbilt University, Nashville, Tennessee, 37232, United States; orcid.org/0000-0003-0168-1445; Phone: 615-322-8700; Email: craig.lindsley@vanderbilt.edu; Fax: 615-343-3088

Changho Han – Department of Pharmacology, Warren Center for Neuroscience Drug Discovery, Vanderbilt University, Nashville, Tennessee 37232, United States; Email: changho.han@vanderbilt.edu; orcid.org/0000-0002-0832-7070

TABLE OF CONTENTS

Additional Available DMPK Properties of Selected M_5 Antagonists....S3

Binding affinity of VU6032423 (38) against M_1-M_4 receptor subtypes....S4

hERG channel test of VU6035406 on SyncroPatch....S5

Multispecies Metabolite Profiling and Identification....S6

Plasma and Brain Exposure of VU6035406 in SD Rat after PO Administration....S7

CYP Phenotyping Studies....S8

PBL in Rhesus after IV Administration of Cassette....S9

Eurofins Lead Profiling Screen Data....S10

FASTPatch Cardiac Channel Panel Assay....S16

Procedures for Biological Experiments....S18

General Methods....S26

Synthetic Procedures and Characterizations....S28

Compound Characterizations – ^{1}H-NMR and ^{13}C-NMR spectra....S47

HRMS data....S116

Purity analysis by LC-MS..S122
References...S134

Additional Available DMPK Properties of Selected M_5 Antagonists

Cmpd	Human (mL/min/kg)		Rat (mL/min/kg)		PPB (%f_u)		BHB (%f_u)	K_p	$K_{p,uu}$
	Cl_{int}	Cl_{hep}	Cl_{int}	Cl_{int}	Human	Rat	Rat	Rat	Rat
**Table 1**									
10	86.8	16.9	348	58.3	0.080	0.095	0.063	0.36	0.24
12	120	17.9	741	64.0	0.058	0.018	0.055	-	-
13	-	-	-	-	-	-	-	-	-
14	27.6	11.9	181	50.5	0.052	0.085	0.072	-	-
15	-	-	-	-	-	-	-	-	-
16	-	-	-	-	-	-	-	-	-
17	-	-	-	-	-	-	-	-	-
18	-	-	-	-	-	-	-	-	-
19	6.94	5.2	17.6	14.1	0.139	0.286	0.366	0.10	0.13
20	69.6	16.1	132	45.8	0.132	0.295	0.606	0.16	0.33
21	42.8	14.1	58.2	31.8	0.414	0.279	0.300	0.17	0.18
22	22.2	10.8	94.9	40.3	0.348	0.205	0.166	0.21	0.17
23	50.2	14.8	64.2	33.5	0.460	0.189	0.385	BLQ	BLQ
24	26.3	11.7	13.7	11.4	0.669	0.446	0.235	0.25	0.13
25	82.8	16.8	222	53.2	0.139	0.135	0.123	0.5	0.45
26	10	6.8	43.3	26.8	0.401	0.262	0.233	0.26	0.23
**Table 2**									
27	3.22	2.8	23.4	17.5	0.552	0.195	0.325	0.27	0.45
28	41.7	14.0	124	44.8	0.195	0.076	0.133	0.34	0.60
29	14.5	8.6	41.8	26.2	0.484	0.066	0.338	0.15	0.75
30	39.5	13.7	135	46.1	0.245	0.144	0.094	0.40	0.26
31	60	15.6	126	45.0	0.251	0.106	0.114	0.42	0.45
32	112	17.7	155	48.2	0.145	0.055	0.093	0.36	0.6
33	-	-	-	-	-	-	-	-	-
34	21.5	10.6	81.5	37.6	0.432	0.305	0.504	BLQ	BLQ
35	15.2	8.82	22.4	17	0.743	0.610	0.300	0.12	0.06
36	35.2	13.2	170	49.6	0.378	0.318	0.147	0.88	0.41
37	12.5	7.9	70.9	35.2	0.342	0.186	0.166	0.28	0.25

<u>**Binding affinity of VU6032423 (38) against M_1-M_4 receptor subtypes**</u>

- Eurofins Discovery Study #: TW04-0006986
- Significant responses (≥ 50% inhibition for biochemical assays) were highlighted in the primary assays listed below:

Cat #	Assay Name	Batch*	Spec.	Rep.	Conc.	% Inh.	IC_{50}*	K_i	n_H	R
**Compound: VU6032423-03-02, PT #: 1235297**										
252610	Muscarinic M_1	452990	hum	2	10 μM	39	>10.0 μM			
			hum	2	1 μM	2				
			hum	2	0.1 μM	4				
			hum	2	10 nM	-11				
			hum	2	1 nM	-6				
252710	Muscarinic M_2	452991	hum	2	10 μM	76	2.70 μM	1.13 μM	0.87	
			hum	2	1 μM	30				
			hum	2	0.1 μM	7				
			hum	2	10 nM	-5				
			hum	2	1 nM	-5				
252810	Muscarinic M_3	452992	hum	2	10 μM	32	>10.0 μM			
			hum	2	1 μM	7				
			hum	2	0.1 μM	6				
			hum	2	10 nM	-6				
			hum	2	1 nM	8				
252910	Muscarinic M_4	452993	hum	2	10 μM	51	9.83 μM	2.12 μM	1.01	
			hum	2	1 μM	8				
			hum	2	0.1 μM	6				
			hum	2	10 nM	4				
			hum	2	1 nM	8				

Assay: 252710 - 1 Muscarinic M_2

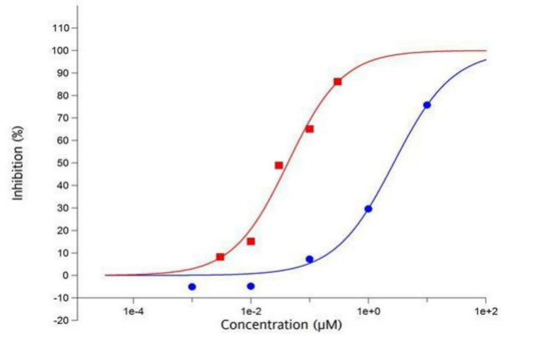

Compound Name	IC_{50}	K_i	n_H
VU6032423-03-02 (1235297)	2.70 μM	1.13 μM	0.87
4-DAMP	0.043 μM	17.9 nM	0.93

Assay: 252910 - 1 Muscarinic M_4

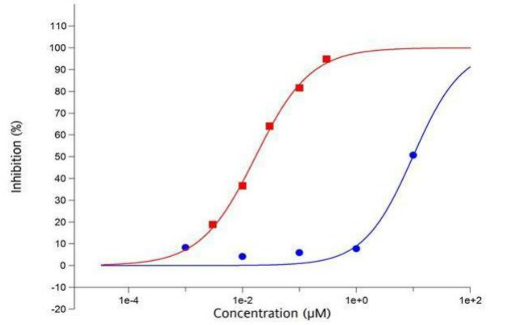

Compound Name	IC_{50}	K_i	n_H
VU6032423-03-02 (1235297)	9.83 μM	2.12 μM	1.01
4-DAMP	16.9 nM	3.65 nM	0.90

hERG channel test of VU6035406 on SyncroPatch

This study was conducted to evaluate the effects of **VU6035406** on the hERG potassium channels, using the automated patch clamp method (SyncroPatch 384PE). Amitriptyline was used as a control compound.

- WuXi App Tech Study Number: DEED-20200528

Compound ID	Conc (uM)	% Inhibition	Std Deviation	DR Curve
Amitriptyline	0.30	7.92	1.93	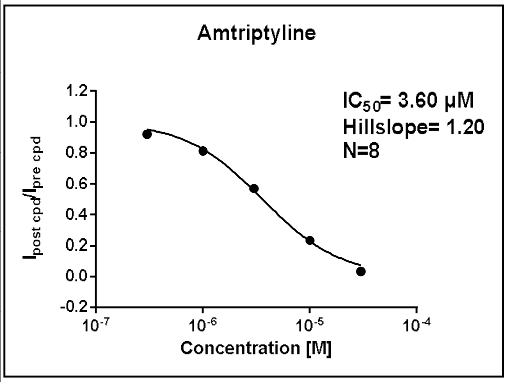
	1.00	18.90	2.51	
	3.00	43.16	4.27	
	10.00	76.78	3.34	
	30.00	96.86	1.43	

Compound ID	Conc (uM)	% Inhibition	Std Deviation	DR Curve
VU6035406-01	0.30	6.10	2.59	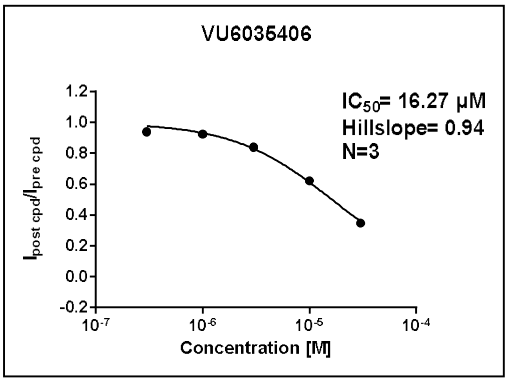
	1.00	7.55	2.24	
	3.00	16.02	5.08	
	10.00	37.79	3.23	
	30.00	65.22	1.28	

Multispecies Metabolite Profiling and Identification

VU6035406 (44)

Proposed Metabolite Structures

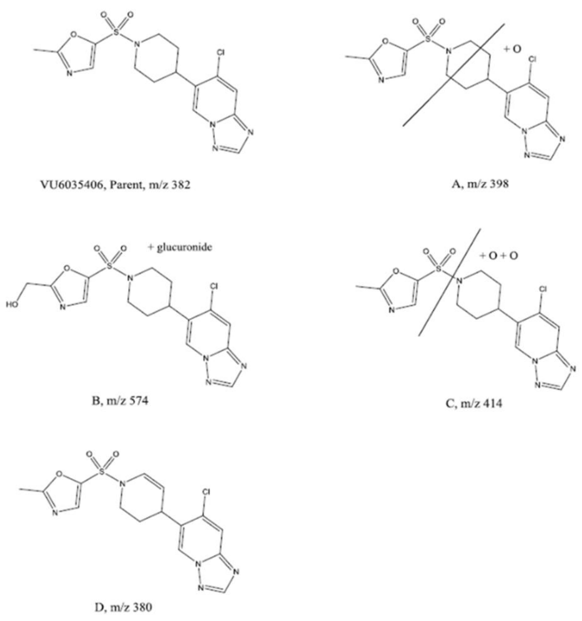

Extracted Ion Chromatograms – 4 hr Hepatocytes

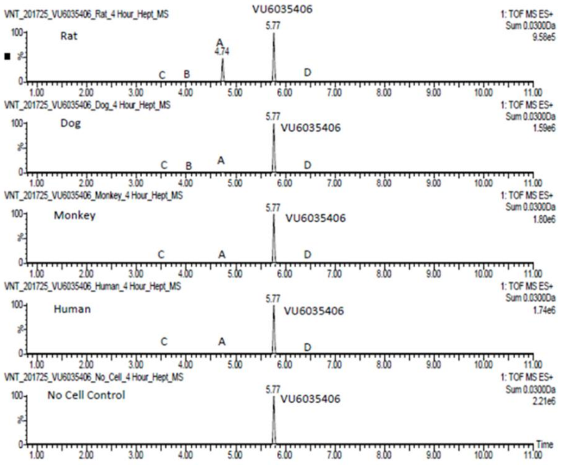

Ions monitored: 382.07+414.06+400.08+574.1+398.07+380.06+237.09+253.09+364.11+400.08+558.11+574.10+478.03+687.14 +501.08+689.16+503.09+703.14+517.07+705.15+519.09

Samples	MS Peak Areas				
	Parent	A	B	C	D
Rat	31818	12984	239	156	125
Dog	54321	1356	184	22	169
Monkey	60293	1079	ND	321	110
Human	59626	1625	ND	161	168
No Cell Control	75647	ND	ND	ND	ND

ND- Not Detected

VU6036864 (45)

Proposed Metabolite Structures

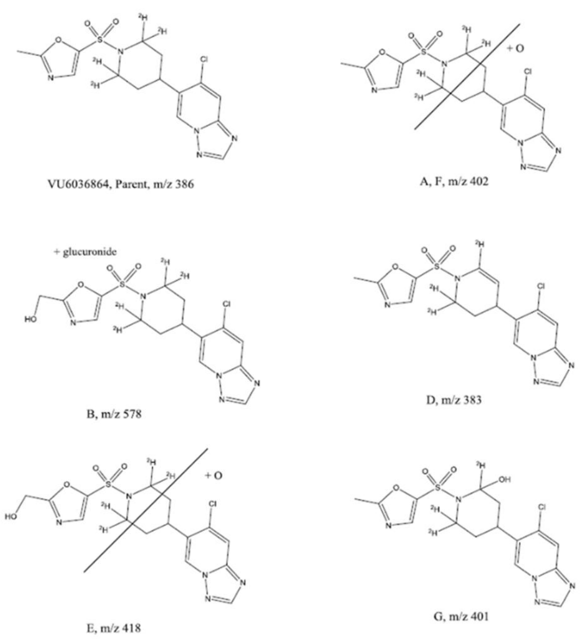

Extracted Ion Chromatograms – 4 hr Hepatocytes

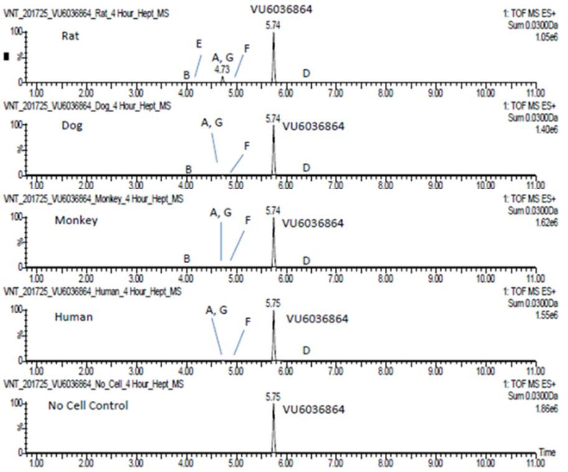

Ions monitored: 386.10+402.09+401.09+418.08+383.07+417.08+384.08+578.12+400.08+416.07+368.13+398.06+384.08+562.13 +577.11+691.17+505.10+507.12+693.18+707.16+709.18

Samples	MS Peak Areas						
	Parent	A	B	D	E	F	G
Rat	35186	4940	359	85	517	242	1947
Dog	47055	732	165	60	ND	38	226
Monkey	55011	721	26	25	ND	116	128
Human	52899	817	ND	44	ND	155	167
No Cell Control	65552	ND	ND	ND	ND	ND	ND

ND- Not Detected

Plasma and Brain Exposure of VU6035406 in SD Rat after PO Administration

- Vehicle: 10% Tween80 in water
- Dose: 10 mg/kg
- LLOQ: 0.5 ng/mL

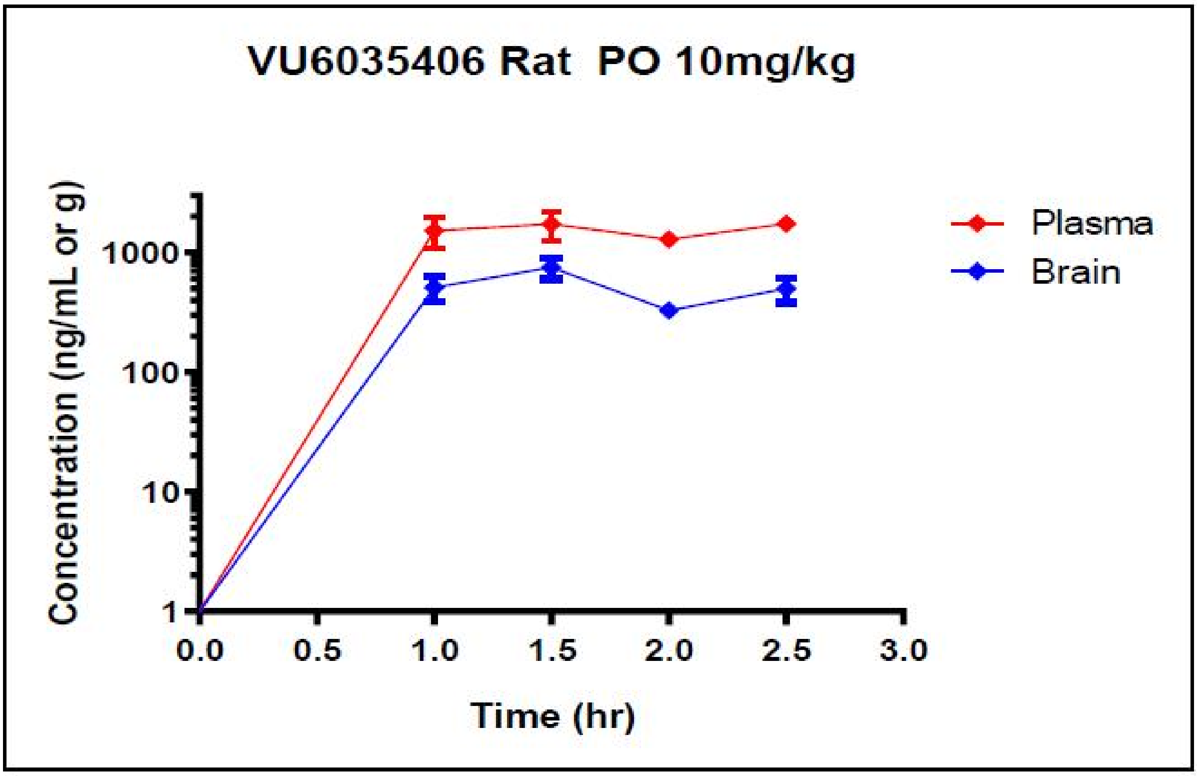

Rat PO Pharmacokinetics (n=3)								
VU#	Dose (mg/kg)	time (h)	Plasma (ng/mL)	Plasma Total (mM)	Plasma Free (nM)	Brain (ng/g)	Brain Total (µM)	Brain Free (nM)
VU6035406	10	1	1525	4	855.2	509	1.34	222
		1.5	1730	4.54	970.2	753	1.98	328.8
		2	1290	3.39	723.4	329	0.86	143.5
		2.5	1740	4.57	975.8	499	1.31	217.9

CYP Phenotyping Studies

This study was conducted to evaluate the % contribution of individual CYP enzymes to the metabolism of each compound.

		VU-6035406			VU-6032423			VU-6036864		
CYP	Abundance	CL_{int} (µL/min/pmol)	CL_{int} corrected for abundance*	Contribution (%)	CL_{int} (µL/min/pmol)	CL_{int} corrected for abundance*	Contribution (%)	CL_{int} (µL/min/ pmol)	CL_{int} corrected for abundance*	Contribution (%)
CYP1A2	0.101	Stable	Stable	Stable	Stable	Stable	Stable	Stable	Stable	Stable
CYP2B6	0.041	Stable	Stable	Stable	Stable	Stable	Stable	Stable	Stable	Stable
CYP2C8	0.058	Stable	Stable	Stable	Stable	Stable	Stable	Stable	Stable	Stable
CYP2C9	0.157	Stable	Stable	Stable	0.0364	0.00571	0.81	Stable	Stable	Stable
CYP2C19	0.028	0.0474	0.00133	0.18	0.1816	0.00509	0.72	0.0313	0.00088	0.298
CYP2D6	0.033	0.0640	0.00211	0.29	0.09	0.00304	0.432	0.0572	0.00189	0.643
CYP3A4	0.24	3.036	0.729	**98.9**	2.840	0.682	**96.9**	1.20	0.288	**98.0**
CYP3A5	0.044	0.100	0.00442	0.600	0.186	0.00819	1.2	0.074	0.00325	1.11

*Abundance: CYP1A2, 10.1%; 2B6, 4.1%; 2C8, 5.8%; 2C9, 15.7%; 2C19, 2.8%; 2D6, 3.3%; CYP3A4, 24%; 3A5, 4.4% (DMD: 2014, V 42, page 1349)

Half-life and Intrinsic Clearance of VDU Compounds after Incubation with Recombinant CYPs

CYP Isoforms	VU-6035406		VU-6032423		VU-6036864		Positive Control	
	$t_{1/2}$ (minutes)	CL_{int} (µL/min/pmol)	$t_{1/2}$ (minutes)	CL_{int} (µL/min/pmol)	$t_{1/2}$ (minutes)	CL_{int} (µL/min/pmol)	$t_{1/2}$ (minutes)	CL_{int} (µL/min/pmol)
**rCYP1A2**	Stable	Stable	Stable	Stable	Stable	Stable	18.7	0.371
**rCYP2B6**	Stable	Stable	Stable	Stable	Stable	Stable	34.2	0.203
**rCYP2C8**	Stable	Stable	Stable	Stable	Stable	Stable	4.22	16.4
**rCYP2C9**	Stable	Stable	191	0.0364	Stable	Stable	17.1	4.06
**rCYP2C19**	146.3	0.0474	38.2	0.182	221.7	0.0313	47.4	0.146
**rCYP2D6**	108.4	0.0640	75.3	0.0921	121.1	0.0572	11.0	6.3
**rCYP3A4**	2.28	3.04	2.44	2.84	5.78	1.199	24.8	1.40
**rCYP3A5**	69.0	0.100	37.2	0.186	93.8	0.074	11.8	5.88

All three compounds showed no time-dependent inhibition of CYP3A4 at a substrate concentration of 10 µM, and the microsomal metabolism of all of the compounds was almost entirely dependent on CYP3A4. An apparent deuterium isotope effect was observed in the intrinsic clearance of **VU6035406 (44)/VU6036864 (45)**.

PBL in Rhesus after IV Administration of Cassette

Animals: Male, Rhesus (n=1)
Vehicle: 10% EtOH, 40% PEG400, 50% Saline
Dose: 1 mg/kg (each compound)
Time point: 30 min
Matrix: EDTA plasma, multiple brain samples

VU6032423	Concentration (ng/mL or g) Systemic	Brain	Brain:Plasma K_p	Avg Brain (ng/g)	Avg Brain:Plasma K_p
White Matter	698.2	2419	3.46	918	1.31
Striatum		1134	1.62		
Globus pallidus		714	1.02		
Hippocampus		666	0.95		
Thalamus		531	0.76		
PFC		**	**		
Parietal Lobe		712	1.02		
Occipital Lobe		591	0.85		
Cerebellum		575	0.82		

VU6035406	Concentration (ng/mL or g) Systemic	Brain	Brain:Plasma K_p	Avg Brain (ng/g)	Avg Brain:Plasma K_p
White Matter	615.5	1862	3.03	864	1.40
Striatum		1055	1.71		
Globus pallidus		804	1.31		
Hippocampus		630	1.02		
Thalamus		582	0.95		
PFC		**	**		
Parietal Lobe		705	1.15		
Occipital Lobe		682	1.11		
Cerebellum		591	0.96		

VU6036864	Concentration (ng/mL or g) Plasma Systemic	Brain	Brain:Plasma K_p	Avg Brain (ng/g)	Avg Brain:Plasma K_p
White Matter	548.4	1127	2.06	701	1.28
Striatum		783	1.43		
Globus pallidus		686	1.25		
Hippocampus		619	1.13		
Thalamus		618	1.13		
PFC		**	**		
Parietal Lobe		654	1.19		
Occipital Lobe		624	1.14		
Cerebellum		498	0.91		

** The sample was a misinjection on mass spec. No data acquired.

Eurofins Lead Profiling Screen Data

This is a radioligand binding panel of 68 targets including GPCRs, ion channels, transporters and nuclear hormones. Biochemical assay results are presented as the percent inhibition of specific binding at 10 µM concentration of **VU6032423**.

- Eurofins Study number: TW04-0006688

LeadProfilingScreen of **VU6032423**.

Target	Species	Concentration	Replicate	% Inhibition
Adenosine A_1	hum	10 µM	2	-13
Adenosine A_{2A}	hum	10 µM	2	9
Adenosine A_3	hum	10 µM	2	-4
Adrenergic α_{1A}	hum	10 µM	2	5
Adrenergic α_{1B}	hum	10 µM	2	5
Adrenergic α_{1D}	hum	10 µM	2	-2
Adrenergic α_{2A}	hum	10 µM	2	3
Adrenergic β_1	hum	10 µM	2	5
Adrenergic β_2	hum	10 µM	2	-6
Androgen (Testosterone)	hum	10 µM	2	-18
Bradykinin B_1	hum	10 µM	2	9
Bradykinin B_2	hum	10 µM	2	-3
Calcium Channel L-Type, Benzothiazepine	rat	10 µM	2	6
Calcium Channel L-Type, Dihydropyridine	rat	10 µM	2	-10
Calcium Channel N-Type	rat	10 µM	2	-7
Cannabinoid CB_1	hum	10 µM	2	1
Dopamine D_1	hum	10 µM	2	16
Dopamine D_{2S}	hum	10 µM	2	-3
Dopamine D_3	hum	10 µM	2	-5
Dopamine $D_{4.4}$	hum	10 µM	2	31
Endothelin ET_A	hum	10 µM	2	-1
Endothelin ET_B	hum	10 µM	2	14
Epidermal Growth Factor (EGF)	hum	10 µM	2	-1
Estrogen ERα	hum	10 µM	2	-4
$GABA_A$, Flunitrazepam, Central	rat	10 µM	2	0
$GABA_A$, Muscimol, Central	rat	10 µM	2	5
$GABA_{B1A}$	hum	10 µM	2	-4
Glucocorticoid	hum	10 µM	2	-8
Glutamate, Kainate	rat	10 µM	2	10
Glutamate, NMDA, Agonism	rat	10 µM	2	-9
Glutamate, NMDA, Glycine	rat	10 µM	2	14
Glutamate, NMDA, Phencyclidine	rat	10 µM	2	5
Histamine H_1	hum	10 µM	2	-8
Histamine H_2	hum	10 µM	2	-5
Histamine H_3	hum	10 µM	2	-3
Imidazoline I_2, Central	rat	10 µM	2	44
Interleukin IL-1 R1	hum	10 µM	2	-1
Leukotriene, Cysteinyl $CysLT_1$	hum	10 µM	2	12
Melatonin MT_1	hum	10 µM	2	12
Muscarinic M_1	hum	10 µM	2	46

Muscarinic M_2	hum	10 µM	2	72
Muscarinic M_3	hum	10 µM	2	38
Neuropeptide Y Y_1	hum	10 µM	2	1
Neuropeptide Y Y_2	hum	10 µM	2	-2
Nicotinic Acetylcholine α1, Bungarotoxin	hum	10 µM	2	-1
Nicotinic Acetylcholine α3β4	hum	10 µM	2	12
Opiate δ_1 (OP1, DOP)	hum	10 µM	2	17
Opiate κ (OP2, KOP)	hum	10 µM	2	8
Opiate µ (OP3, MOP)	hum	10 µM	2	7
Phorbol Ester	mouse	10 µM	2	-18
Platelet Activating Factor (PAF)	hum	10 µM	2	10
Potassium Channel [K_{ATP}]	ham	10 µM	2	-1
Potassium Channel hERG	hum	10 µM	2	23
Prostanoid EP_4	hum	10 µM	2	-5
Purinergic P2X	rat	10 µM	2	22
Purinergic P2Y, Non-selective	rat	10 µM	2	2
Rolipram	rat	10 µM	2	9
Serotonin (5-Hydroxytryptamine) 5-HT_{1A}	hum	10 µM	2	-2
Serotonin (5-Hydroxytryptamine) 5-HT_{2B}	hum	10 µM	2	4
Serotonin (5-Hydroxytryptamine) 5-HT_3	hum	10 µM	2	-14
Sigma σ_1	hum	10 µM	2	-13
Sodium Channel, Site 2	rat	10 µM	2	18
Tachykinin NK_1	hum	10 µM	2	10
Thyroid Hormone	rat	10 µM	2	7
Transporter, Dopamine (DAT)	hum	10 µM	2	13
Transporter, GABA	rat	10 µM	2	-1
Transporter, Norepinephrine (NET)	hum	10 µM	2	9
Transporter, Serotonin (5-Hydroxytryptamine) (SERT)	hum	10 µM	2	3

- ham=Hamster; hum=Human

This is a radioligand binding panel of 68 targets including GPCRs, ion channels, transporters and nuclear hormones. Biochemical assay results are presented as the percent inhibition of specific binding at 10 µM concentration of **VU6035406**.

- Eurofins Study number: TW04-0007601

LeadProfilingScreen of **VU6035406**.

Target	Species	Concentration	Replicate	% Inhibition
Adenosine A_1	hum	10 µM	2	-9
Adenosine A_{2A}	hum	10 µM	2	-6
Adenosine A_3	hum	10 µM	2	0
Adrenergic α_{1A}	hum	10 µM	2	-5
Adrenergic α_{1B}	hum	10 µM	2	-7
Adrenergic α_{1D}	hum	10 µM	2	-13
Adrenergic α_{2A}	hum	10 µM	2	-11
Adrenergic β_1	hum	10 µM	2	1
Adrenergic β_2	hum	10 µM	2	-7
Androgen (Testosterone)	hum	10 µM	2	-3
Bradykinin B_1	hum	10 µM	2	-17
Bradykinin B_2	hum	10 µM	2	-5
Calcium Channel L-Type, Benzothiazepine	rat	10 µM	2	27
Calcium Channel L-Type, Dihydropyridine	rat	10 µM	2	-16
Calcium Channel N-Type	rat	10 µM	2	8
Cannabinoid CB_1	hum	10 µM	2	2
Dopamine D_1	hum	10 µM	2	9
Dopamine D_{2S}	hum	10 µM	2	-11
Dopamine D_3	hum	10 µM	2	-8
Dopamine $D_{4.4}$	hum	10 µM	2	21
Endothelin ET_A	hum	10 µM	2	-2
Endothelin ET_B	hum	10 µM	2	-6
Epidermal Growth Factor (EGF)	hum	10 µM	2	3
Estrogen ERα	hum	10 µM	2	-7
$GABA_A$, Flunitrazepam, Central	rat	10 µM	2	-4
$GABA_A$, Muscimol, Central	rat	10 µM	2	12
$GABA_{B1A}$	hum	10 µM	2	-13
Glucocorticoid	hum	10 µM	2	0
Glutamate, Kainate	rat	10 µM	2	-6
Glutamate, NMDA, Agonism	rat	10 µM	2	18
Glutamate, NMDA, Glycine	rat	10 µM	2	-7
Glutamate, NMDA, Phencyclidine	rat	10 µM	2	-4
Histamine H_1	hum	10 µM	2	-14
Histamine H_2	hum	10 µM	2	-11
Histamine H_3	hum	10 µM	2	-4
Imidazoline I_2, Central	rat	10 µM	2	0
Interleukin IL-1 R1	hum	10 µM	2	9
Leukotriene, Cysteinyl $CysLT_1$	hum	10 µM	2	-11
Melatonin MT_1	hum	10 µM	2	10
Muscarinic M_1	hum	10 µM	2	61
Muscarinic M_2	hum	10 µM	2	75
Muscarinic M_3	hum	10 µM	2	41
Neuropeptide Y Y_1	hum	10 µM	2	-7

Neuropeptide Y Y_2	hum	10 µM	2	9
Nicotinic Acetylcholine α1, Bungarotoxin	hum	10 µM	2	-2
Nicotinic Acetylcholine α3β4	hum	10 µM	2	14
Opiate δ_1 (OP1, DOP)	hum	10 µM	2	2
Opiate κ (OP2, KOP)	hum	10 µM	2	0
Opiate µ (OP3, MOP)	hum	10 µM	2	1
Phorbol Ester	mouse	10 µM	2	-1
Platelet Activating Factor (PAF)	hum	10 µM	2	-5
Potassium Channel [K_{ATP}]	ham	10 µM	2	-2
Potassium Channel hERG	hum	10 µM	2	11
Prostanoid EP_4	hum	10 µM	2	5
Purinergic P2X	rat	10 µM	2	10
Purinergic P2Y, Non-selective	rat	10 µM	2	6
Rolipram	rat	10 µM	2	0
Serotonin (5-Hydroxytryptamine) 5-HT_{1A}	hum	10 µM	2	-4
Serotonin (5-Hydroxytryptamine) 5-HT_{2B}	hum	10 µM	2	16
Serotonin (5-Hydroxytryptamine) 5-HT_3	hum	10 µM	2	1
Sigma σ_1	hum	10 µM	2	-11
Sodium Channel, Site 2	rat	10 µM	2	0
Tachykinin NK_1	hum	10 µM	2	25
Thyroid Hormone	rat	10 µM	2	11
Transporter, Dopamine (DAT)	hum	10 µM	2	7
Transporter, GABA	rat	10 µM	2	6
Transporter, Norepinephrine (NET)	hum	10 µM	2	3
Transporter, Serotonin (5-Hydroxytryptamine) (SERT)	hum	10 µM	2	-4

- ham=Hamster; hum=Human

This is a radioligand binding panel of 68 targets including GPCRs, ion channels, transporters and nuclear hormones. Biochemical assay results are presented as the percent inhibition of specific binding at 10 µM concentration of **VU6036864**.

Study number: TW04-0007741

LeadProfilingScreen of **VU6036864**.

Target	Species	Concentration	Replicate	% Inhibition
Adenosine A_1	hum	10 µM	2	-1
Adenosine A_{2A}	hum	10 µM	2	-2
Adenosine A_3	hum	10 µM	2	6
Adrenergic α_{1A}	hum	10 µM	2	-13
Adrenergic α_{1B}	hum	10 µM	2	-7
Adrenergic α_{1D}	hum	10 µM	2	1
Adrenergic α_{2A}	hum	10 µM	2	7
Adrenergic β_1	hum	10 µM	2	0
Adrenergic β_2	hum	10 µM	2	8
Androgen (Testosterone)	hum	10 µM	2	-16
Bradykinin B_1	hum	10 µM	2	-3
Bradykinin B_2	hum	10 µM	2	-4
Calcium Channel L-Type, Benzothiazepine	rat	10 µM	2	9
Calcium Channel L-Type, Dihydropyridine	rat	10 µM	2	-6
Calcium Channel N-Type	rat	10 µM	2	-3
Cannabinoid CB_1	hum	10 µM	2	1
Dopamine D_1	hum	10 µM	2	-8
Dopamine D_{2S}	hum	10 µM	2	1
Dopamine D_3	hum	10 µM	2	-4
Dopamine $D_{4.4}$	hum	10 µM	2	-14
Endothelin ET_A	hum	10 µM	2	-9
Endothelin ET_B	hum	10 µM	2	1
Epidermal Growth Factor (EGF)	hum	10 µM	2	-22
Estrogen ERα	hum	10 µM	2	6
$GABA_A$, Flunitrazepam, Central	rat	10 µM	2	-3
$GABA_A$, Muscimol, Central	rat	10 µM	2	15
$GABA_{B1A}$	hum	10 µM	2	3
Glucocorticoid	hum	10 µM	2	0
Glutamate, Kainate	rat	10 µM	2	18
Glutamate, NMDA, Agonism	rat	10 µM	2	-2
Glutamate, NMDA, Glycine	rat	10 µM	2	3
Glutamate, NMDA, Phencyclidine	rat	10 µM	2	-4
Histamine H_1	hum	10 µM	2	3
Histamine H_2	hum	10 µM	2	0
Histamine H_3	hum	10 µM	2	-11
Imidazoline I_2, Central	rat	10 µM	2	36
Interleukin IL-1 R1	hum	10 µM	2	5
Leukotriene, Cysteinyl $CysLT_1$	hum	10 µM	2	0
Melatonin MT_1	hum	10 µM	2	7
Muscarinic M_1	hum	10 µM	2	67
Muscarinic M_2	hum	10 µM	2	84
Muscarinic M_3	hum	10 µM	2	52
Neuropeptide Y Y_1	hum	10 µM	2	4

Neuropeptide Y Y_2	hum	10 µM	2	-1
Nicotinic Acetylcholine α1, Bungarotoxin	hum	10 µM	2	4
Nicotinic Acetylcholine α3β4	hum	10 µM	2	1
Opiate δ_1 (OP1, DOP)	hum	10 µM	2	-4
Opiate κ (OP2, KOP)	hum	10 µM	2	-7
Opiate µ (OP3, MOP)	hum	10 µM	2	-4
Phorbol Ester	mouse	10 µM	2	-7
Platelet Activating Factor (PAF)	hum	10 µM	2	-4
Potassium Channel [K_{ATP}]	ham	10 µM	2	-18
Potassium Channel hERG	hum	10 µM	2	3
Prostanoid EP_4	hum	10 µM	2	-11
Purinergic P2X	rat	10 µM	2	-9
Purinergic P2Y, Non-selective	rat	10 µM	2	-9
Rolipram	rat	10 µM	2	8
Serotonin (5-Hydroxytryptamine) 5-HT_{1A}	hum	10 µM	2	-2
Serotonin (5-Hydroxytryptamine) 5-HT_{2B}	hum	10 µM	2	9
Serotonin (5-Hydroxytryptamine) 5-HT_3	hum	10 µM	2	3
Sigma σ_1	hum	10 µM	2	-3
Sodium Channel, Site 2	rat	10 µM	2	2
Tachykinin NK_1	hum	10 µM	2	2
Thyroid Hormone	rat	10 µM	2	12
Transporter, Dopamine (DAT)	hum	10 µM	2	3
Transporter, GABA	rat	10 µM	2	-10
Transporter, Norepinephrine (NET)	hum	10 µM	2	-6
Transporter, Serotonin (5-Hydroxytryptamine) (SERT)	hum	10 µM	2	11

- ham=Hamster; hum=Human

FASTPatch Cardiac Channel Panel Assay

VU6032423 (38) was evaluated at room temperature using the QPatch HT® (Sophion Bioscience A/S, Denmark), an automatic parallel patch clamp system. The test article was evaluated at a concentration of 10 µM and tested in at least three cells (n ≥ 3). The duration of exposure to each test article concentration was at least three (3) minutes. A summary of the results for each ion channel to **VU6032423 (38)** is shown in **Tables S9a** and **S9b**.

Charles River Laboratories Study Number: 200317.WVN

Effects of VU6032423 (38) on Ion Channel Current

<table>
<tr><th>Ion Channel</th><th>Test Article ID</th><th>Conc (µM)</th><th>Mean % Inhibition</th><th>Standard Deviation</th><th>Standard Error</th><th>n</th><th>Individual Data Points (% Inhibition)</th></tr>
<tr><td rowspan="3">hCav1.2</td><td rowspan="3">VU6032423</td><td rowspan="3">10</td><td rowspan="3">3.2</td><td rowspan="3">2.6</td><td rowspan="3">1.5</td><td rowspan="3">3</td><td>5.7</td></tr>
<tr><td>3.5</td></tr>
<tr><td>0.5</td></tr>
<tr><td rowspan="6">hCav3.2</td><td rowspan="6">VU6032423</td><td rowspan="6">10</td><td rowspan="6">5.8</td><td rowspan="6">3.2</td><td rowspan="6">1.3</td><td rowspan="6">6</td><td>1.4</td></tr>
<tr><td>2.3</td></tr>
<tr><td>9.7</td></tr>
<tr><td>7.3</td></tr>
<tr><td>7.1</td></tr>
<tr><td>7.1</td></tr>
<tr><td rowspan="3">HCN2</td><td rowspan="3">VU6032423</td><td rowspan="3">10</td><td rowspan="3">3.8</td><td rowspan="3">1.3</td><td rowspan="3">0.7</td><td rowspan="3">3</td><td>3.1</td></tr>
<tr><td>2.9</td></tr>
<tr><td>5.2</td></tr>
<tr><td rowspan="7">hERG</td><td rowspan="7">VU6032423</td><td rowspan="7">10</td><td rowspan="7">62.8</td><td rowspan="7">3.4</td><td rowspan="7">1.3</td><td rowspan="7">7</td><td>67.7</td></tr>
<tr><td>60.6</td></tr>
<tr><td>62.4</td></tr>
<tr><td>60.4</td></tr>
<tr><td>60.6</td></tr>
<tr><td>60.2</td></tr>
<tr><td>67.6</td></tr>
<tr><td rowspan="3">hKv1.3</td><td rowspan="3">VU6032423</td><td rowspan="3">10</td><td rowspan="3">3.6</td><td rowspan="3">1.2</td><td rowspan="3">0.7</td><td rowspan="3">3</td><td>4.6</td></tr>
<tr><td>3.9</td></tr>
<tr><td>2.3</td></tr>
<tr><td rowspan="5">hKv1.5</td><td rowspan="5">VU6032423</td><td rowspan="5">10</td><td rowspan="5">2.0</td><td rowspan="5">8.7</td><td rowspan="5">3.9</td><td rowspan="5">5</td><td>-1.1</td></tr>
<tr><td>7.7</td></tr>
<tr><td>7.2</td></tr>
<tr><td>8.2</td></tr>
<tr><td>-12.1</td></tr>
</table>

Effects of VU6032423 (38) on Ion Channel Current

Ion Channel	Test Article ID	Conc (µM)	Mean % Inhibition	Standard Deviation	Standard Error	n	Individual Data Points (% Inhibition)
hKvLQT1/minK	VU6032423	10	8.0	12.6	6.3	4	-10.7
							16.3
							14.5
							11.8
hNav1.5 (Tonic)	VU6032423	10	6.2	3.9	1.5	7	7.7
							8.4
							10.8
							6.5
							1.4
							8.7
							0.2
hNav1.5 (Phasic)	VU6032423	10	10.3	4.0	1.5	7	14.0
							12.8
							11.8
							12.3
							11.6
							6.9
							2.8

Procedures for Biological Experiments

Calcium mobilization assays:

To measure the functional activity of antagonist compounds in a cellular assay, human or rat muscarinic receptor subtype 5 (M_5) was stably expressed in the Chinese hamster ovary (CHO) cells to evoke a decrease in intracellular calcium to an EC_{80} concentration of acetylcholine (ACh) agonist. The stable M_5-CHO cells were cultured in F12 medium containing 10% fetal bovine serum, 20 mM HEPES, 0.5 mg/ml G418, and antibiotics/antimycotic. All reagents used were from Life Technologies (Carlsbad, CA) unless otherwise noted. The muscarinic isoform selectivity of the compounds was also determined in the Ca mobilization assay using CHO cells stably expressing human and rat M_1 and M_3. Their activities at M_2 and M_4 receptors were also measured in CHO cells stably co-expressing chimeric G_{qi5} proteins.

Briefly, the day before the assay, stable M_5-CHO cells (15,000 cells/20 µL/well) were plated in black-walled, clear-bottomed, 384 well plates (Greiner Bio-One, Monroe, NC) in culture medium without G418, and then incubated overnight at 37 °C in the presence of 5% CO_2. The next day, calcium assay buffer (Hank's balanced salt solution (HBSS), 20 mM HEPES, 2.5 mM Probenecid, 4.16 mM sodium bicarbonate (Sigma-Aldrich, St. Louis, MO)) was prepared to dilute compounds, agonists, and Fluo-4-acetomethoxyester (Fluo-4-AM, Ion Biosciences), fluorescent calcium indicator dye. Compounds were serially diluted 1:3 into 10 point concentration response curves in DMSO using the Bravo Liquid Handler (Agilent, Santa Clara, CA), transferred to a 384 well daughter plate using an Echo acoustic liquid handler (Beckman Coulter, Indianapolis, Indiana), and diluted in assay Buffer to a 2X final concentration. The agonist plates were prepared using acetylcholine (ACh, Sigma-Aldrich, St. Louis, MO) concentrations for the EC_{20}, EC_{80}, and EC_{MAX} responses by diluting in assay buffer to a 5X final concentration. The 2X dye solution (2.3 µM) was prepared by mixing a 2.3 mM Fluo-4-AM stock in DMSO with 10% (w/v) pluronic acid F-127 in a 1:1 ratio in assay buffer. Using a microplate washer (BioTek, Winooski, VT), cells were washed with assay buffer 3 times to remove medium. After the final wash, 20 µL of assay buffer remained in the cell plates. Immediately, 20 µL of the 2X dye solution (final 1.15 µM) was added to each well of the cell plate using a Multidrop Combi dispenser (Thermo Fisher, Waltham, MA). After cells were incubated with the dye solutions for 45 min at 37 °C in the presence of 5% CO_2, the dye solutions were removed and replaced with assay buffer using a microplate washer, leaving 20 µL of assay buffer in the cell plate. The compound, agonist, and cell plates were placed inside the Functional Drug Screening System (FDSS 7000 or uCell, Hamamatsu, Japan) to measure the calcium flux. Briefly, after establishment of a fluorescence baseline for 2 seconds (excitation, 480 nm; emission, 530 nm), 20 µL of test compound or vehicle was added to the cells, and the response was measured. =140 seconds later, 10 µL (5X) of an EC_{20} concentration of ACh agonist was added to the cells, and the response of the cells was measured. 125 seconds later, 12 µl (5X) of an EC_{80} concentration of ACh agonist and the response of the cells was measured for 90 seconds.

Calcium fluorescence was recorded as fold over basal fluorescence and raw data were normalized to the maximal response to ACh agonist (EC_{Max}). Compound-evoked decrease in calcium response in the presence of ACh EC_{80} agonist was determined as inhibition activity of antagonist, and potency (IC_{50}) and maximum inhibition response (% ACh min) of compounds were determined using a four-parameter logistical equation using GraphPad Prism (La Jolla, CA) or the Dotmatics software platform (Woburn, MA) :

$$y = bottom + \frac{top - bottom}{1 + 10^{(LogEC50 - A)Hillslope}}$$

where *A* is the molar concentration of the compound; *bottom* and *top* denote the lower and upper plateaus of the concentration-response curve; HillSlope is the Hill coefficient that describes the steepness of the curve; and EC_{50} is the molar concentration of compound required to generate a response halfway between the *top* and *bottom*.

Radioligand Binding Assays:

Binding assays were performed by Eurofins Panlabs Discovery Services (New Taipei City, Taiwan) to evaluate the activity of compound VU6032423. In brief, compounds were incubated with cell membrane (Human recombinant CHO-K1 cells) and 0.80 nM [^{3}H] N-Methylscopolamine for 2 hours at 25 °C in assay buffer (50 mM Tris-HCl, pH 7.4, 10 mM $MgCl_2$, 1 mM EDTA). Atropine was used to determine non-specific binding. Results are presented as the percent inhibition of specific binding. Muscarinic receptor antagonist 4-DAMP was utilized as a positive control. IC_{50} values were determined by a non-linear, least squares regression analysis using MathIQTM (ID Business Solutions Ltd., UK). K_i values were calculated using the equation of Cheng and Prusoff (Cheng, Y., Prusoff, W.H., Biochem. Pharmacol. 22:3099-3108, 1973) using the observed IC_{50} of the tested compound, the concentration of radioligand employed in the assay, and the historical values for the K_D of the ligand (1.30 nM, obtained experimentally at Eurofins Panlabs, Inc.).

Plasma protein binding and Brain homogenate binding:

The protein binding of each compound was determined in plasma via equilibrium dialysis employing rapid equilibrium dialysis (RED) plates (ThermoFisher Scientific, Rochester, NY). Plasma was added to the 96 well plate containing test compound and mixed thoroughly for a final concentration of 5 µM. Subsequently, an aliquot of the plasma-compound mixture was transferred to the *cis* chamber (red) of the RED plate, with a phosphate buffer (25 mM, pH 7.4) in the *trans* chamber. The RED plate was sealed and incubated for 6 hours at 37 °C with shaking (120 rpm). At completion, aliquots from each chamber

were transferred to a new 96 well plate and were diluted 1:1 with either plasma (*trans*) or buffer (*cis*), at which time ice-cold acetonitrile containing internal standard (50 nM carbamazepine) (3 volumes) was added to extract the matrices. The plate was centrifuged (3000 RCF, 10 min) and supernatants were transferred and diluted 1:1 (supernatant: water) into a new 96 well plate, which was then sealed in preparation for LC/MS/MS analysis. Each compound was assayed in triplicate within the same 96-well plate.

A similar approach was used to determine the degree of brain homogenate binding, which employed the same methodology and procedure with the following modifications: 1) a final compound concentration of 1 µM was used, 2) naïve rat brains were homogenized in DPBS (1:3 composition of brain: DPBS, w/w) using a Mini-Bead Beater™ machine in order to obtain brain homogenate, which was then treated in the same manner as the plasma samples in the previously described plasma protein binding assay.

A similar approach was used to determine the degree of brain homogenate binding, which employed the same methodology and procedure with the following modifications: 1) a final compound concentration of 1 µM was used, 2) naïve rat brains were homogenized in DPBS (1:3 composition of brain: DPBS, w/w) using a Mini-Bead Beater™ machine in order to obtain brain homogenate, which was then treated in the same manner as the plasma samples in the previously described plasma protein binding assay. Fraction unbound for both plasma and brain samples was determined using Equation 4.

$$f_u = \frac{Conc_{buffer}}{Conc_{plasma}}$$

Equation 4 Determination of fraction unbound in plasma.

The diluted fraction unbound (f_u2) in brain was calculated in the same manner by using brain homogenate rather than plasma. Undiluted fraction unbound for the brain was calculated using Equation 5

$$f_u = \frac{1/4}{\left\{\left(\frac{1}{fu2}\right) - 1\right\} + 1/4}$$

Equation 5 Determination of fraction unbound in brain. F_u2 represents the diluted fraction unbound.

Intrinsic clearance:

Human or rat hepatic microsomes (0.5 mg/mL) and 1 µM test compound were incubated in 100 mM potassium phosphate pH 7.4 buffer with 3 mM $MgCl_2$ at 37 °C with constant shaking. After a 5 min preincubation, the reaction was initiated by the addition of NADPH (1 mM). At selected time intervals (0, 3, 7, 15, 25, and 45 min), aliquots were taken and subsequently placed into a 96-well plate containing cold acetonitrile with internal standard (50 ng/mL carbamazepine). Plates were then centrifuged at 3000 rcf (4

°C) for 10 min, and the supernatant was transferred to a separate 96-well plate and diluted 1:1 with water for LC/MS/MS analysis. The *in vitro* half-life ($_{1/2}$, min, Eq. 1), intrinsic clearance (CL_{int}, mL/min/kg, Eq. 2), and subsequent predicted hepatic clearance (CL_{hep}, mL/min/kg, Eq. 3) was determined employing the following equations:

(1) $T_{1/2} = \frac{Ln(2)}{K}$

where k represents the slope from linear regression analysis of the natural log percent remaining of a test compound as a function of incubation time

(2) $CL_{int} = \frac{0.693}{in\ vitro T_{1/2}}\ x\ \frac{mL\ incubation}{mg\ microsomes}\ x\ \frac{45\ mg\ microsomes}{gram\ liver}\ x\ \frac{20^{a}\ gram\ liver}{kg\ body\ wt}$

^{a}scale-up factors: of 20 (human) or 45 (rat)

(3) $CL_{hep} = \frac{Q_h \cdot CL_{int}}{Q_h + CL_{int}}$

where Q_h (hepatic blood flow, mL/min/kg) is 21 (human) or 70 (rat).

LC/MS/MS Bioanalysis of Samples from Plasma Protein Binding and Intrinsic Clearance Assays: Samples were analyzed on a Thermo Electron TSQ Quantum Ultra triple quad mass spectrometer (San Jose, CA) via electrospray ionization (ESI) with two Themo Electron Accella pumps (San Jose, CA), and a Leap Technologies CTC PAL autosampler (Carrboro, NC). Analytes were separated by gradient elution on a dual column system with two Thermo Hypersil Gold (2.1 x 30 mm, 1.9 µm) columns (San Jose, CA) thermostated at 40 °C. HPLC mobile phase A was 0.1% formic acid in water and mobile phase B was 0.1% formic acid in acetonitrile. The gradient started at 10% B after a 0.2 min hold and was linearly increased to 95% B over 0.8 min; hold at 95% B for 0.2 min; returned to 10% B in 0.1 min. The total run time was 1.3 min and the HPLC flow rate was 0.8 mL/min. While pump 1 ran the gradient method, pump 2 equilibrated the alternate column isocratically at 10% B. Compound optimization, data collection, and processing was performed using Thermo Electron's QuickQuan software (v2.3) and Xcalibur (v2.0.7 SP1).

***In vivo* DMPK experimental:**

Animal care and use:

All animal study procedures were approved by the Vanderbilt University Institutional Animal Care and Use Committee and were conducted in accordance with the National Institutes of Health regulations of animal care covered in Principles of Laboratory Animal Care (National Institutes of Health).

In-life phase

Compounds were formulated as a solution in ethanol, PEG400, and saline (1:4:5 v/v, respectively) and administered as a single IV dose (1 mL/kg) to male, Sprague Dawley rats (*n* = 1-2) via injection into a surgically-implanted jugular vein catheter. Blood samples were collected serially from a surgically implanted carotid artery catheter in each animal over multiple post-administration time points (0.033, 0.117, 0.25, 0.5, 1, 2, 4, 7, and 24 hours) into chilled, K_2EDTA anticoagulant-fortified tubes and immediately placed on wet ice. The blood samples were then centrifuged (1700 rcf, 5 minutes, 4 ˚C) in order to obtain plasma samples, which were stored at -80 ˚C until analysis by LC-MS/MS.

For determination of the brain over plasma ratio (Kp), compounds were formulated in 8% ethanol, 32% PEG400 and 60% DMSO (v/v/v) and administered as a single 0.2 mg/kg IV dose (1 mL/kg) to male, Sprague Dawley rats (*n* = 1; 316 gram body weights) via injection into a surgically-implanted jugular vein catheter. At 15 min post dosing, blood sample was collected serially (i.e., terminally) into chilled, K_2EDTA anticoagulant-fortified tube and immediately placed on wet ice. The blood sample was then centrifuged (1700 rcf, 5 minutes, 4 ˚C) to obtain plasma sample. At the same post-administration time point, whole brain sample was obtained by rapid dissection, rinsed with PBS, and immediately frozen in individual tissue collection box (dry ice). All brain and plasma samples were stored at -80 ˚C until analysis by LC-MS/MS.

For PO PK studies, compounds were formulated in 10% Tween 80 in water and administered orally as a single dose of 10 mg/kg to male Sprague Dawley rats (10 ml/kg, n=2). Blood samples were collected serially from a surgically implanted carotid artery catheter in each animal over multiple post-administration time points (0.25, 0.5, 1, 2, 4, 7, and 24 hours) into chilled, K_2EDTA anticoagulant-fortified tubes and immediately placed on wet ice. The blood

samples were then centrifuged (1700 rcf, 5 minutes, 4 ˚C) in order to obtain plasma samples, which were stored at -80 ˚C until analysis by LC-MS/MS.

For determination of the brain over plasma ratio (Kp), compounds were formulated in ethanol, PEG400, and saline (1:7:2 v/v, respectively) and administered as a single IV dose (2 mL/kg) to male rhesus monkey (*n* = 1) via injection into a surgically-implanted jugular vein catheter. At 30 min post dosing, blood sample was collected terminally into chilled, K_2EDTA anticoagulant-fortified tube and immediately placed on wet ice. The blood sample was then centrifuged (1700 rcf, 5 minutes, 4 ˚C) to obtain plasma sample. At the same post-administration time point, whole brain sample was obtained by rapid dissection, rinsed with PBS, and immediately frozen in individual tissue collection box (dry ice). All brain and plasma samples were stored at -80 ˚C until analysis by LC-MS/MS.

Determination of brain to plasma ratio:

Sample Analysis: Concentrations in plasma and brain homogenates were quantified by liquid chromatography tandem mass spectrometry (LC-MS/MS). Whole brains were homogenized in 3 mL of 70:30 IPA:water in a mini bead beater for 3 min, and centrifuged at 3,500 g for 5 min. 5 uL of the supernatant was diluted in 15 uL of blank plasma for quantification of the analytes. Plasma samples were centrifuged at 3,500 g for 5 min. A standard curve was generated by diluting the analytes DMSO stocks with blank plasma to obtain a final concentration of 10,000 ng/ml followed by a serial dilution down to 0.5 ng/ml. Quality controls were generated by a serial dilution of the 5,000 ng/ml standard curve solution in blank plasma to obtain 3 concentrations of 500, 50, and 5 ng/ml. 20 uL of brain diluted in plasma, plasma, blank plasma, standard curve and QC samples were loaded in a V-bottom 96-well plate. 120 uL of acetonitrile containing 0.05 uM carbamazepine (internal standard) was added to each well and the plate was centrifuged at 3,500 g for 5 min. 60 uL of the supernatant of each well (protein free) was transferred to a new 96-well plate containing 60 uL of water. The plates were sealed for analysis by LC-MS/MS.

Plasma and brain tissue samples originating from in vivo studies were analyzed by electrospray ionization using an AB Sciex Q-TRAP 5500 (Foster City, CA) that was coupled to a Shimadzu LC-20AD pump (Columbia, MD) and a Leap Technologies CTC PAL auto-sampler (Carrboro, NC). Analytes were separated by gradient elution using a C18 column (3 x 50 mm, 3 mm; Fortis

Technologies Ltd, Cheshire, UK) that was thermostated at 40 °C. HPLC mobile phase A was 0.1% formic acid in water (pH unadjusted); mobile phase B was 0.1% formic acid in acetonitrile (pH unadjusted). A 10% B gradient was held for 0.2 min and was linearly increased to 90% B over 0.8 min, with an isocratic hold for 0.5 min, before transitioning to 10% B over 0.05 min. The column was re-equilibrated (1 min) before the next sample injection. The total run time was 2.55 min, and the HPLC flow rate was 0.5 ml/min. The source temperature was set at 500 °C, and mass spectral analyses were performed using a Turbo-Ion spray source in positive ionization mode (5.0-kV spray voltage) and using multiple-reaction monitoring of transitions specific for the analytes. All data were analyzed using AB Sciex Analyst 1.5.1 software.

Brain plasma concentration ratio (K_p) was calculated by dividing brain concentration by plasma concentration for each animal. Unbound brain to unbound plasma concentration ratio ($K_{p,uu}$) is calculated using the following formula: $K_{p,uu}$ = (Brain ng/g x brain f_u)/(plasma ng/ml x plasma f_u).

Rat discrete tissue distribution studies following oral administration in rats:

Adult male Sprague Dawley rats (Envigo, Indianapolis, Indiana) were used to determine time-dependent changes in plasma and brain levels of VU6032423 and VU6035406 following administration by oral gavage (p.o.) according to procedures approved by the Vanderbilt University Institutional Animal Care and Use Committee.[4] Briefly, compounds were formulated as microsuspensions in 10% Tween 80 in water and administered in a volume of 10 mL/kg body weight to achieve doses of 0.1, 1.0, and 10 mg/kg. Animals were then deeply anesthetized with isoflurane for collection of terminal trunk blood and brain. Trunk blood was collected in EDTA-fortified tubes and plasma separated by centrifugation at 4 °C for 10 min at 1700 g. Brain and plasma samples were flash frozen on dry ice and stored at -80 °C until bioanalysis.

Pharmacokinetic profiles in rats following oral single escalating doses:

Single escalating oral dosing in Sprague-Dawley rats was performed at Frontage Laboratories according to their non-GLP Standard Operating Procedure and IACUC protocls. In short, compounds were formulated in 10% Tween 80 in water and dosed at 10 ml/kg. At different times, arterial blood was collected from a femoral artery catheter, and compound concentration was determined in plasma by LC-MS/MS following their non-GLP protocol. PK parameters were determined using Phoenix WinNonlin software (version 6.3).

Pharmacokinetic profiles in dogs following IV and PO single dose:

IV PO PK studies were performed at Frontage Laboratories according to their non-GLP Standard Operating Procedure and IACUC protocls. In short, compounds were formulated as a solution in ethanol, PEG400, and saline (1:7:2 v/v, respectively) and administered as a single IV dose (1 mL/kg) to male Beagle dogs (n = 3). For the PO PK study, compounds were formulated in 0.5% methylcellulose, 0.1 % Tween 80 in saline and administered by gavage as a single PO dose (5 ml/kg) to male Beagle dogs. At different times, arterial blood was collected from a femoral artery catheter, and compound concentration was determined in plasma by LC-MS/MS following their non-GLP protocol. PK parameters were determined using Phoenix WinNonlin software (version 6.3).

In-vitro determination of blood-brain barrier penetration Potential:

Blood-brain barrier penetration was determined using MDR1-MDCK cell monolayers by Absorption Systems, following their protocol. In short, compounds were incubated at 5 μM final concentration on one side of the cell monolayer for 2 hours. Compounds concentration on either side of the monolayer was determined by LC-MS/MS and apparent permeability and efflux ratio were determined as described in Wang, Q. et al. [1].

General Methods

All reactions were carried out employing standard chemical techniques under an inert atmosphere. Solvents used for extraction, washing, and chromatography were HPLC grade. All reagents were purchased from commercial sources and were used without further purification. All microwave reactions were carried out in sealed tubes in a Biotage Initiator microwave synthesis reactor. Temperature control was automated via IR sensor and all indicated temperatures correspond to the maximal temperature reached during each experiment. Analytical HPLC was performed on an Agilent 1200 LCMS with UV detection at 215 nm and 254 nm along with ELSD detection and electrospray ionization, with all final compounds showing > 95% purity and a parent mass ion consistent with the desired structure. Low resolution mass spectra were obtained on an Agilent 6120 or 6150 with ESI source. All NMR spectra were recorded on a 400 MHz Brüker AV-400 instrument. ^{1}H chemical shifts are reported as δ values in ppm relative to the residual solvent peak ($CDCl_3$ = 7.26). Data are reported as follows: chemical shift, multiplicity (br. = broad, s = singlet, d = doublet, t = triplet, q = quartet, dd = doublet of doublets, m = multiplet), coupling constant (Hz), and integration. ^{13}C chemical shifts are reported as δ values in ppm relative to the residual solvent peak ($CDCl_3$ = 77.16). High resolution mass spectra were obtained on an Agilent 6540 UHD Q-TOF with ESI source. Automated flash column chromatography was performed on a Teledyne ISCO Combiflash Rf system. For compounds that were purified on a Gilson preparative reversed-phase HPLC, the system comprised of a 333 aqueous pump with solvent-selection valve, 334 organic pump, GX-271 or GX-281 liquid hander, two column switching valves, and a 155 UV detector. UV wavelength for fraction collection was user-defined, with absorbance at 254 nm always monitored. Method: Phenomenex Axia-packed Luna C18, 30 x 50 mm, 5 µm column. Mobile phase: CH_3CN in H_2O (0.1% TFA). Gradient conditions: 0.75 min equilibration, followed by user defined gradient (starting organic percentage, ending organic percentage, duration), hold at 95% CH_3CN in H_2O (0.1% TFA) for 1 min, 50 mL/min, 23 ºC. Melting points were recorded on an OptiMelt automated melting point system by Stanford Research Systems. cLogP, MW, and TPSA were calculated using PerkinElmer ChemDraw professional version 20.1.0.110.

SFC Purification

Analytical Separation:

Chiral SFC separation was performed on a Thar (Waters) Investigator. Column: Chiral Technologies CHIRALPAK IF, 4.6 x 250 mm, 5 um. Gradient conditions: 20% to 50% IPA in CO_2 over 7 min, hold at 50% CO_2 for 1 min. Flow rate: 3.5 mL/min. Column temperature: 40° C. System backpressure: 100 bar.

Preparative Separation:

Chiral SFC separation was performed on a PIC Solution SFC-PICLab PREP 100. Column: Chiral Technologies CHIRALPAK IF, 20 x 250 mm, 5 um. Conditions: 30% IPA in CO_2. Flow rate: 80 mL/min. Column temperature: 40° C. System backpressure: 100 bar.

Synthetic Procedure for Selected Compounds

Synthesis of 9

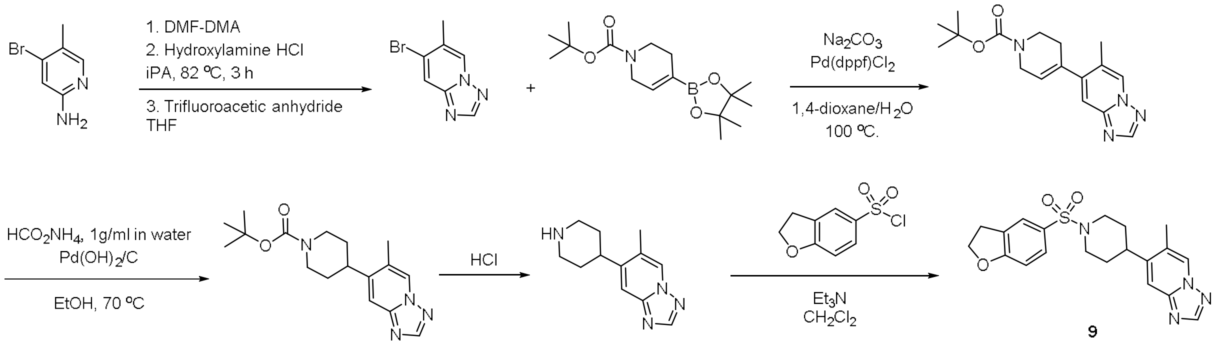

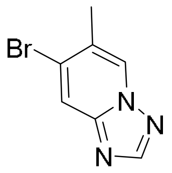

7-bromo-6-methyl-[1,2,4]triazolo[1,5-*a*]pyridine

Step 1. 4-bromo-5-methylpyridin-2-amine (320.0 mg, 1.71 mmol, 1 eq) was added to a round bottom flask. iPA (10 mL) and DMF•DMA (0.30 mL, 2.22 mmol, 1.3 eq) were then added. The resulting mixture was heated to 82 ºC for 3 h, after which time the reaction was cooled to 50 ºC. NH_2OH•HCl (154.6 mg, 2.22 mmol, 1.3 eq) was added in one portion, and the reaction was stirred at 50 ºC for 2 h, after which time the reaction was cooled to room temperature and concentrated under reduced pressure. This hydroxy-formamidine crude mixture was directly used without further purification.

Step 2. THF (10 mL) was added to the crude mixture of hydroxy-formamidine. The resulting mixture was cooled to 0 ºC. Trifluoroacetic anhydride (0.71 mL, 5.12 mmol, 3 eq) was then added by syringe, and the reaction was stirred at room temperature overnight, after which time the reaction was quenched with 1 N NaOH, and then extracted with $CHCl_3$/iPA solution (3:1). The combined organic extracts were concentrated and dried over Na_2SO_4, and solvents were filtered and concentrated. The crude residue was then purified by silica gel column chromatography (0-70% EtOAc in hexanes) to give the product (277.5 mg, 76%). ^1H NMR (400 MHz, $CDCl_3$) δ 8.46 – 8.41 (m, 1H), 8.28 (s, 1H), 8.02 (s, 1H), 2.44 (s, 3H). ^{13}C NMR (101 MHz, $CDCl_3$) δ 154.1, 149.8, 128.6, 126.7, 124.7, 119.2, 20.0. ES-MS $[M+H]^+$ = 212.1/214.1

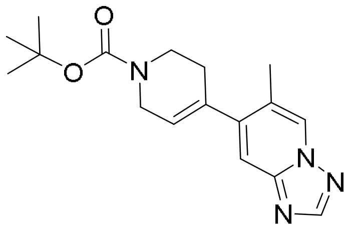

***tert*-butyl 4-(6-methyl-[1,2,4]triazolo[1,5-*a*]pyridin-7-yl)-3,6-dihydropyridine-1(2*H*)-carboxylate**

7-bromo-6-methyl-[1,2,4]triazolo[1,5-*a*]pyridine (215.0 mg, 1.0 mmol, 1 eq), *tert*-butyl 4-(4,4,5,5-tetramethyl-1,3,2-dioxaborolan-2-yl)-3,6-dihydropyridine-1(2*H*)-carboxylate (407.6 mg, 1.3 mmol, 1.3 eq.), Pd(dppf)Cl_2 (74.4 mg, 0.1 mmol, 0.1 eq), and Na_2CO_3 (328.5 mg, 3.0 mmol, 3 eq) were charged into a microwave vial which was sealed and placed under an inert atmosphere. 1,4-Dioxane (10 mL) and H_2O (5 mL) were added via syringe and the reaction mixture was purged with N_2. The reaction was then heated to 90 ºC. Upon completion by LCMS, the reaction mixture was filtered through a pad of Celite which was rinsed thoroughly with EtOAc/CH_2Cl_2. The filtrate was concentrated under reduced pressure and purified using column chromatography (0-100% EtOAc in hexanes) to provide the title compound (296.2 mg, 93%). ^{1}H NMR (400 MHz, $CDCl_3$) δ 8.40 (s, 1H), 8.32 (s, 1H), 7.56 (s, 1H), 5.74 (s, 1H), 4.08 (q, J = 2.9 Hz, 2H), 3.65 (t, J = 5.6 Hz, 2H), 2.41 – 2.35 (m, 2H), 2.34 (d, J = 1.1 Hz, 3H), 1.51 (s, 9H). ES-MS $[M+H]^+$ = 315.2.

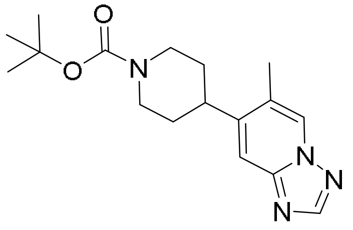

***tert*-butyl 4-(6-methyl-[1,2,4]triazolo[1,5-*a*]pyridin-7-yl)piperidine-1-carboxylate**

tert-butyl 4-(6-methyl-[1,2,4]riazolo[1,5-*a*]pyridin-7-yl)-3,6-dihydropyridine-1(2*H*)-carboxylate (295.0 mg, 0.94 mmol, 1.0 eq) $Pd(OH)_2/C$ (66.0 mg, 0.094 mmol, 0.1 eq, 20%w/w), and aqueous ammonium formate solution (1g/mL) (1.2 mL, 18.8 mmol, 20 eq) in H_2O were added to a vial followed by EtOH (4 mL). The mixture was placed under an H_2 atmosphere. The mixture was heated at 70 °C. Upon completion, the reaction was allowed to cool to room temperature, and the resulting mixture was filtered through Celite and thoroughly washed with MeOH. The filtrate was concentrated and then taken up in CH_2Cl_2 and H_2O

(1:1), and the aqueous layer was extracted with CH_2Cl_2. The combined organic layers were dried with Na_2SO_4, filtered, and concentrated under reduced pressure. The crude material was purified by column chromatography (0-100% EtOAc in hexanes) to give the product (183.1 mg, 62%). ^{1}H NMR (400 MHz, $CDCl_3$) δ 8.36 (s, 1H), 8.24 (s, 1H), 7.53 (s, 1H), 4.29 (s, 2H), 2.95 – 2.74 (m, 3H), 2.39 (d, J = 1.1 Hz, 3H), 1.82 (dt, J = 13.2, 2.6 Hz, 2H), 1.68 – 1.57 (m, 2H), 1.48 (s, 9H). ^{13}C NMR (101 MHz, $CDCl_3$) δ 154.8, 153.4, 149.7, 148.9, 126.7, 123.3, 112.3, 79.9, 44.3, 38.6 (2), 32.3 (2), 28.6 (3), 16.6. ES-MS $[M+H]^+$ = 317.3.

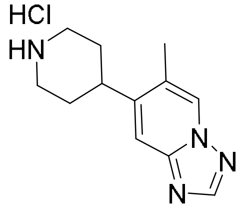

6-methyl-7-(piperidin-4-yl)-[1,2,4]triazolo[1,5-*a*]pyridine

To a solution of *tert*-butyl 4-(6-methyl-[1,2,4]triazolo[1,5-*a*]pyridin-7-yl)piperidine-1-carboxylate (108.0 mg, 0.34 mmol, 1.0 eq) in DCM (2 mL) was added 4 M HCl in dioxane (0.85 mL, 3.4 mmol, 10 eq) at rt. After 16 h, the reaction was concentrated to dryness to give the title compound (86 mg, 99%) as HCl salt. The crude was used in the next step directly without further purification. ES-MS $[M+H]^+$ = 217.3.

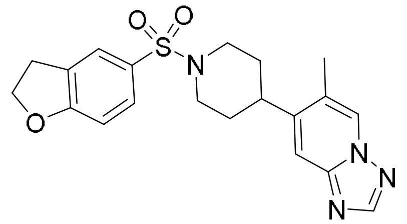

7-(1-((2,3-dihydrobenzofuran-5-yl)sulfonyl)piperidin-4-yl)-6-methyl-[1,2,4]triazolo[1,5-*a*]pyridine (9)

2,3-dihydrobenzofuran-5-sulfonyl chloride (30.3 mg, 0.14 mmol, 1 eq) and 6-methyl-7-(piperidin-4-yl)-[1,2,4]triazolo[1,5-*a*]pyridine; hydrochloride (35.0 mg, 0.14 mmol, 1 eq) were added to a vial. DCM (1 ml) and *N*,*N*-diisopropylethylamine (72.4 µL, 0.42 mmol, 3 eq) were added, and the resulting mixture was stirred at rt for 30 min. H_2O (1 mL) was added to quench the reaction. The reaction mixture was extracted with DCM (2 x). The combined organic layer was passed through a phase separator and concentrated to give the crude. The crude was then purified by reverse phase HPLC (5%-95% MeCN in 0.1% TFA aqueous

solution over 6 min) and fractions containing product were basified with sat. aq. $NaHCO_3$, and extracted with DCM (2 mL x 2). The organic extracts were filtered through a phase separator and concentrated to give the title compound (23.8 mg, 43%). ^{1}H NMR (400 MHz, $CDCl_3$) δ 8.34 (s, 1H), 8.25 (s, 1H), 7.65 – 7.55 (m, 2H), 7.53 (s, 1H), 6.89 (d, *J* = 8.3 Hz, 1H), 4.70 (t, *J* = 8.8 Hz, 2H), 4.01 – 3.93 (m, 2H), 3.30 (t, *J* = 8.8 Hz, 2H), 2.63 (tt, *J* = 11.3, 4.0 Hz, 1H), 2.41 (td, *J* = 11.7, 3.2 Hz, 2H), 2.31 (d, *J* = 1.0 Hz, 3H), 1.94 – 1.78 (m, 4H). ^{13}C NMR (101 MHz, $CDCl_3$) δ 164.1, 153.9, 150.0, 147.6, 129.4, 128.5, 127.6, 126.8, 125.0, 122.8, 112.5, 109.7, 72.4, 46.8, 37.7, 31.7, 29.2, 16.5. ES-MS $[M+H]^+$ = 399.2.

Syntheis of 10

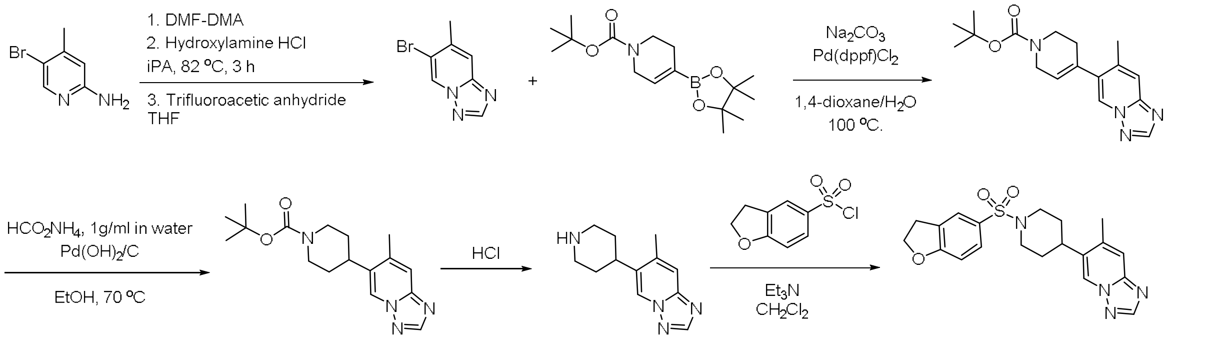

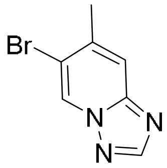

6-bromo-7-methyl-[1,2,4]triazolo[1,5-*a*]pyridine

Step 1. 5-bromo-4-methylpyridin-2-amine (15.0 g, 80.2 mmol, 1 eq) was added to a round bottom flask. iPA (270 mL) and DMF•DMA (13.9 mL, 104.3 mmol, 1.3 eq) were then added. The resulting mixture was heated to 82 °C for 3 h, after which time the reaction was cooled to 50 °C. NH_2OH•HCl (7.25 g, 104.3 mmol, 1.3 eq) was added in one portion, and the reaction was stirred at 50 °C for 2 h, after which time the reaction was cooled to room temperature and concentrated under reduced pressure. This hydroxy-formamidine crude mixture was directly used without further purification.

Step 2. THF (300 mL) was added to the crude mixture of hydroxy-formamidine. The resulting mixture was cooled to 0 °C. Trifluoroacetic anhydride (33.4 mL, 240.6 mmol, 3 eq) was then added slowly, and the reaction was stirred at room temperature overnight, after which time the reaction was quenched with 1 N NaOH, and then extracted with $CHCl_3$/iPA solution (3:1). The combined organic extracts were concentrated

and dried over Na_2SO_4, and solvents were filtered and concentrated. The crude residue was then purified by silica gel column chromatography (0-100% EtOAc in hexanes) to give the title compound (12.2 g, 72%). ^{1}H NMR (400 MHz, $CDCl_3$) δ 8.76 (s, 1H), 8.27 (s, 1H), 7.62 (s, 1H), 2.52 (d, J = 1.0 Hz, 3H). ^{13}C NMR (101 MHz, $CDCl_3$) δ 154.5, 150.0, 141.1, 128.7, 115.9, 112.3, 23.1. ES-MS $[M+H]^+$ = 212.2 and 214.3.

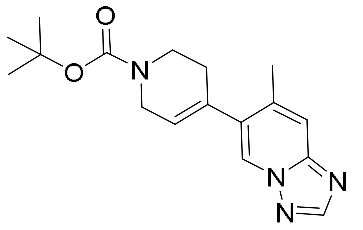

***tert*-butyl 4-(7-methyl-[1,2,4]triazolo[1,5-*a*]pyridin-6-yl)-3,6-dihydropyridine-1(2*H*)-carboxylate**

6-bromo-7-methyl-[1,2,4]triazolo[1,5-*a*]pyridine (500.0 mg, 2.36 mmol, 1 eq), *tert*-butyl 4-(4,4,5,5-tetramethyl-1,3,2-dioxaborolan-2-yl)-3,6-dihydropyridine-1(2*H*)-carboxylate (947.8 mg, 1.3 mmol, 1.3 eq), Pd(dppf)Cl_2 (173.0 mg, 0.24 mmol, 0.1 eq), and Na_2CO_3 (764.0 mg, 3.0 mmol, 3 eq) were charged into a microwave vial which was sealed and placed under an inert atmosphere. 1,4-Dioxane (10 mL) and H_2O (5 mL) were added via syringe and the reaction mixture was purged with N_2 and subjected to microwave radiation at 140 °C. After 30 min, the reaction mixture was filtered through a pad of Celite which was rinsed thoroughly with EtOAc/CH_2Cl_2. The filtrate was concentrated under reduced pressure and purified using column chromatography (0-100% EtOAc in hexanes) to provide the title compound (603.5 mg, 81%). ^{1}H NMR (400 MHz, $CDCl_3$) δ 8.31 (s, 1H), 8.26 (s, 1H), 7.52 (s, 1H), 5.74 (s, 1H), 4.08 (d, J = 2.9 Hz, 2H), 3.65 (t, J = 5.6 Hz, 2H), 2.39 (d, J = 1.1 Hz, 3H), 2.38 – 2.32 (s, 2H), 1.51 (s, 9H). ES-MS $[M+H]^+$ = 315.2.

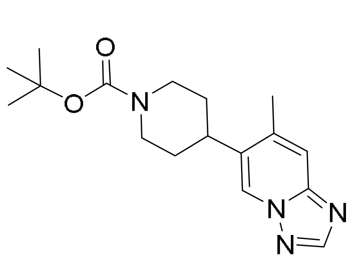

***tert*-butyl 4-(7-methyl-[1,2,4]triazolo[1,5-*a*]pyridin-6-yl)piperidine-1-carboxylate**

tert-butyl 4-(7-methyl-[1,2,4]triazolo[1,5-*a*]pyridin-6-yl)-3,6-dihydropyridine-1(2*H*)-carboxylate (1.0 g, 3.18 mmol, 1.0 eq), Pd$(OH)_2$/C (223.3 mg, 18.3 mmol, 0.1 eq, 20%w/w), and aqueous ammonium formate

solution (1g/mL) (3.66 mL, 58.1 mmol, 18.3 eq) in H_2O were added to a vial followed by EtOH (8 mL). The mixture was placed under an H_2 atmosphere. The mixture was heated at 70 °C. Upon completion, the reaction was allowed to cool to room temperature, and the resulting mixture was filtered through Celite and thoroughly washed with MeOH. The filtrate was concentrated and then taken up in CH_2Cl_2 and H_2O (1:1), and the aqueous layer was extracted with CH_2Cl_2. The combined organic layers were dried with Na_2SO_4, filtered, and concentrated under reduced pressure. The crude material was purified by column chromatography (0-100% EtOAc in hexanes) to give the product (846.0 mg, 84%). ^{1}H NMR (400 MHz, $CDCl_3$) δ 8.34 (s, 1H), 8.23 (s, 1H), 7.52 (s, 1H), 4.29 (s, 2H), 2.92 - 2.75 (m, 3H), 2.47 (s, 3H), 1.88 (d, *J* = 13.1, 2H), 1.58 (qd, *J* = 12.6, 4.2 Hz, 2H), 1.48 (s, 9H). ^{13}C NMR (101 MHz, $CDCl_3$) δ 154.8, 153.9, 149.4, 140.2, 131.9, 124.8, 116.2, 79.9, 44.5, 36.9, 32.7, 28.6, 19.8. ES-MS $[M+H]^+$ = 317.4.

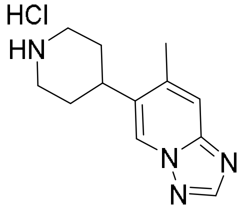

7-methyl-6-(piperidin-4-yl)-[1,2,4]triazolo[1,5-*a*]pyridine

To a solution of *tert*-butyl 4-(7-methyl-[1,2,4]triazolo[1,5-*a*]pyridin-6-yl)piperidine-1-carboxylate (28.5 mg, 0.09 mmol, 1.0 eq) in a mixture of 1,4-dioxane (0.5 mL) and MeOH (0.1 mL) was added 4 M HCl in dioxane (0.34 mL, 1.35 mmol, 15 eq) at rt. Upon completion by LCMS analysis, the reaction was concentrated to dryness to give the title compound (22.7 mg, 99%) as HCl salt. The crude was used in the next step directly without further purification. ES-MS $[M+H]^+$ = 217.2.

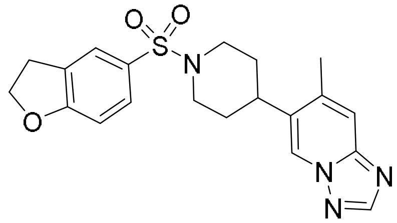

6-(1-((2,3-dihydrobenzofuran-5-yl)ulfonyl)piperidin-4-yl)-7-methyl-[1,2,4]triazolo[1,5-*a*]pyridine (10)

2,3-dihydrobenzofuran-5-sulfonyl chloride(9.3 mg, 0.04 mmol, 1 eq) and 7-methyl-6-(piperidin-4-yl)-[1,2,4]triazolo[1,5-*a*]pyridine; hydrochloride (10.7 mg, 0.04 mmol, 1 eq) were added to a vial. DCM (0.5

ml) and *N,N*-diisopropylethylamine (22 μL, 0.127 mmol, 3 eq) were added, and the resulting mixture was stirred at rt for 30 min, after which time H_2O (1 mL) was added to quench the reaction. The reaction mixture was extracted with DCM (2 x). The combined organic layer was passed through a phase separator and concentrated to give the crude. The crude was then purified by reverse phase HPLC (5%-95% MeCN in 0.1% TFA aqueous solution over 6 min) and fractions containing product were basified with sat. aq. $NaHCO_3$, and extracted with DCM (2 mL x 2). The organic extracts were filtered through a phase separator and concentrated to give the title compound (10.1 mg, 60%). ^{1}H NMR (400 MHz, $CDCl_3$) δ 8.39 (s, 1H), 8.34 (s, 1H), 7.68 (s, 1H), 7.62 (s, 1H), 7.59 (dd, *J* = 8.4, 2.1 Hz, 1H), 6.90 (d, *J* = 8.3 Hz, 1H), 4.71 (t, *J* = 8.9 Hz, 2H), 3.98 (d, *J* = 11.7 Hz, 2H), 3.30 (t, *J* = 8.8 Hz, 2H), 2.66 (tt, *J* = 12.1, 3.3 Hz, 1H), 2.45 – 2.37 (m, 2H), 2.43 (s, 3H) 1.97 (d, *J* = 13.8 Hz,, 2H), 1.83 (qd, *J* = 13.3, 12.7, 4.0 Hz, 2H). ES-MS $[M+H]^+$ = 399.4.

Synthesis of 33

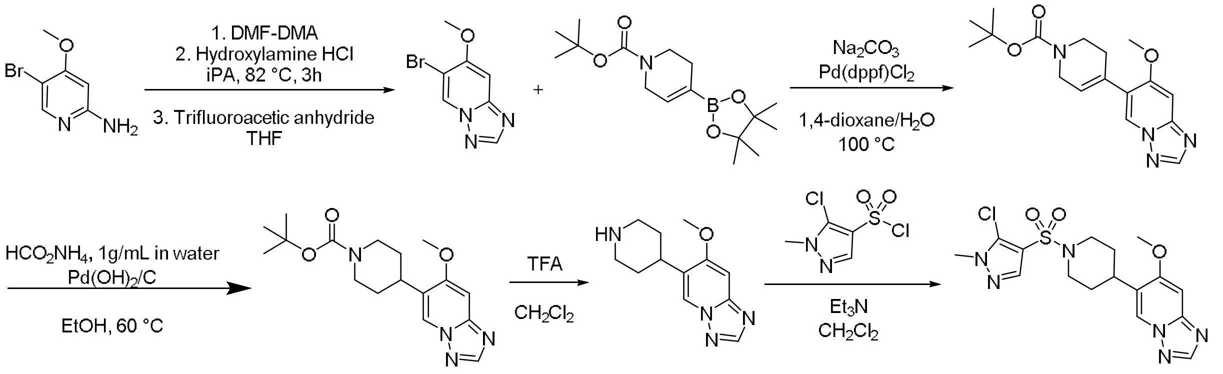

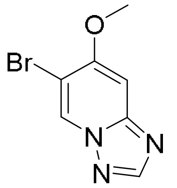

6-bromo-7-methoxy-[1,2,4]triazolo[1,5-*a*]pyridine

Step 1. 5-Bromo-4-methoxy-pyridin-2-amine (1.22 g, 6.0 mmol, 1 eq) was added to a round bottom flask. iPA (15 mL) and DMF•DMA (1.20 mL, 9.0 mmol, 1.5 eq) were then added. The resulting mixture was heated to 82 ºC for 3 h, after which time the reaction was cooled to 50 ºC. NH_2OH•HCl (625.0 mg, 9.0 mmol, 1.5 eq) was added in one portion, and the reaction was stirred at 50 ºC for 2 h, after which time the

reaction was cooled to room temperature and concentrated under reduced pressure. This hydroxy-formamidine crude mixture was directly used without further purification.

Step 2. THF (30 mL) was added to the crude mixture of hydroxy-formamidine. The resulting mixture was cooled to 0 °C. Trifluoroacetic anhydride (2.5 mL, 18.0 mmol, 3 eq) was then added by syringe, and the reaction was stirred at room temperature overnight, after which time the reaction was quenched with 1 N NaOH, and then extracted with $CHCl_3$/iPA solution (3:1). The combined organic extracts were concentrated and dried over Na_2SO_4, and solvents were filtered and concentrated. The crude residue was then purified by silica gel column chromatography (0-100% EtOAc in hexanes) to give the product (278.0 mg, 20%). ^{1}H NMR (400 MHz, DMSO) δ 9.34 (s, 1H), 8.37 (s, 1H), 7.39 (s, 1H), 3.99 (s, 3H); ES-MS $[M+H]^+$ = 228.2 and 230.2.

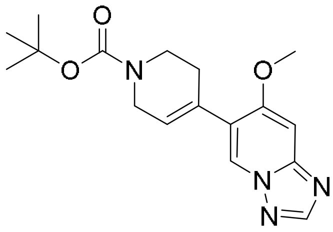

tert-butyl 4-(7-methoxy-[1,2,4]triazolo[1,5-_a_]pyridin-6-yl)-3,6-dihydropyridine-1(2_H_)-carboxylate

6-Bromo-7-methoxy-[1,2,4]triazolo[1,5-*a*]pyridine (228.0 mg, 1.0 mmol, 1 eq.), *tert*-butyl 4-(4,4,5,5-tetramethyl-1,3,2-dioxaborolan-2-yl)-3,6-dihydropyridine-1(2*H*)-carboxylate (371.0 mg, 1.2 mmol, 1.2 eq.), Pd(dppf)Cl_2 (123.0 mg, 0.15 mmol, 0.15 eq), and Na_2CO_3 (324.0 mg, 3.0 mmol, 3.0 eq) were charged into a microwave vial which was sealed and placed under an inert atmosphere. 1,4-Dioxane (8 mL) and H_2O (4 mL) were added via syringe and the reaction mixture was purged with N_2. The reaction was then heated to 100 °C. Upon completion by LCMS analysis, the reaction mixture was filtered through a pad of Celite which was rinsed thoroughly with EtOAc/CH_2Cl_2. The filtrate was concentrated under reduced pressure and purified using column chromatography (0-100% MeOH/DCM with NH_4OH additive) to provide the title compound (250 mg, 76%). ^{1}H NMR (400 MHz, $CDCl_3$) δ 8.29 (s, 1H), 8.23 (s, 1H), 7.04 (s, 1H), 5.86 (m, 1H), 4.07 (q, J = 2.9 Hz, 2H), 3.94 (s, 3H), 3.62 (t, J = 5.6 Hz, 2H), 2.47 (m, 2H), 1.50 (s, 9H); ES-MS $[M+H]^+$ = 331.2.

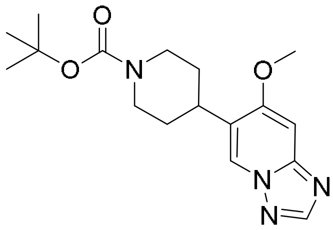

***tert*-Butyl 4-(7-methoxy-[1,2,4]triazolo[1,5-*a*]pyridin-6-yl)piperidine-1-carboxylate**

tert-Butyl 4-(7-methoxy-[1,2,4]triazolo[1,5-*a*]pyridin-6-yl)-3,6-dihydropyridine-1(2*H*)-carboxylate (250.0 mg, 0.76 mmol, 1.0 eq.) $Pd(OH)_2/C$ (119.0 mg, 0.17 mmol, 0.2 eq, 20%w/w), and aqueous ammonium formate solution (1g/mL) (0.946 mL, 15.1 mmol, 20 eq) in H_2O were added to a vial followed by EtOH (7.5 mL). The mixture was placed under an H_2 atmosphere. The mixture was heated at 60 °C. Upon completion, the reaction was allowed to cool to room temperature, and the resulting mixture was filtered through Celite and thoroughly washed with MeOH. The filtrate was concentrated and then taken up in CH_2Cl_2 and H_2O (1:1), and the aqueous layer was extracted with CH_2Cl_2. The combined organic layers were dried with Na_2SO_4, filtered, and concentrated under reduced pressure. The crude material was purified by column chromatography (0-100% MeOH/DCM with NH_4OH additive) to give the title compound (186 mg, 74%). ^{1}H NMR (400 MHz, $CDCl_3$) δ 8.22 (s, 1H), 8.17 (s, 1H), 6.98 (s, 1H), 4.27 (s, 2H), 3.95 (s, 3H), 3.02 (tt, J = 12.2, 3.3 Hz, 1H), 2.84 (t, J = 12.3 Hz, 2H), 1.92 (d, J = 12.7 Hz, 2H), 1.61 – 1.50 (m, 2H), 1.48 (s, 9H); ES-MS $[M+H]^+$ = 333.2.

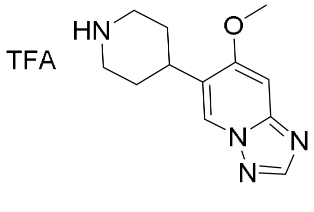

7-Methoxy-6-(piperidin-4-yl)-[1,2,4]triazolo[1,5-*a*]pyridine

To a solution of *tert*-butyl 4-(7-methoxy-[1,2,4]triazolo[1,5-*a*]pyridin-6-yl)piperidine-1-carboxylate (186.0 mg, 0.56 mmol, 1.0 eq) in DCM (5.6 mL) was added trifluoroacetic acid (0.43 mL, 5.6 mmol, 10 eq) at rt. After 16 h, the reaction was concentrated to dryness to give the title compound (129 mg, 99%) as TFA salt. The crude was used in the next step directly without further purification. ^{1}H NMR (400 MHz, $CDCl_3$) δ 8.24 (s, 1H), 8.16 (s, 1H), 6.96 (s, 1H), 3.94 (s, 3H), 3.21 (dt, J = 12.5, 2.9 Hz, 2H), 2.99 (tt, J = 12.1, 3.3 Hz, 1H), 2.79 (td, J = 12.2, 2.4 Hz, 2H), 1.92 (dp, J = 12.3, 2.4 Hz, 2H), 1.68 (s, 2H), 1.62 – 1.48 (m, 2H);

^{13}C NMR (101 MHz, $CDCl_3$) δ 159.9, 154.0, 150.9, 126.4, 125.4, 93.7, 56.1, 47.3 (2), 34.7, 33.2 (2); ES-MS $[M+H]^+$ = 233.2.

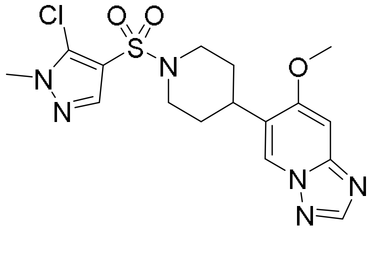

6-(1-((5-chloro-1-methyl-1*H*-pyrazol-4-yl)sulfonyl)piperidin-4-yl)-7-methoxy-[1,2,4]triazolo[1,5-*a*]pyridine (33)

5-Chloro-1-methyl-1*H*-pyrazole-4-sulfonyl chloride (32.2 mg, 0.14 mmol, 1 eq) and 7-methoxy-6-(piperidin-4-yl)-[1,2,4]triazolo[1,5-*a*]pyridine 2,2,2-trifluoroacetate (29.8 mg, 0.14 mmol, 1 eq) were added to a vial. DCM (1 ml) and *N*,*N*-diisopropylethylamine (72.4 µL, 0.42 mmol, 3 eq) were added, and the resulting mixture was stirred at rt for 18 h. H_2O (1 mL) was added to quench the reaction. The reaction mixture was extracted with DCM (2 x). The combined organic layer was passed through a phase separator and concentrated to give the crude. The crude was then purified by reverse phase HPLC (5-95% MeCN in 0.1% TFA aqueous solution over 6 min) and fractions containing product were basified with sat. aq. $NaHCO_3$, and extracted with DCM (2 mL x 2). The organic extracts were filtered through a phase separator and concentrated to give the title compound (8 mg, 14%); ^1H NMR (400 MHz, $CDCl_3$) δ 8.23 (s, 1H), 8.18 (s, 1H), 7.80 (s, 1H), 6.98 (s, 1H), 4.00 (dp, *J* = 11.7, 1.9 Hz, 2H), 3.93 (s, 3H), 3.92 (s, 3H), 2.87 (tt, *J* = 12.3, 3.3 Hz, 1H), 2.58 (td, *J* = 12.1, 2.4 Hz, 2H), 2.03 (dt, *J* = 12.6, 2.5 Hz, 2H), 1.87 – 1.73 (m, 2H); ^{13}C NMR (101 MHz, $CDCl_3$) δ 159.5, 154.2, 151.0, 140.4, 129.0, 125.3, 124.5, 115.3, 94.1, 56.2, 46.7 (2), 37.3, 33.9, 31.0 (2); ES-MS $[M+H]^+$ = 411.3.

Synthesis of 36

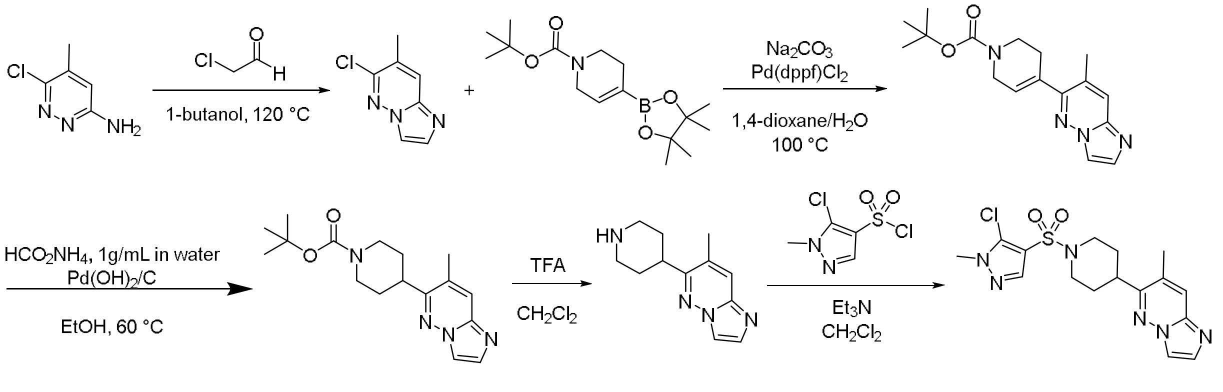

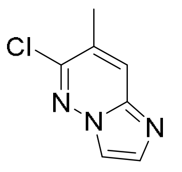

6-chloro-7-methylimidazo[1,2-*b*]pyridazine

To a solution of 6-chloro-5-methylpyridazin-3-amine (500 mg, 3.48 mmol) in 1-butanol (5 ml) was added an aqueous solution of 2-chloroacetaldehyde solution (487 μl, 3.83 mmol). The mixture was refluxed overnight. The mixture was concentrated onto Celite and purified by silica gel column chromatography (0 - 10% MeOH/DCM) to afford the title compound (135 mg, 23%). ^{1}H NMR (400 MHz, $CDCl_3$) δ 7.87 (s, 1H), 7.77 (s, 1H), 7.71 (d, J = 1.3 Hz, 1H), 2.45 (d, J = 1.1 Hz, 3H); ^{13}C NMR (101 MHz, $CDCl_3$) δ 148.7, 138.8, 134.2, 127.7, 125.6, 116.3, 19.9; ES-MS $[M+H]^+$ = 168.0 and 170.0.

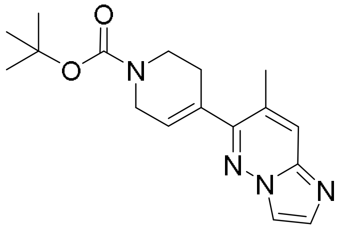

***tert*-butyl 4-(7-methylimidazo[1,2-*b*]pyridazin-6-yl)-3,6-dihydropyridine-1(2*H*)-carboxylate**

6-Chloro-7-methylimidazo[1,2-*b*]pyridazine (135.0 mg, 0.80 mmol, 1 eq), *tert*-butyl 4-(4,4,5,5-tetramethyl-1,3,2-dioxaborolan-2-yl)-3,6-dihydropyridine-1(2*H*)-carboxylate (299.0 mg, 0.97 mmol, 1.2 eq), Pd(dppf)Cl_2 (59.0 mg, 0.08 mmol, 0.1 eq), and Na_2CO_3 (260.0 mg, 2.4 mmol, 3.0 eq) were charged

into a microwave vial which was sealed and placed under an inert atmosphere. 1,4-Dioxane (3.2 mL) and H_2O (0.8 mL) were added via syringe and the reaction mixture was purged with N_2. The reaction was then heated to 90 ºC. Upon completion by LCMS analysis, the reaction mixture was filtered through a pad of Celite which was rinsed thoroughly with EtOAc/CH_2Cl_2. The filtrate was concentrated under reduced pressure and purified using column chromatography (0-100% MeOH/DCM with NH_4OH additive) to provide the title compound (137 mg, 54%). ^{1}H NMR (400 MHz, $CDCl_3$) δ 7.86 (s, 1H), 7.71 (s, 1H), 7.68 (d, J = 1.2 Hz, 1H), 5.93 (s, 1H), 4.17 – 4.09 (m, 2H), 3.68 (t, J = 5.6 Hz, 2H), 2.55 – 2.47 (m, 2H), 2.37 (d, J = 1.1 Hz, 3H), 1.50 (s, 9H); ES-MS $[M+H]^+$ = 315.2.

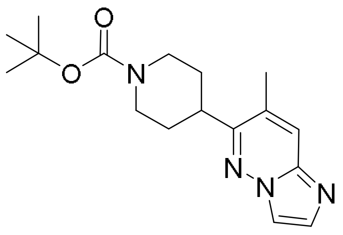

***tert*-butyl 4-(7-methylimidazo[1,2-*b*]pyridazin-6-yl)piperidine-1-carboxylate**

tert-Butyl 4-(7-methyl-[1,2-*b*]pyridazin-6-yl)piperidine-1-carboxylate (117.0 mg, 0.37 mmol, 1.0 eq) $Pd(OH)_2$/C (57.0 mg, 0.084 mmol, 0.22 eq, 20%w/w), and aqueous ammonium formate solution (1g/mL) (0.465 mL, 7.44 mmol, 20 eq) in H_2O were added to a vial followed by EtOH (3.5 mL). The mixture was placed under an H_2 atmosphere. The mixture was heated at 60 °C. Upon completion, the reaction was allowed to cool to room temperature, and the resulting mixture was filtered through Celite and thoroughly washed with MeOH. The filtrate was concentrated and then taken up in CH_2Cl_2 and H_2O (1:1), and the aqueous layer was extracted with CH_2Cl_2. The combined organic layers were dried with Na_2SO_4, filtered, and concentrated under reduced pressure. The crude material was purified by column chromatography (0-100% MeOH/DCM with NH_4OH additive) to give the title compound (88 mg, 75%). ^{1}H NMR (400 MHz, $CDCl_3$) δ 7.82 (s, 1H), 7.67 – 7.61 (m, 2H), 4.45 – 4.13 (m, 2H), 2.98 (p, J = 7.6 Hz, 1H), 2.93 – 2.75 (m, 2H), 2.41 (d, J = 1.1 Hz, 3H), 1.91 – 1.81 (m, 4H), 1.49 (s, 9H); ^{13}C NMR (101 MHz, $CDCl_3$) δ 157.2, 154.8, 138.9, 133.2, 126.8, 124.8, 116.0, 79.7, 44.2, 39.2 (2), 30.8 (2), 28.6 (3), 18.9; ES-MS $[M+H]^+$ = 317.2.

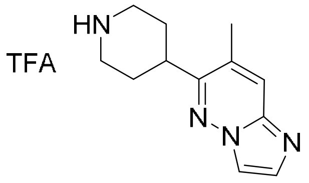

7-Methyl-6-(piperidin-4-yl)imidazo[1,2-*b*]pyridazine

To a solution of *tert*-butyl 4-(7-methylimidazo[1,2-*b*]pyridazin-6-yl)piperidine-1-carboxylate (88.0 mg, 0.28 mmol, 1.0 eq) in DCM (2.8 mL) was added trifluoroacetic acid (0.21 mL, 2.8 mmol, 10 eq) at rt. After 16 h, the reaction was concentrated to dryness to give the title compound (60 mg, 100%) as TFA salt. The crude was used in the next step directly without further purification. ^{1}H NMR (400 MHz, $CDCl_3$) δ 8.19 (s, 1H), 7.92 (dd, *J* = 1.7, 0.7 Hz, 1H), 7.77 (d, *J* = 1.6 Hz, 1H), 3.64 (dt, *J* = 13.1, 3.5 Hz, 2H), 3.23 (ddt, *J* = 10.5, 7.1, 3.6 Hz, 1H), 3.15 (t, *J* = 12.5 Hz, 2H), 2.50 (d, *J* = 1.1 Hz, 3H), 2.40 – 2.25 (m, 2H), 2.16 (d, *J* = 14.5 Hz, 2H), NH protons overlapped with the water peak.; ES-MS $[M+H]^+$ = 217.1.

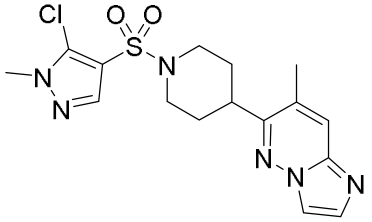

6-(1-((5-Chloro-1-methyl-1*H*-pyrazol-4-yl)sulfonyl)piperidin-4-yl)-7-methylimidazo[1,2-*b*]pyridazine (36)

5-Chloro-1-methyl-1*H*-pyrazole-4-sulfonyl chloride (29.8 mg, 0.14 mmol, 1 eq) and 7-methyl-6-(piperidin-4-yl)imidazo[1,2-*b*]pyridazine 2,2,2-trifluoroacetate (29.8 mg, 0.14 mmol, 1 eq) were added to a vial. DCM (1 ml) and *N*,*N*-diisopropylethylamine (72.4 µL, 0.42 mmol, 3 eq) were added, and the resulting mixture was stirred at rt for 18 h. H_2O (1 mL) was added to quench the reaction. The reaction mixture was extracted with DCM (2 x). The combined organic layer was passed through a phase separator and concentrated to give the crude. The crude was then purified by reverse phase HPLC (5-95% MeCN in 0.1% TFA aqueous solution over 6 min) and fractions containing product were basified with sat. aq. $NaHCO_3$, and extracted with DCM (2 mL x 2). The organic extracts were filtered through a phase separator and concentrated to give the title compound (7 mg, 13%). ^{1}H NMR (400 MHz, $CDCl_3$) δ 7.83 (t, *J* = 1.0 Hz, 1H), 7.81 (s, 1H), 7.65 (d, *J* = 1.3 Hz, 1H), 7.64 (t, *J* = 1.0 Hz, 1H), 4.04 – 3.94 (m, 2H), 3.93 (s, 3H), 2.84 (tt, *J* = 11.4, 3.6

Hz, 1H), 2.60 (td, J = 12.0, 2.7 Hz, 2H), 2.36 (d, J = 1.1 Hz, 3H), 2.17 – 2.02 (m, 2H), 2.02 – 1.91 (m, 2H); ^{13}C NMR (101 MHz, $CDCl_3$) δ 156.3, 140.4, 138.8, 133.4, 129.0, 126.4, 125.0, 116.1, 115.4, 46.4 (2), 38.3, 37.3, 30.2 (2), 18.8; ES-MS $[M+H]^+$ = 395.3.

Intermediate Example 46a. 5-Bromo-4-cyclopropylpyridin-2-amine

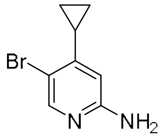

To a solution of 4-cyclopropylpyridin-2-amine (500.0 mg, 3.73 mmol, 1 eq) in CH_3CN (37 mL) at 0 °C, NBS (630.1 mg, 3.54 mmol, 0.95 eq) was added in one portion, and the reaction stirred for 4 h at rt. Upon completion, the reaction was concentrated in vacuo and purified by column chromatography (0-20% MeOH in CH_2Cl_2) to give the title compound (793.9 mg, 99%). ^{1}H NMR (400 MHz, $CDCl_3$) δ 8.08 (s, 1H), 5.99 (s, 1H), 4.37 (br s, 2H), 2.10 (tt, J = 8.4, 5.2 Hz, 1H), 1.09 – 1.03 (m, 2H), 0.72 – 0.67 (m, 2H). ES-MS $[M+H]^+$ = 213 and 215.

Intermediate Example 48c. 6-Bromo-5-methyl-[1,2,4]triazolo[1,5-*a*]pyrimidine

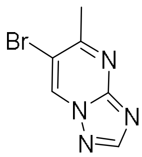

6-Bromo-5-methyl-[1,2,4]triazolo[1,5-*a*]pyrimidine.
The title compound was prepared from 5-bromo-4-methyl-pyrimidin-2-amine similar to General Procedure: Triazolopyridine Synthesis (45 mg, 2%). ^{1}H-NMR (400 MHz, DMSO-d_6) δ 9.81 (s, 1H), 8.60 (s, 1H), 3.32 (s, 3H). ES-MS $[M+H]^+$ = 213.2 and 215.2.

Intermediate Example 53a. 5,6-Dihydro-4*H*-pyrrolo[1,2-*b*]pyrazole-3-sulfonyl chloride

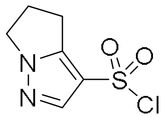

Step 1. Sulfur trioxide dimethylformamide complex (850 mg, 5.55 mmol, 1.2 eq) was added to a slurry of 5,6-dihydro-4*H*-pyrrolo[1,2-*b*]pyrazole (500 mg, 4.62 mmol, 1.0 eq) in DCE (12 mL) under N_2. The reaction was heated to 85 ºC for overnight and then cooled to room temperature. **Step 2.** Thionyl chloride (0.4 mL, 5.55 mmol, 1.2 eq) was added dropwise and the reaction was slowly heated over the course of 1 h, by which time it had reached 75 ºC. The mixture was allowed to cool to room temperature and CH_2Cl_2 (5 mL) and H_2O (3 mL) were added. The organic extract was separated, filtered through a phase separator and concentrated to afford the crude mixture of title compound (1186.5 mg). This crude mixture of title compound was used for the next step without further purification. ES-MS $[M+H]^+$ = 207.0.

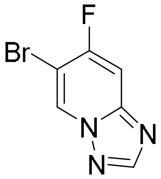

6-bromo-7-fluoro-[1,2,4]triazolo[1,5-*a*]pyridine

The title compound was prepared from 5-bromo-4-fluoroyridin-2-amine in 90% yield over 2 steps according to General Procedure. ^{1}H NMR (400 MHz, $CDCl_3$) δ 8.81 (d, J = 6.1 Hz, 1H), 8.32 (s, 1H), 7.49 (d, J = 8.0 Hz, 1H). ^{13}C NMR (101 MHz, $CDCl_3$) δ 159.4 (d, J = 258.6 Hz, 1C), 155.6, 150.6 (d, J = 13.1 Hz, 1C), 130.7 (d, J = 3.0 Hz, 1C), 101.4 (d, J = 25.3 Hz, 1C), 100.1 (d, J = 27.3 Hz, 1C). ES-MS $[M+H]^+$ = 216.2 and 218.2.

***tert*-Butyl 4-(7-fluoro-[1,2,4]triazolo[1,5-*a*]pyridin-6-yl)-3,6-dihydropyridine-1(2*H*)-carboxylate**

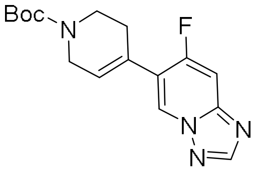

Step A. *tert*-Butyl 4-(7-fluoro-[1,2,4]triazolo[1,5-*a*]pyridin-6-yl)-3,6-dihydropyridine-1(2*H*)-carboxylate. 6-Bromo-7-fluoro-[1,2,4]triazolo[1,5-*a*]pyridine (498 mg, 2.31 mmol, 1.2 eq), *N*-Boc-3,6-dihydro-2*H*-pyridine-4-boronic acid pinacol ester (600 mg, 1.94 mmol, 1.0 eq), Pd(dppf)Cl_2•DCM (159 mg, 0.19 mmol, 0.1 eq), and Na_2CO_3 (629 mg, 5.82 mmol, 3.0 eq) were added to a microwave vial. The reaction mixture was purged with N_2. A 1,4-dioxane/H_2O solution (7:1) (8 mL, degassed) was then added via syringe. The resulting mixture was heated in a microwave reactor at 140 ºC for 30 min, after which time the reaction was cooled to room temperature and the reaction mixture was diluted with H_2O (3 mL) and extracted with CH_2Cl_2 (3 x 10 mL). The combined extracts were dried over Na_2SO_4, filtered, and concentrated to dryness. The crude residue was then purified by column chromatography (0-100% EtOAc in hexanes to 0-20% MeOH in CH_2Cl_2) to give the title compound (464.0 mg, 75%). ^{1}H-NMR (400 MHz, $CDCl_3$) δ 8.42 (d, J = 6.7 Hz, 1H), 8.21 (s, 1H), 7.30 (d, J = 10.3 Hz, 1H), 5.98 (s, 1H), 4.08 – 3.98 (m, 2H), 3.58 (t, J = 5.6 Hz, 2H), 2.43 (s, 2H), 1.41 (s, 9H). ES-MS $[M+H]^+$ = 319.0.

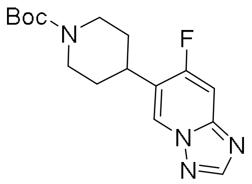

Step B. *tert*-Butyl 4-(7-fluoro-[1,2,4]triazolo[1,5-*a*]pyridin-6-yl)piperidine-1-carboxylate. *tert*-Butyl 4-(7-fluoro-[1,2,4]triazolo[1,5-*a*]pyridin-6-yl)-3,6-dihydro-2*H*-pyridine-1-carboxylate (464 mg, 1.46 mmol, 1.0 eq) was dissolved in EtOH (4 mL), and aqueous ammonium formate solution (1g/mL) (1.7 mL, 26.6 mmol, 18.3 eq) and 20%wt $Pd(OH)_2/C$ (102 mg, 0.15 mmol, 0.1 eq, 20%w/w) were added. The reaction mixture was purged with H_2. The reaction mixture was heated at 70 ºC in a microwave vial for 2 h. The reaction mixture was cooled to room temperature and solvents were filtered and concentrated under reduced pressure. The crude residue was diluted with CH_2Cl_2 (10 mL) and H_2O (2 mL), and extracted with CH_2Cl_2 (3 x 10 mL). The combined extracts were dried over Na_2SO_4, filtered, and concentrated to dryness. The crude residue was then purified by column chromatography (0-100% EtOAc in hexanes) to give the title compound (436.5 mg, 93%). ^1H NMR (400 MHz, $CDCl_3$) δ 8.47 (d, J = 6.5 Hz, 1H), 8.30 (s, 1H), 7.41 (d, J = 9.9 Hz, 1H), 4.33 (s, 2H), 3.03 (ddd, J = 12.7, 9.0, 3.6 Hz, 1H), 2.91 (s, 2H), 1.98 (d, J = 12.8 Hz, 2H), 1.75 - 1.60 (m, 2H), 1.50 (s, 9H). ES-MS $[M+H]^+$ = 321.4.

Intermediate Example 48b. *tert*-Butyl 4-(7-methyl-[1,2,4]triazolo[1,5-*b*]pyridazin-6-yl)piperidine-1-carboxylate

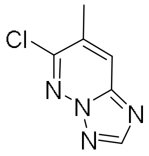

Step A. 6-Chloro-7-methyl-[1,2,4]triazolo[1,5-*b*]pyridazine. Step 1. 6-Chloro-5-methylpyridazin-3-amine (1.44 g, 10 mmol, 1 eq) was dissolved in 2-propanol (20 mL, 0.4 M) and *N,N*-dimethylformamide dimethyl acetal (1.7 mL, 13.0 mmol, 1.3 eq) was added dropwise. The resulting solution was heated at 82 ºC for 3 h to provide the *N,N*-dimethyl formamidine intermediate (ES-MS $[M+H]^+$ = 199.2). After cooling to 50 ºC, hydroxylamine hydrochloride (903 mg, 13.0 mmol, 1.3 eq) was added. The reaction mixture was stirred at 50 ºC for 2 h and concentrated under reduced pressure to provide the *N*'-hydroxy-formamidine intermediate (ES-MS $[M+H]^+$ = 187.2) which was used for the next step without further purification. **Step 2.** The crude mixture of *N*'-(6-chloro-5-methylpyridazin-3-yl)-*N*-hydroxyformimidamide (1.87 g, 10.0 mmol, 1 eq) was suspended in THF (50 mL). The resulting suspension was cooled to 0 ºC and trifluoroacetic anhydride (4.2 mL, 30.0 mmol, 3.0 eq) was added dropwise. The reaction mixture was allowed to warm to room temperature and stirred overnight. The precipitate was filtered using a Büchner funnel and washed

with cold THF to provide a 1st batch of the title compound as a white solid. The filtrate was concentrated under reduced pressure and purified using column chromatography (50-80% EtOAc in CH_2Cl_2) to give a 2nd batch of the title compound. Two batches were combined (1.34 g, 79% over 2 steps). ^{1}H-NMR (400 MHz, DMSO-d_6) δ 8.65 (s, 1H), 8.48 (J = 1.0 Hz, 1H), 2.49 (J = 1.0 Hz, 3H). ES-MS $[M+H]^+$ = 169.2.

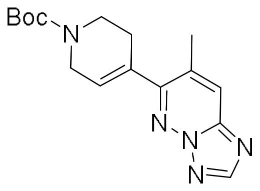

Step B. *tert*-Butyl 4-(7-methyl-[1,2,4]triazolo[1,5-*b*]pyridazin-6-yl)-3,6-dihydropyridine-1(2*H*)-carboxylate. 6-Chloro-7-methyl-[1,2,4]triazolo[1,5-*b*]pyridazine (506 mg, 3.0 mmol, 1 eq.), *N*-Boc-3,6-dihydro-2*H*-pyridine-4-boronic acid pinacol ester (1.21 g, 3.9 mmol, 1.3 eq.), Pd(dppf)Cl_2•DCM (246 mg, 0.3 mmol, 0.1 eq), and Na_2CO_3 (972 mg, 9.0 mmol, 3 eq) were charged into a microwave vial which was sealed and placed under an inert atmosphere. 1,4-Dioxane (10 mL) and H_2O (5 mL) were added via syringe and the reaction mixture was purged with N_2 and subjected to microwave radiation at 140 °C. After 30 min, the reaction mixture was filtered through a pad of Celite which was rinsed thoroughly with EtOAc/CH_2Cl_2. The filtrate was concentrated under reduced pressure and purified using column chromatography (0-100% EtOAc in hexanes) to provide the title compound (785.0 mg, 83%). ^{1}H-NMR (400 MHz, DMSO-d_6) δ 8.57 (s, 1H), 8.28 (d, J = 1.0 Hz, 1H), 6.11 (s, 1H), 4.05 – 4.09 (m, 2H), 3.58 (dd, J = 5.5, 5.5 Hz, 2H), 2.43 – 2.50 (m, 5H), 1.45 (s, 9H). ES-MS $[M+H]^+$ = 316.4.

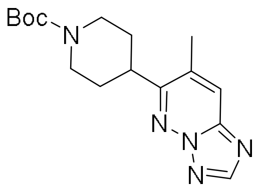

Step C. *tert*-Butyl 4-(7-methyl-[1,2,4]triazolo[1,5-*b*]pyridazin-6-yl)piperidine-1-carboxylate. *tert*-Butyl 4-(7-methyl-[1,2,4]triazolo[1,5-*b*]pyridazin-6-yl)-3,6-dihydropyridine-1(2*H*)-carboxylate (785 mg, 2.50 mmol, 1.0 eq.) Pd$(OH)_2$/C (175 mg, 0.25 mmol, 0.1 eq, 20%w/w), and aqueous ammonium formate solution (1g/mL) (2.5 mL, 45.0 mmol, 18 eq) in H_2O were added to a vial, which was sealed and placed under a H_2 atmosphere. EtOH (10 mL) was added by syringe, and the mixture was heated at 50 °C for 2 h. The resulting mixture was filtered through Celite and washed with MeOH and concentrated under reduced pressure. The residue was then diluted with CH_2Cl_2 (3 mL) and H_2O (1 mL) and extracted with CH_2Cl_2 (3 x 5 mL). The combined organics were passed through a phase separator, concentrated under reduced pressure. The crude mixture of title compound was used for the next step without further purification. (790

mg). ^{1}H-NMR (400 MHz, $CDCl_3$) δ 8.41 (s, 1H), 7.91 (s, 1H), 4.42 – 4.19 (m, 2H), 3.06 (m, 1H), 2.87 (m, 2H), 2.54 (s, 3H), 2.24 (m, 2H), 1.97 (m, 2H), 1.49 (s, 9H). ES-MS $[M+H]^+$ = 318.4.

Intermediate Characterizations – ^{1}H-NMR and/or ^{13}C-NMR Spectra

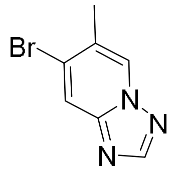

Chemical Formula: $C_7H_6BrN_3$

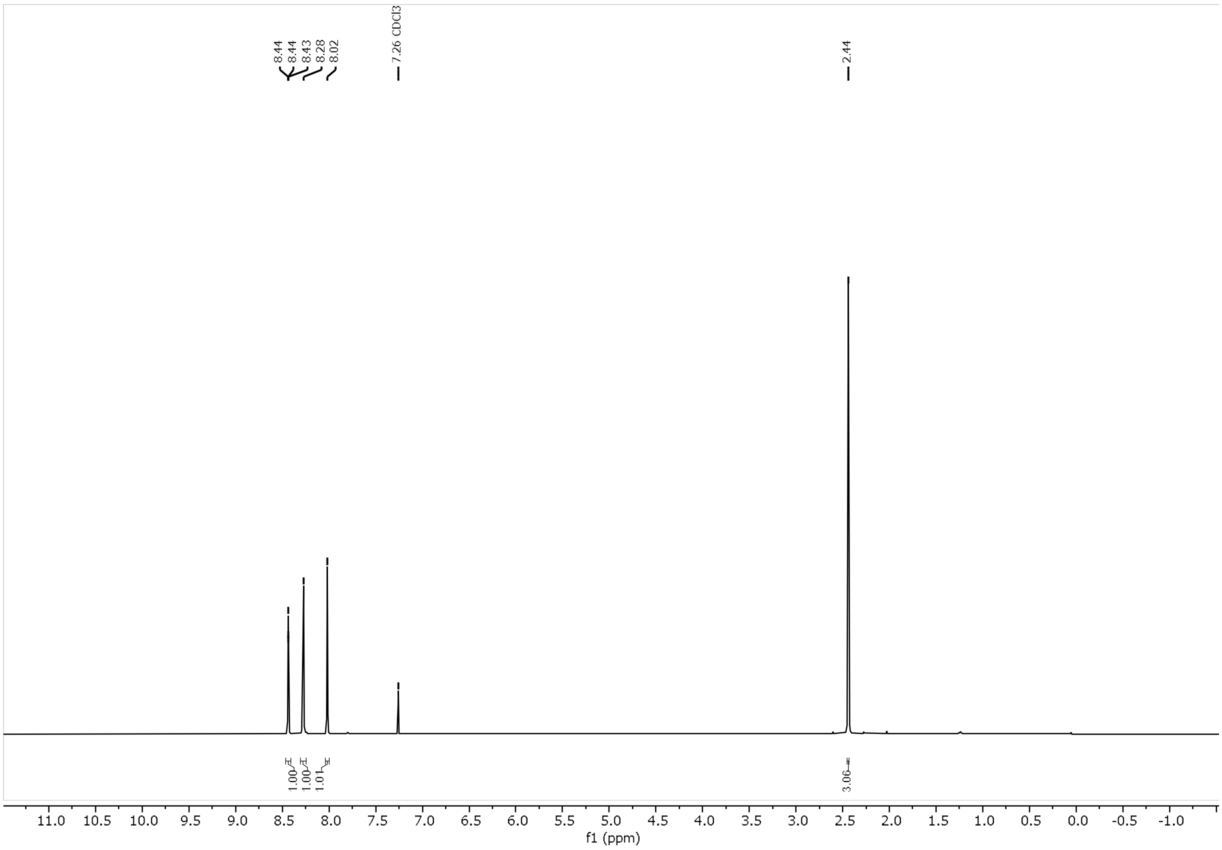

^{1}H NMR (400 MHz, $CDCl_3$) δ 8.46 – 8.41 (m, 1H), 8.28 (s, 1H), 8.02 (s, 1H), 2.44 (s, 3H).

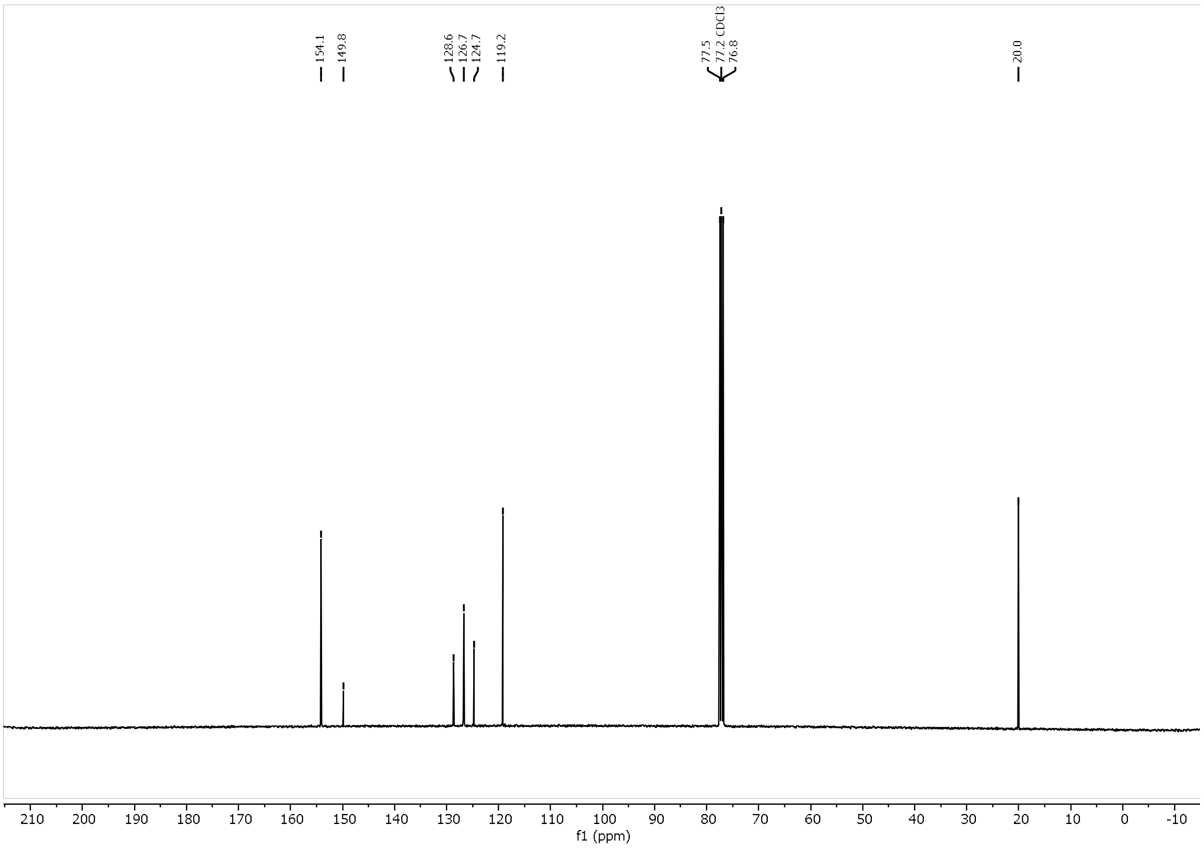

^{13}C NMR (101 MHz, $CDCl_3$) δ 154.1, 149.8, 128.6, 126.7, 124.7, 119.2, 20.0.

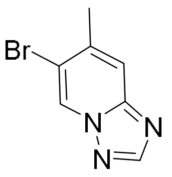

Chemical Formula: $C_7H_6BrN_3$

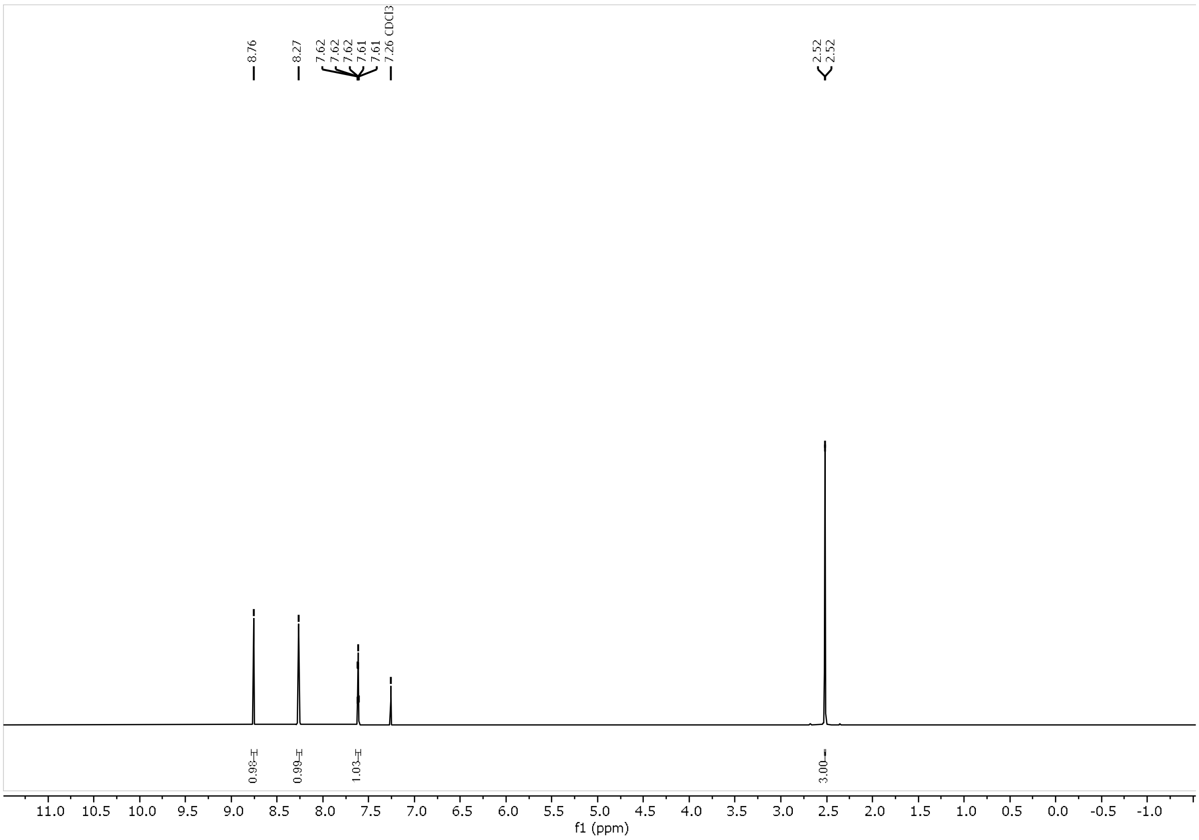

^{1}H NMR (400 MHz, $CDCl_3$) δ 8.76 (s, 1H), 8.27 (s, 1H), 7.62 (s, 1H), 2.52 (d, J = 1.0 Hz, 3H).

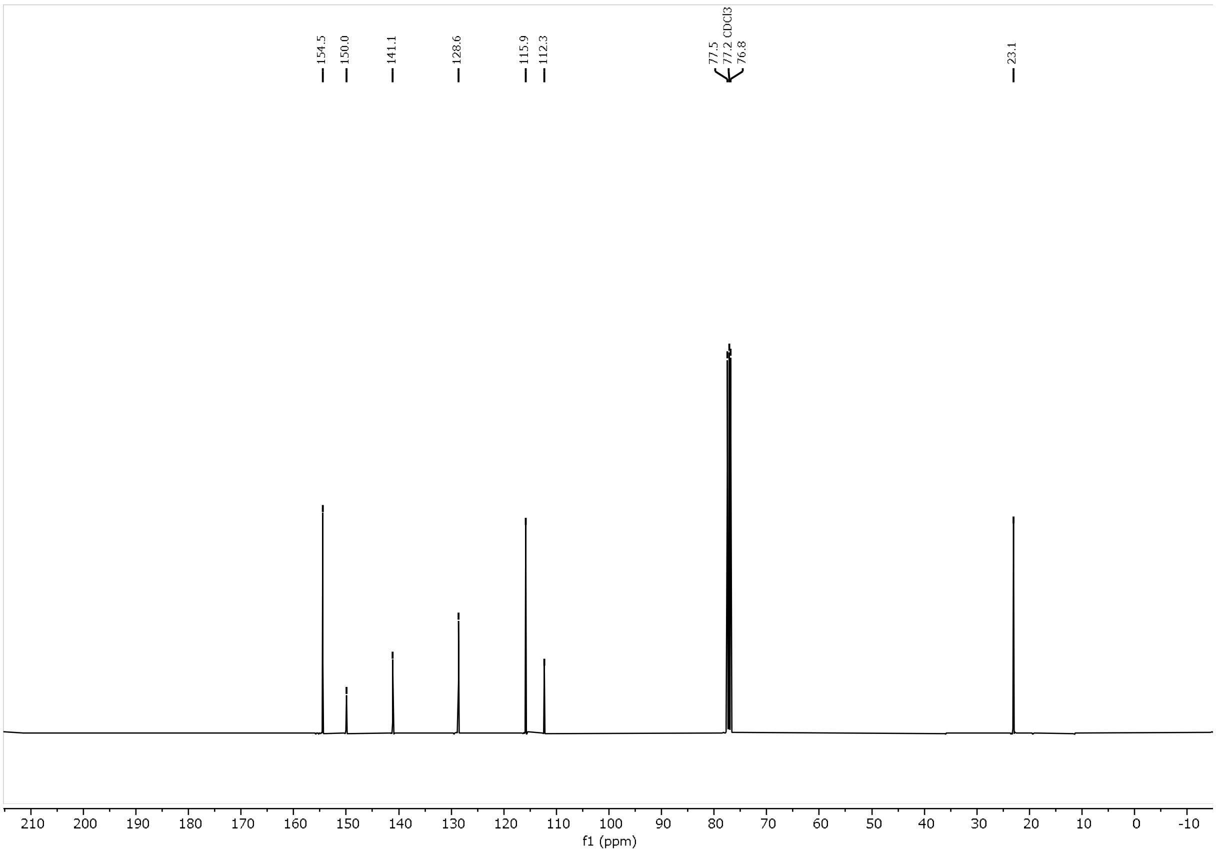

^{13}C NMR (101 MHz, $CDCl_3$) δ 154.5, 150.0, 141.1, 128.7, 115.9, 112.3, 23.1.

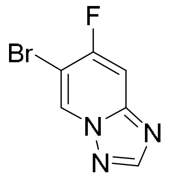

Chemical Formula: $C_6H_3BrFN_3$

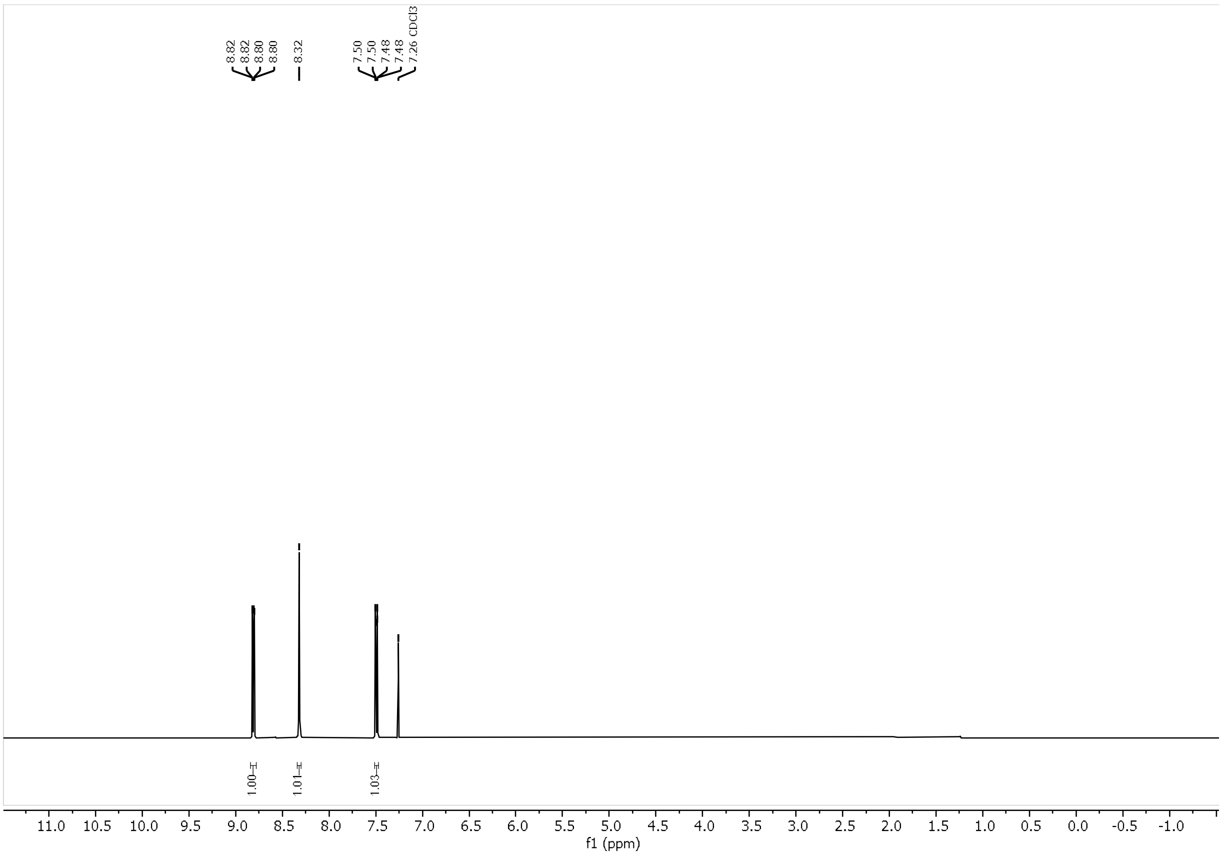

^{1}H NMR (400 MHz, $CDCl_3$) δ 8.81 (d, J = 6.1 Hz, 1H), 8.32 (s, 1H), 7.49 (d, J = 8.0 Hz, 1H).

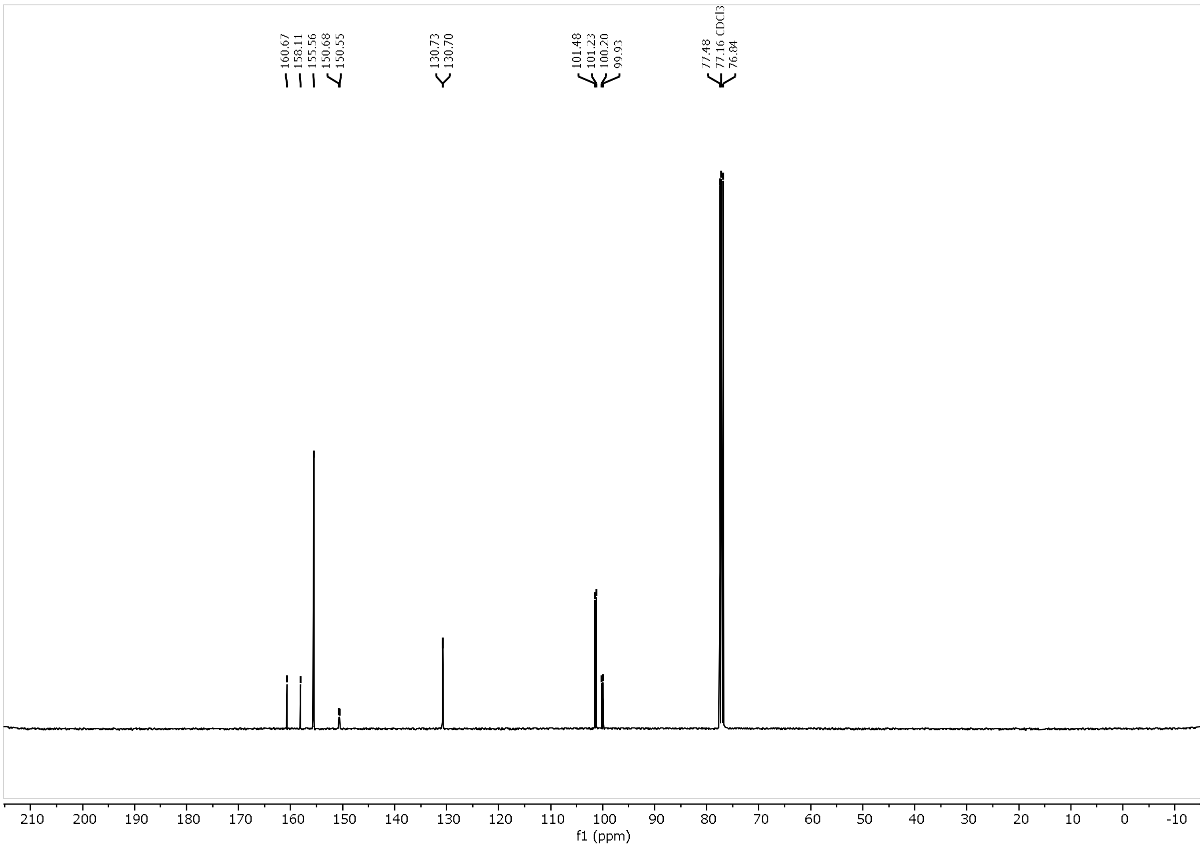

^{13}C NMR (101 MHz, $CDCl_3$) δ 159.4 (d, *J* = 258.6 Hz, 1C), 155.6, 150.6 (d, *J* = 13.1 Hz, 1C), 130.7 (d, *J* = 3.0 Hz, 1C), 101.4 (d, *J* = 25.3 Hz, 1C), 100.1 (d, *J* = 27.3 Hz, 1C).

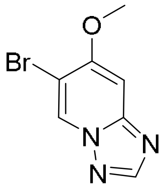

Chemical Formula: $C_7H_6BrN_3O$

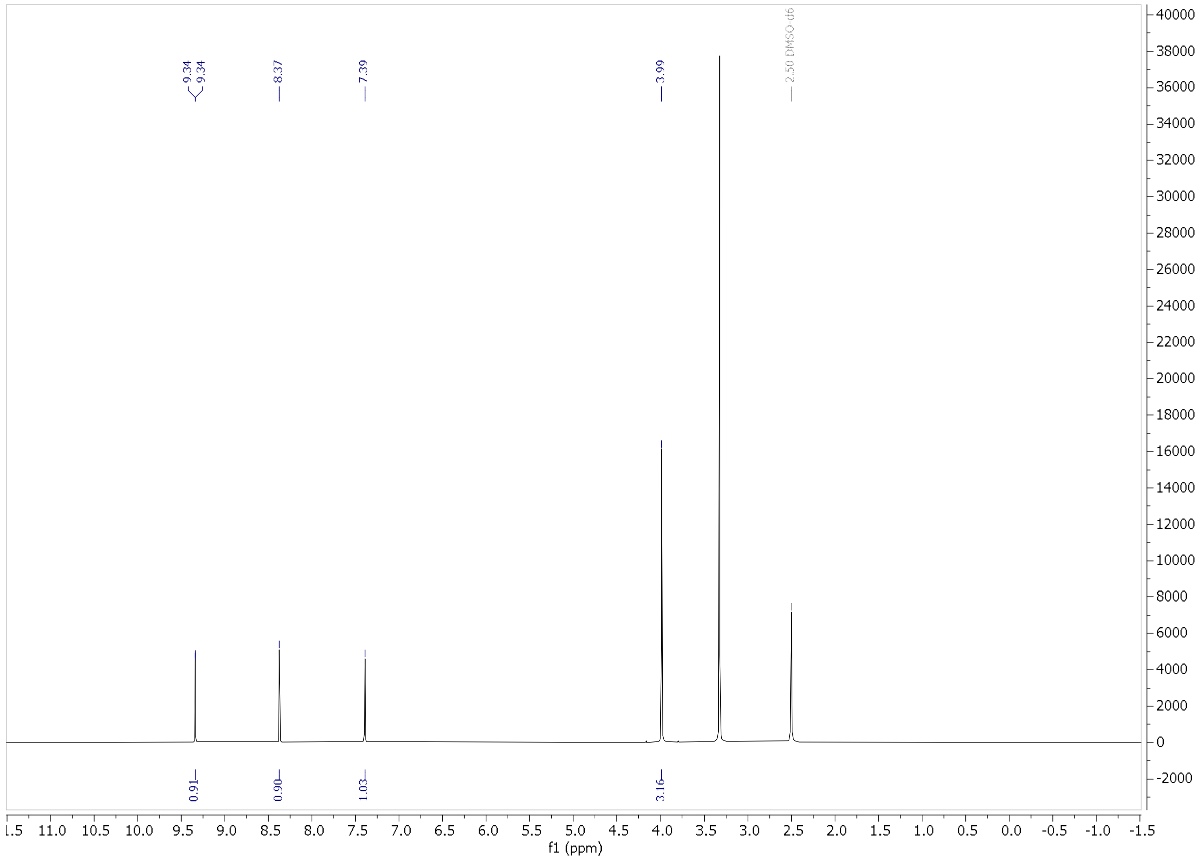

^{1}H NMR (400 MHz, DMSO) δ 9.34 (s, 1H), 8.37 (s, 1H), 7.39 (s, 1H), 3.99 (s, 3H).

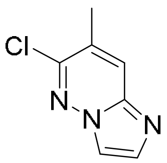

Chemical Formula: $C_7H_6ClN_3$

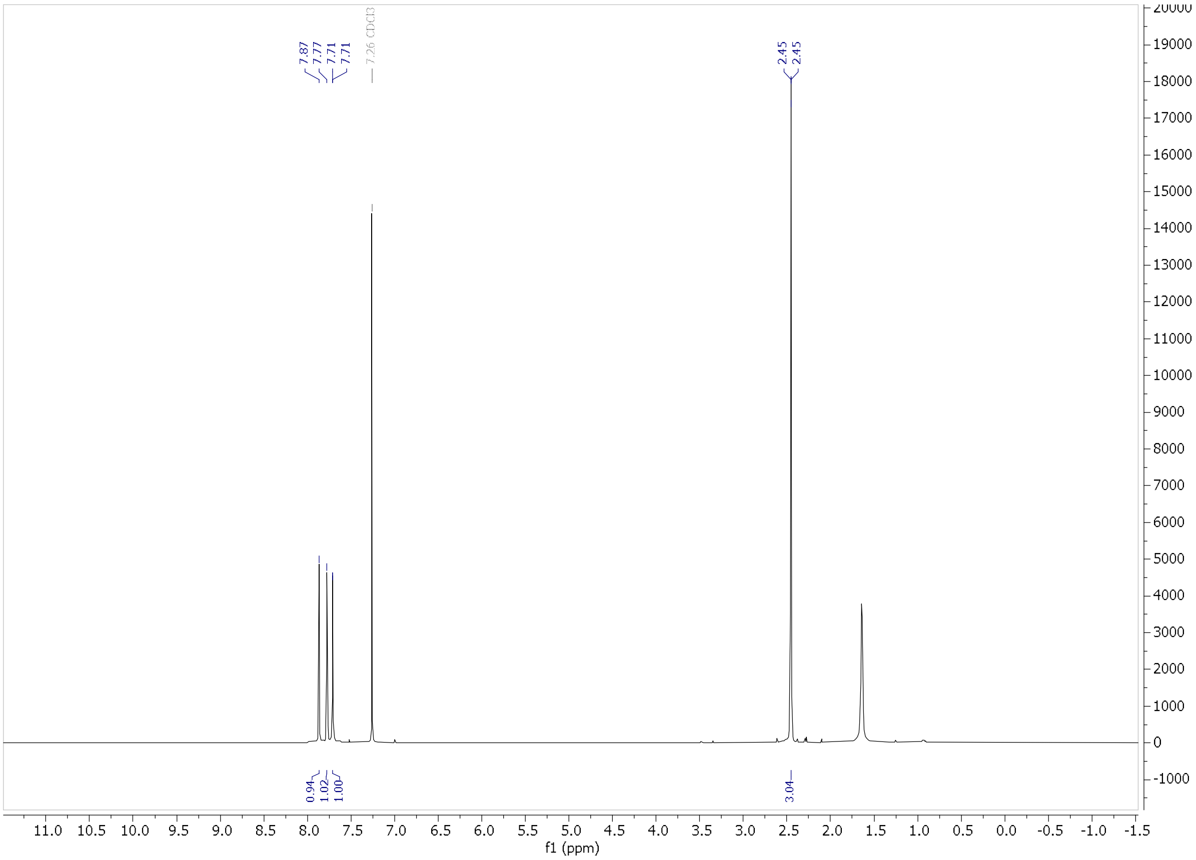

^{1}H NMR (400 MHz, $CDCl_3$) δ 7.87 (s, 1H), 7.77 (s, 1H), 7.71 (d, J = 1.3 Hz, 1H), 2.45 (d, J = 1.1 Hz, 3H).

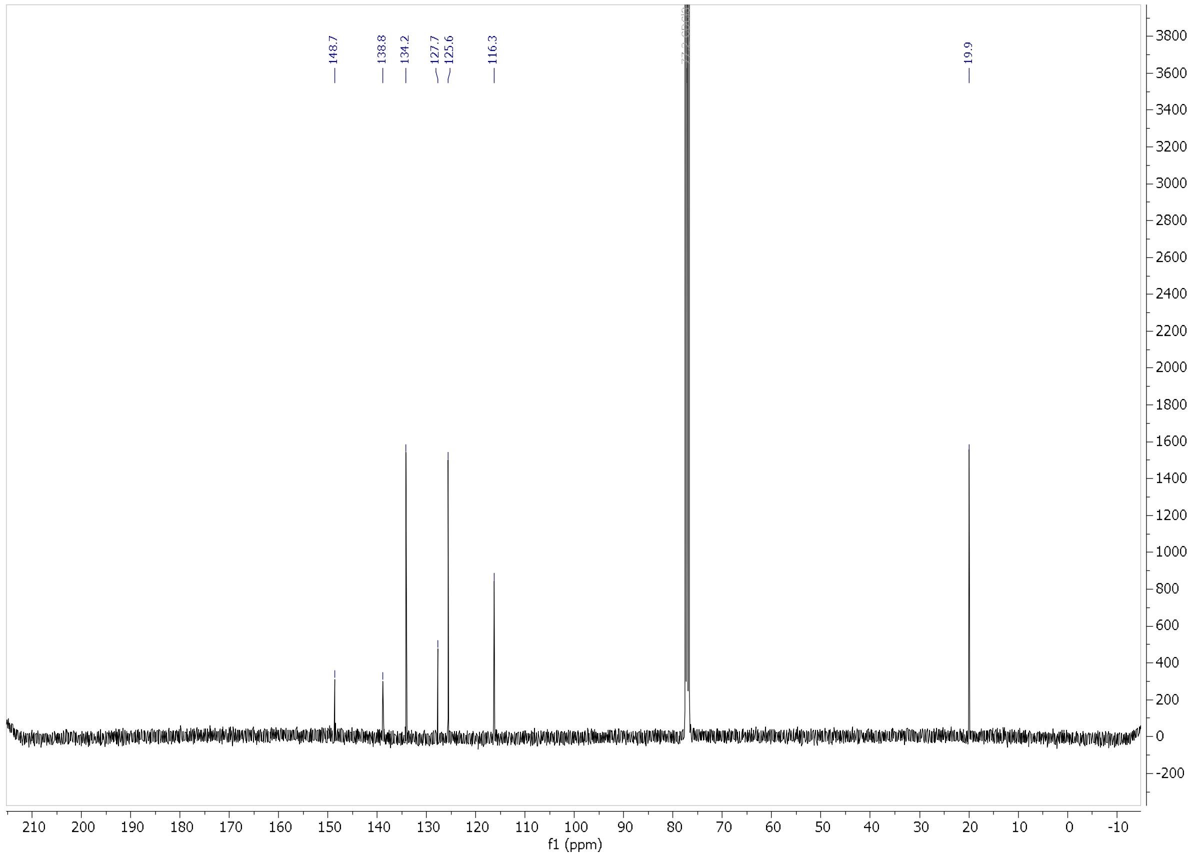

^{13}C NMR (101 MHz, $CDCl_3$) δ 148.7, 138.8, 134.2, 127.7, 125.6, 116.3, 19.9

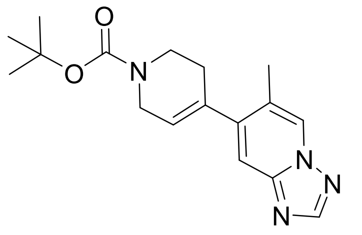

Chemical Formula: $C_{17}H_{22}N_4O_2$

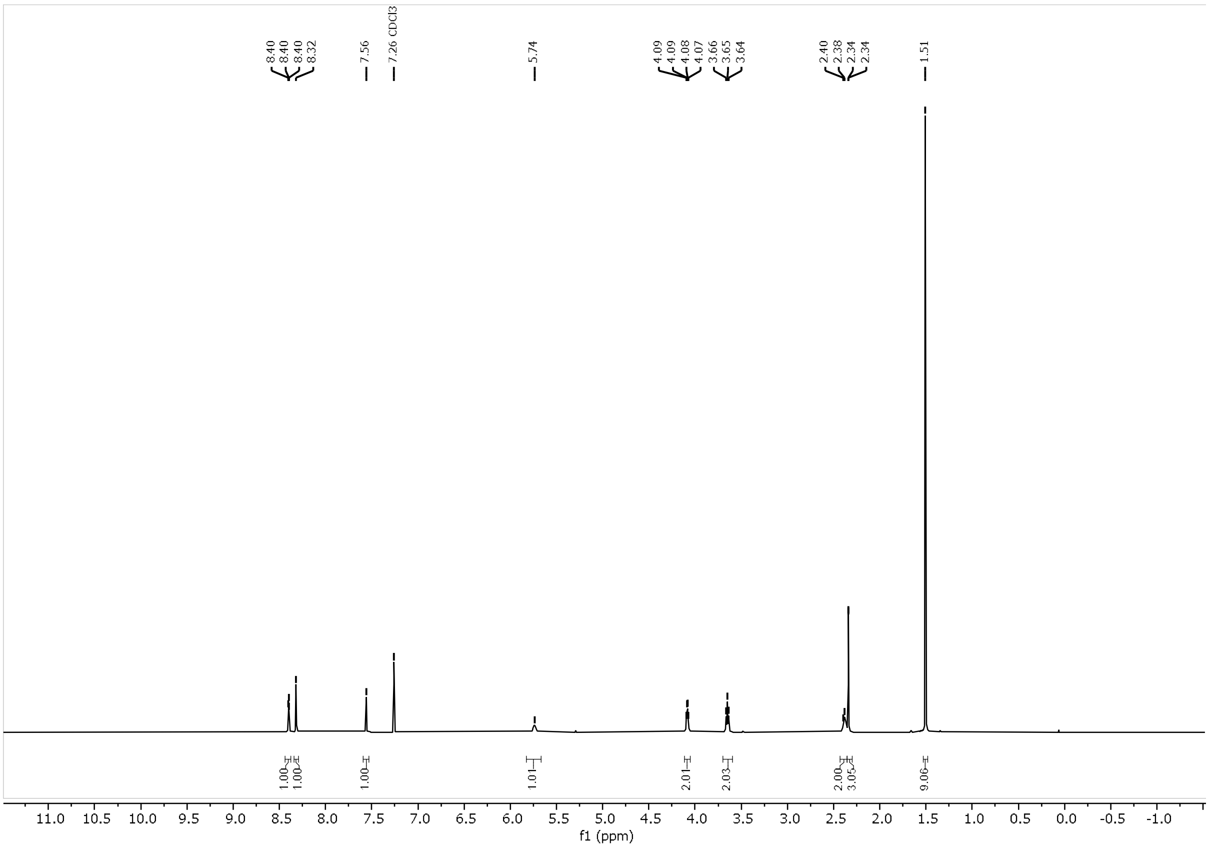

^{1}H NMR (400 MHz, $CDCl_3$) δ 8.40 (s, 1H), 8.32 (s, 1H), 7.56 (s, 1H), 5.74 (s, 1H), 4.08 (q, J = 2.9 Hz, 2H), 3.65 (t, J = 5.6 Hz, 2H), 2.41 – 2.35 (m, 2H), 2.34 (d, J = 1.1 Hz, 3H), 1.51 (s, 9H).

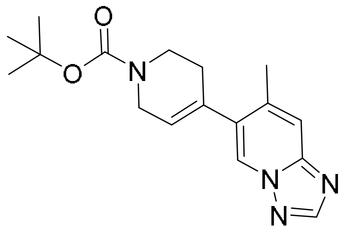

Chemical Formula: $C_{17}H_{22}N_4O_2$

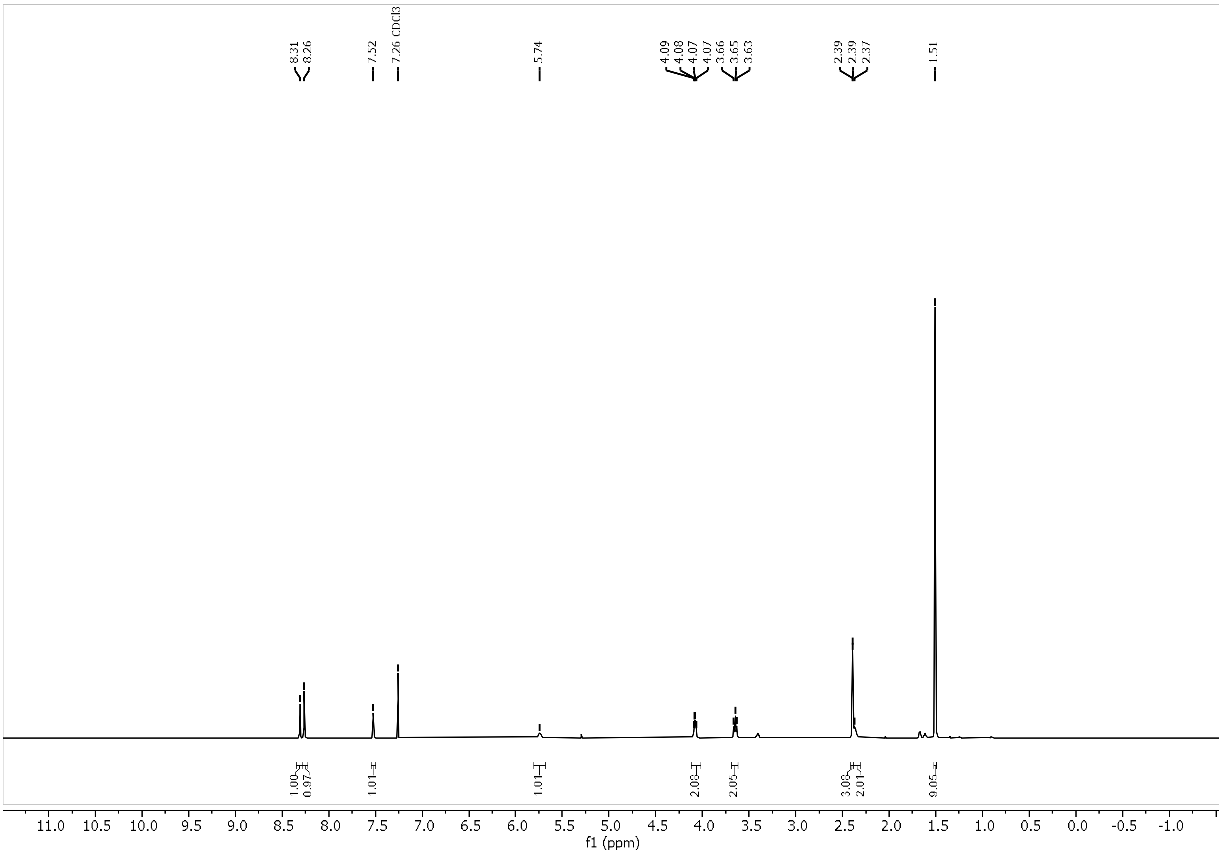

^{1}H NMR (400 MHz, $CDCl_3$) δ 8.31 (s, 1H), 8.26 (s, 1H), 7.52 (s, 1H), 5.74 (s, 1H), 4.08 (d, J = 2.9 Hz, 2H), 3.65 (t, J = 5.6 Hz, 2H), 2.39 (d, J = 1.1 Hz, 3H), 2.38 – 2.32 (s, 2H), 1.51 (s, 9H).

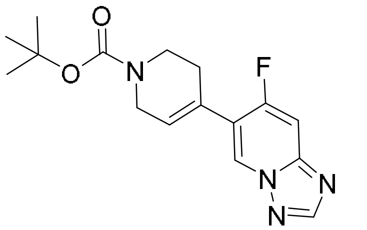

Chemical Formula: $C_{16}H_{19}FN_4O_2$

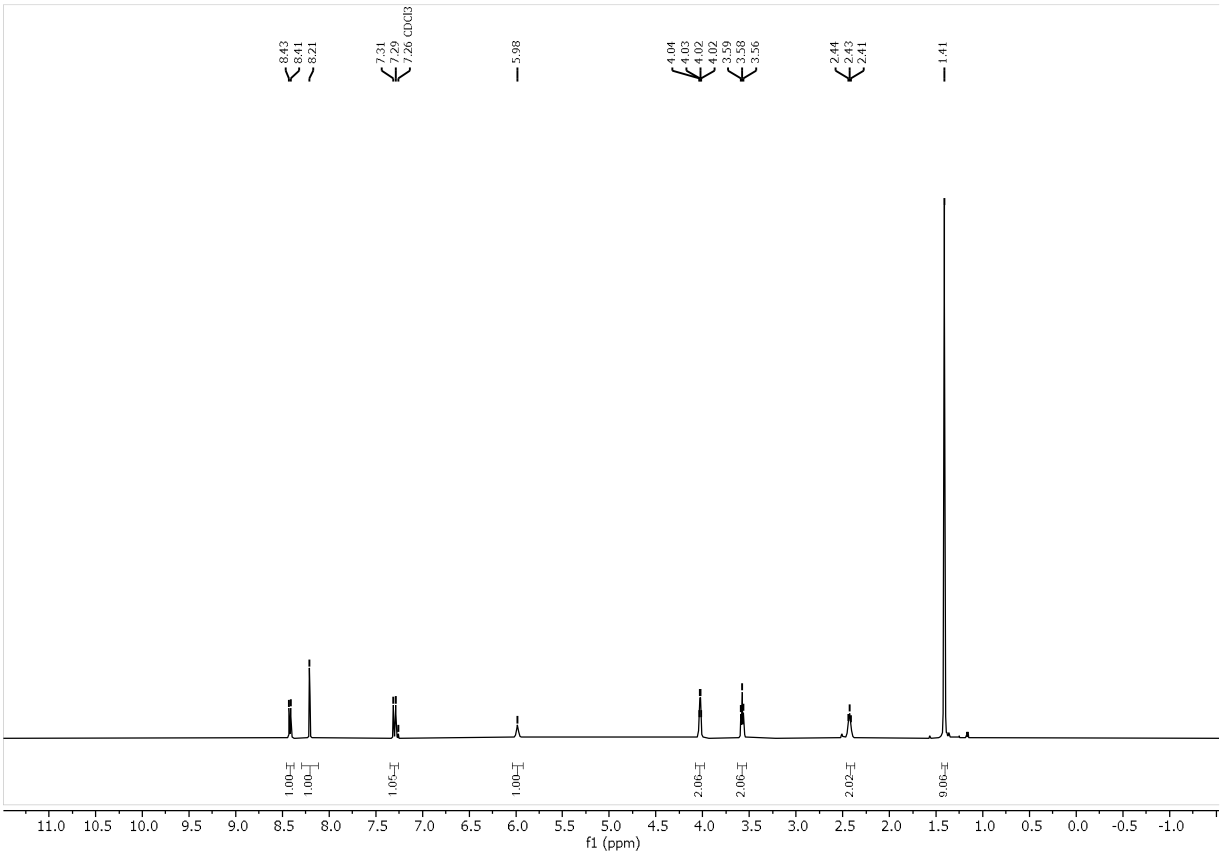

^{1}H-NMR (400 MHz, $CDCl_3$) δ 8.42 (d, J = 6.7 Hz, 1H), 8.21 (s, 1H), 7.30 (d, J = 10.3 Hz, 1H), 5.98 (s, 1H), 4.08 – 3.98 (m, 2H), 3.58 (t, J = 5.6 Hz, 2H), 2.43 (s, 2H), 1.41 (s, 9H).

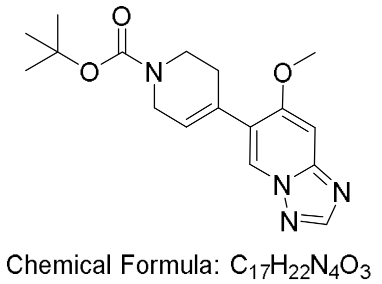

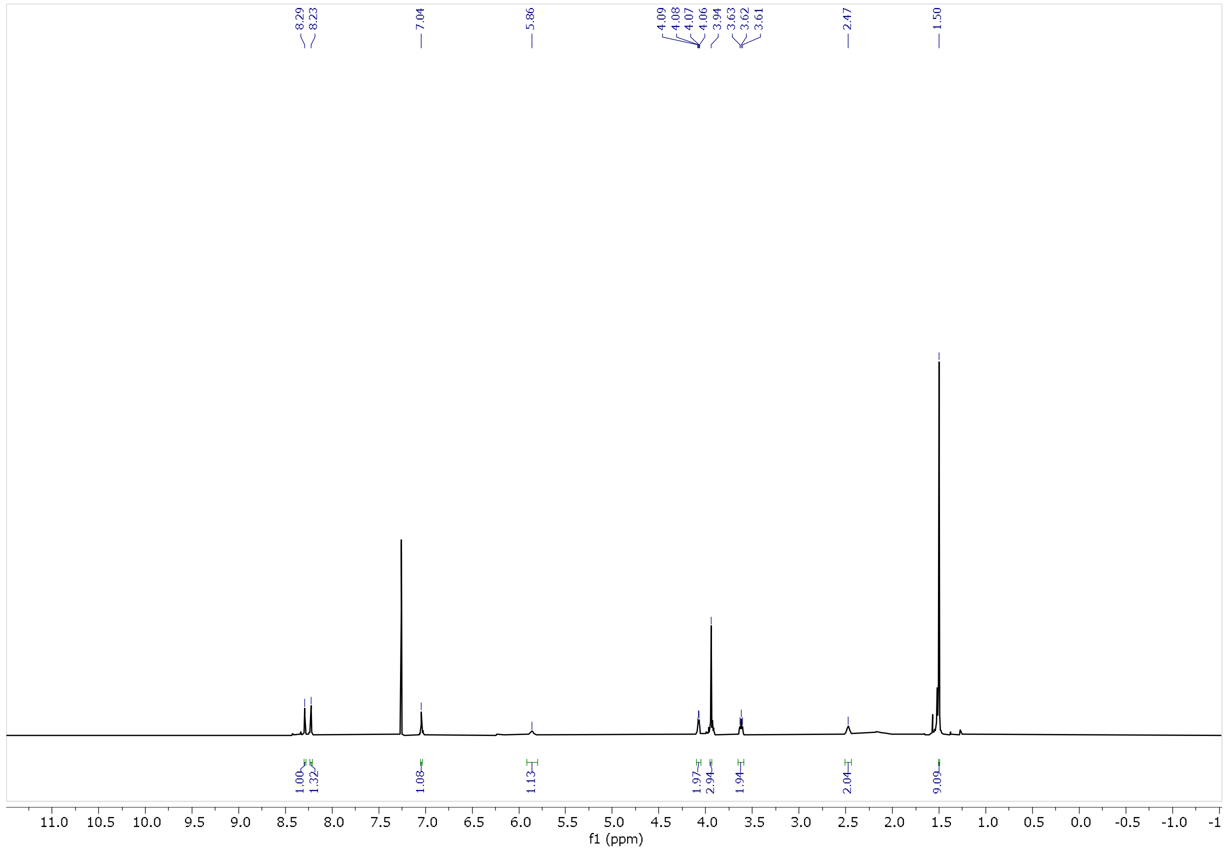

^{1}H NMR (400 MHz, $CDCl_3$) δ 8.29 (s, 1H), 8.23 (s, 1H), 7.04 (s, 1H), 5.86 (m, 1H), 4.07 (q, *J* = 2.9 Hz, 2H), 3.94 (s, 3H), 3.62 (t, *J* = 5.6 Hz, 2H), 2.47 (m, 2H), 1.50 (s, 9H).

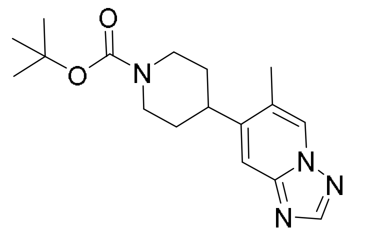

Chemical Formula: $C_{17}H_{24}N_4O_2$

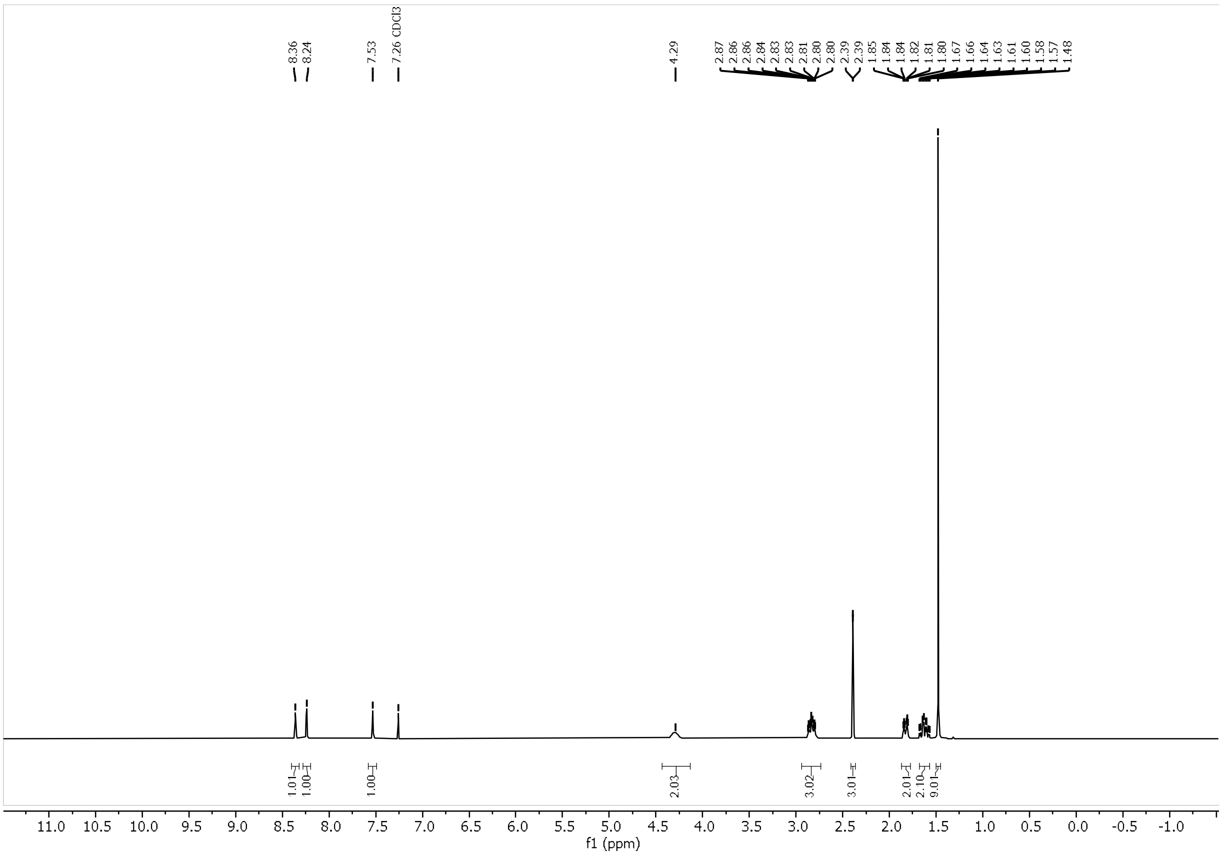

^{1}H NMR (400 MHz, $CDCl_3$) δ 8.36 (s, 1H), 8.24 (s, 1H), 7.53 (s, 1H), 4.29 (s, 2H), 2.95 – 2.74 (m, 3H), 2.39 (d, J = 1.1 Hz, 3H), 1.82 (dt, J = 13.2, 2.6 Hz, 2H), 1.68 – 1.57 (m, 2H), 1.48 (s, 9H).

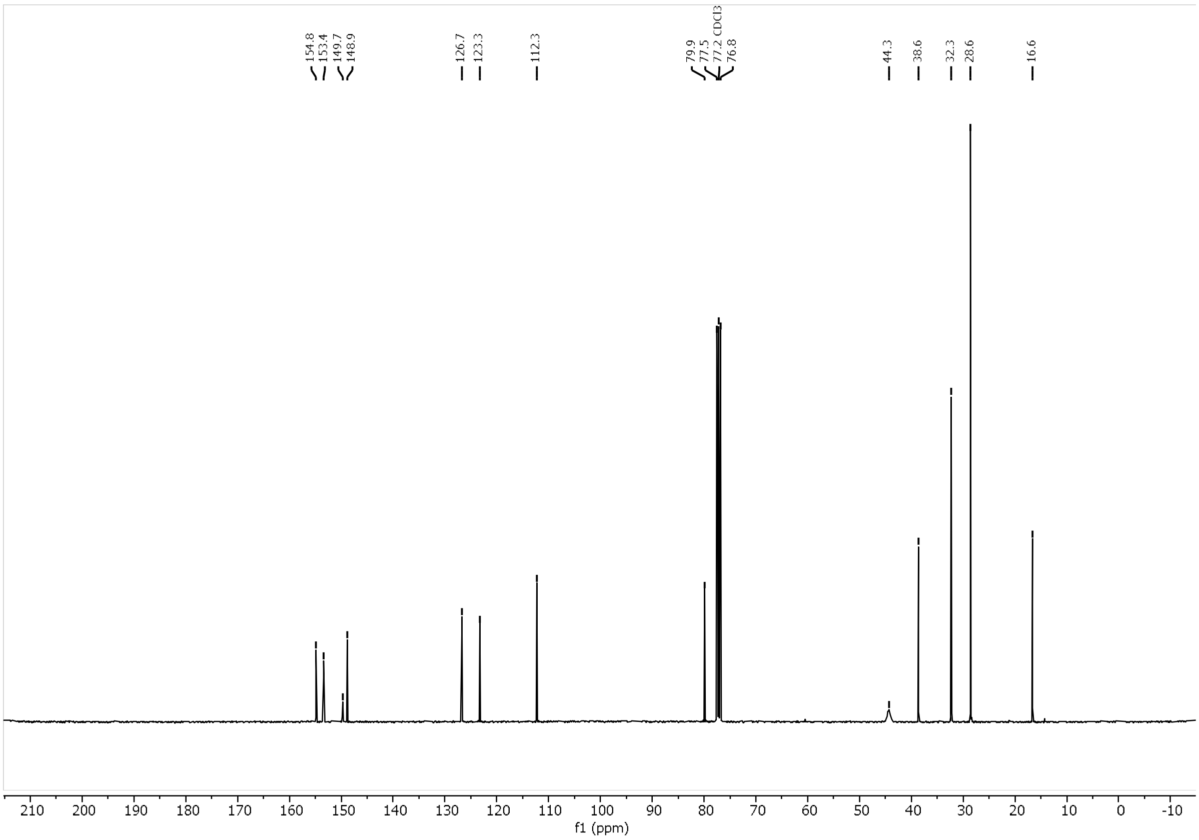

^{13}C NMR (101 MHz, $CDCl_3$) δ 154.8, 153.4, 149.7, 148.9, 126.7, 123.3, 112.3, 79.9, 44.3, 38.6 (2), 32.3 (2), 28.6 (3), 16.6.

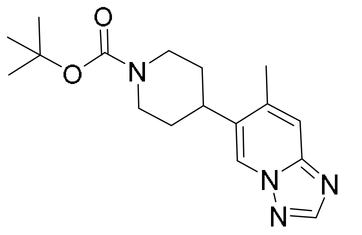

Chemical Formula: $C_{17}H_{24}N_4O_2$

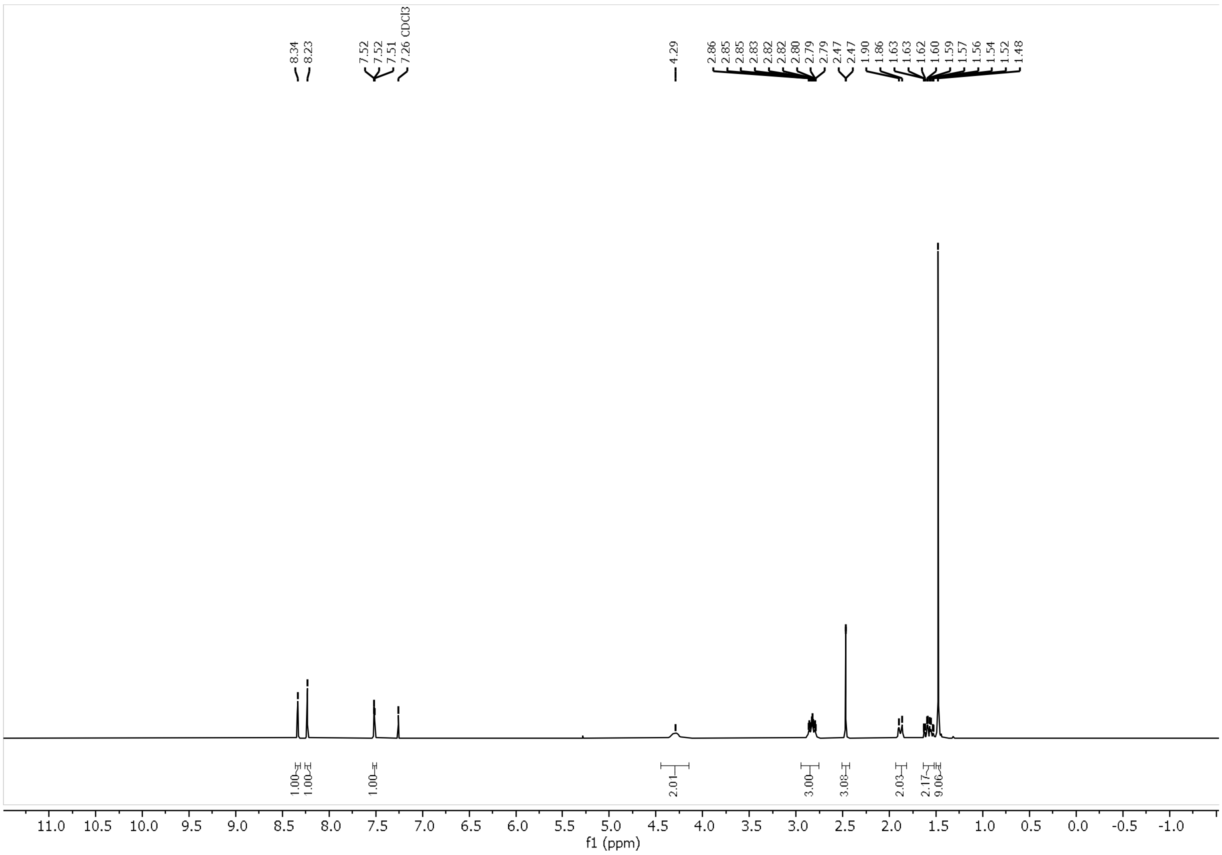

^{1}H NMR (400 MHz, $CDCl_3$) δ 8.34 (s, 1H), 8.23 (s, 1H), 7.52 (s, 1H), 4.29 (s, 2H), 2.92 - 2.75 (m, 3H), 2.47 (s, 3H), 1.88 (d, *J* = 13.1, 2H), 1.58 (qd, *J* = 12.6, 4.2 Hz, 2H), 1.48 (s, 9H).

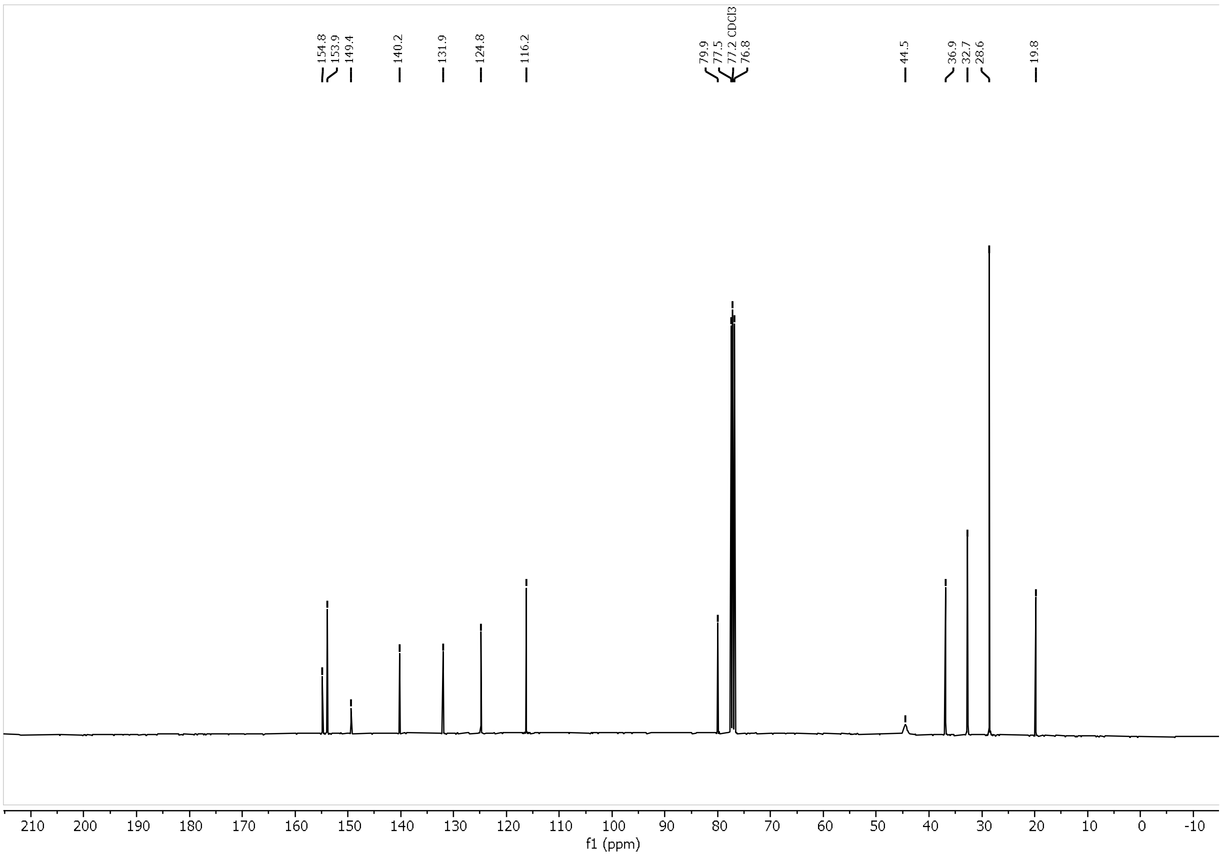

^{13}C NMR (101 MHz, $CDCl_3$) δ 154.8, 153.9, 149.4, 140.2, 131.9, 124.8, 116.2, 79.9, 44.5, 36.9 (2), 32.7 (2), 28.6 (3), 19.8.

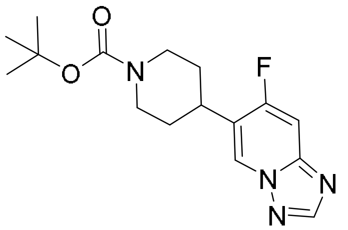

Chemical Formula: $C_{16}H_{21}FN_4O_2$

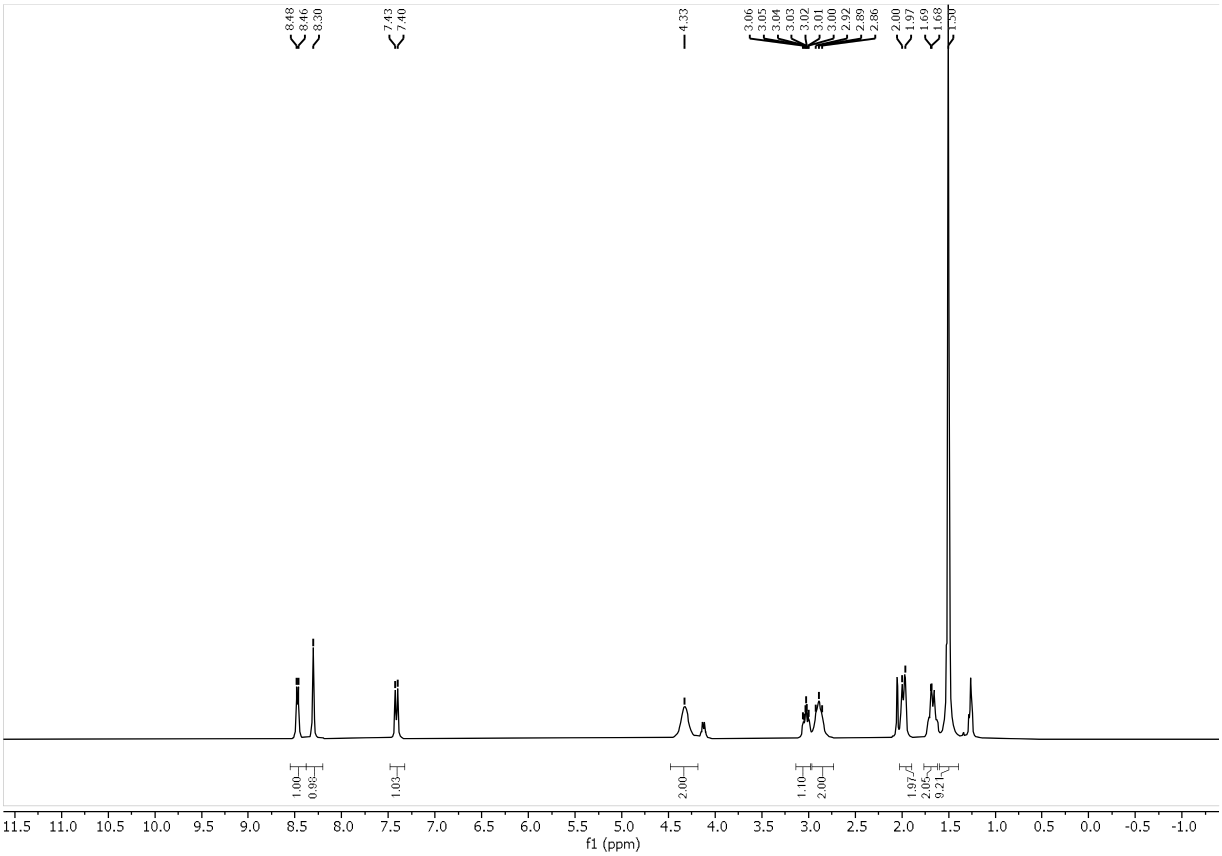

^{1}H NMR (400 MHz, $CDCl_3$) δ 8.47 (d, J = 6.5 Hz, 1H), 8.30 (s, 1H), 7.41 (d, J = 9.9 Hz, 1H), 4.33 (s, 2H), 3.03 (ddd, J = 12.7, 9.0, 3.6 Hz, 1H), 2.91 (s, 2H), 1.98 (d, J = 12.8 Hz, 2H), 1.75 - 1.60 (m, 2H), 1.50 (s, 9H).

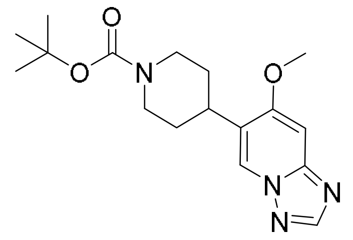

Chemical Formula: $C_{17}H_{24}N_4O_3$

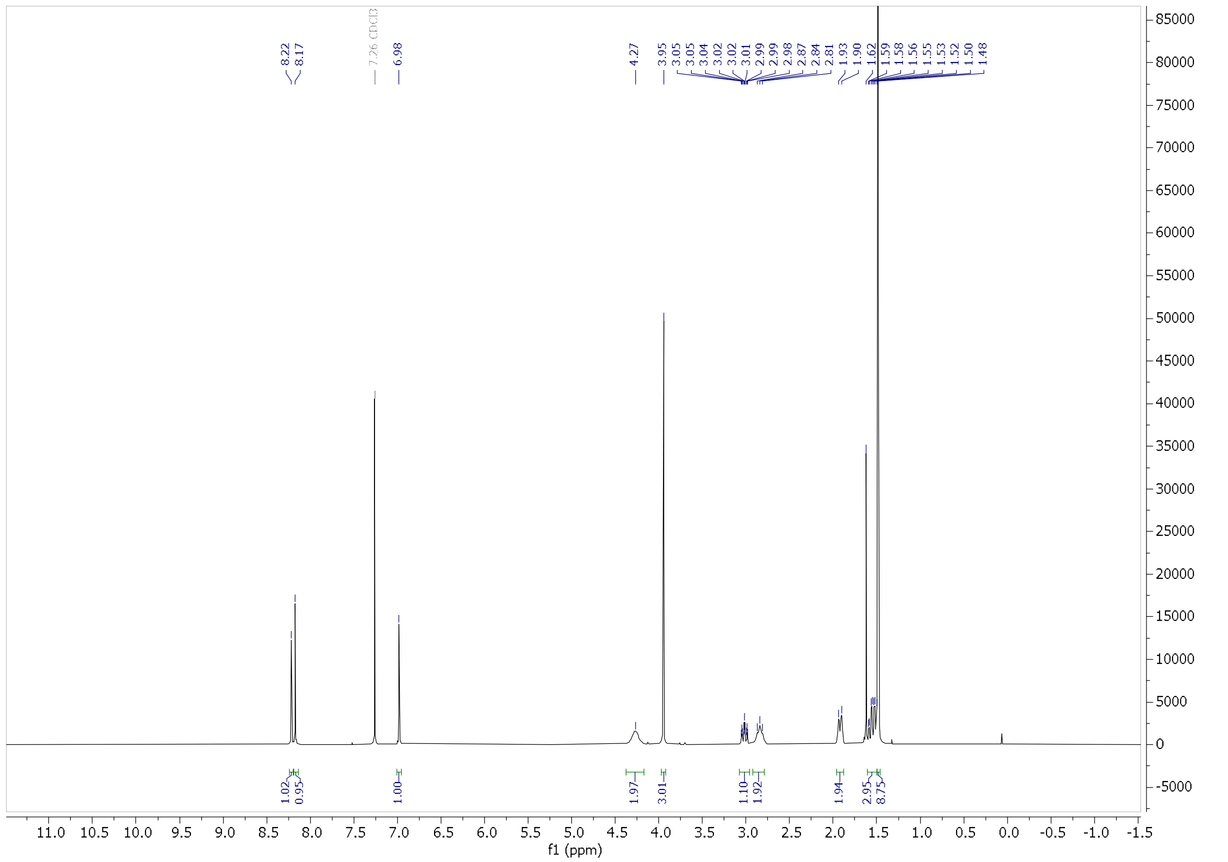

^{1}H NMR (400 MHz, $CDCl_3$) δ 8.22 (s, 1H), 8.17 (s, 1H), 6.98 (s, 1H), 4.27 (s, 2H), 3.95 (s, 3H), 3.02 (tt, J = 12.2, 3.3 Hz, 1H), 2.84 (t, J = 12.3 Hz, 2H), 1.92 (d, J = 12.7 Hz, 2H), 1.61 – 1.50 (m, 2H), 1.48 (s, 9H).

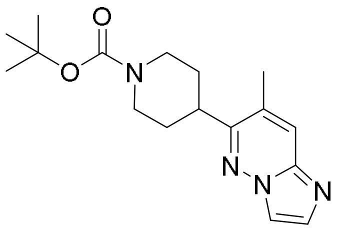

Chemical Formula: $C_{17}H_{24}N_4O_2$

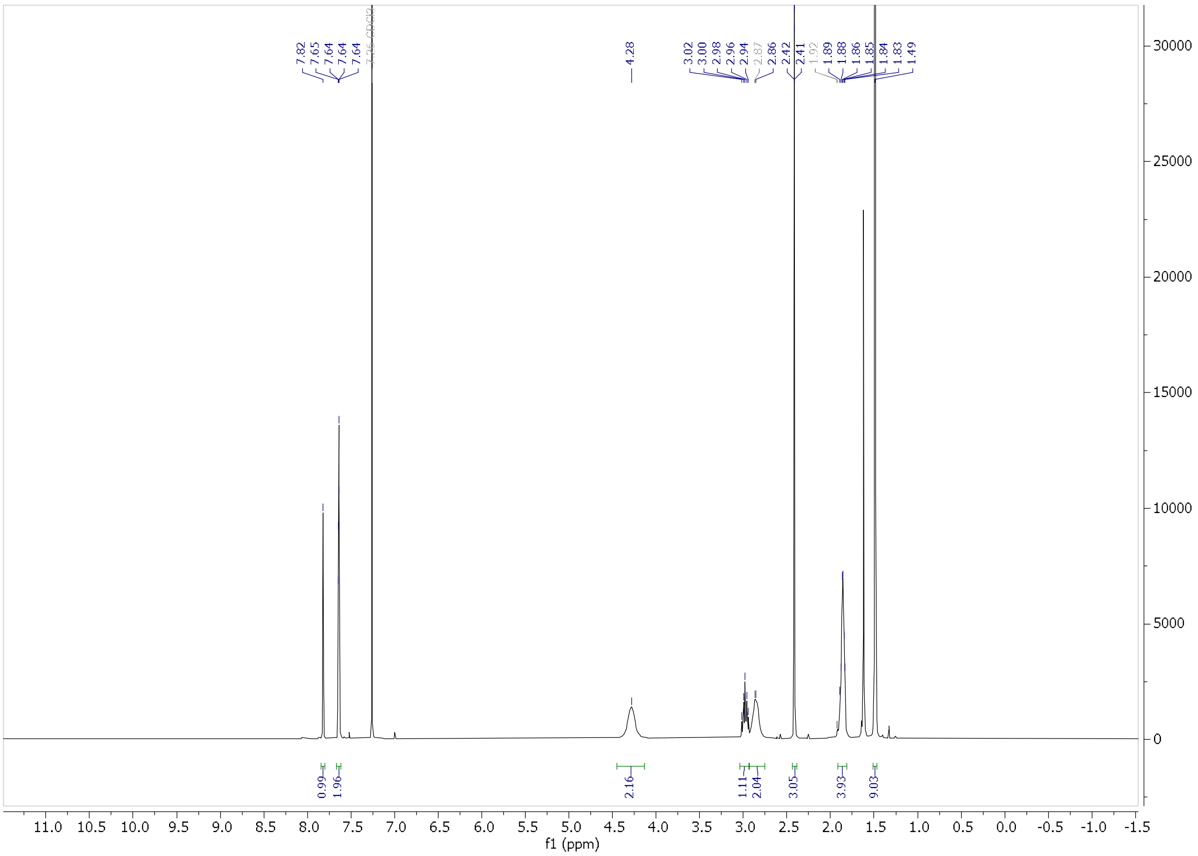

^{1}H NMR (400 MHz, $CDCl_3$) δ 7.82 (s, 1H), 7.67 – 7.61 (m, 2H), 4.45 – 4.13 (m, 2H), 2.98 (p, *J* = 7.6 Hz, 1H), 2.93 – 2.75 (m, 2H), 2.41 (d, *J* = 1.1 Hz, 3H), 1.91 – 1.81 (m, 4H), 1.49 (s, 9H)

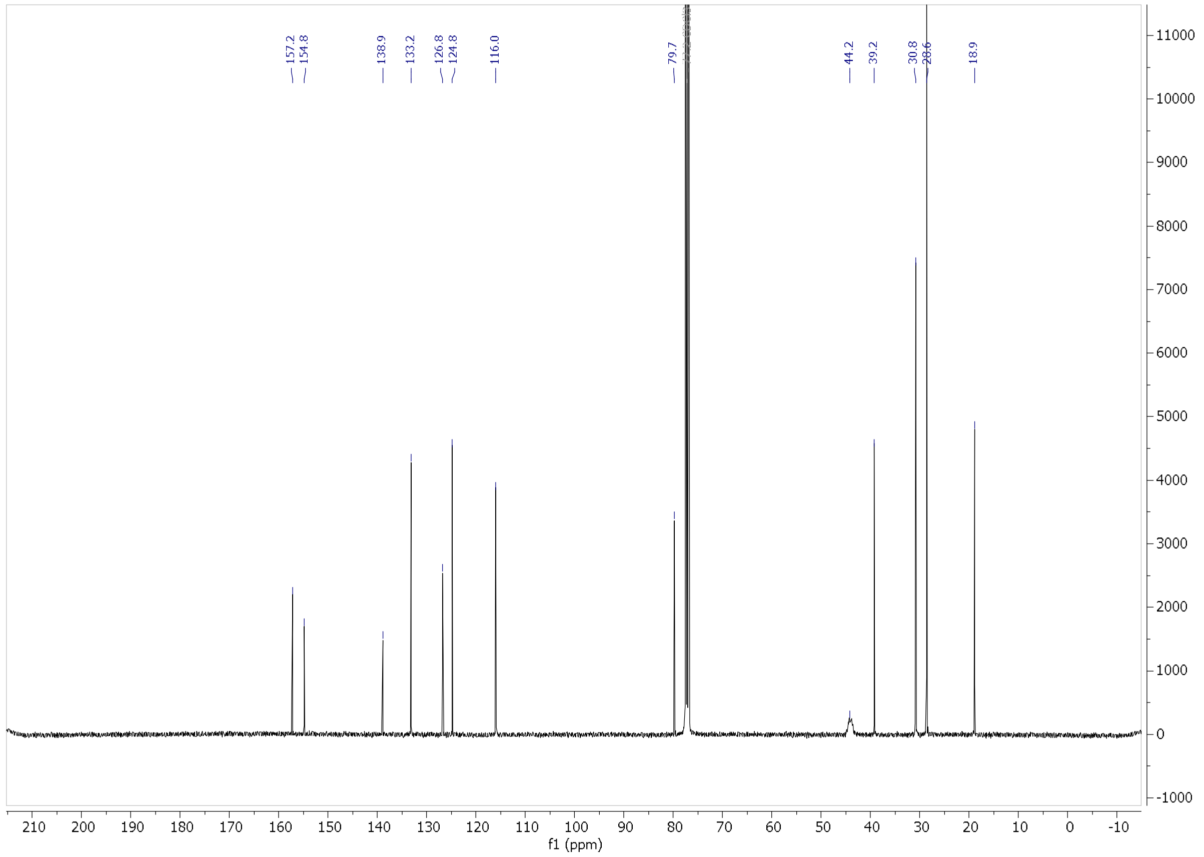

^{13}C NMR (101 MHz, $CDCl_3$) δ 157.2, 154.8, 138.9, 133.2, 126.8, 124.8, 116.0, 79.7, 44.2, 39.2 (2), 30.8 (2), 28.6 (3), 18.9

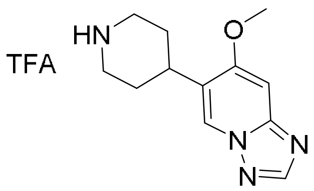

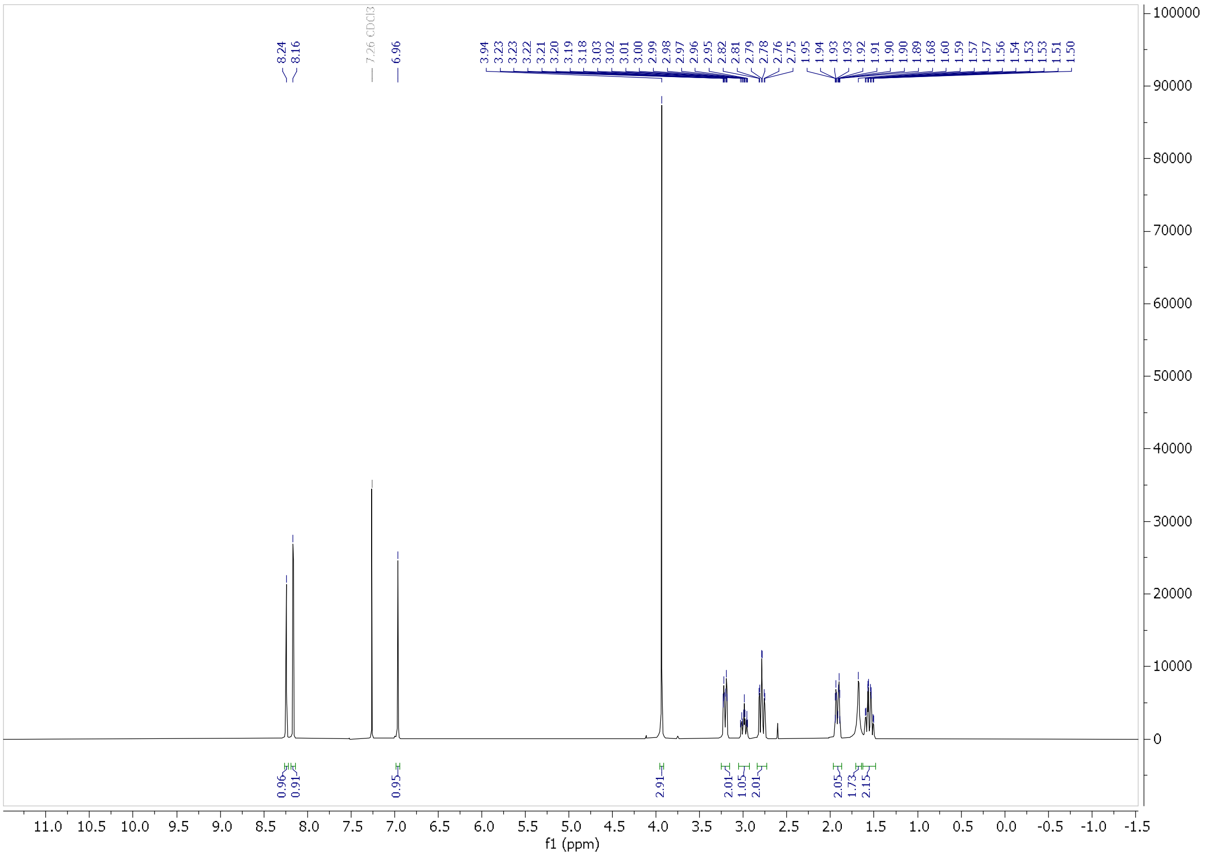

^{1}H NMR (400 MHz, $CDCl_3$) δ 8.24 (s, 1H), 8.16 (s, 1H), 6.96 (s, 1H), 3.94 (s, 3H), 3.21 (dt, *J* = 12.5, 2.9 Hz, 2H), 2.99 (tt, *J* = 12.1, 3.3 Hz, 1H), 2.79 (td, *J* = 12.2, 2.4 Hz, 2H), 1.92 (dp, *J* = 12.3, 2.4 Hz, 2H), 1.68 (s, 2H), 1.62 – 1.48 (m, 2H).

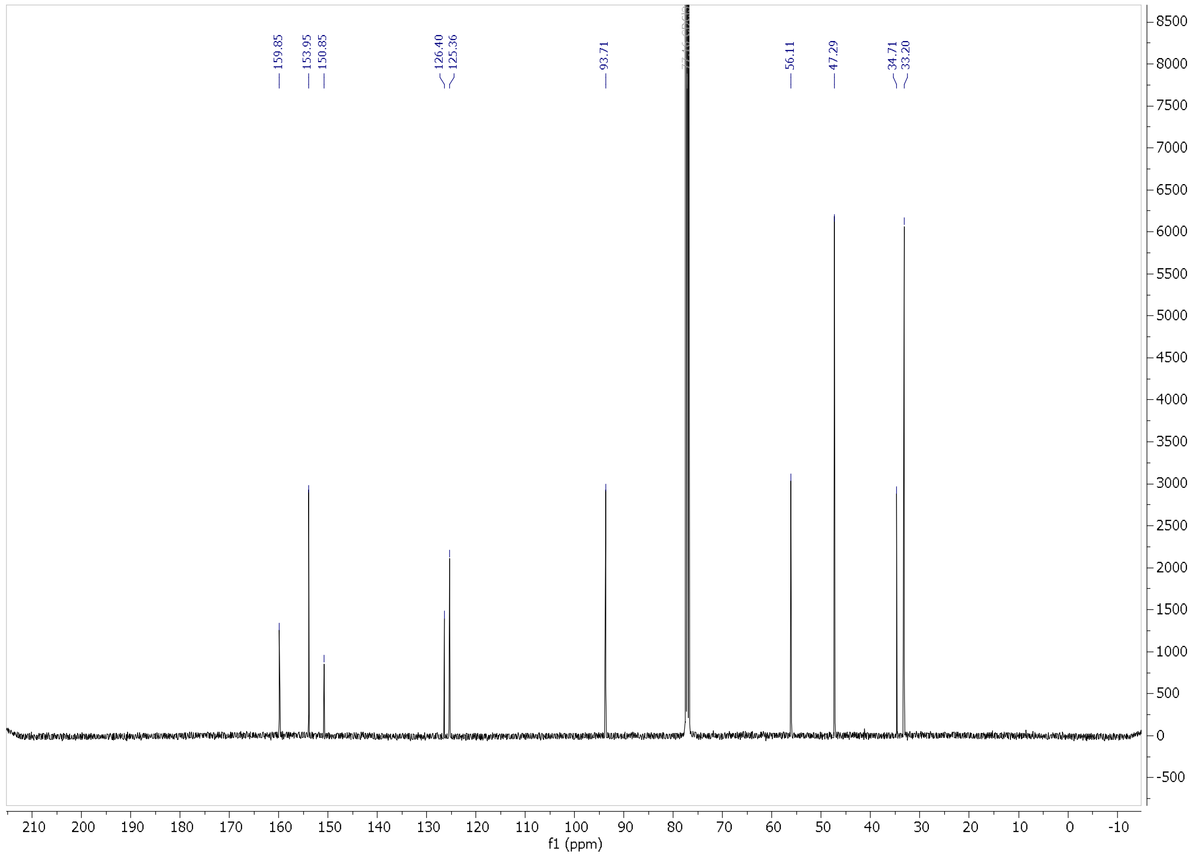

^{13}C NMR (101 MHz, $CDCl_3$) δ 159.9, 154.0, 150.9, 126.4, 125.4, 93.7, 56.1, 47.3 (2), 34.7, 33.2 (2)

Final Compound Characterizations – ^{1}H-NMR and/or ^{13}C-NMR Spectra

Compound 9

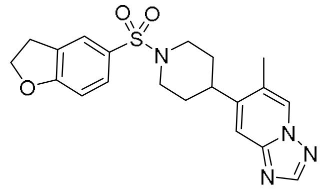

Chemical Formula: $C_{20}H_{22}N_4O_3S$

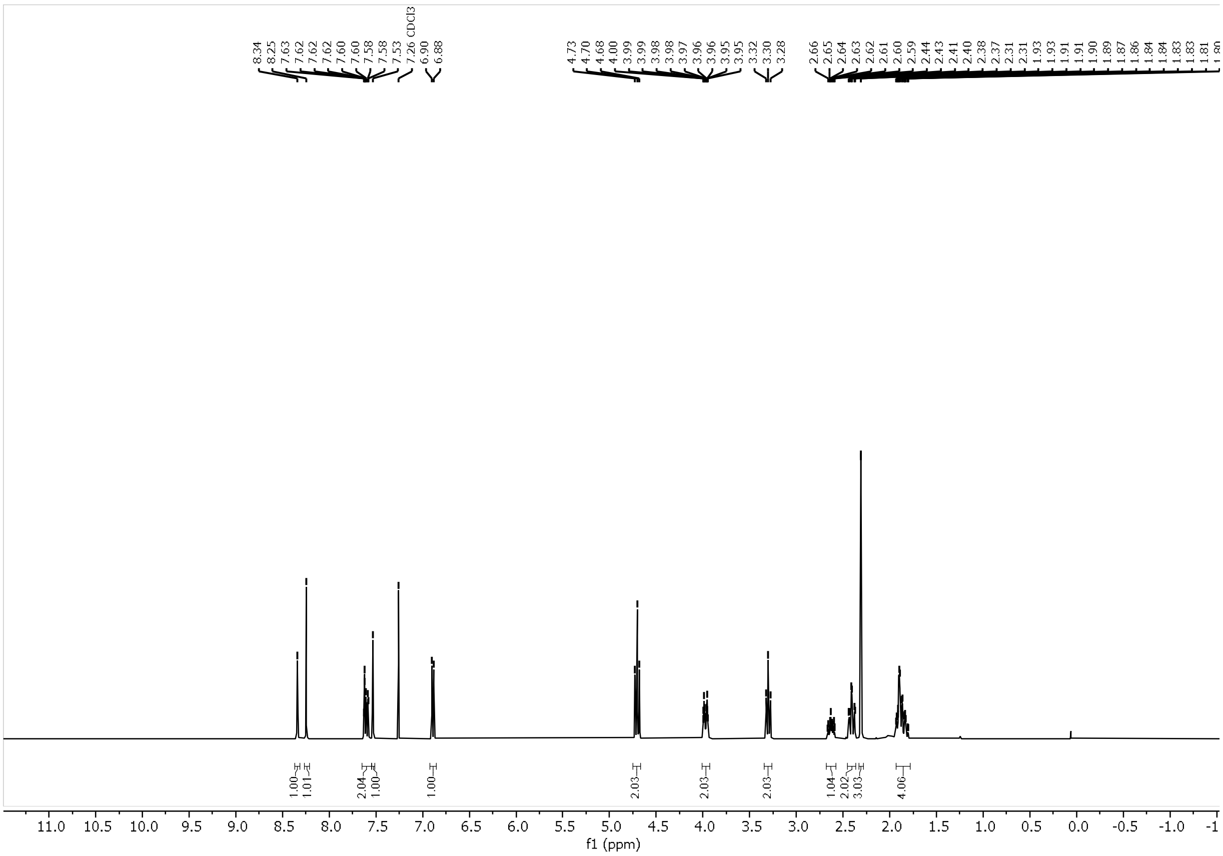

^{1}H NMR (400 MHz, $CDCl_3$) δ 8.34 (s, 1H), 8.25 (s, 1H), 7.65 – 7.55 (m, 2H), 7.53 (s, 1H), 6.89 (d, J = 8.3 Hz, 1H), 4.70 (t, J = 8.8 Hz, 2H), 4.01 – 3.93 (m, 2H), 3.30 (t, J = 8.8 Hz, 2H), 2.63 (tt, J = 11.3, 4.0 Hz, 1H), 2.41 (td, J = 11.7, 3.2 Hz, 2H), 2.31 (d, J = 1.0 Hz, 3H), 1.94 – 1.78 (m, 4H).

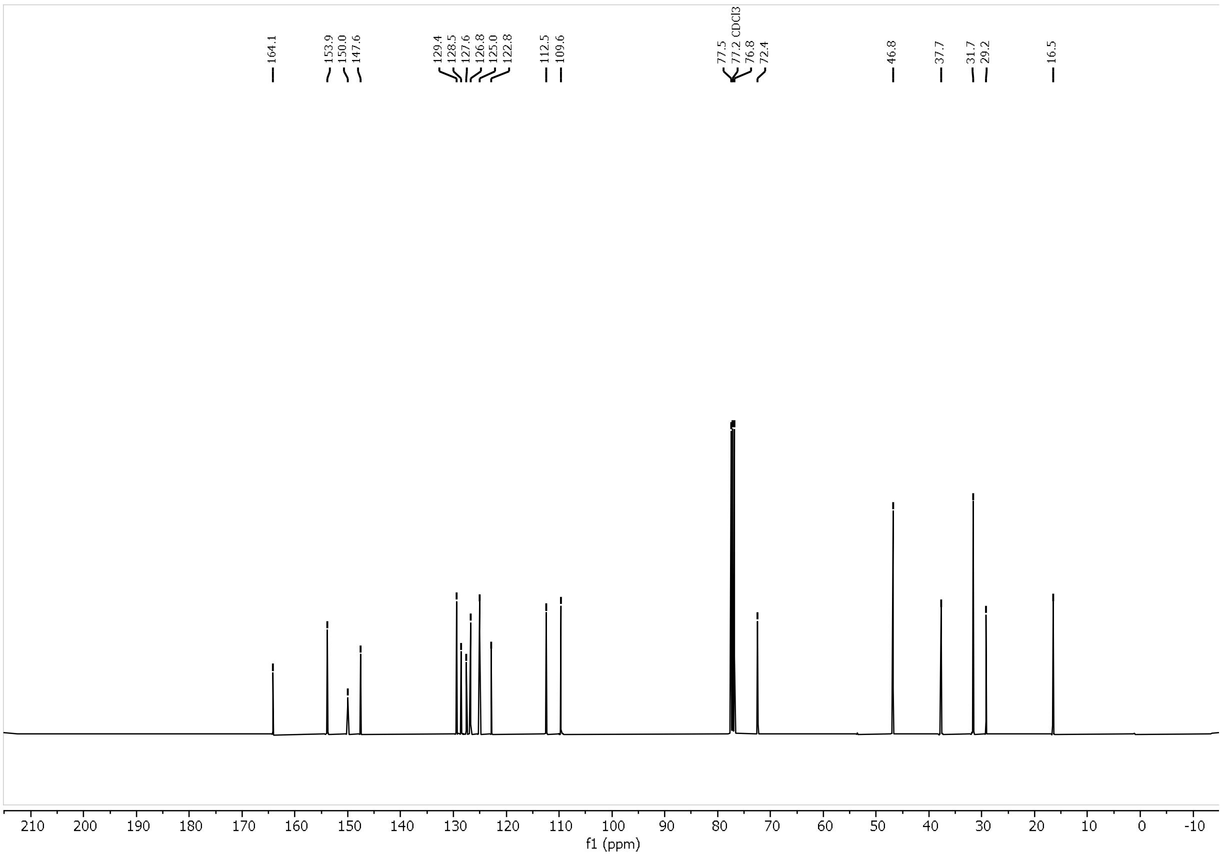

^{13}C NMR (101 MHz, $CDCl_3$) δ 164.1, 153.9, 150.0, 147.6, 129.4, 128.5, 127.6, 126.8, 125.0, 122.8, 112.5, 109.7, 72.4, 46.8 (2), 37.7, 31.7 (2), 29.2, 16.5.

Compound 10

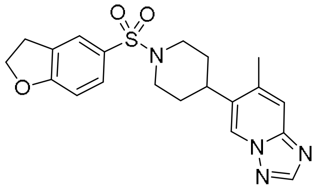

Chemical Formula: $C_{20}H_{22}N_4O_3S$

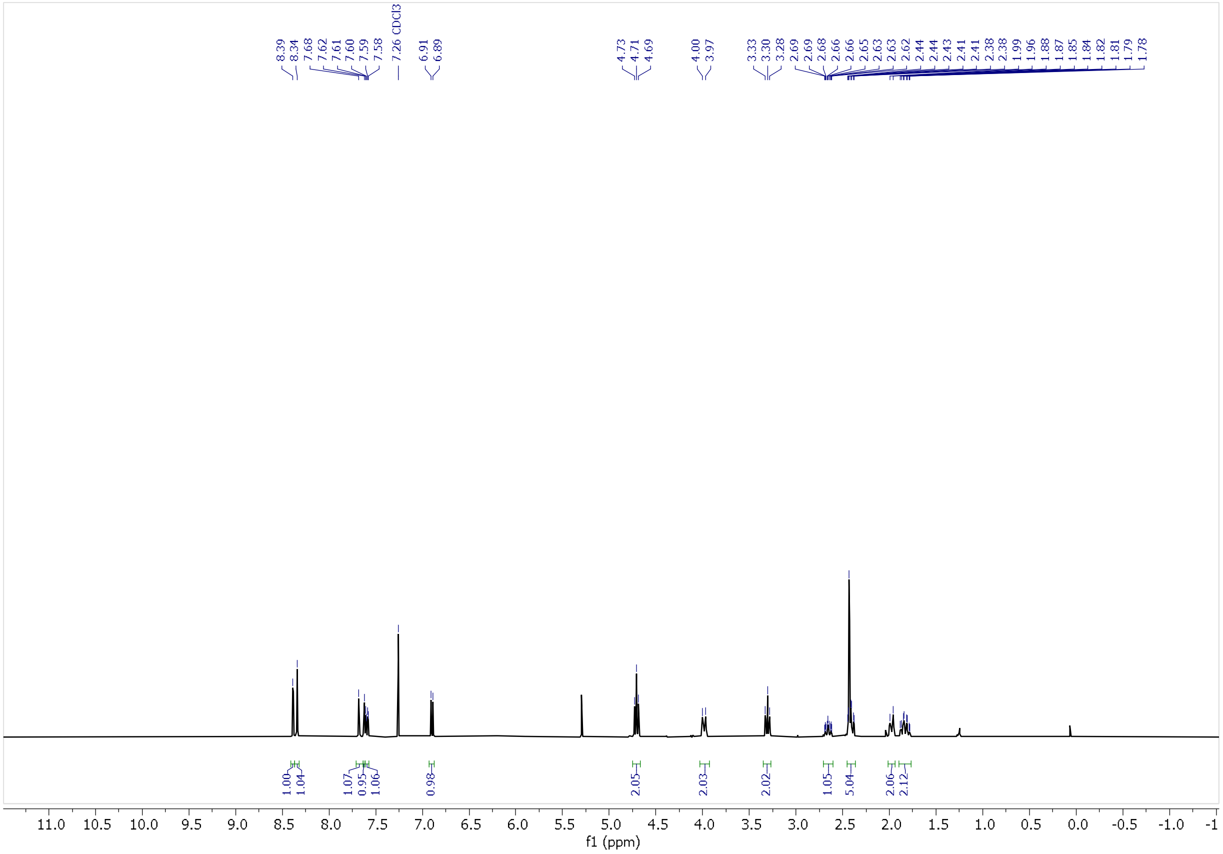

^{1}H NMR (400 MHz, $CDCl_3$) δ 8.39 (s, 1H), 8.34 (s, 1H), 7.68 (s, 1H), 7.62 (s, 1H), 7.59 (dd, J = 8.4, 2.1 Hz, 1H), 6.90 (d, J = 8.3 Hz, 1H), 4.71 (t, J = 8.9 Hz, 2H), 3.98 (d, J = 11.7 Hz, 2H), 3.30 (t, J = 8.8 Hz, 2H), 2.66 (tt, J = 12.1, 3.3 Hz, 1H), 2.45 – 2.37 (m, 2H), 2.43 (s, 3H) 1.97 (d, J = 13.8 Hz,, 2H), 1.83 (qd, J = 13.3, 12.7, 4.0 Hz, 2H).

Compound 11

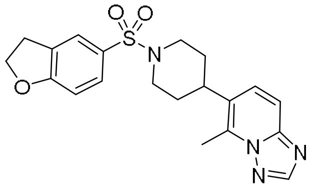

Chemical Formula: $C_{20}H_{22}N_4O_3S$

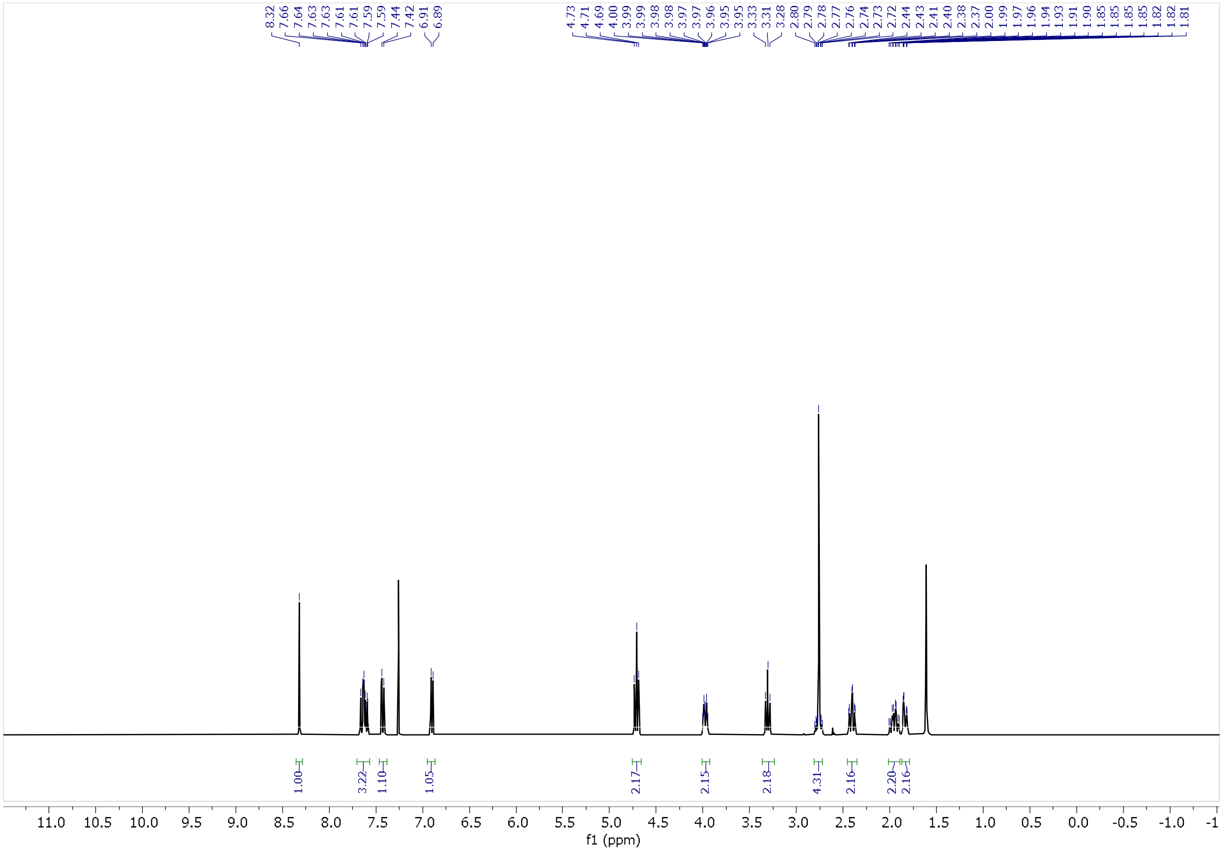

^{1}H NMR (400 MHz, $CDCl_3$) δ 8.32 (s, 1H), 7.70 – 7.57 (m, 3H), 7.43 (d, J = 9.3 Hz, 1H), 6.90 (d, J = 8.3 Hz, 1H), 4.71 (t, J = 8.8 Hz, 2H), 3.97 (dp, J = 11.4, 2.1 Hz, 2H), 3.31 (t, J = 8.8 Hz, 2H), 2.80 – 2.72 (m, 1H) 2.76 (s, 3H), 2.40 (td, J = 11.9, 2.7 Hz, 2H), 1.95 (qd, J = 12.9, 12.2, 4.3 Hz, 2H), 1.87 – 1.79 (m, 2H).

Compound 12

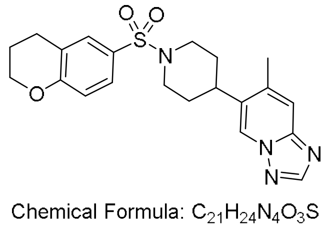

Chemical Formula: $C_{21}H_{24}N_4O_3S$

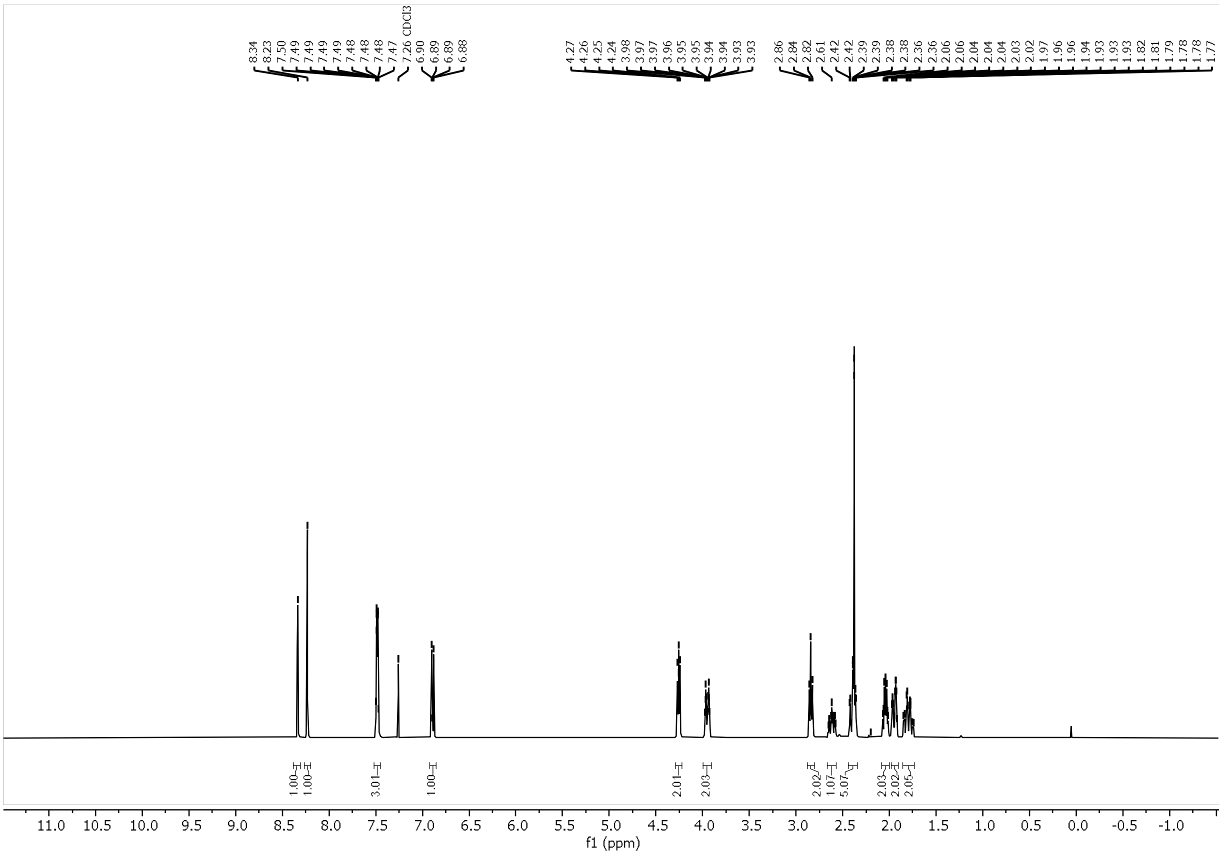

^{1}H NMR (400 MHz, $CDCl_3$) δ 8.34 (s, 1H), 8.23 (s, 1H), 7.52 – 7.45 (m, 3H), 6.92 – 6.86 (m, 1H), 4.29 – 4.22 (m, 2H), 4.00 – 3.90 (m, 2H), 2.84 (t, J = 6.4 Hz, 2H), 2.61 (tt, J = 12.1, 3.4 Hz, 1H), 2.44 – 2.34 (m, 5H), 2.09 – 2.00 (m, 2H), 1.98 – 1.91 (m, 2H), 1.86 – 1.73 (m, 2H).

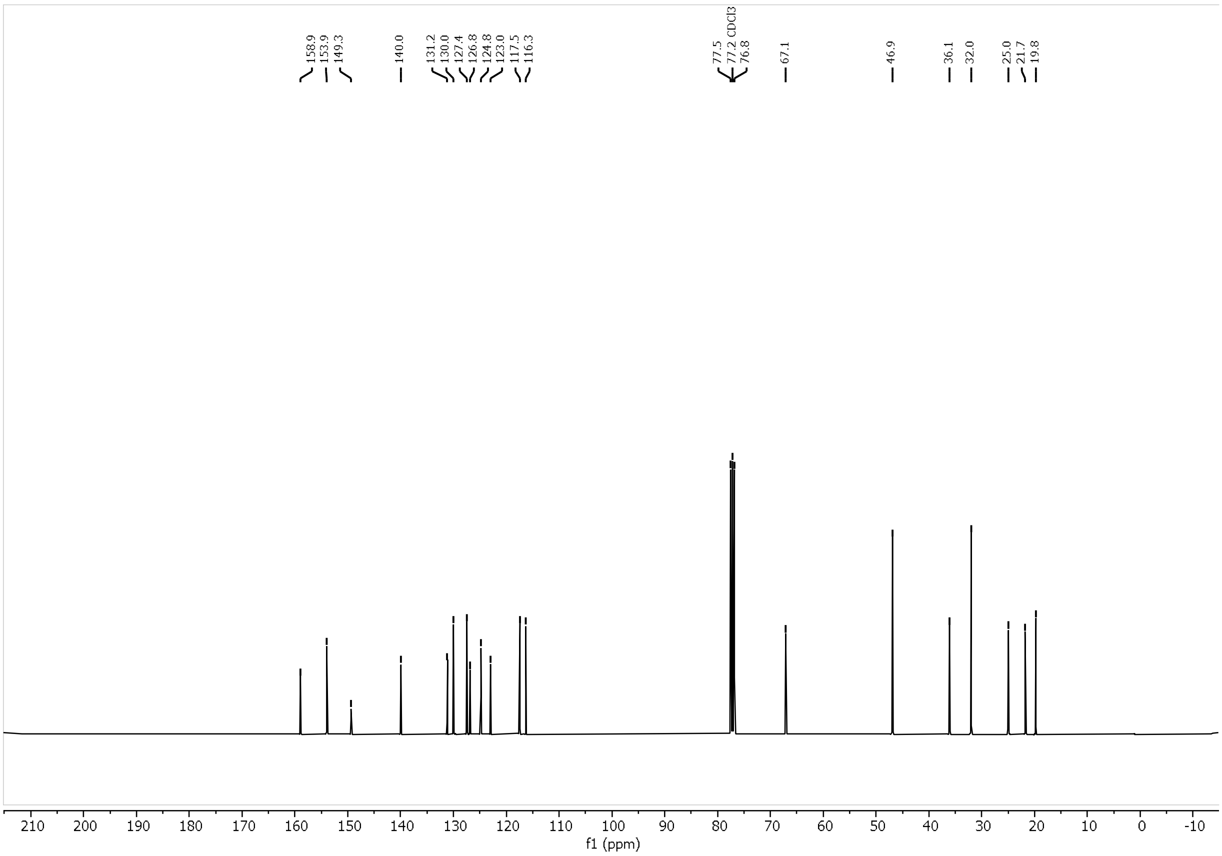

^{13}C NMR (101 MHz, $CDCl_3$) δ 158.9, 153.9, 149.3, 140.0, 131.2, 130.0, 127.4, 126.8, 124.8, 123.0, 117.5, 116.3, 67.1, 46.9 (2), 36.1, 32.0 (2), 25.0, 21.8, 19.8.

Compound 13

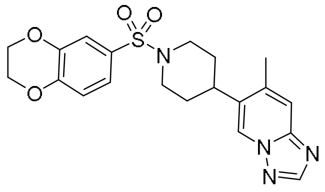

Chemical Formula: $C_{20}H_{22}N_4O_4S$

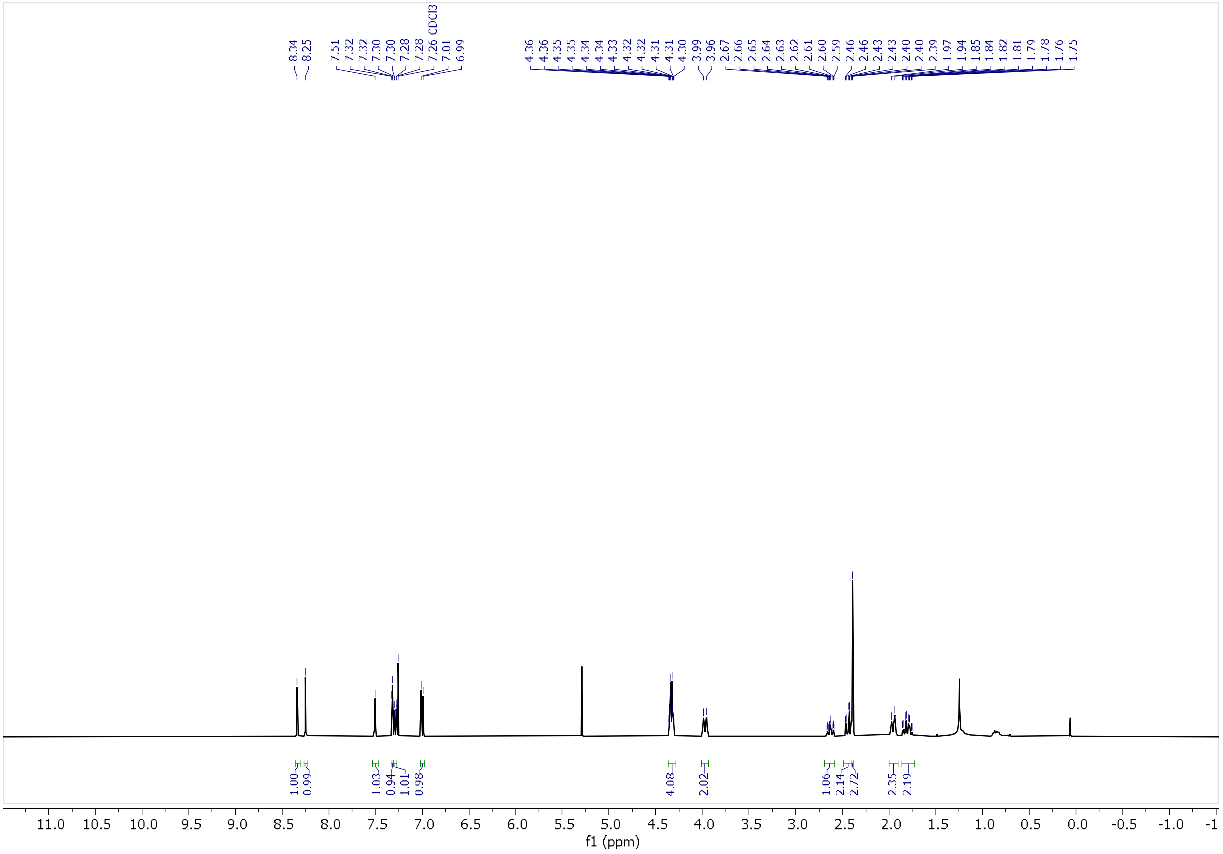

^{1}H NMR (400 MHz, $CDCl_3$) δ 8.34 (s, 1H), 8.25 (s, 1H), 7.51 (s, 1H), 7.32 (d, J = 2.1 Hz, 1H), 7.29 (dd, J = 8.4, 2.2 Hz, 1H), 7.00 (d, J = 8.5 Hz, 1H), 4.37 – 4.28 (m, 4H), 3.97 (d, J = 11.7 Hz, 2H), 2.63 (tt, J = 12.1, 3.3 Hz, 1H), 2.43 (td, J = 12.0, 2.5 Hz, 2H), 2.39 (s, 3H), 1.96 (d, J = 13.0 Hz, 2H), 1.80 (qd, J = 12.6, 3.9 Hz, 2H).

Compound 14

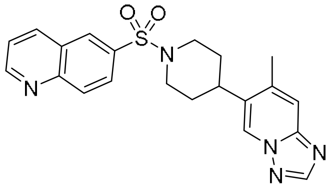

Chemical Formula: $C_{21}H_{21}N_5O_2S$

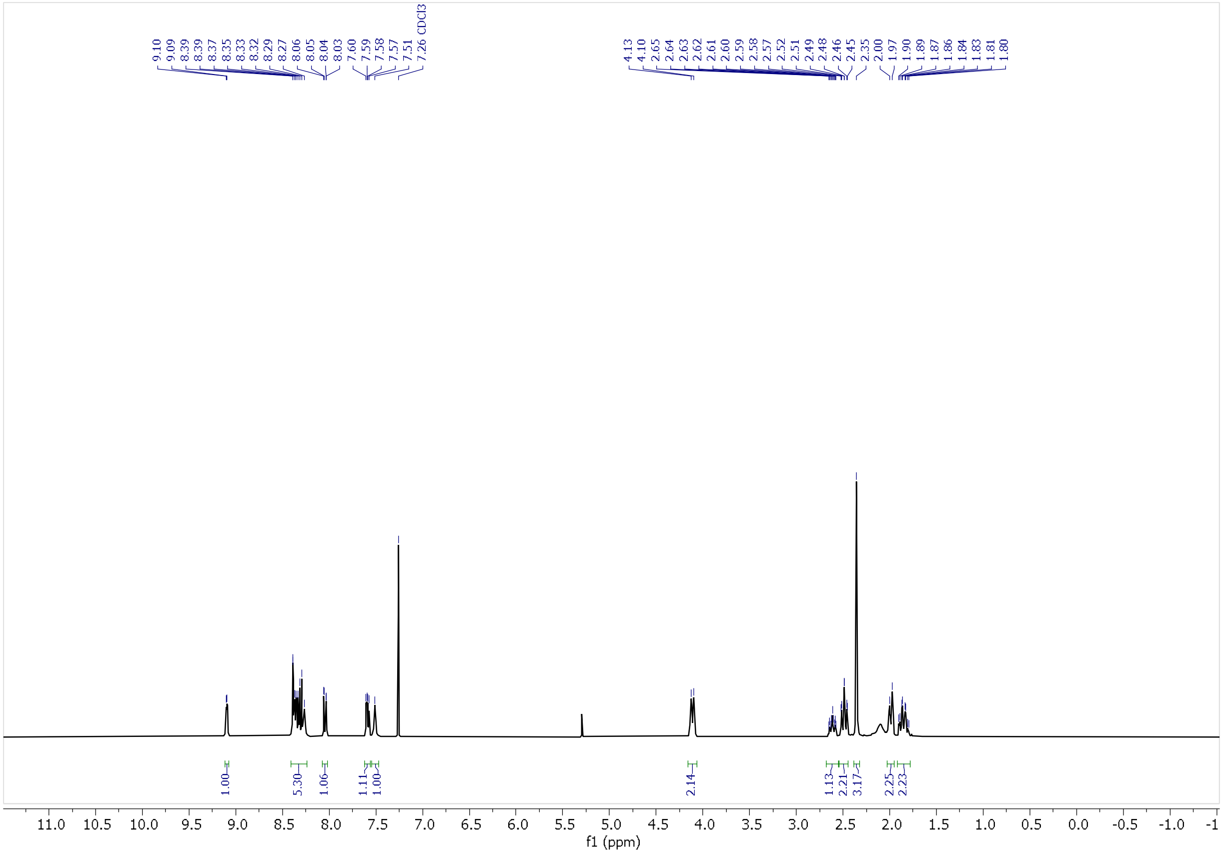

^{1}H NMR (400 MHz, $CDCl_3$) δ 9.10 (d, J = 2.5 Hz, 1H), 8.41 – 8.24 (m, 5H), 8.04 (dd, J = 8.8, 2.1 Hz, 1H), 7.59 (dd, J = 8.3, 4.3 Hz, 1H), 7.51 (s, 1H), 4.11 (d, J = 11.7 Hz, 2H), 2.61 (tt, J = 12.2, 3.3 Hz, 1H), 2.49 (td, J = 11.9, 1.9 Hz, 2H), 2.35 (s, 3H), 1.99 (d, J = 12.2 Hz, 2H), 1.85 (qd, J = 13.1, 12.6, 4.0 Hz, 2H).

Compound 15

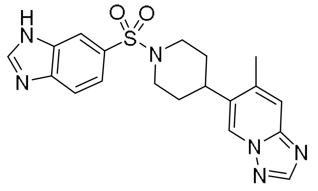

Chemical Formula: $C_{19}H_{20}N_6O_2S$

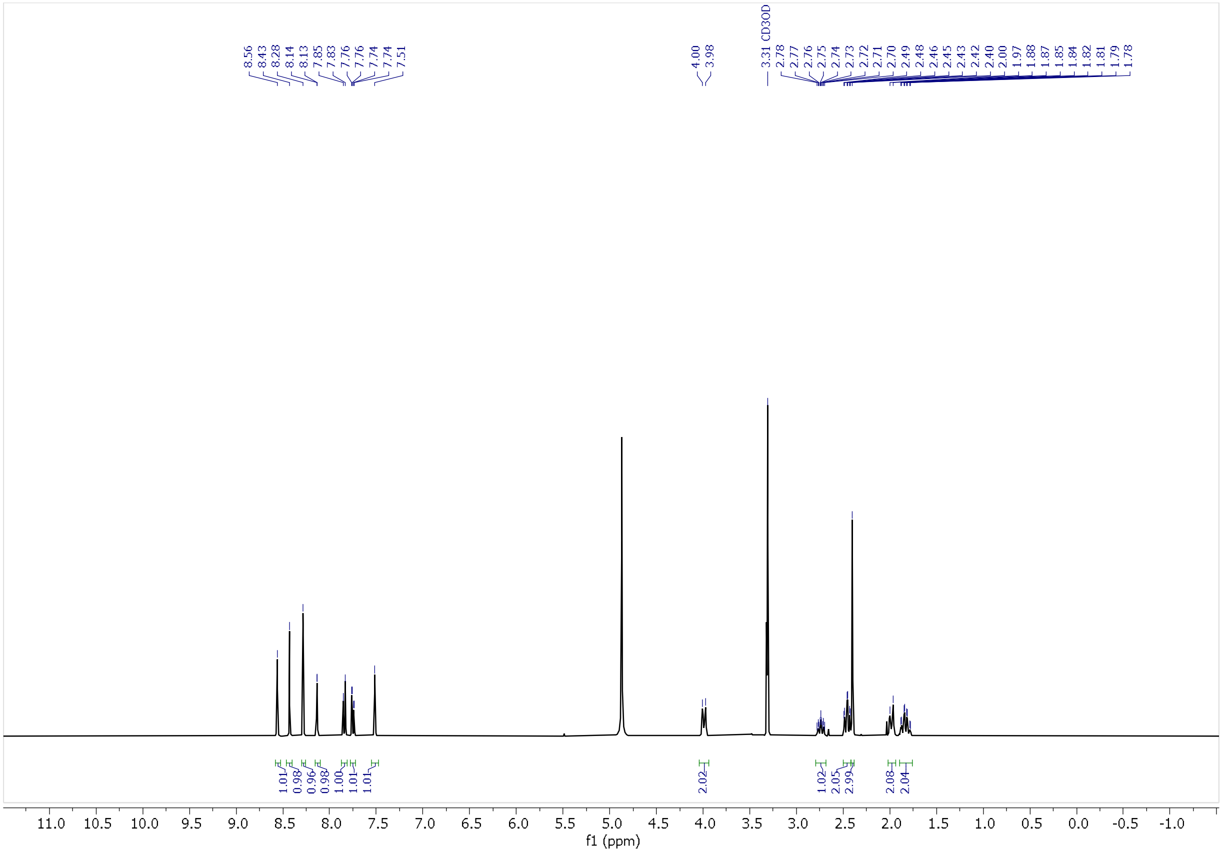

^{1}H NMR (400 MHz, MeOD) δ 8.56 (s, 1H), 8.43 (s, 1H), 8.28 (s, 1H), 8.13 (d, *J* = 1.2 Hz, 1H), 7.84 (d, *J* = 8.5 Hz, 1H), 7.75 (dd, *J* = 8.5, 1.7 Hz, 1H), 7.51 (s, 1H), 3.99 (d, *J* = 11.7 Hz, 2H), 2.74 (tt, *J* = 12.0, 3.2 Hz, 1H), 2.46 (td, *J* = 12.0, 2.5 Hz, 2H), 2.40 (s, 3H), 1.98 (d, *J* = 12.4 Hz, 2H), 1.83 (qd, *J* = 12.6, 3.9 Hz, 2H).

Compound 16

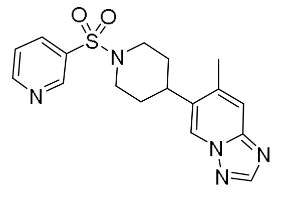

Chemical Formula: $C_{17}H_{19}N_5O_2S$

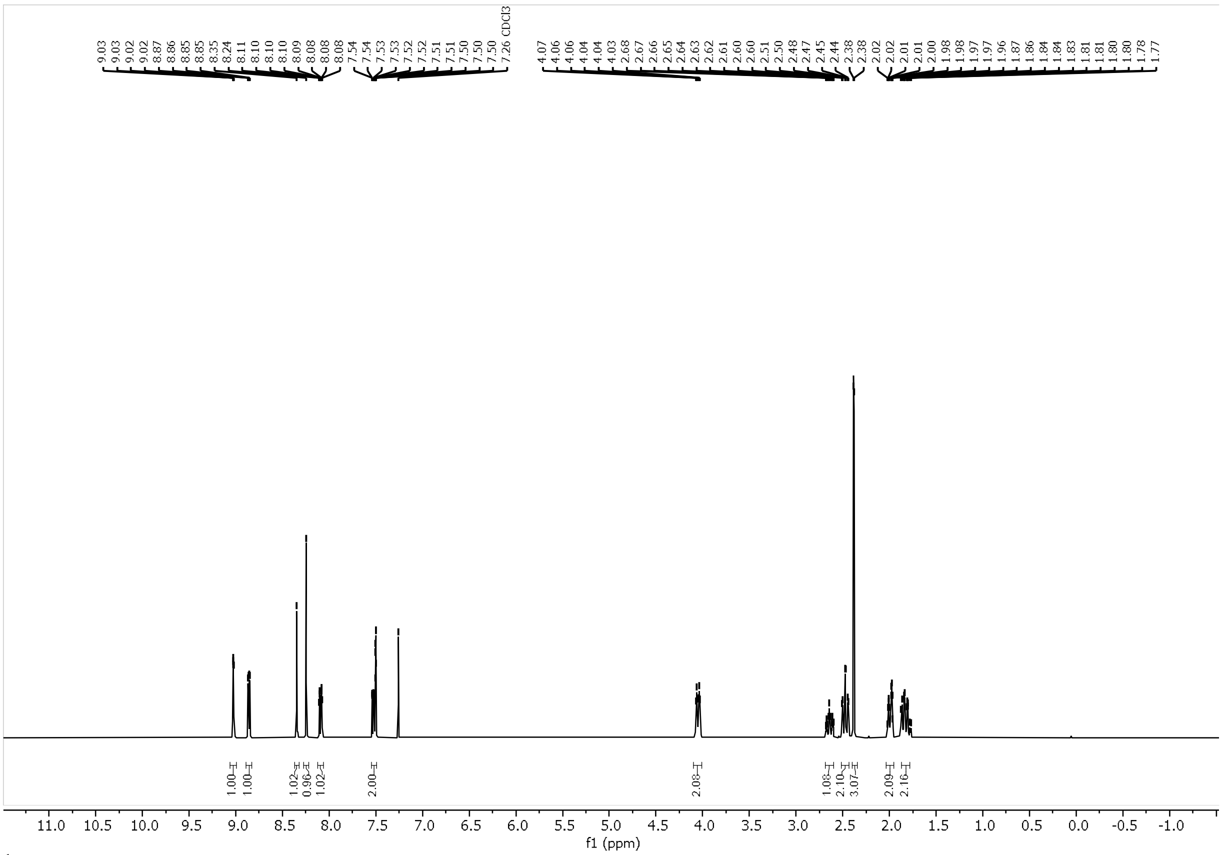

^{1}H NMR (400 MHz, $CDCl_3$) δ 9.03 (dd, J = 2.4, 0.8 Hz, 1H), 8.86 (dd, J = 4.9, 1.6 Hz, 1H), 8.35 (s, 1H), 8.24 (s, 1H), 8.09 (ddd, J = 8.0, 2.3, 1.6 Hz, 1H), 7.55 – 7.49 (m, 2H), 4.05 (dt, J = 11.6, 2.3 Hz, 2H), 2.69 – 2.60 (m, 1H), 2.47 (td, J = 12.1, 2.4 Hz, 2H), 2.38 (d, J = 1.0 Hz, 3H), 2.03 – 1.95 (m, 2H), 1.87 – 1.78 (m, 2H).

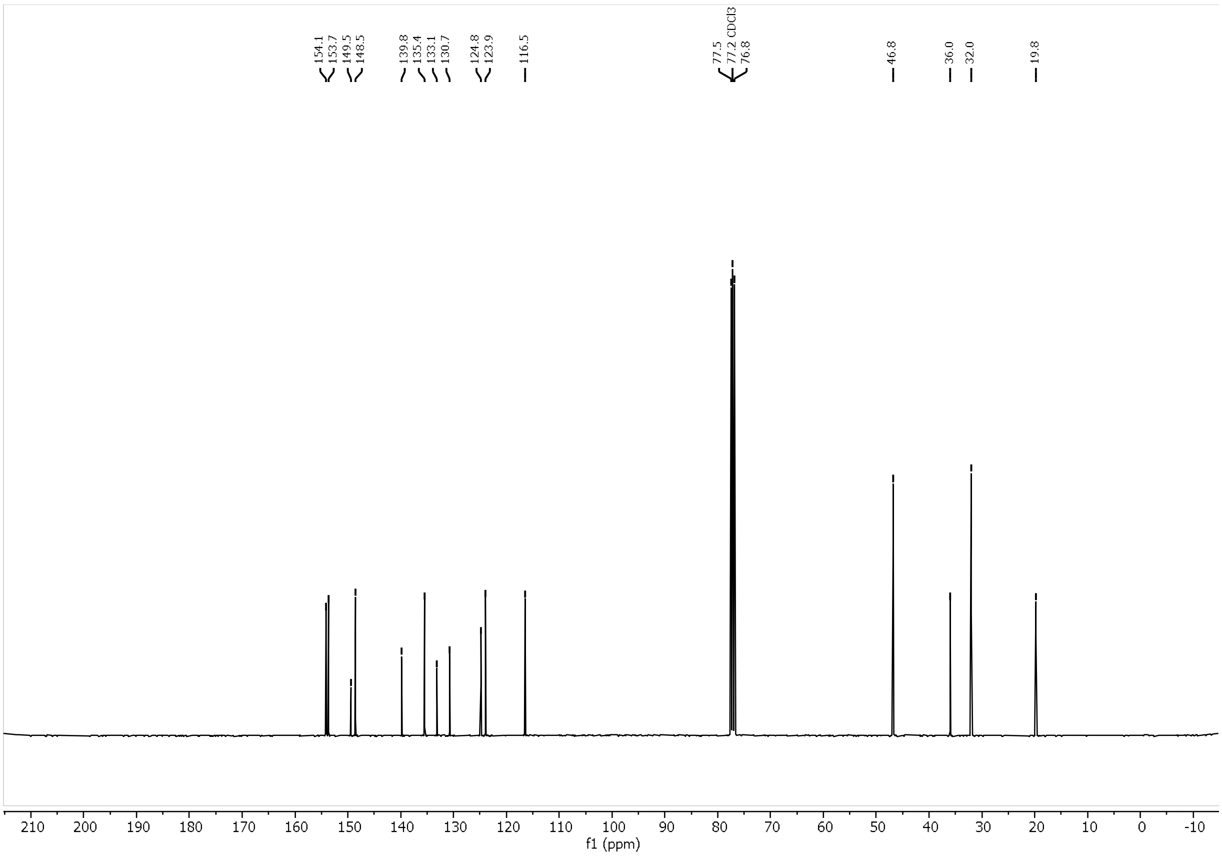

^{13}C NMR (101 MHz, $CDCl_3$) δ 154.1, 153.7, 149.5, 148.6, 139.8, 135.4, 133.1, 130.7, 124.8, 123.9, 116.5, 46.8 (2), 36.0, 32.0 (2), 19.8.

Compound 17

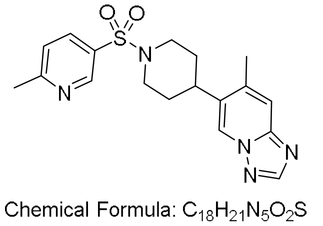

Chemical Formula: $C_{18}H_{21}N_5O_2S$

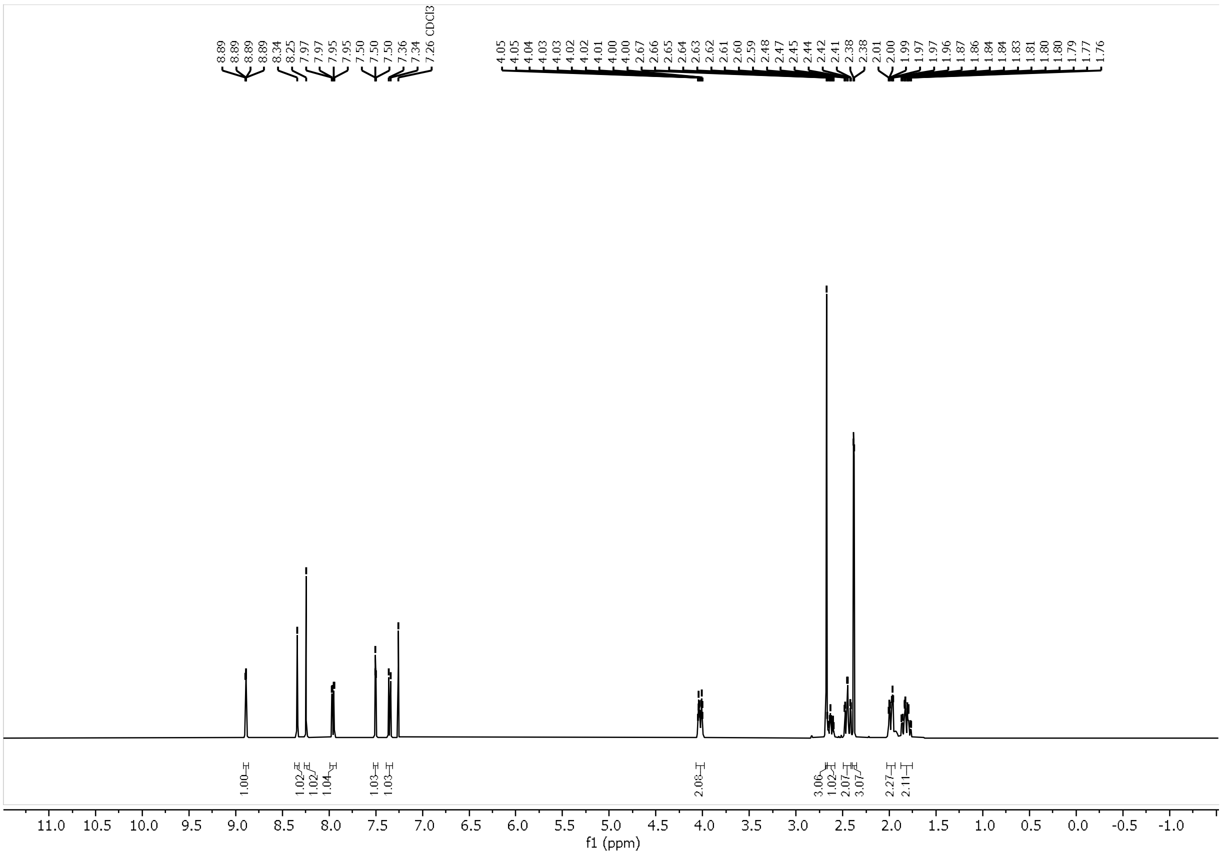

^{1}H NMR (400 MHz, $CDCl_3$) δ 8.89 (dd, J = 2.4, 0.8 Hz, 1H), 8.34 (s, 1H), 8.25 (s, 1H), 7.96 (dd, J = 8.1, 2.4 Hz, 1H), 7.50 (s, 1H), 7.35 (d, J = 8.1 Hz, 1H), 4.07 – 3.98 (m, 2H), 2.67 (s, 3H), 2.62 (dt, J = 12.1, 3.3 Hz, 1H), 2.45 (td, J = 12.0, 2.4 Hz, 2H), 2.38 (d, J = 1.0 Hz, 3H), 2.02 - 1.93 (m, 2H), 1.87 – 1.75 (m, 2H).

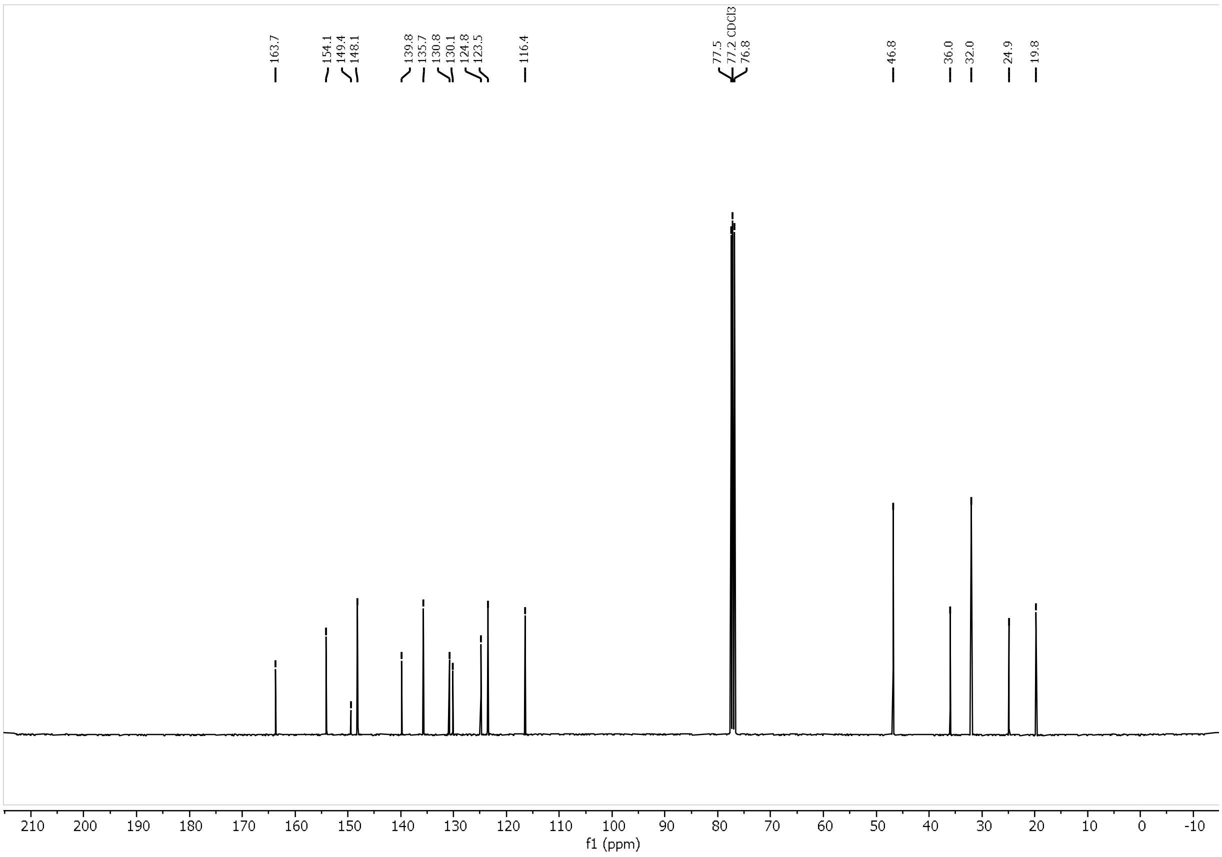

^{13}C NMR (101 MHz, $CDCl_3$) δ 163.7, 154.1, 149.4, 148.1, 139.8, 135.7, 130.8, 130.1, 124.8, 123.5, 116.4, 46.8 (2), 36.0, 32.0 (2), 24.9, 19.8.

Compound 18

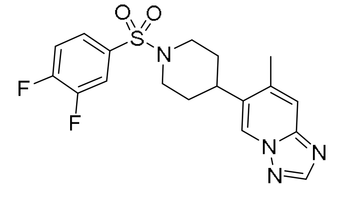

Chemical Formula: $C_{18}H_{18}F_2N_4O_2S$

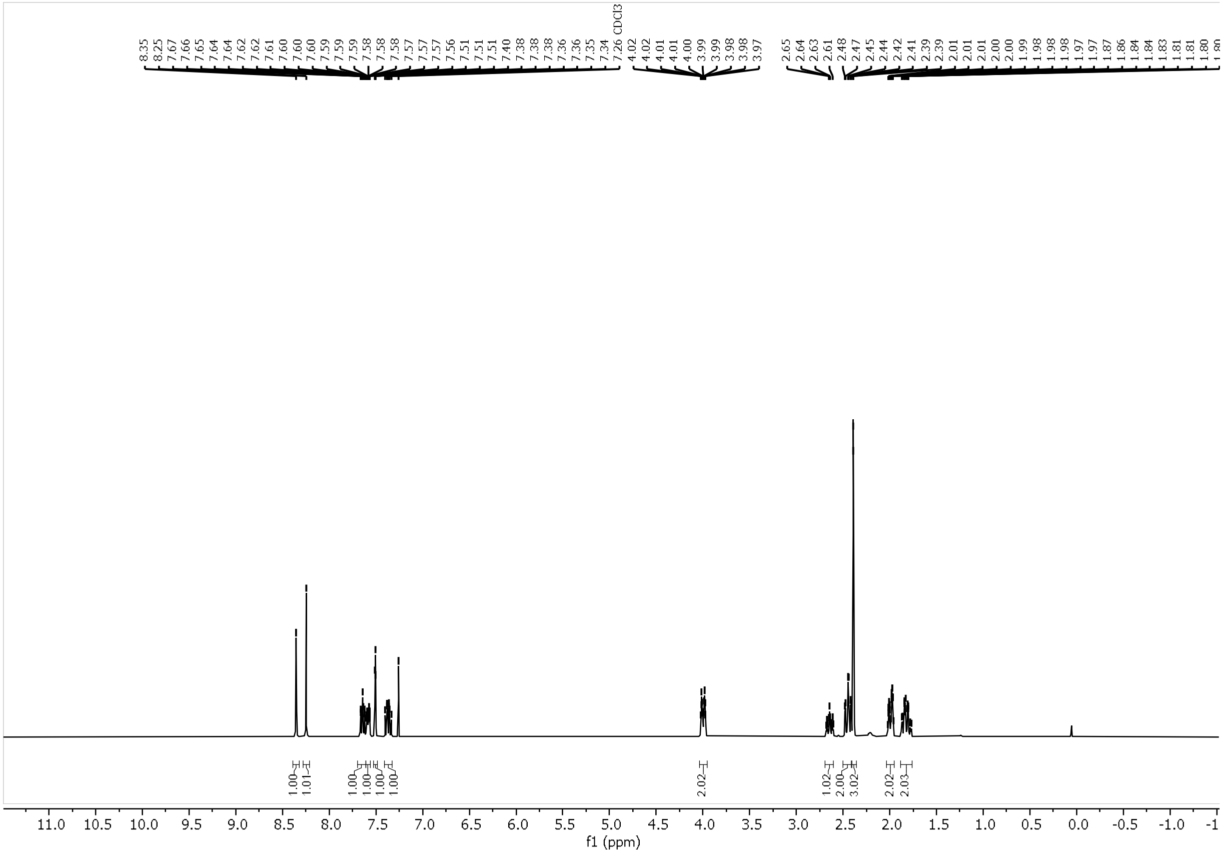

^{1}H NMR (400 MHz, $CDCl_3$) δ 8.35 (s, 1H), 8.25 (s, 1H), 7.64 (ddd, J = 9.3, 7.2, 2.2 Hz, 1H), 7.58 (dddd, J = 8.6, 3.9, 2.2, 1.4 Hz, 1H), 7.51 (s, 1H), 7.37 (ddd, J = 9.6, 8.6, 7.3 Hz, 1H), 4.04 – 3.95 (m, 2H), 2.64 (tt, J = 12.1, 3.3 Hz, 1H), 2.44 (td, J = 12.0, 2.4 Hz, 2H), 2.39 (d, J = 0.9 Hz, 3H), 2.03 – 1.95 (m, 2H), 1.88 – 1.76 (m, 2H).

Compound 19

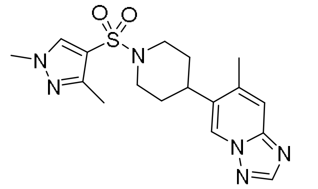

Chemical Formula: $C_{17}H_{22}N_6O_2S$

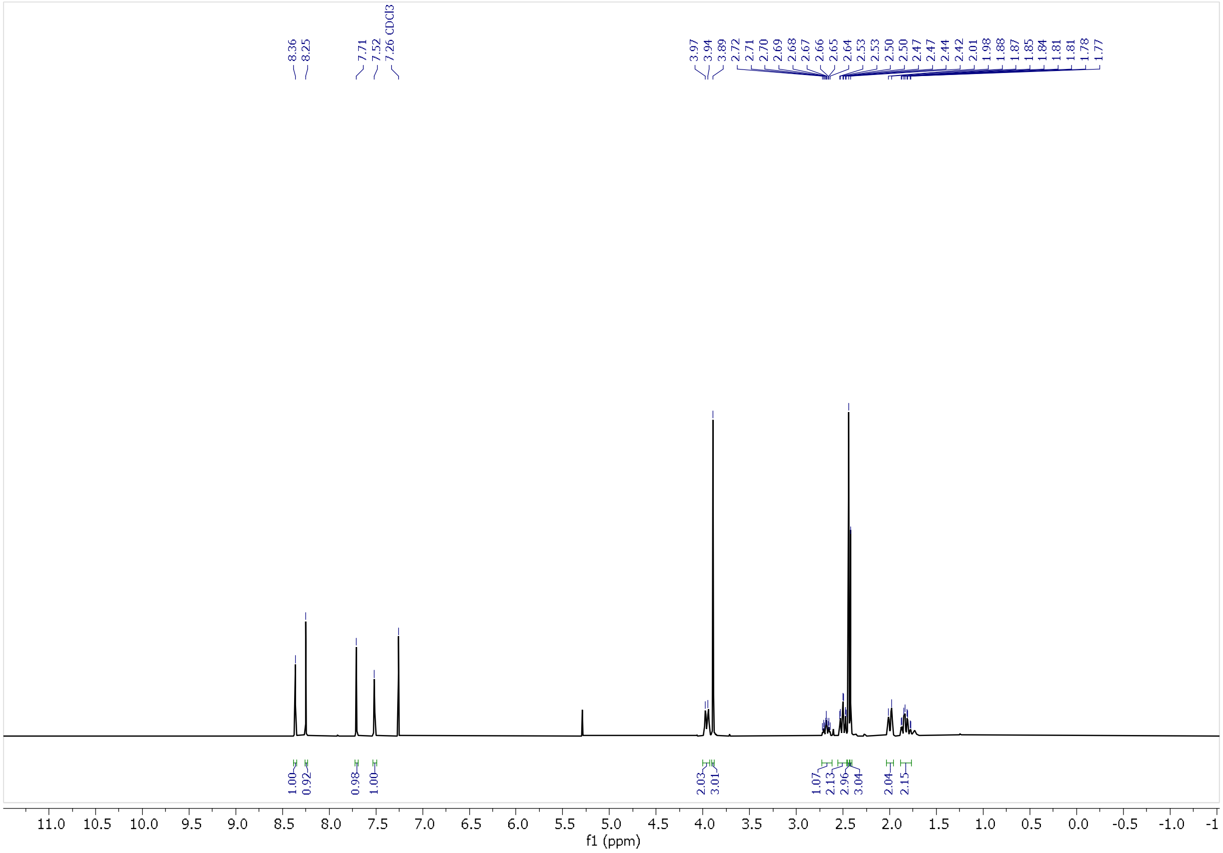

^{1}H NMR (400 MHz, $CDCl_3$) δ 8.36 (s, 1H), 8.25 (s, 1H), 7.71 (s, 1H), 7.52 (s, 1H), 3.96 (d, J = 11.5 Hz, 2H), 3.89 (s, 3H), 2.68 (tt, J = 12.1, 3.3 Hz, 1H), 2.50 (td, J = 12.0, 2.5 Hz, 2H), 2.44 (s, 3H), 2.42 (s, 3H), 2.00 (d, J = 14.1 Hz, 2H), 1.83 (qd, J = 13.4, 12.7, 4.0 Hz, 2H).

Compound 20

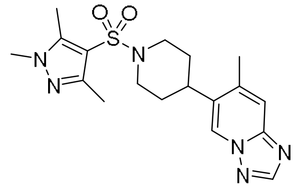

Chemical Formula: $C_{18}H_{24}N_6O_2S$

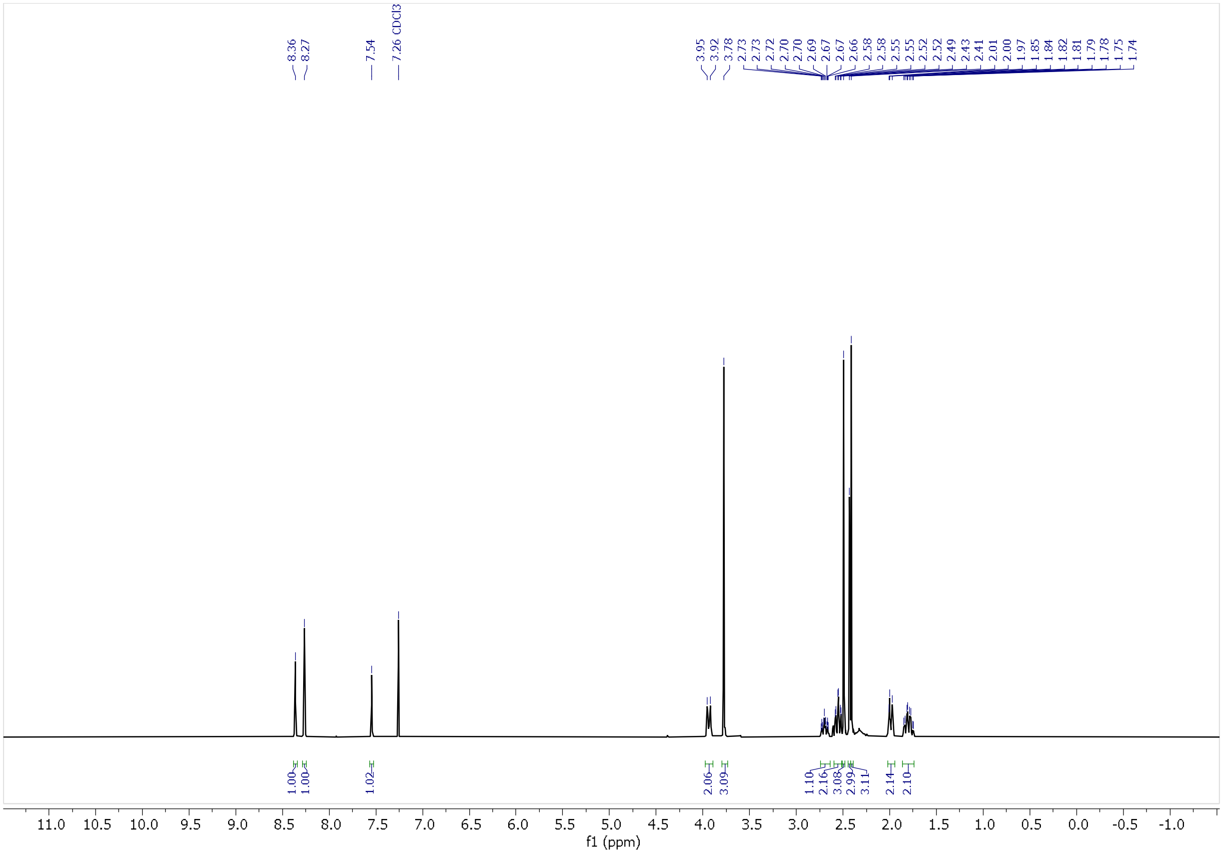

^{1}H NMR (400 MHz, $CDCl_3$) δ 8.36 (s, 1H), 8.27 (s, 1H), 7.54 (s, 1H), 3.94 (d, J = 11.5 Hz, 2H), 3.78 (s, 3H), 2.70 (tt, J = 12.1, 3.3 Hz, 1H), 2.55 (td, J = 12.0, 2.5 Hz, 2H), 2.49 (s, 3H), 2.43 (s, 3H), 2.41 (s, 3H), 2.02 – 1.95 (m, 2H), 1.80 (qd, J = 12.8, 3.9 Hz, 2H).

Compound 21

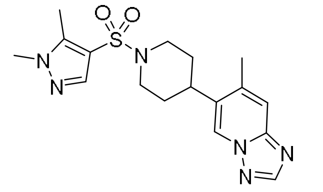

Chemical Formula: $C_{17}H_{22}N_6O_2S$

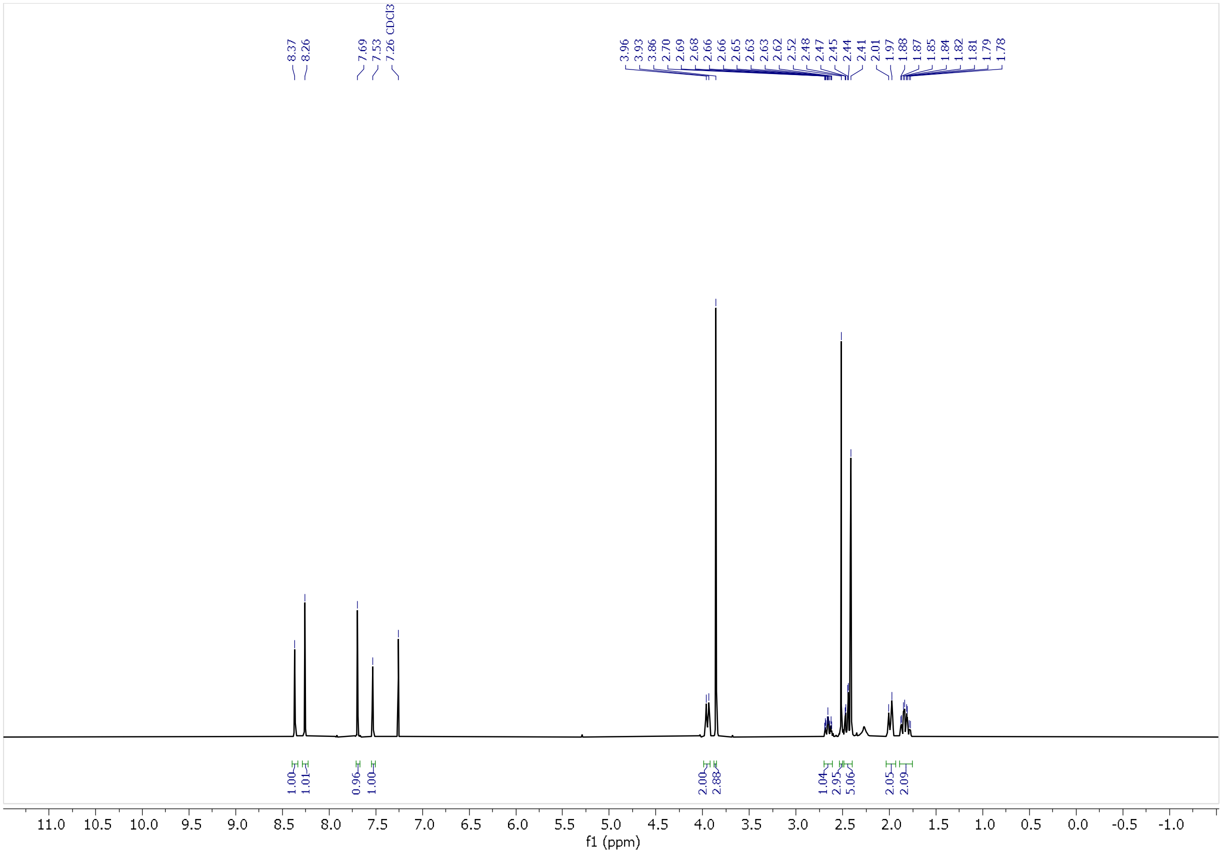

^{1}H NMR (400 MHz, $CDCl_3$) δ 8.37 (s, 1H), 8.26 (s, 1H), 7.69 (s, 1H), 7.53 (s, 1H), 3.95 (d, *J* = 11.4 Hz, 2H), 3.86 (s, 3H), 2.66 (tt, *J* = 12.0, 3.3 Hz, 1H), 2.52 (s, 3H), 2.49 – 2.40 (m, 5H), 1.99 (d, *J* = 14.1 Hz, 2H), 1.83 (qd, *J* = 12.7, 3.9 Hz, 2H).

Compound 22

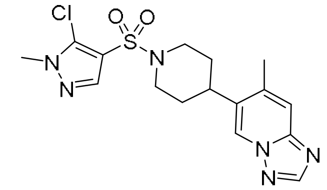

Chemical Formula: $C_{16}H_{19}ClN_6O_2S$

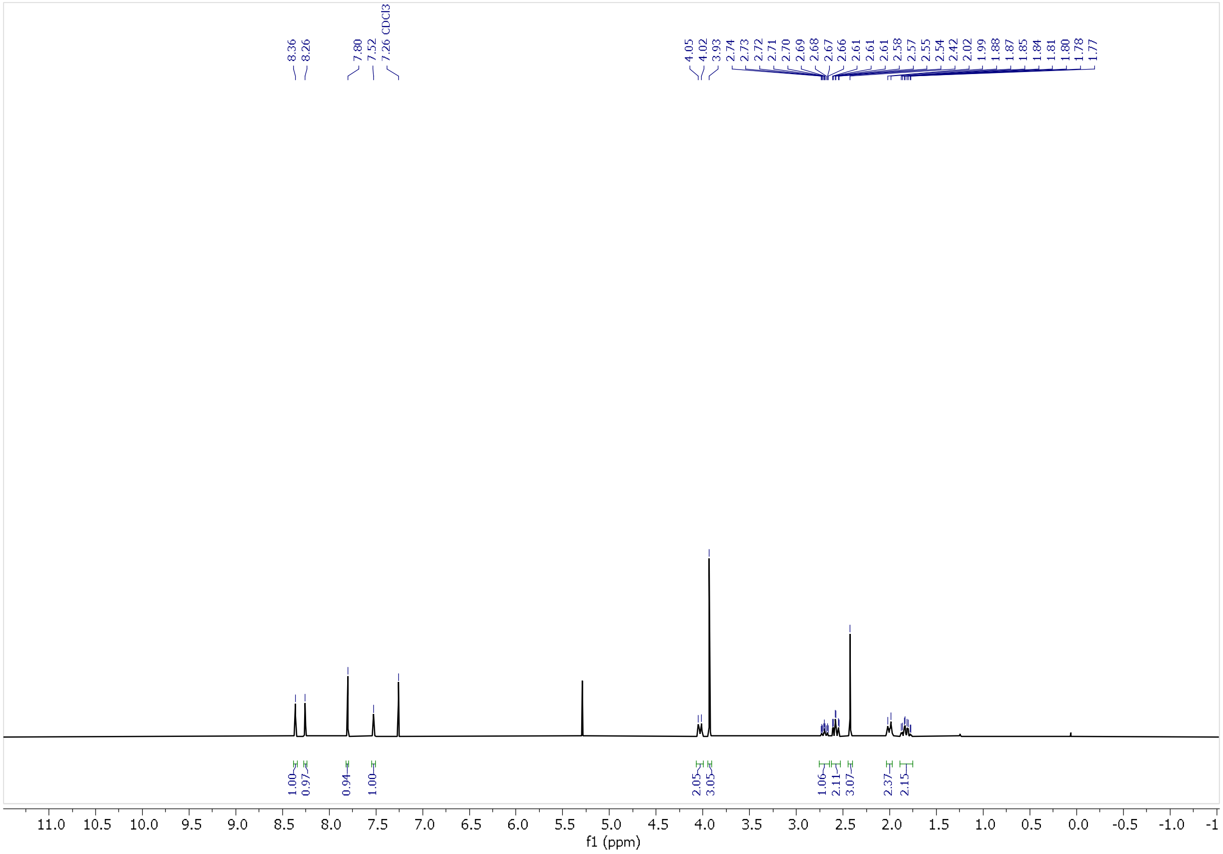

^{1}H NMR (400 MHz, $CDCl_3$) δ 8.36 (s, 1H), 8.26 (s, 1H), 7.80 (s, 1H), 7.52 (s, 1H), 4.03 (d, J = 11.8 Hz, 2H), 3.93 (s, 3H), 2.70 (tt, J = 12.2, 3.3 Hz, 1H), 2.58 (td, J = 12.1, 11.4, 1.8 Hz, 2H), 2.42 (s, 3H), 2.00 (d, J = 13.4 Hz, 2H), 1.82 (qd, J = 12.6, 4.0 Hz, 2H).

Compound 23

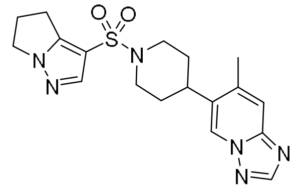

Chemical Formula: $C_{18}H_{22}N_6O_2S$

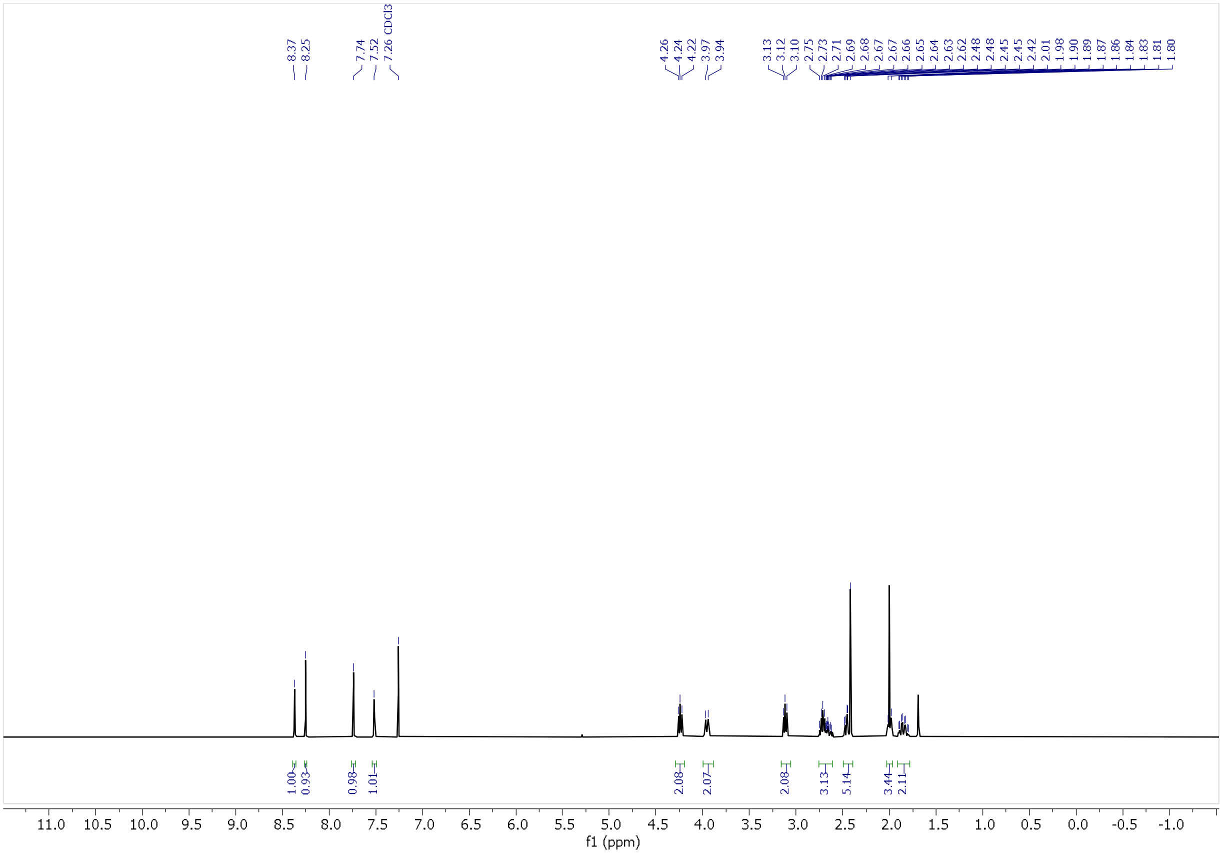

^{1}H NMR (400 MHz, $CDCl_3$) δ 8.37 (s, 1H), 8.25 (s, 1H), 7.74 (s, 1H), 7.52 (s, 1H), 4.24 (t, *J* = 7.4 Hz, 2H), 3.96 (d, *J* = 11.4 Hz, 2H), 3.12 (t, *J* = 7.5 Hz, 2H), 2.75 – 2.61 (m, 3H), 2.49 – 2.39 (m, 5H), 2.00 (d, *J* = 11.6 Hz, 2H), 1.85 (qd, *J* = 13.3, 12.7, 4.0 Hz, 2H). *Acetone peak at 2.0 ppm.

Compound 24

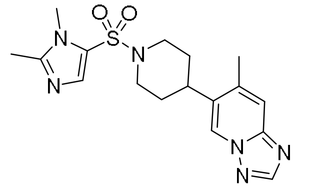

Chemical Formula: $C_{17}H_{22}N_6O_2S$

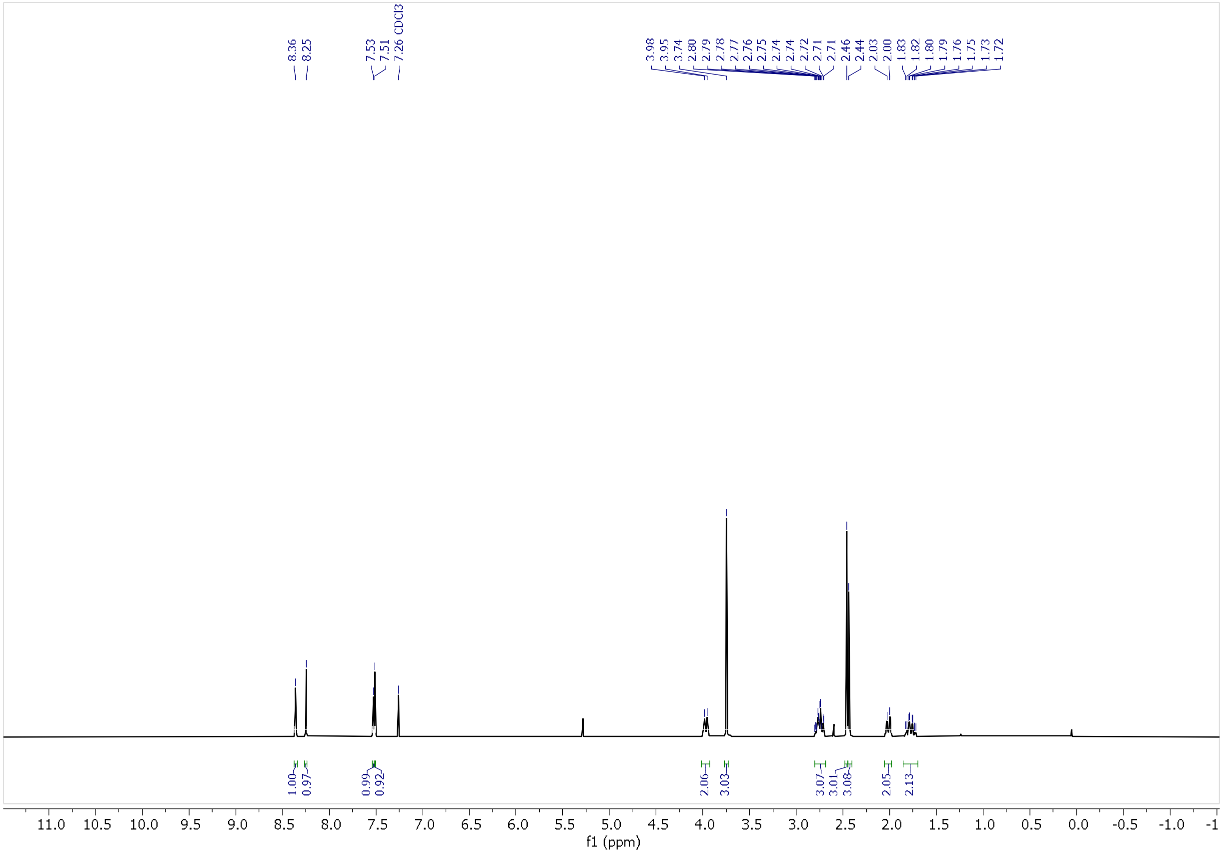

^{1}H NMR (400 MHz, $CDCl_3$) δ 8.36 (s, 1H), 8.25 (s, 1H), 7.53 (s, 1H), 7.51 (s, 1H), 3.97 (d, J = 12.0 Hz, 2H), 3.74 (s, 3H), 2.80 – 2.69 (m, 3H), 2.46 (s, 3H), 2.44 (s, 3H), 2.01 (d, J = 12.7 Hz, 2H), 1.77 (qd, J = 13.4, 12.8, 4.1 Hz, 2H).

Compound 25

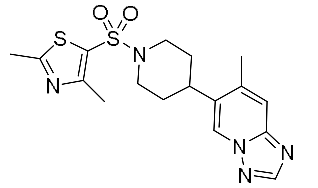

Chemical Formula: $C_{17}H_{21}N_5O_2S_2$

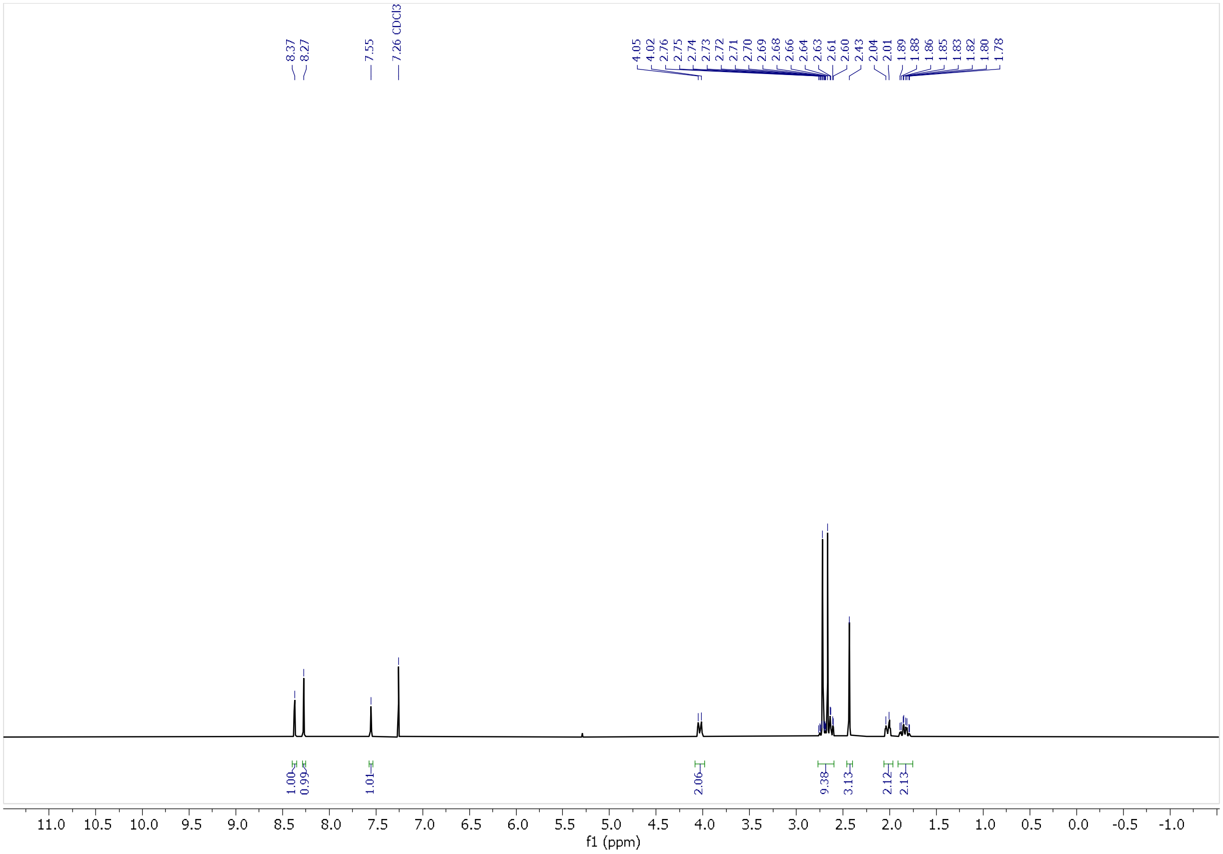

^{1}H NMR (400 MHz, $CDCl_3$) δ 8.37 (s, 1H), 8.27 (s, 1H), 7.55 (s, 1H), 4.03 (d, J = 11.6 Hz, 2H), 2.77 – 2.60 (m, 9H), 2.43 (s, 3H), 2.02 (d, J = 13.1 Hz, 2H), 1.84 (qd, J = 12.8, 4.1 Hz, 2H).

Compound 26

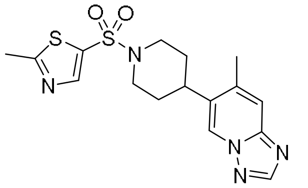

Chemical Formula: $C_{16}H_{19}N_5O_2S_2$

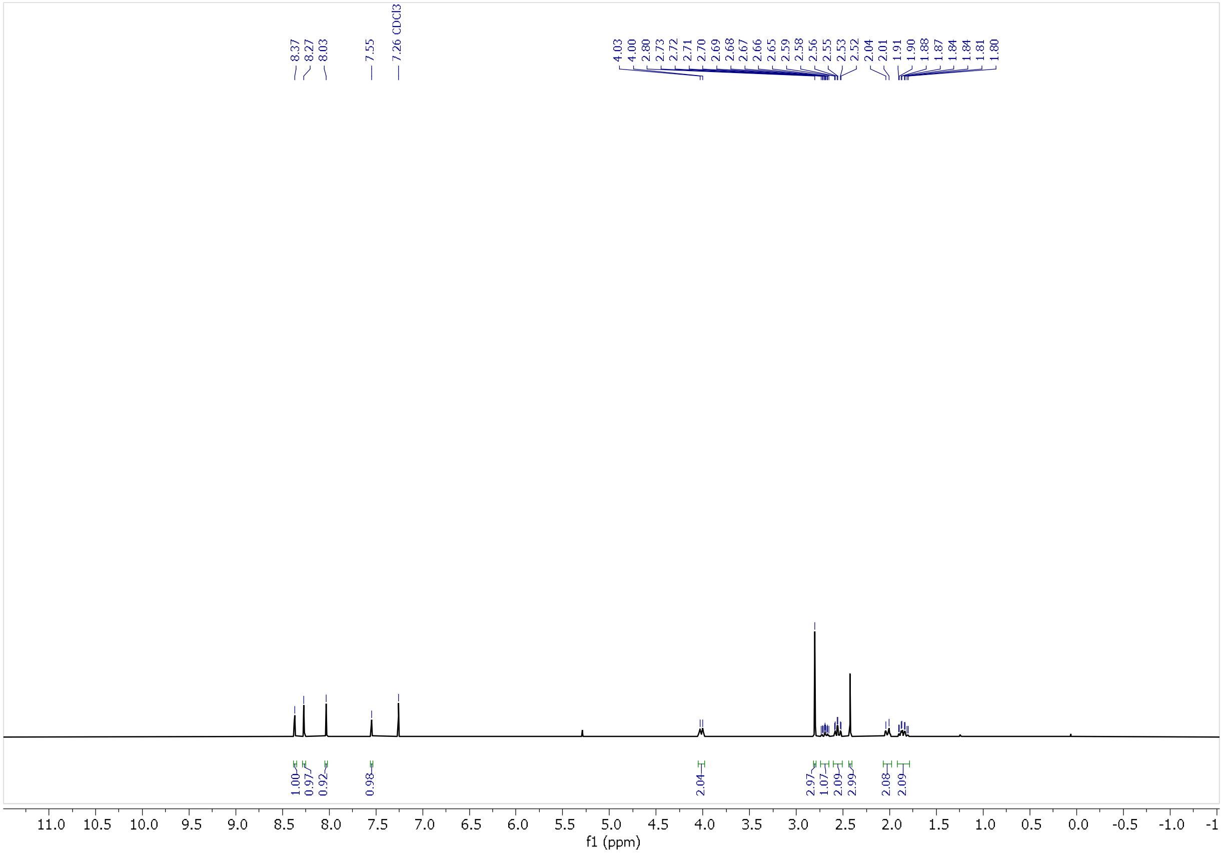

^{1}H NMR (400 MHz, $CDCl_3$) δ 8.37 (s, 1H), 8.27 (s, 1H), 8.03 (s, 1H), 7.55 (s, 1H), 4.02 (d, J = 11.6 Hz, 2H), 2.80 (s, 3H), 2.69 (tt, J = 12.2, 3.2 Hz, 1H), 2.56 (td, J = 12.0, 2.6 Hz, 2H), 2.42 (d, J = 1.0 Hz, 3H), 2.03 (d, J = 13.0 Hz, 2H), 1.86 (qd, J = 12.6, 3.9 Hz, 2H).

Compound 27

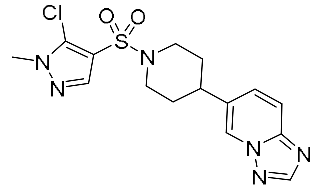

Chemical Formula: $C_{15}H_{17}ClN_6O_2S$

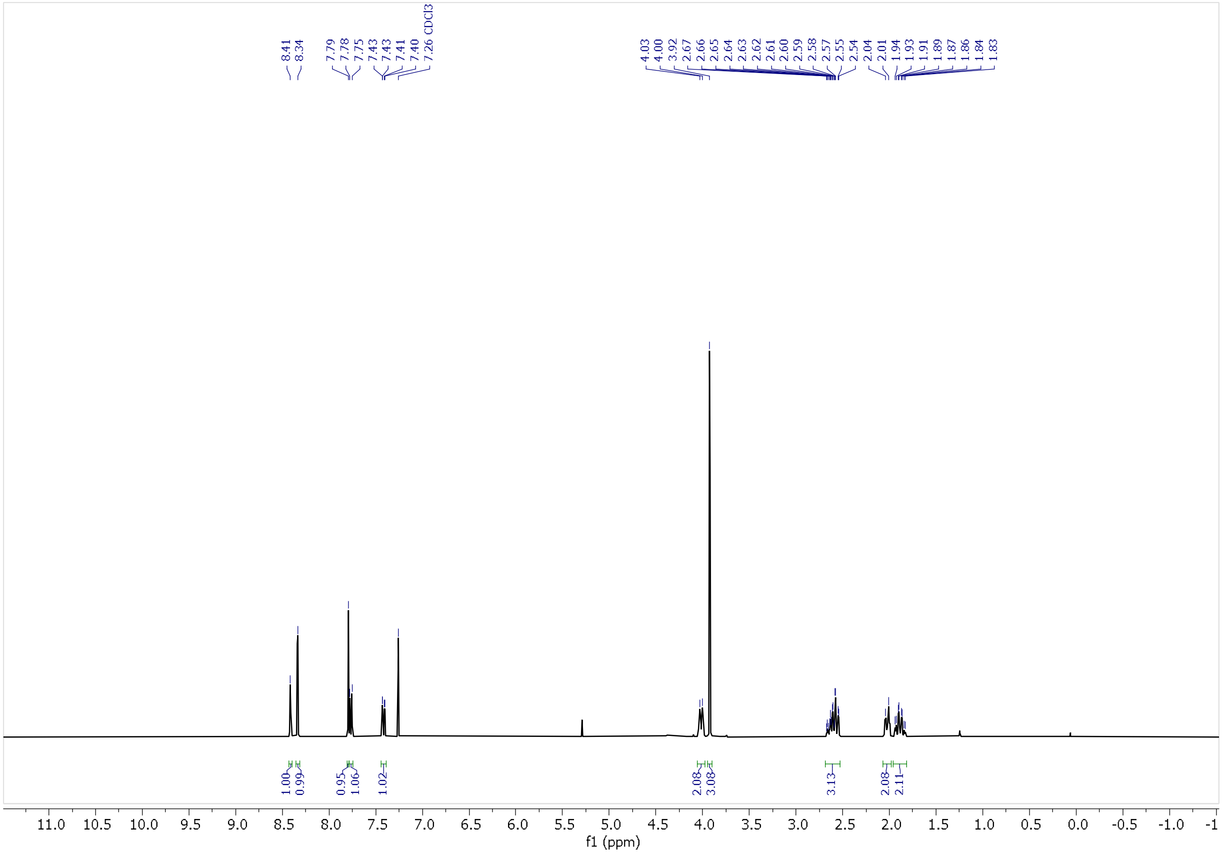

^{1}H NMR (400 MHz, $CDCl_3$) δ 8.41 (s, 1H), 8.34 (s, 1H), 7.79 (s, 1H), 7.77 (d, J = 9.2 Hz, 1H), 7.42 (dd, J = 9.2, 1.8 Hz, 1H), 4.02 (d, J = 11.8 Hz, 2H), 3.92 (s, 3H), 2.69 – 2.53 (m, 3H), 2.02 (d, J = 12.0 Hz, 2H), 1.88 (qd, J = 13.4, 12.8, 4.1 Hz, 2H).

Compound 28

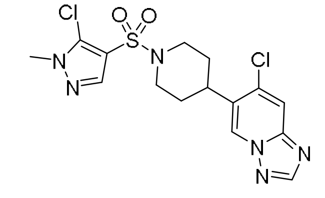

Chemical Formula: $C_{15}H_{16}Cl_2N_6O_2S$

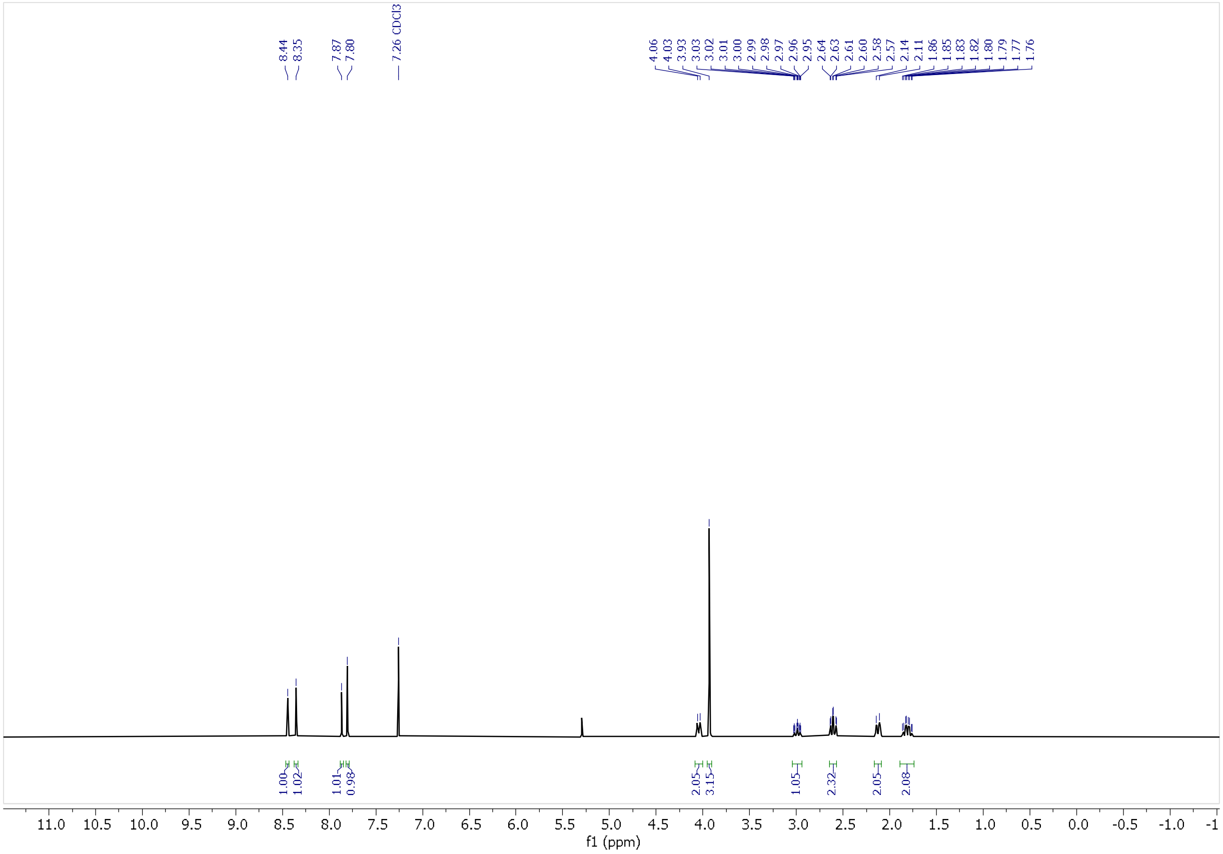

^{1}H NMR (400 MHz, $CDCl_3$) δ 8.44 (s, 1H), 8.35 (s, 1H), 7.87 (s, 1H), 7.80 (s, 1H), 4.05 (d, J = 11.8 Hz, 2H), 3.93 (s, 3H), 2.99 (tt, J = 12.3, 3.3 Hz, 1H), 2.61 (td, J = 12.2, 2.5 Hz, 2H), 2.13 (d, J = 12.7 Hz, 2H), 1.81 (qd, J = 12.5, 4.0 Hz, 2H).

Compound 29

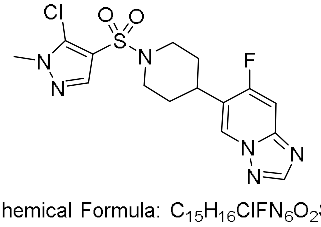

Chemical Formula: $C_{15}H_{16}ClFN_6O_2S$

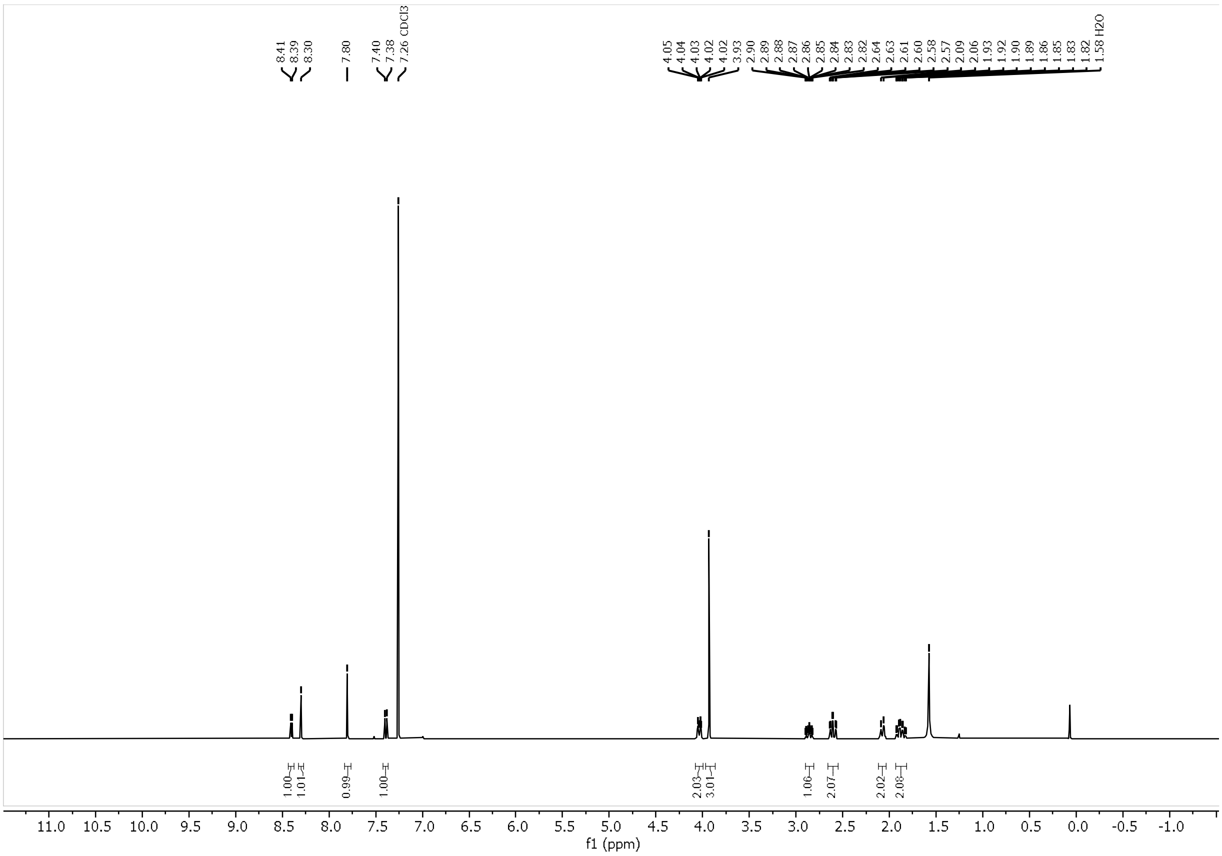

^{1}H NMR (400 MHz, $CDCl_3$) δ 8.40 (d, *J* = 6.5 Hz, 1H), 8.30 (s, 1H), 7.80 (s, 1H), 7.39 (d, *J* = 9.9 Hz, 1H), 4.07 – 3.99 (m, 2H), 3.93 (s, 3H), 2.86 (tt, *J* = 12.3, 3.4 Hz, 1H), 2.60 (td, *J* = 12.2, 2.4 Hz, 2H), 2.08 (d, *J* = 13.0 Hz, 2H), 1.87 (qd, *J* = 12.6, 4.1 Hz, 2H).

Compound 30

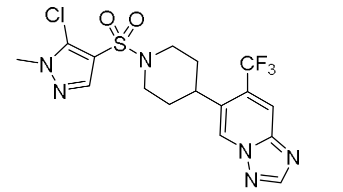

Chemical Formula: $C_{16}H_{16}ClF_3N_6O_2S$

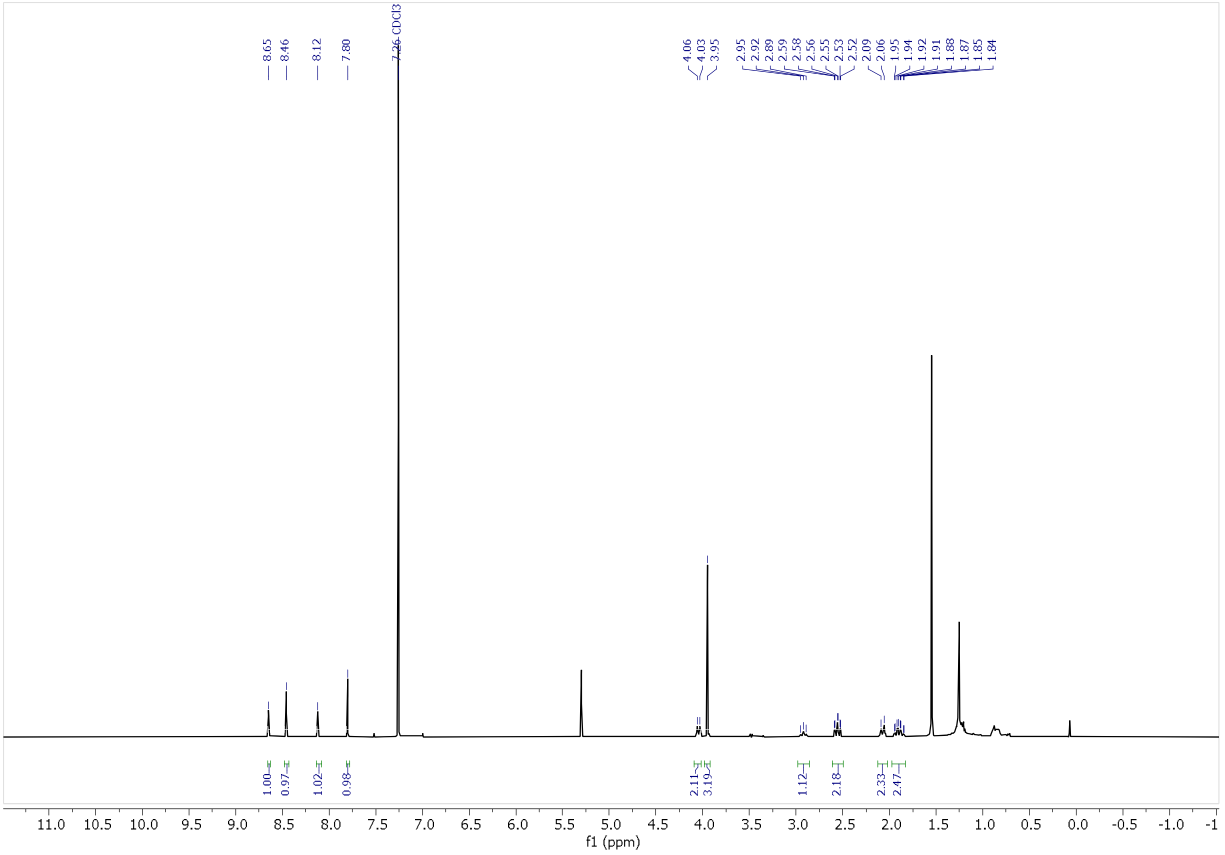

^{1}H NMR (400 MHz, $CDCl_3$) δ 8.65 (s, 1H), 8.46 (s, 1H), 8.12 (s, 1H), 7.80 (s, 1H), 4.04 (d, J = 11.5 Hz, 2H), 3.95 (s, 3H), 2.92 (t, J = 12.2 Hz, 1H), 2.55 (td, J = 12.2, 2.3 Hz, 2H), 2.07 (d, J = 13.0 Hz, 2H), 1.89 (qd, J = 12.6, 3.9 Hz, 2H).

Compound 31

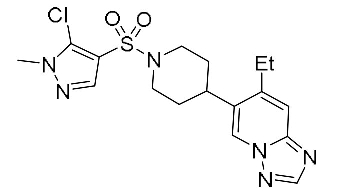

Chemical Formula: $C_{17}H_{21}ClN_6O_2S$

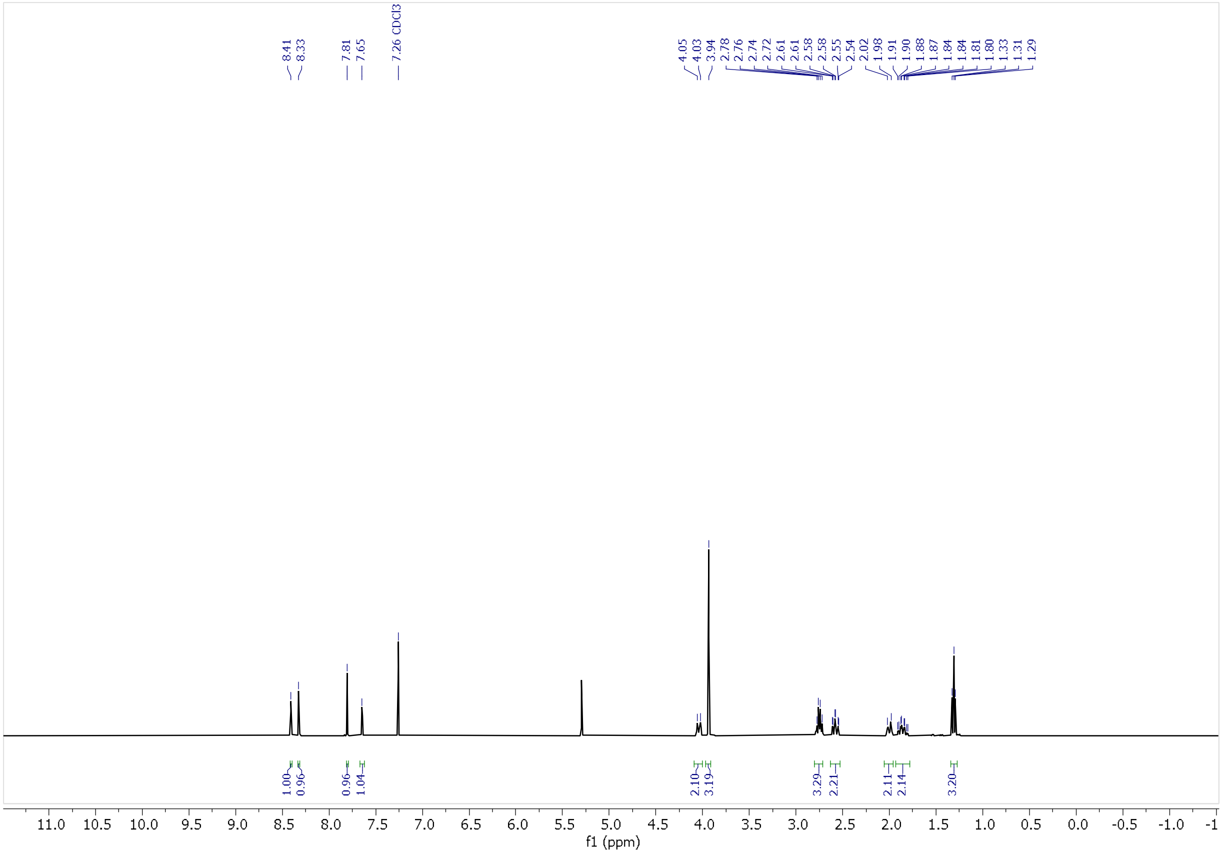

^{1}H NMR (400 MHz, $CDCl_3$) δ 8.41 (s, 1H), 8.33 (s, 1H), 7.81 (s, 1H), 7.65 (s, 1H), 4.04 (d, J = 11.8 Hz, 2H), 3.94 (s, 3H), 2.75 (q, J = 7.3 Hz, 3H), 2.58 (td, J = 12.0, 2.5 Hz, 2H), 2.00 (d, J = 14.1 Hz, 2H), 1.86 (qd, J = 13.4, 12.7, 4.0 Hz, 2H), 1.31 (t, J = 7.4 Hz, 3H).

Compound 32

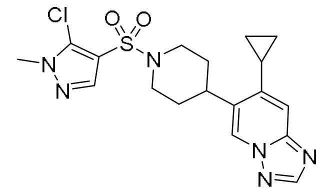

Chemical Formula: $C_{18}H_{21}ClN_6O_2S$

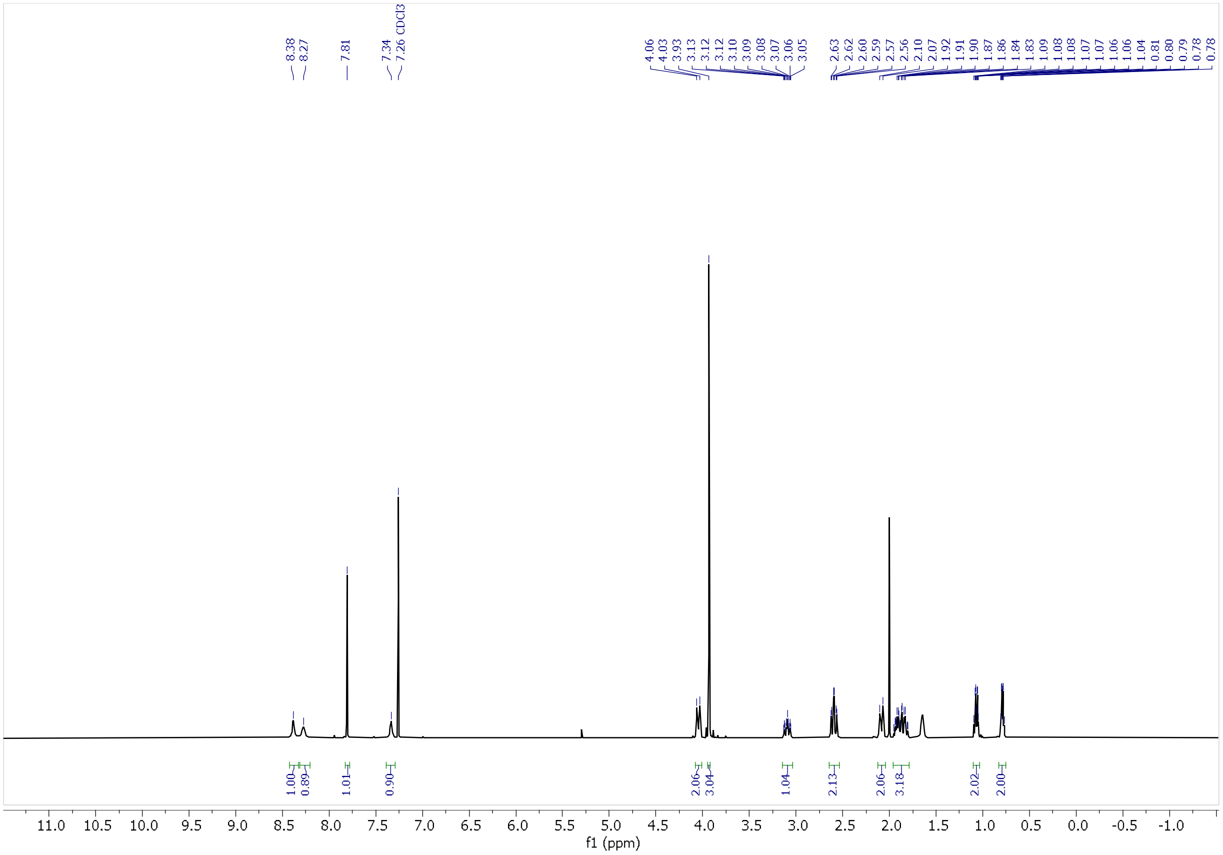

^{1}H NMR (400 MHz, $CDCl_3$) δ 8.38 (s, 1H), 8.27 (s, 1H), 7.81 (s, 1H), 7.34 (s, 1H), 4.05 (d, *J* = 11.8 Hz, 2H), 3.93 (s, 3H), 3.09 (tt, *J* = 12.2, 3.4 Hz, 1H), 2.59 (td, *J* = 12.2, 2.5 Hz, 2H), 2.08 (d, *J* = 14.0 Hz, 2H), 1.96 – 1.78 (m, 3H), 1.11 – 1.04 (m, 2H), 0.83 – 0.76 (m, 2H). *Acetone peak at 2.0 ppm.

Compound 33

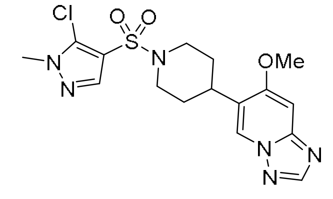

Chemical Formula: $C_{16}H_{19}ClN_6O_3S$

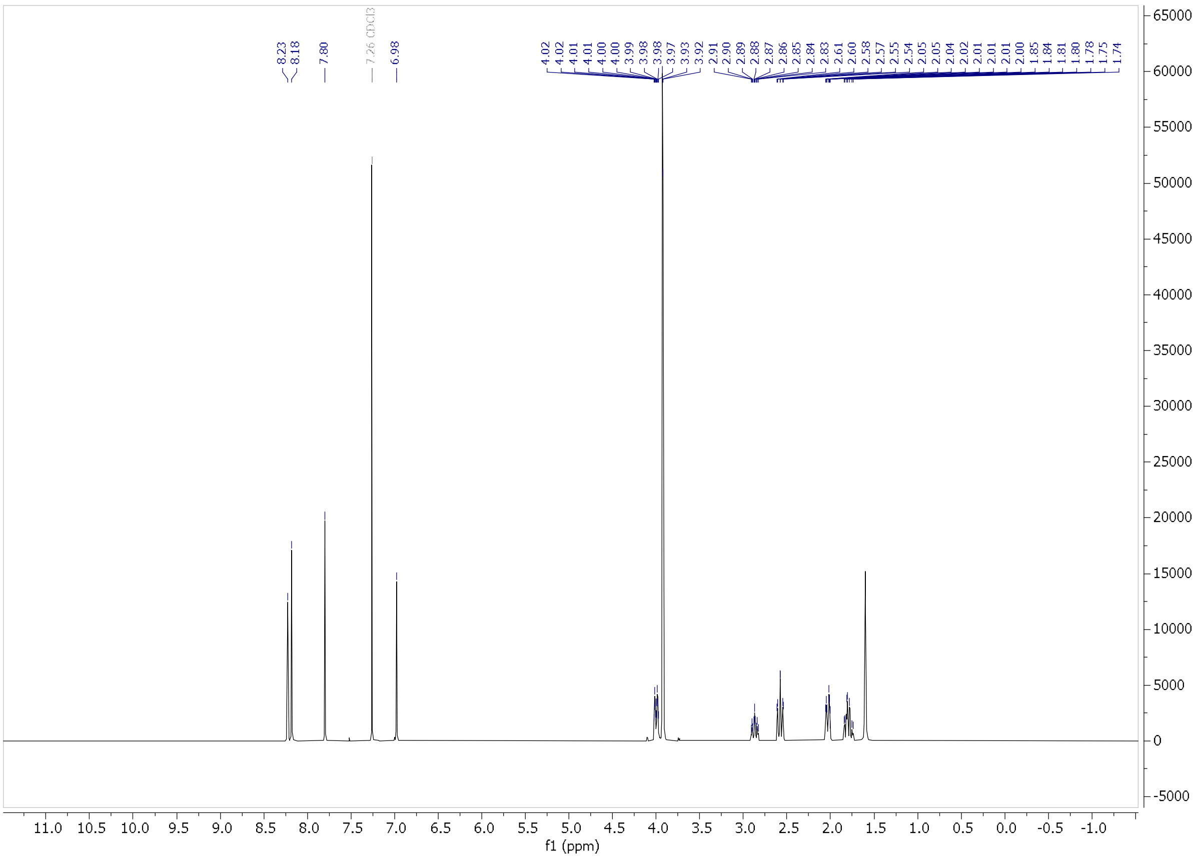

^{1}H NMR (400 MHz, $CDCl_3$) δ 8.23 (s, 1H), 8.18 (s, 1H), 7.80 (s, 1H), 6.98 (s, 1H), 4.00 (dp, *J* = 11.7, 1.9 Hz, 2H), 3.93 (s, 3H), 3.92 (s, 3H), 2.87 (tt, *J* = 12.3, 3.3 Hz, 1H), 2.58 (td, *J* = 12.1, 2.4 Hz, 2H), 2.03 (dt, *J* = 12.6, 2.5 Hz, 2H), 1.87 – 1.73 (m, 2H).

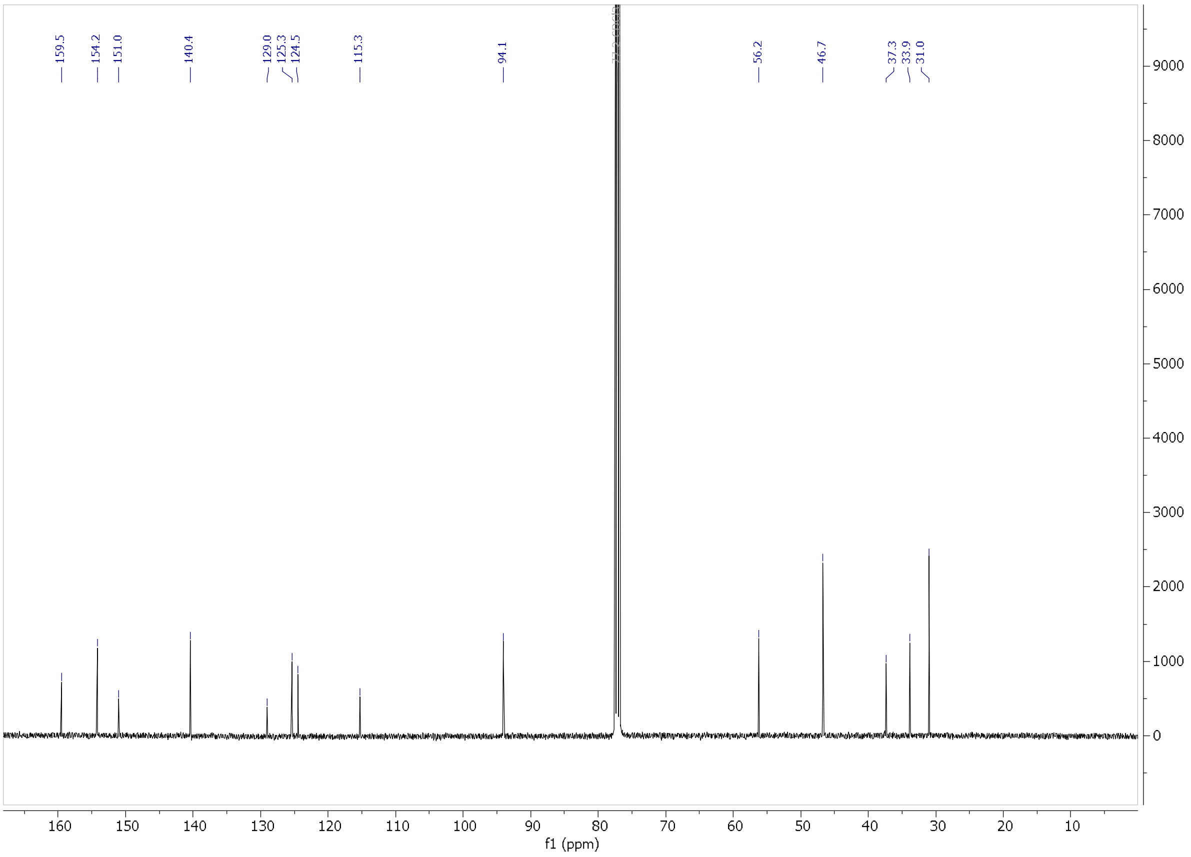

^{13}C NMR (101 MHz, $CDCl_3$) δ 159.5, 154.2, 151.0, 140.4, 129.0, 125.3, 124.5, 115.3, 94.1, 56.2, 46.7 (2), 37.3, 33.9, 31.0 (2).

Compound 34

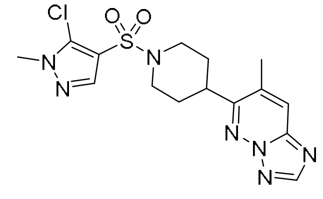

Chemical Formula: $C_{15}H_{18}ClN_7O_2S$

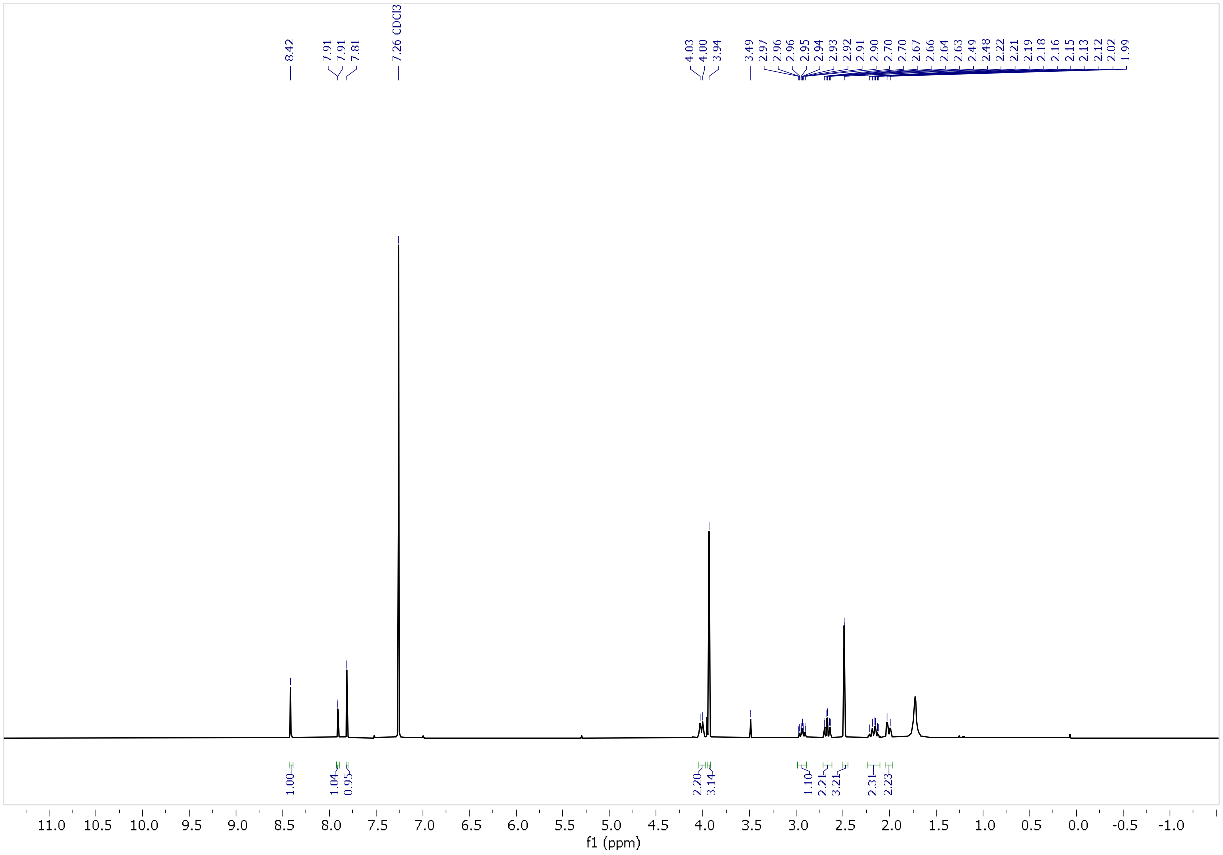

^{1}H NMR (400 MHz, $CDCl_3$) δ 8.42 (s, 1H), 7.91 (d, J = 1.1 Hz, 1H), 7.81 (s, 1H), 4.02 (d, J = 12.2 Hz, 2H), 3.94 (s, 3H), 2.94 (tt, J = 11.2, 3.5 Hz, 1H), 2.67 (td, J = 12.1, 2.7 Hz, 2H), 2.49 (d, J = 1.1 Hz, 3H), 2.24 – 2.10 (m, 2H), 2.01 (d, J = 12.6 Hz, 2H).

Compound 35

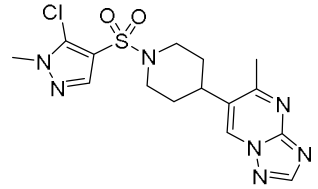

Chemical Formula: $C_{15}H_{18}ClN_7O_2S$

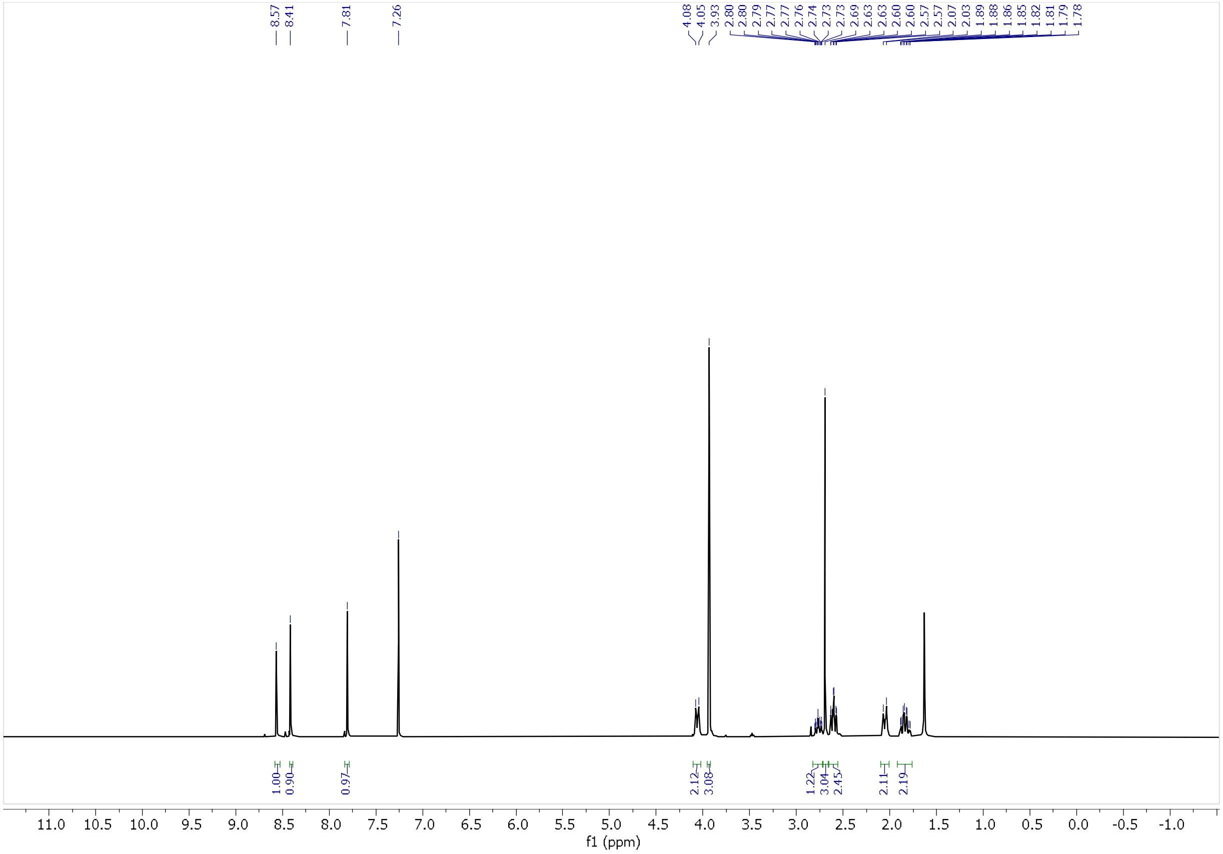

^{1}H NMR (400 MHz, CDCl$_3$) δ 8.57 (s, 1H), 8.41 (s, 1H), 7.81 (s, 1H), 4.06 (d, J = 11.8 Hz, 2H), 3.93 (s, 3H), 2.77 (tt, J = 12.0, 3.2 Hz, 1H), 2.69 (s, 3H), 2.60 (td, J = 12.2, 2.5 Hz, 2H), 2.05 (d, J = 13.5 Hz, 2H), 1.83 (qd, J = 12.7, 4.0 Hz, 2H).

Compound 36

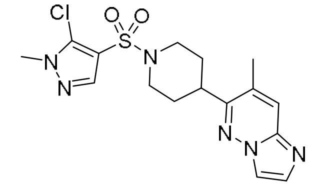

Chemical Formula: $C_{16}H_{19}ClN_6O_2S$

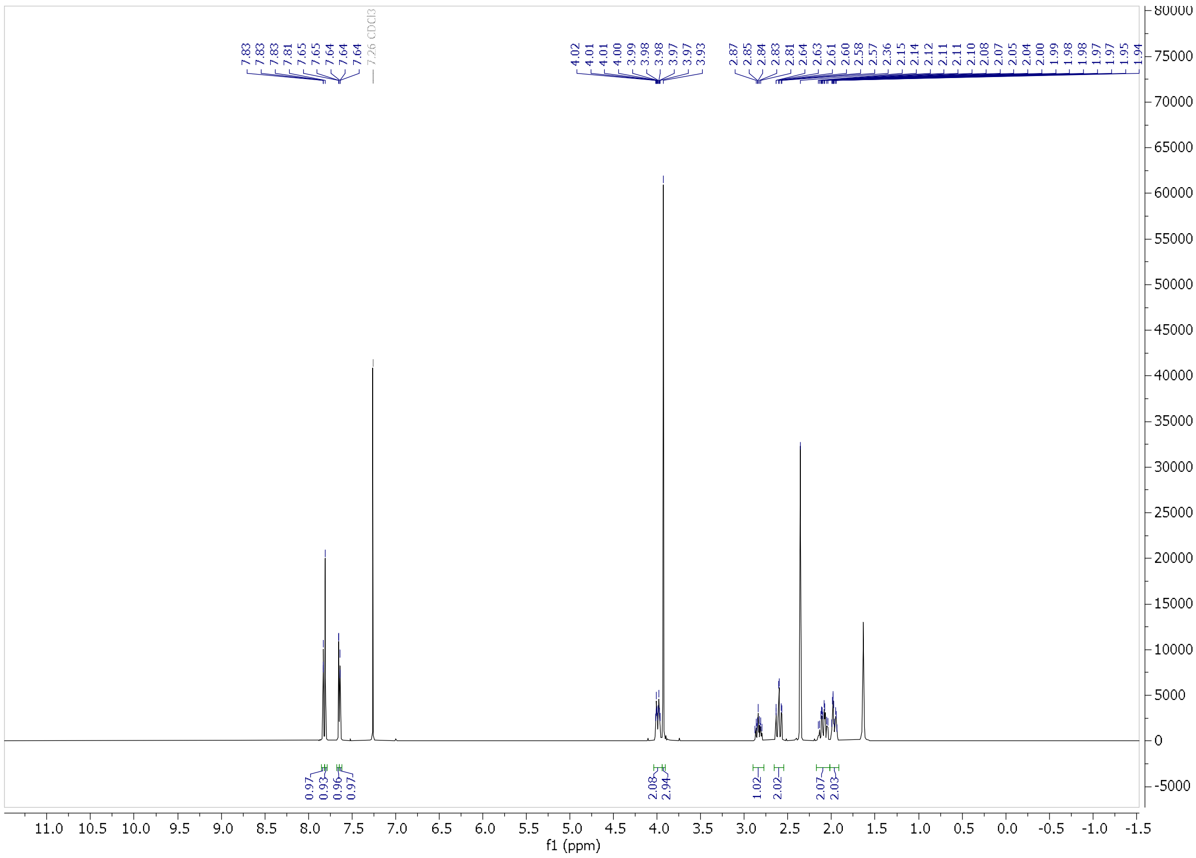

^{1}H NMR (400 MHz, $CDCl_3$) δ 7.83 (t, J = 1.0 Hz, 1H), 7.81 (s, 1H), 7.65 (d, J = 1.3 Hz, 1H), 7.64 (t, J = 1.0 Hz, 2H), 4.04 – 3.94 (m, 2H), 3.93 (s, 3H), 2.84 (tt, J = 11.4, 3.6 Hz, 1H), 2.60 (td, J = 12.0, 2.7 Hz, 2H), 2.36 (d, J = 1.1 Hz, 1H), 2.17 – 2.02 (m, 2H), 2.02 – 1.91 (m, 2H).

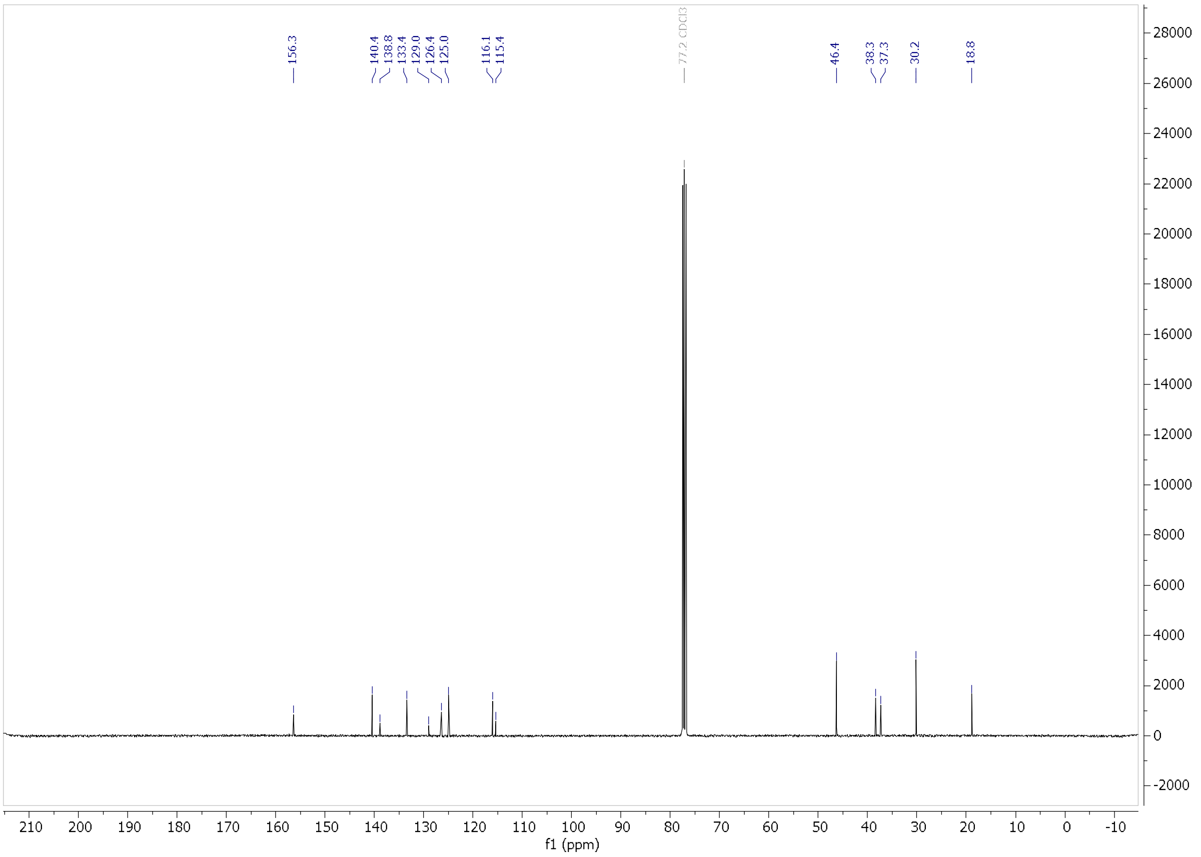

^{13}C NMR (101 MHz, $CDCl_3$) δ 156.3, 140.4, 138.8, 133.4, 129.0, 126.4, 125.0, 116.1, 115.4, 46.4 (2), 38.3, 37.3, 30.2 (2), 18.8.

Compound 37

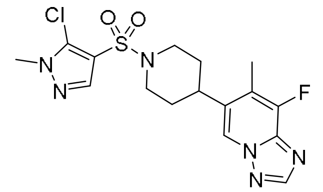

Chemical Formula: $C_{16}H_{18}ClFN_6O_2S$

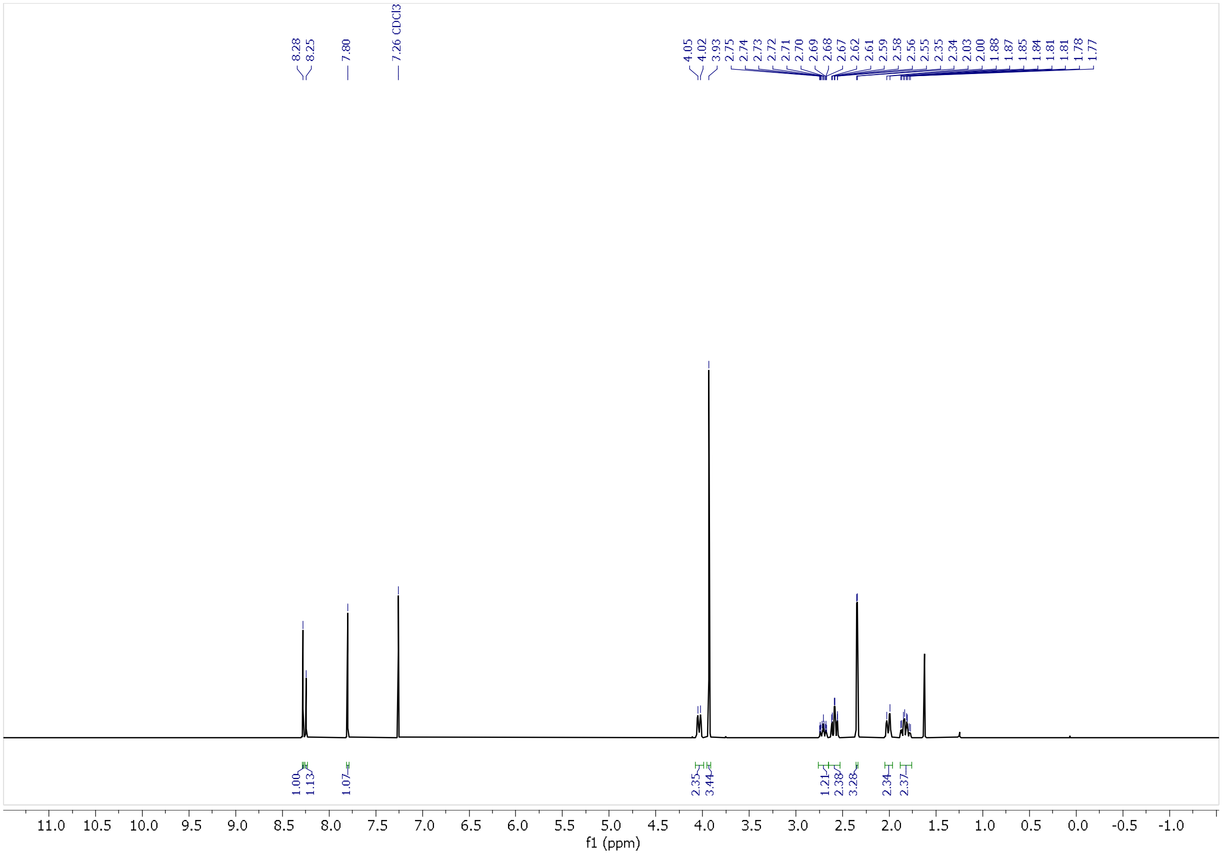

^{1}H NMR (400 MHz, $CDCl_3$) δ 8.28 (s, 1H), 8.25 (s, 1H), 7.80 (s, 1H), 4.04 (d, *J* = 11.7 Hz, 2H), 3.93 (s, 3H), 2.71 (tt, *J* = 12.1, 3.3 Hz, 1H), 2.59 (td, *J* = 12.1, 2.5 Hz, 2H), 2.35 (d, *J* = 2.9 Hz, 3H), 2.01 (d, *J* = 13.6 Hz, 2H), 1.83 (qd, *J* = 13.4, 12.8, 4.1 Hz, 2H).

Compound 38

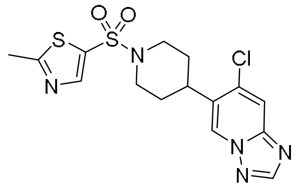

Chemical Formula: $C_{15}H_{16}ClN_5O_2S_2$

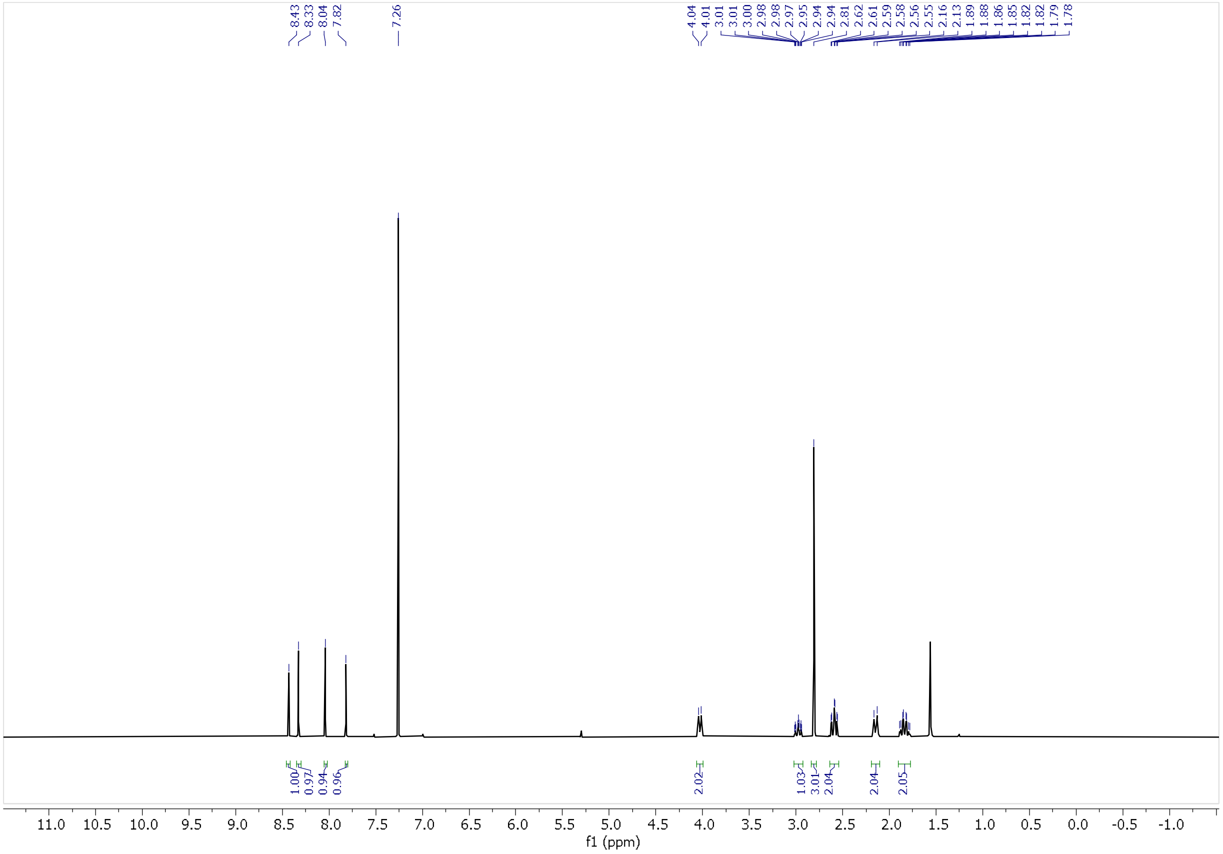

^{1}H NMR (400 MHz, $CDCl_3$) δ 8.43 (s, 1H), 8.33 (s, 1H), 8.04 (s, 1H), 7.82 (s, 1H), 4.03 (d, *J* = 11.4 Hz, 2H), 2.98 (tt, *J* = 12.3, 2.9 Hz, 1H), 2.81 (s, 3H), 2.59 (td, *J* = 12.1, 2.5 Hz, 2H), 2.15 (d, *J* = 13.3 Hz, 2H), 1.84 (qd, *J* = 13.3, 12.8, 4.2 Hz, 2H).

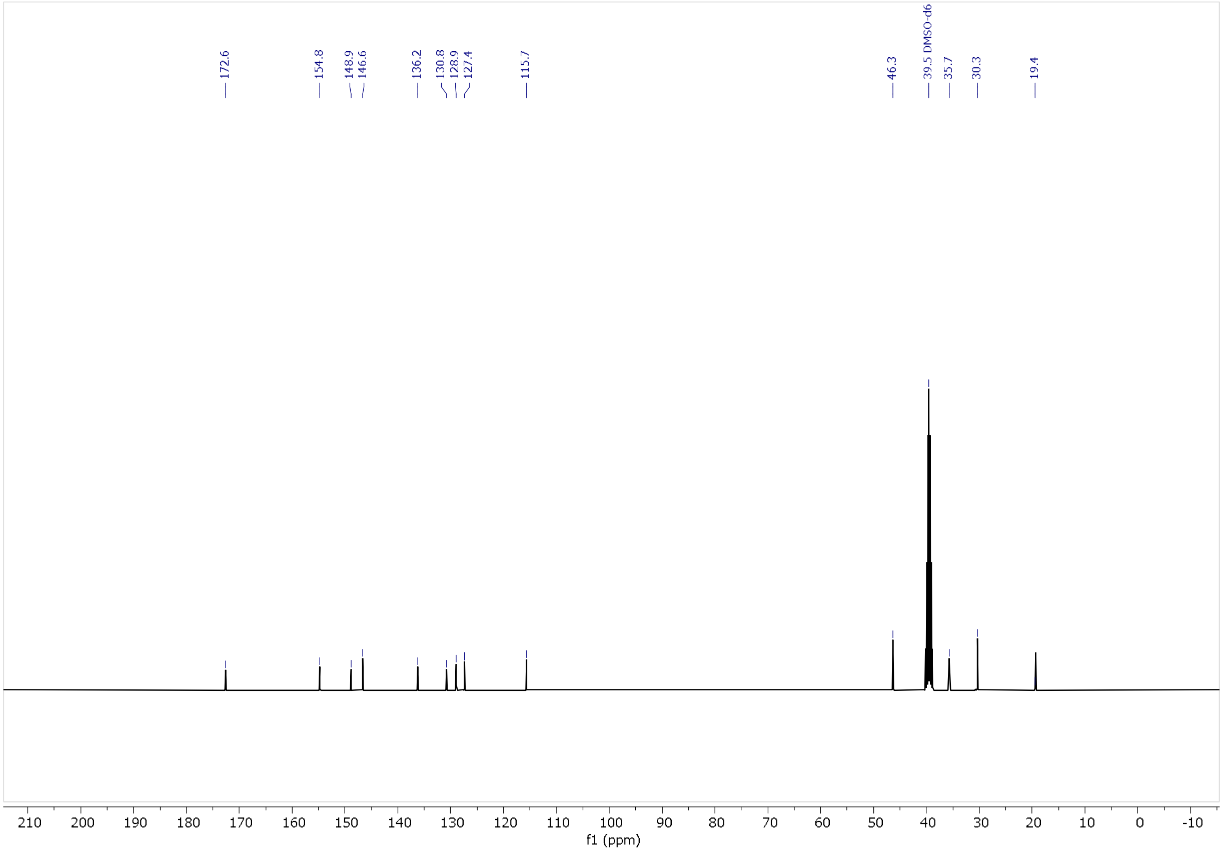

^{13}C NMR (101 MHz, DMSO) δ 172.6, 154.8, 148.9, 146.6, 136.2, 130.8, 128.9, 127.4, 115.7, 46.3 (2), 35.7, 30.3 (2), 19.4.

MP: 230.2-232.1 ºC

Compound 39

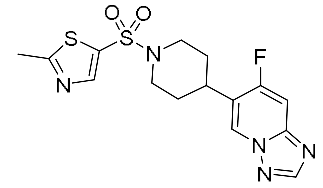

Chemical Formula: $C_{15}H_{16}FN_5O_2S_2$

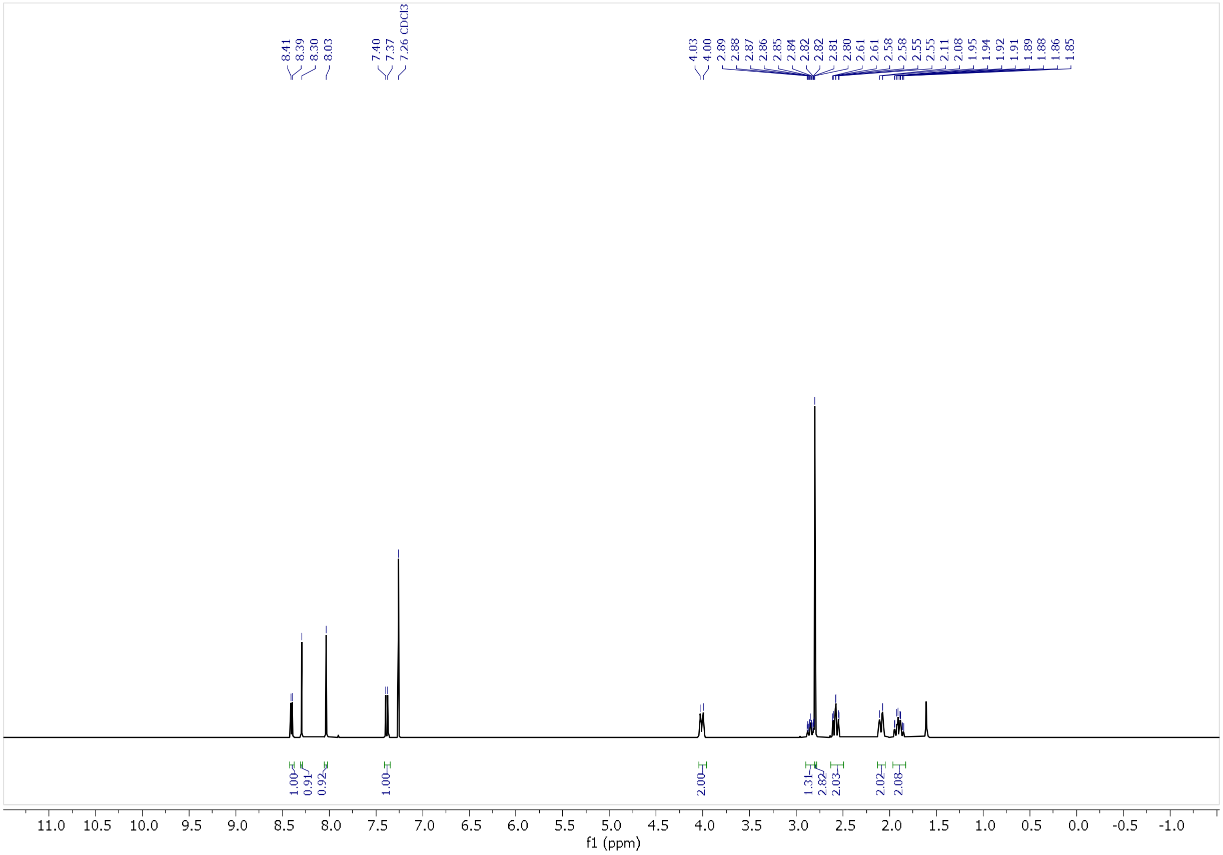

^{1}H NMR (400 MHz, $CDCl_3$) δ 8.40 (d, J = 6.6 Hz, 1H), 8.30 (s, 1H), 8.03 (s, 1H), 7.39 (d, J = 9.9 Hz, 1H), 4.01 (d, J = 11.6 Hz, 2H), 2.85 (ddd, J = 16.0, 12.6, 3.6 Hz, 1H), 2.80 (s, 3H), 2.58 (td, J = 12.2, 2.6 Hz, 2H), 2.09 (d, J = 14.1 Hz, 2H), 1.90 (qd, J = 12.6, 4.1 Hz, 2H).

Compound 40

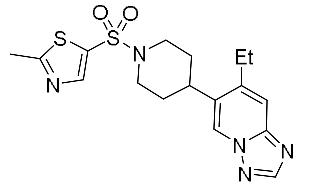

Chemical Formula: $C_{17}H_{21}N_5O_2S_2$

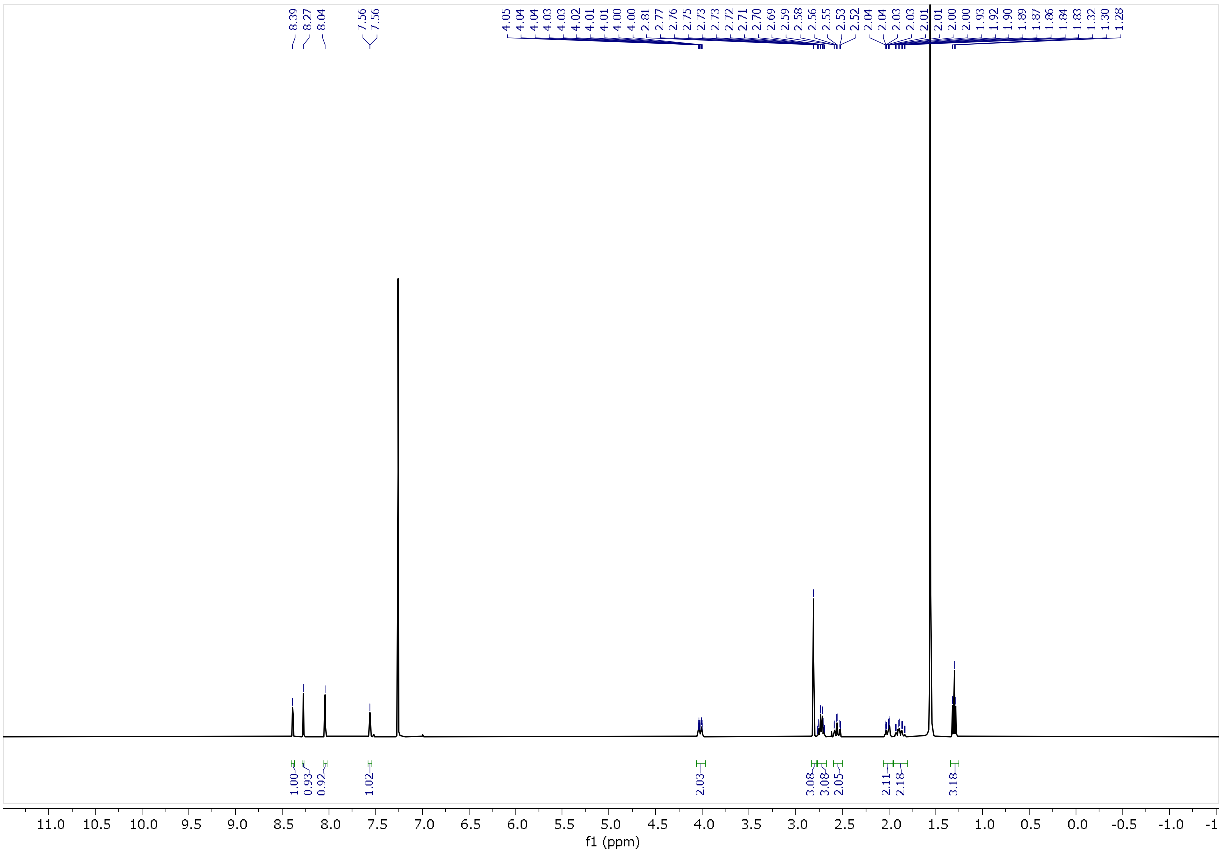

^{1}H NMR (400 MHz, $CDCl_3$) δ 8.39 (s, 1H), 8.27 (s, 1H), 8.04 (s, 1H), 7.58 – 7.54 (m, 1H), 4.02 (ddt, *J* = 11.9, 4.2, 1.9 Hz, 2H), 2.81 (s, 3H), 2.77 – 2.67 (m, 3H), 2.56 (td, *J* = 12.1, 2.6 Hz, 2H), 2.02 (ddd, *J* = 12.6, 4.0, 1.9 Hz, 2H), 1.88 (qd, *J* = 12.5, 3.9 Hz, 2H), 1.30 (t, *J* = 7.4 Hz, 3H).

Compound 41

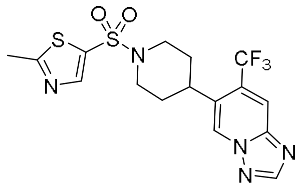

Chemical Formula: $C_{16}H_{16}F_{3}N_{5}O_{2}S_{2}$

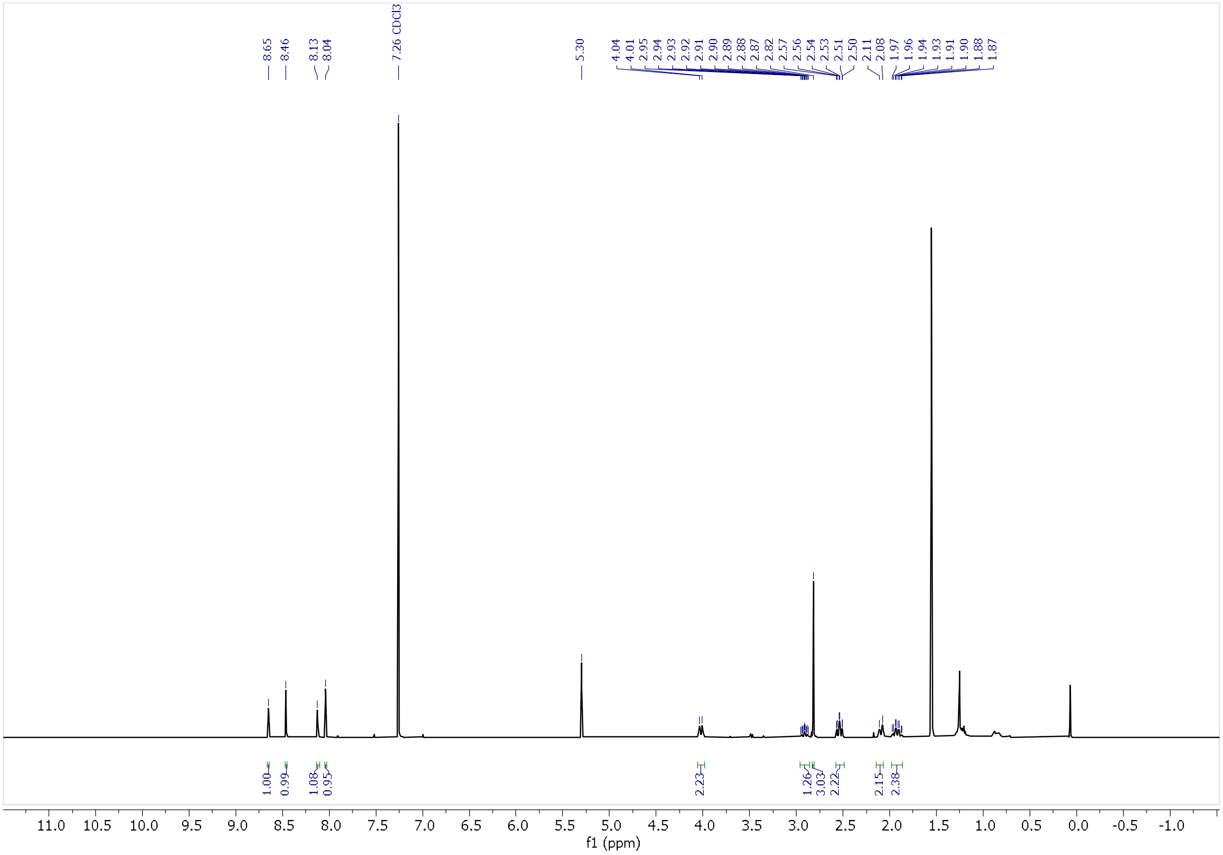

^{1}H NMR (400 MHz, $CDCl_3$) δ 8.65 (s, 1H), 8.46 (s, 1H), 8.13 (s, 1H), 8.04 (s, 1H), 4.02 (d, J = 11.8 Hz, 2H), 2.96 – 2.86 (m, 1H), 2.82 (s, 3H), 2.54 (td, J = 12.1, 2.6 Hz, 2H), 2.09 (d, J = 13.2 Hz, 2H), 1.92 (qd, J = 12.5, 3.7 Hz, 2H).

Compound 42

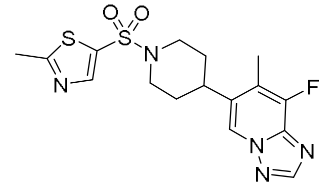

Chemical Formula: $C_{16}H_{18}FN_5O_2S_2$

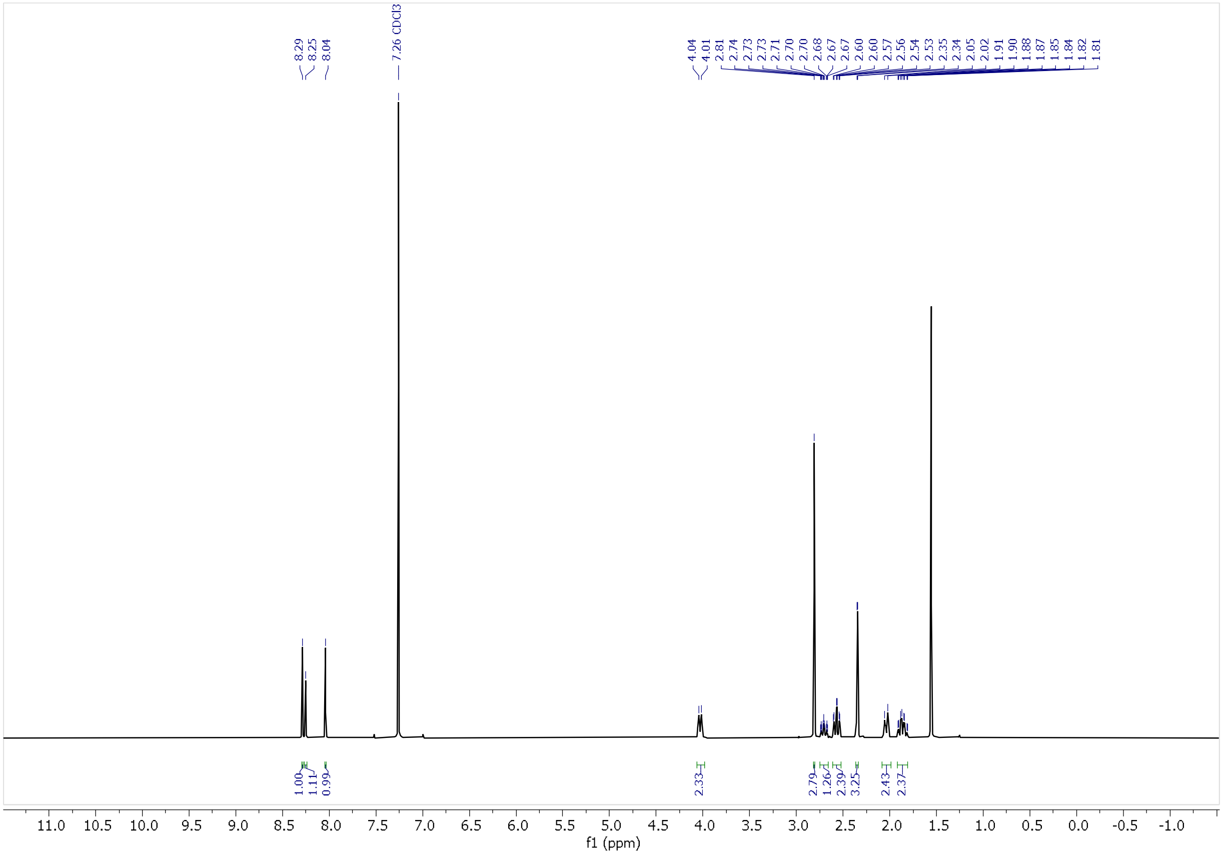

^{1}H NMR (400 MHz, $CDCl_3$) δ 8.29 (s, 1H), 8.25 (s, 1H), 8.04 (s, 1H), 4.03 (d, J = 11.7 Hz, 2H), 2.81 (s, 3H), 2.70 (tt, J = 12.2, 3.2 Hz, 1H), 2.57 (td, J = 12.0, 2.5 Hz, 2H), 2.35 (d, J = 2.9 Hz, 3H), 2.04 (d, J = 13.8 Hz, 2H), 1.86 (qd, J = 13.1, 12.6, 4.0 Hz, 2H).

Compound 43

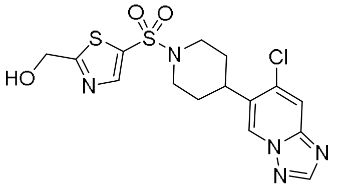

Chemical Formula: $C_{15}H_{16}ClN_5O_3S_2$

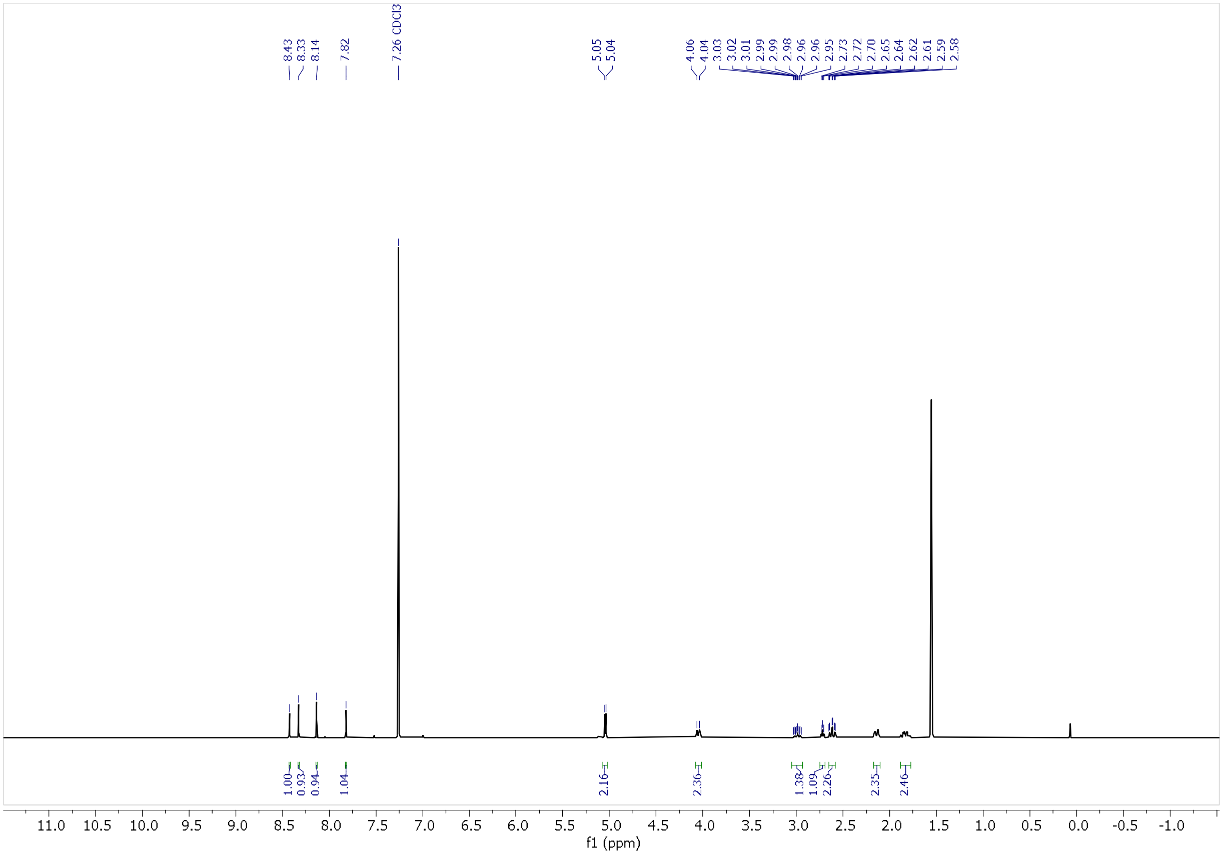

^{1}H NMR (400 MHz, $CDCl_3$) δ 8.43 (s, 1H), 8.33 (s, 1H), 8.14 (s, 1H), 7.82 (s, 1H), 5.05 (d, J = 5.9 Hz, 2H), 4.05 (d, J = 11.5 Hz, 2H), 2.99 (tt, J = 12.2, 3.1 Hz, 1H), 2.72 (t, J = 5.9 Hz, 1H), 2.62 (td, J = 12.0, 2.4 Hz, 2H), 2.14 (d, J = 12.9 Hz, 2H), 1.83 (qd, J = 12.6, 4.1 Hz, 2H).

Compound 44

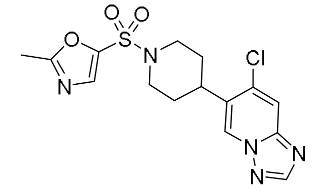

Chemical Formula: $C_{15}H_{16}ClN_5O_3S$

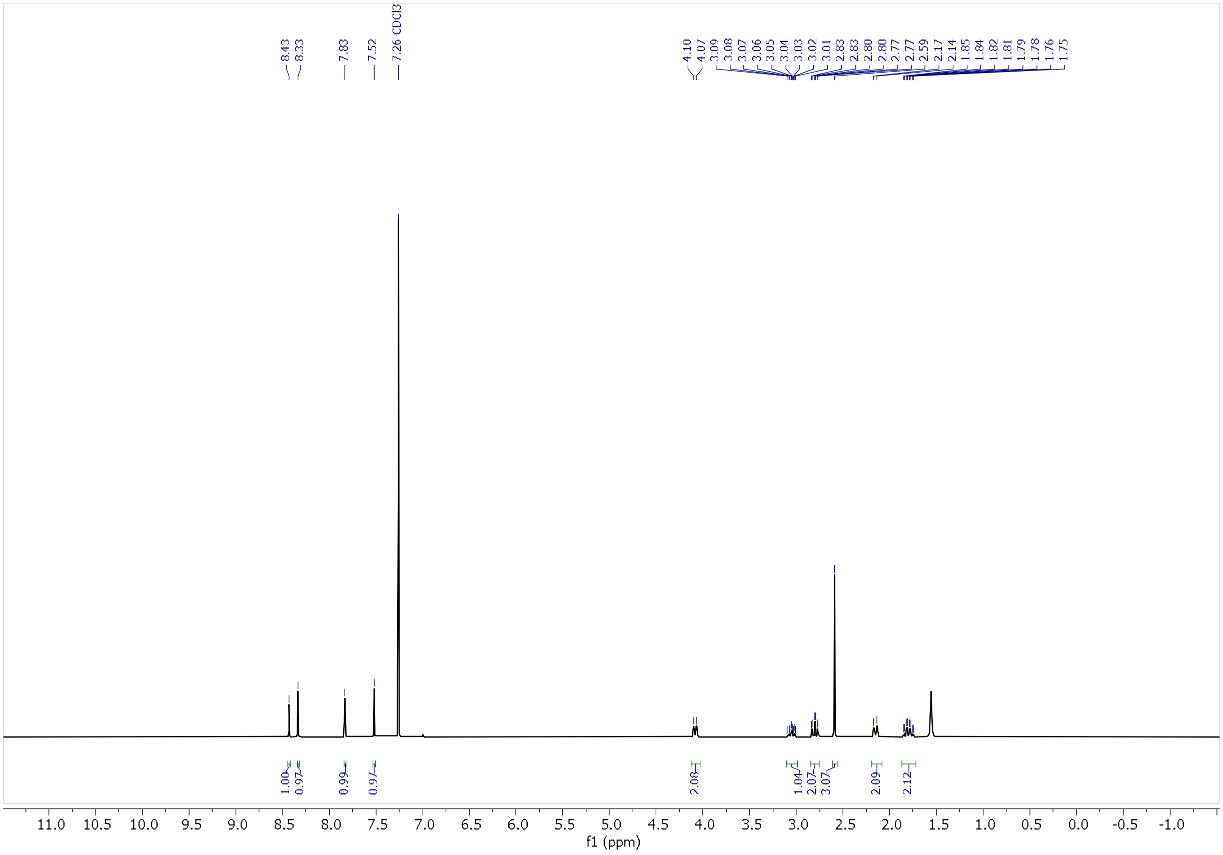

^{1}H NMR (400 MHz, $CDCl_3$) δ 8.43 (s, 1H), 8.33 (s, 1H), 7.83 (s, 1H), 7.52 (s, 1H), 4.08 (d, J = 12.2 Hz, 2H), 3.05 (tt, J = 12.1, 3.0 Hz, 1H), 2.80 (td, J = 12.4, 2.5 Hz, 2H), 2.59 (s, 3H), 2.15 (d, J = 13.1 Hz, 2H), 1.80 (qd, J = 12.6, 4.1 Hz, 2H).

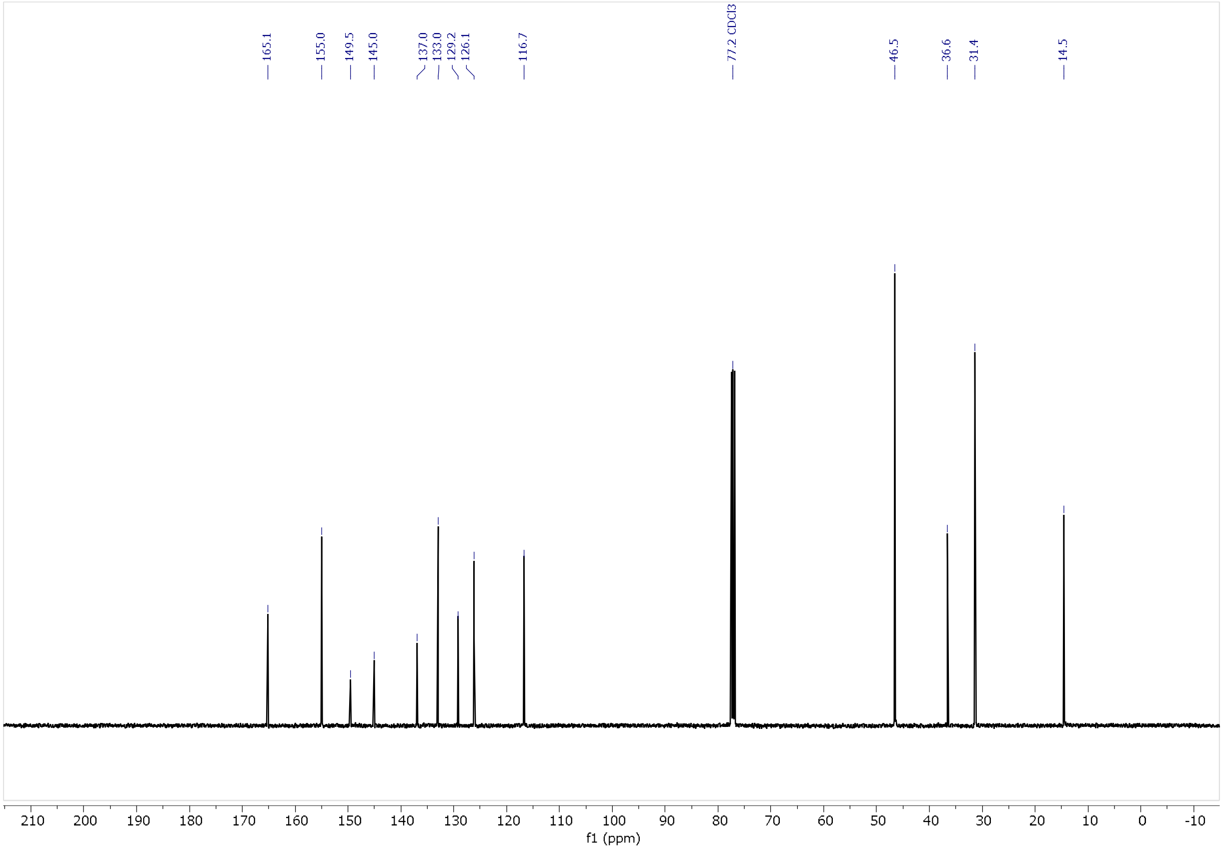

^{13}C NMR (101 MHz, $CDCl_3$) δ 165.1, 155.0, 149.5, 145.0, 137.0, 133.0, 129.2, 126.1, 116.7, 46.5 (2), 36.6, 31.4 (2), 14.5.

MP: 185.1-188.8 ºC

Compound 45

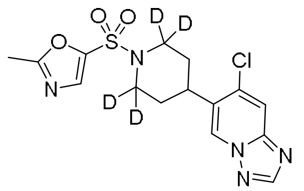

Chemical Formula: $C_{15}H_{12}D_4ClN_5O_3S$

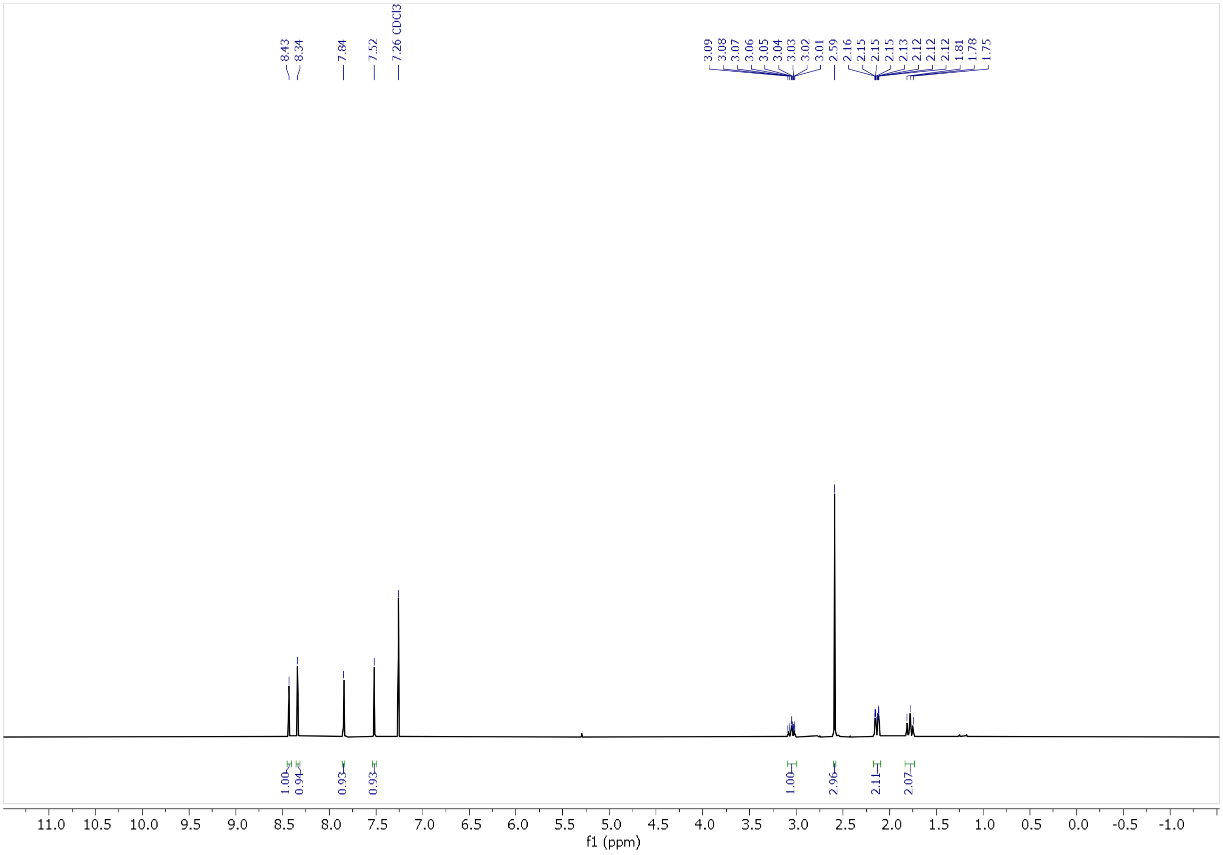

^{1}H NMR (400 MHz, $CDCl_3$) δ 8.43 (s, 1H), 8.34 (s, 1H), 7.84 (s, 1H), 7.52 (s, 1H), 3.05 (tt, J = 12.4, 3.3 Hz, 1H), 2.59 (s, 3H), 2.17 – 2.09 (m, 2H), 1.78 (t, J = 12.9 Hz, 2H).

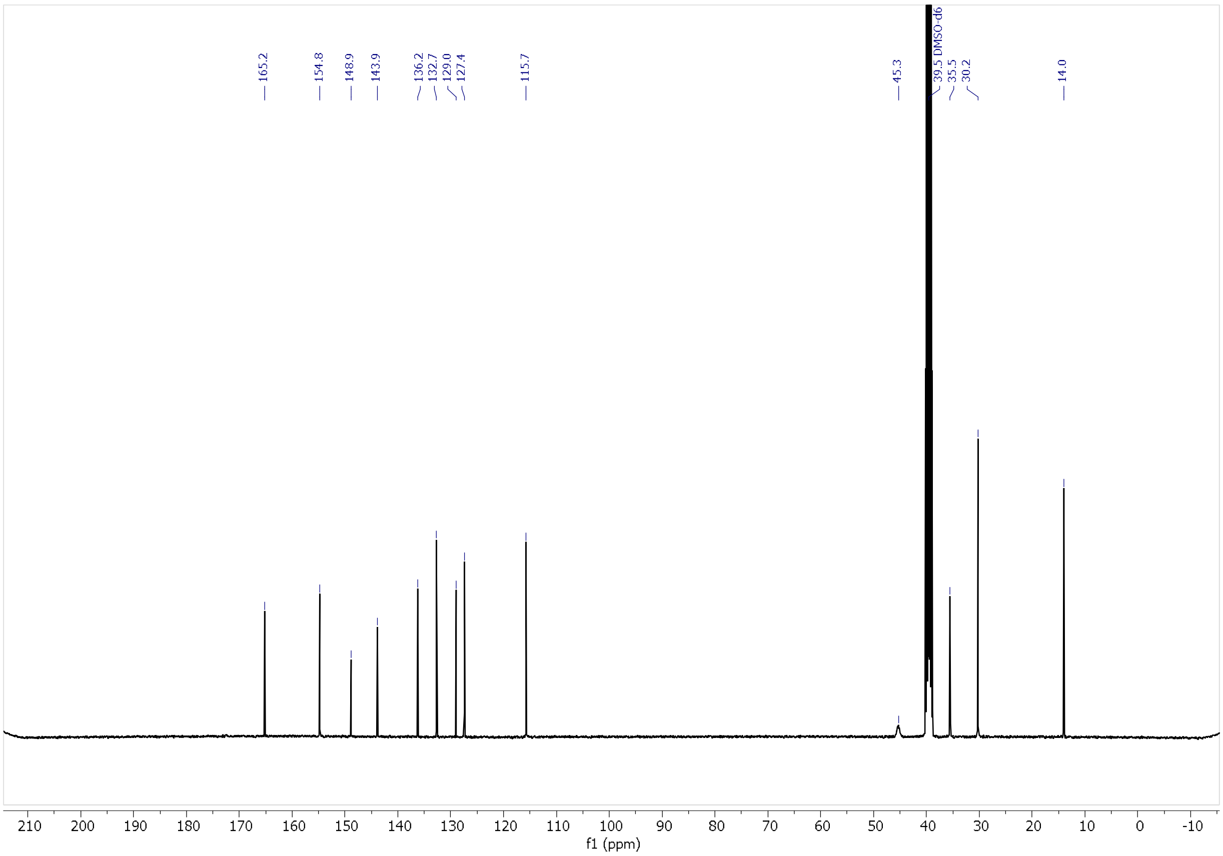

^{13}C NMR (101 MHz, DMSO) δ 165.2, 154.8, 148.9, 143.9, 136.2, 132.7, 129.0, 127.4, 115.7, 45.3 (m, 2), 35.5, 30.2 (2), 14.0.

MP: 184.3-186.8 ºC

HRMS data

Structure	Cmpd	Chemical Formula	Ion	(TOF, ES+) Calc. mass	Found mass
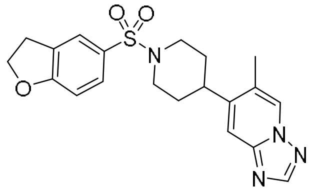	**9**	$C_{20}H_{22}N_4O_3S$	$(M+H)^+$	399.1485	399.1483
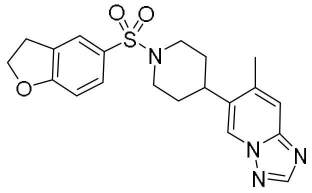	**10**	$C_{20}H_{22}N_4O_3S$	$(M+H)^+$	399.1485	399.1480
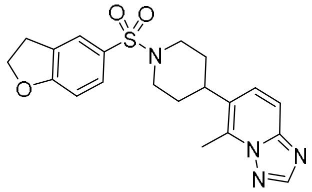	**11**	$C_{20}H_{22}N_4O_3S$	$(M+H)^+$	399.1485	399.1476
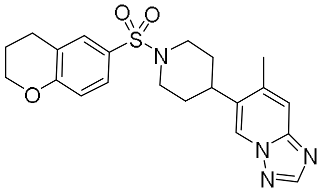	**12**	$C_{21}H_{24}N_4O_3S$	$(M+H)^+$	413.1642	413.1636
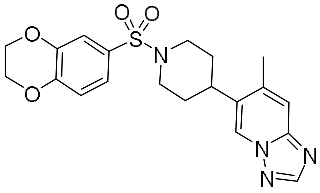	**13**	$C_{20}H_{22}N_4O_4S$	$(M+H)^+$	415.1435	415.1432
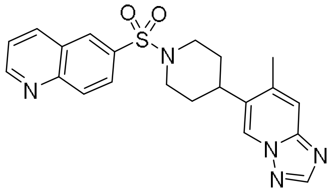	**14**	$C_{21}H_{21}N_5O_2S$	$(M+H)^+$	408.1489	408.1483

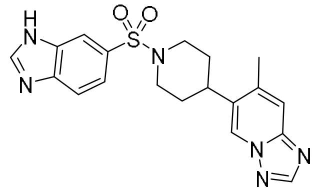	**15**	$C_{19}H_{20}N_6O_2S$	$(M+H)^+$	397.1441	397.1436
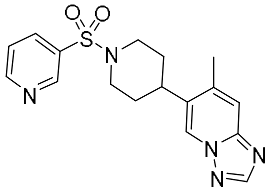	**16**	$C_{17}H_{19}N_5O_2S$	$(M+H)^+$	358.1332	358.1327
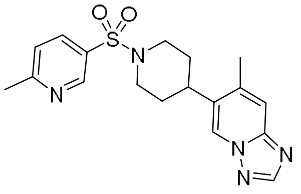	**17**	$C_{18}H_{21}N_5O_2S$	$(M+H)^+$	372.1489	372.1482
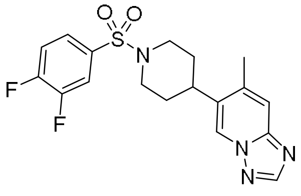	**18**	$C_{18}H_{18}F_2N_4O_2S$	$(M+H)^+$	393.1191	393.1185
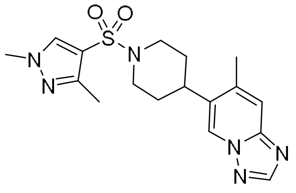	**19**	$C_{17}H_{22}N_6O_2S$	$(M+H)^+$	375.1598	375.1593
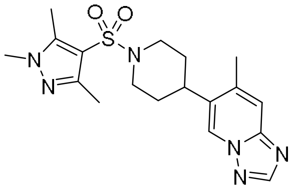	**20**	$C_{18}H_{24}N_6O_2S$	$(M+H)^+$	389.1754	389.1751
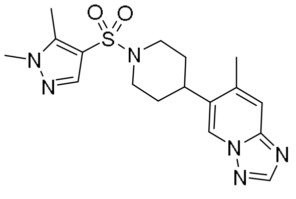	**21**	$C_{17}H_{22}N_6O_2S$	$(M+H)^+$	375.1598	375.1591

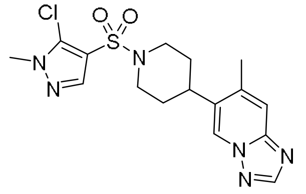	**22**	$C_{16}H_{19}ClN_6O_2S$	$(M+H)^+$	395.1051	395.1043
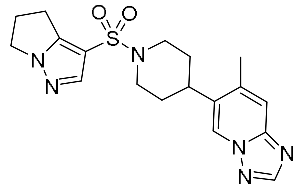	**23**	$C_{18}H_{22}N_6O_2S$	$(M+H)^+$	387.1598	387.1594
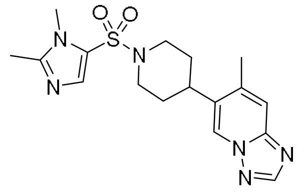	**24**	$C_{17}H_{22}N_6O_2S$	$(M+H)^+$	375.1598	375.1596
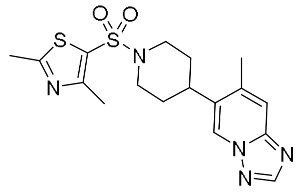	**25**	$C_{17}H_{21}N_5O_2S_2$	$(M+H)^+$	392.1209	392.1202
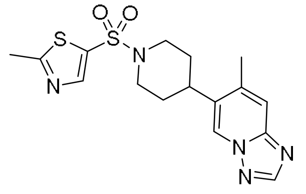	**26**	$C_{16}H_{19}N_5O_2S_2$	$(M+H)^+$	378.1053	378.1045
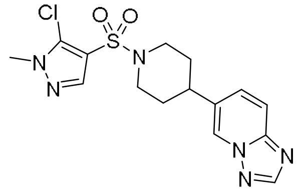	**27**	$C_{15}H_{17}ClN_6O_2S$	$(M+H)^+$	381.0895	381.0891
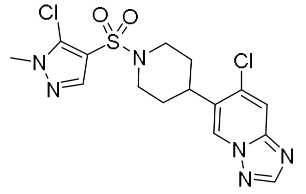	**28**	$C_{15}H_{16}Cl_2N_6O_2S$	$(M+H)^+$	415.0505	415.0501

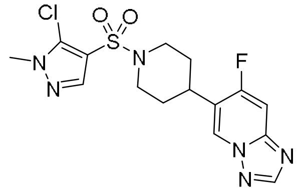	**29**	$C_{15}H_{16}ClFN_6O_2S$	$(M+H)^+$	399.0801	399.0794
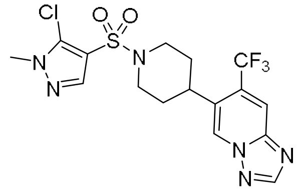	**30**	$C_{16}H_{16}ClF_3N_6O_2S$	$(M+H)^+$	449.0769	449.0763
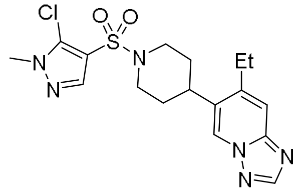	**31**	$C_{17}H_{21}ClN_6O_2S$	$(M+H)^+$	409.1208	409.1202
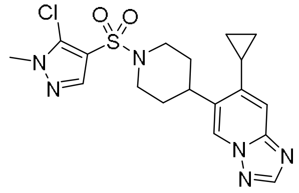	**32**	$C_{18}H_{21}ClN_6O_2S$	$(M+H)^+$	421.1208	421.1205
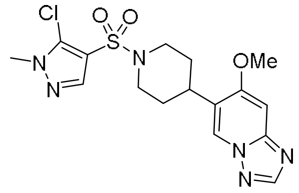	**33**	$C_{16}H_{19}ClN_6O_3S$	$(M+H)^+$	411.1001	411.1002
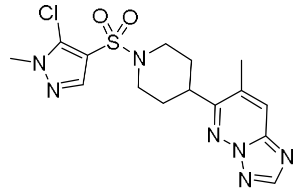	**34**	$C_{15}H_{18}ClN_7O_2S$	$(M+H)^+$	396.1004	396.0997
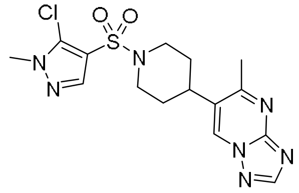	**35**	$C_{15}H_{18}ClN_7O_2S$	$(M+H)^+$	396.1004	396.0997

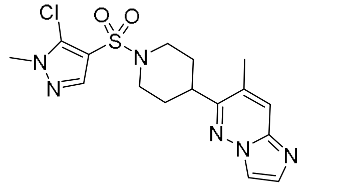	**36**	$C_{16}H_{19}ClN_6O_2S$	$(M+H)^+$	395.1051	395.1052
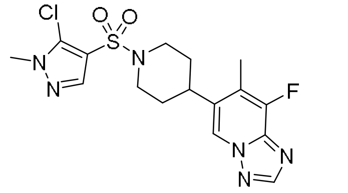	**37**	$C_{16}H_{18}ClFN_6O_2S$	$(M+H)^+$	413.0957	413.0951
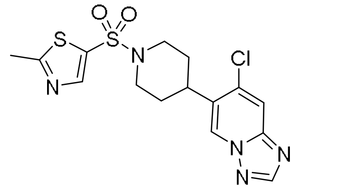	**38**	$C_{15}H_{16}ClN_5O_2S_2$	$(M+H)^+$	398.0507	398.0504
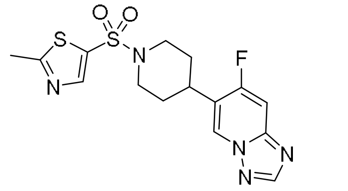	**39**	$C_{15}H_{16}FN_5O_2S_2$	$(M+H)^+$	382.0802	382.0795
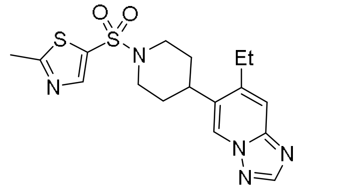	**40**	$C_{17}H_{21}N_5O_2S_2$	$(M+H)^+$	392.1209	392.1205
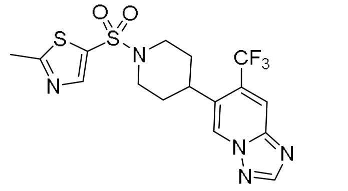	**41**	$C_{16}H_{16}F_3N_5O_2S_2$	$(M+H)^+$	432.0770	432.0766
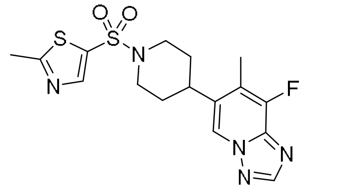	**42**	$C_{16}H_{18}FN_5O_2S_2$	$(M+H)^+$	396.0959	396.0956

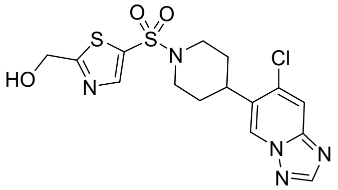	**43**	$C_{15}H_{16}ClN_5O_3S_2$	$(M+H)^+$	414.0456	414.0451
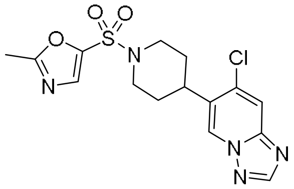	**44**	$C_{15}H_{16}ClN_5O_3S$	$(M+H)^+$	382.0735	382.0728
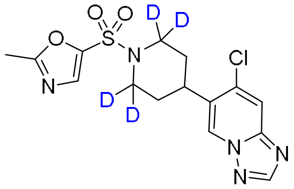	**45**	$C_{15}H_{12}D_4ClN_5O_3S$	$(M+H)^+$	386.0986	386.0986

Purity analysis by LC-MS

<table>
<tr><th>Cmpd</th><th>LC traces (215 nM and 254 nM)</th></tr>
<tr><td>9</td><td>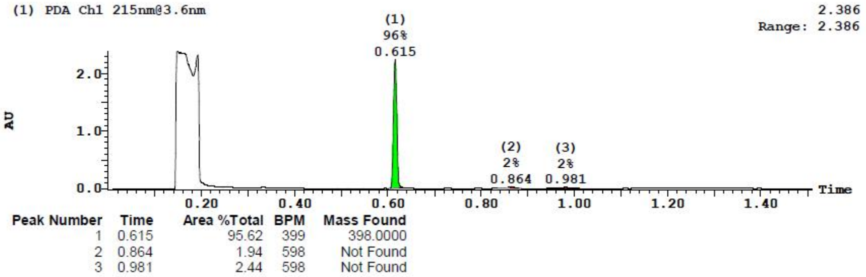

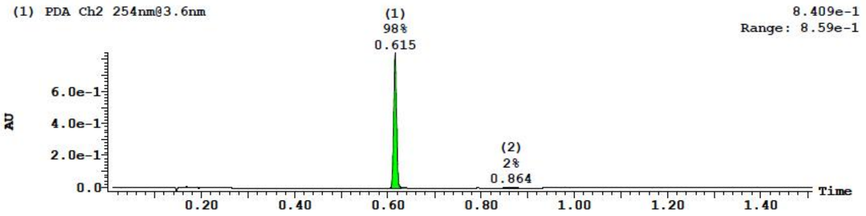
</td></tr>
<tr><td>10</td><td>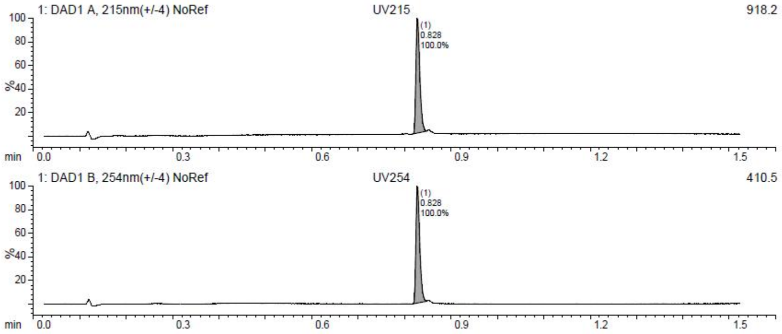
</td></tr>
<tr><td>11</td><td>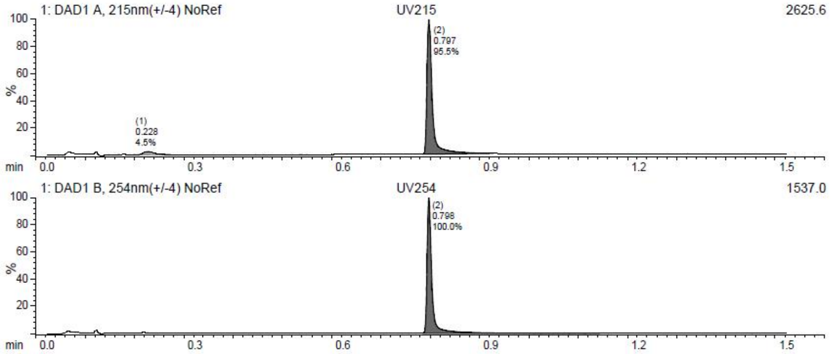
</td></tr>
</table>

12

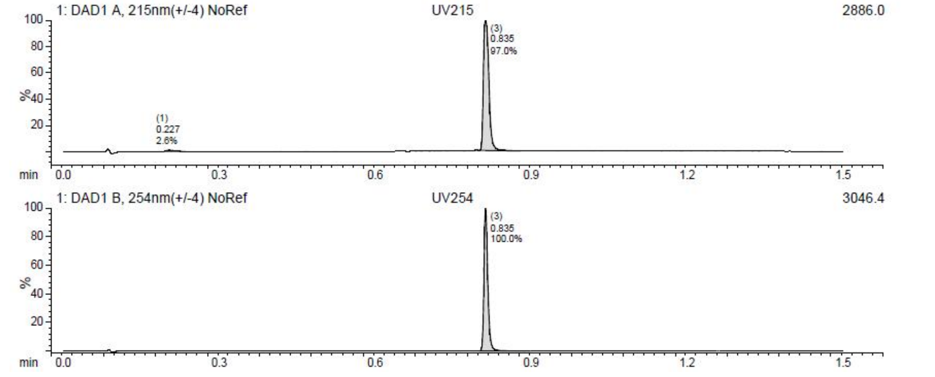
1: DAD1 A, 215nm(+/-4) NoRef
UV215
2886.0
(1)
0.227
2.6%
(3)
0.835
97.0%
1: DAD1 B, 254nm(+/-4) NoRef
UV254
3046.4
(3)
0.835
100.0%
%
min

13

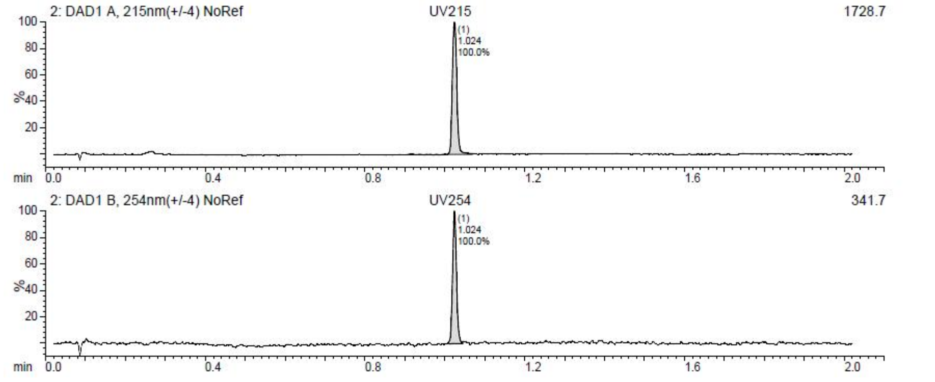
2: DAD1 A, 215nm(+/-4) NoRef
UV215
1728.7
(1)
1.024
100.0%
2: DAD1 B, 254nm(+/-4) NoRef
UV254
341.7
(1)
1.024
100.0%
%
min

14

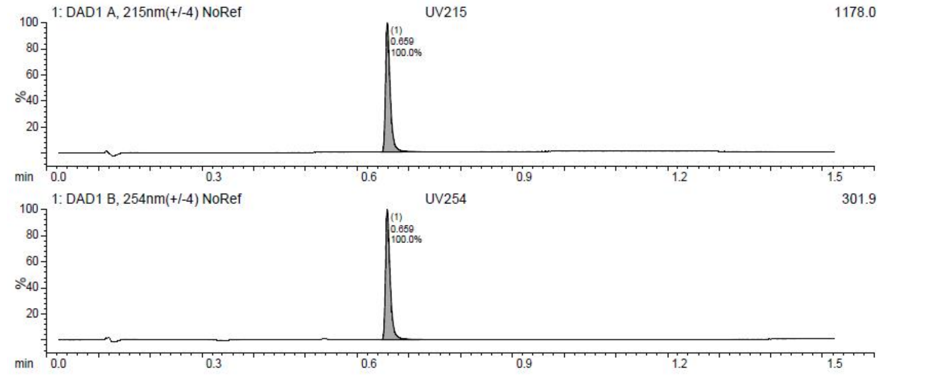
1: DAD1 A, 215nm(+/-4) NoRef
UV215
1178.0
(1)
0.659
100.0%
1: DAD1 B, 254nm(+/-4) NoRef
UV254
301.9
(1)
0.659
100.0%
%
min

15

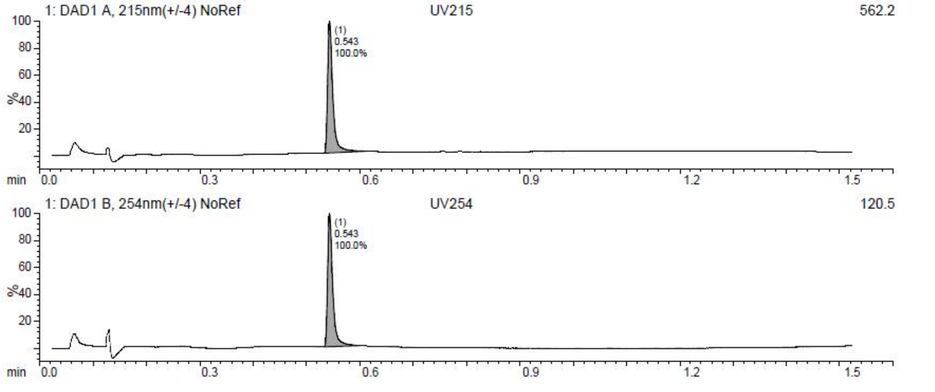
1: DAD1 A, 215nm(+/-4) NoRef
UV215
562.2
(1)
0.543
100.0%
1: DAD1 B, 254nm(+/-4) NoRef
UV254
120.5
(1)
0.543
100.0%
%
min

<table>
<tr><td>16</td><td>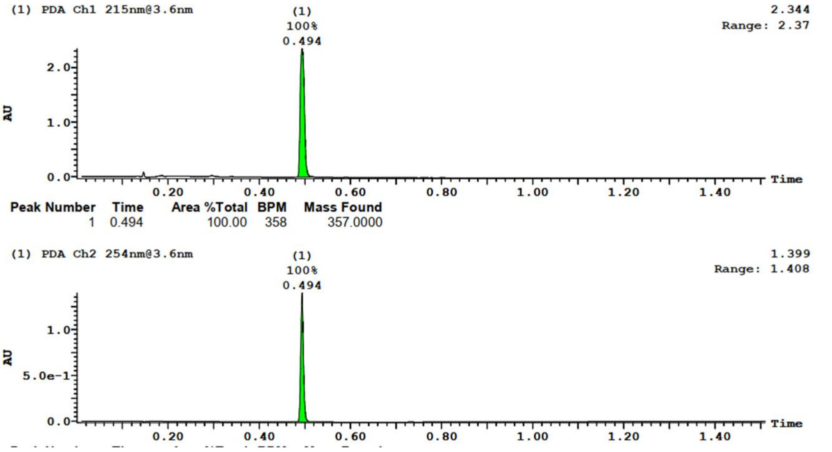
</td></tr>
<tr><td>17</td><td>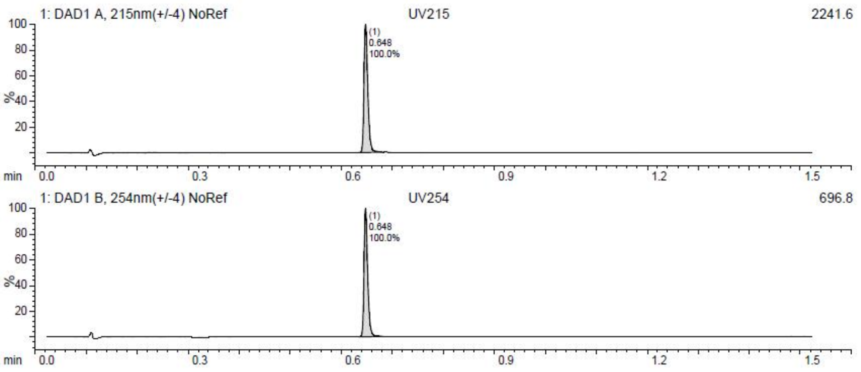
</td></tr>
<tr><td>18</td><td>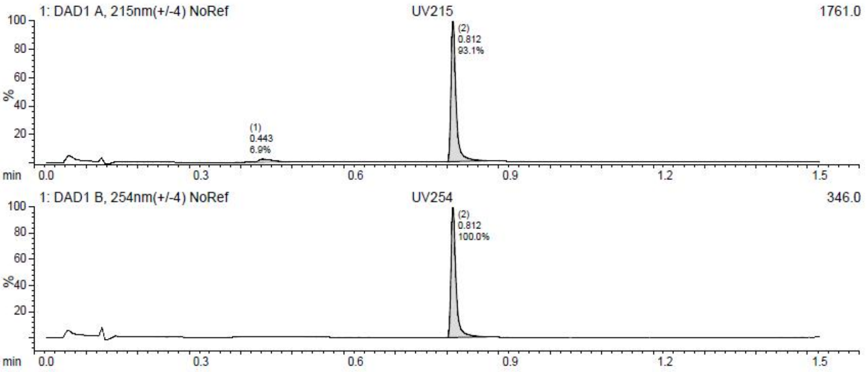
</td></tr>
</table>

19

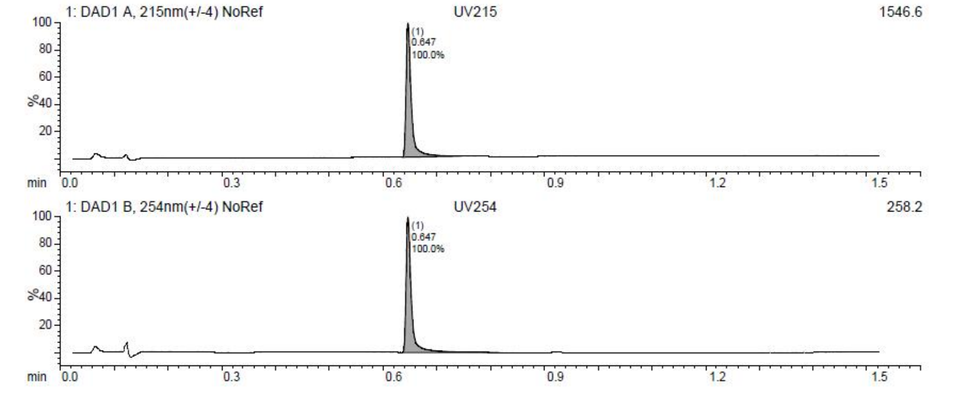
1: DAD1 A, 215nm(+/-4) NoRef
UV215
1546.6
(1)
0.647
100.0%
1: DAD1 B, 254nm(+/-4) NoRef
UV254
258.2
(1)
0.647
100.0%

20

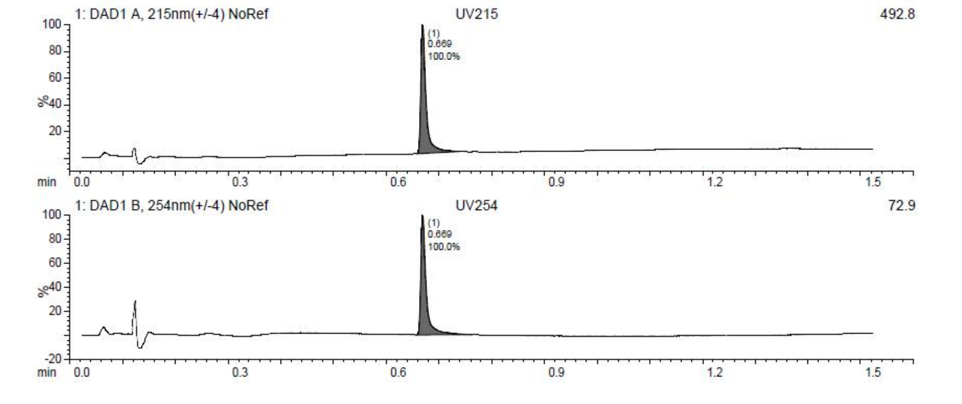
1: DAD1 A, 215nm(+/-4) NoRef
UV215
492.8
(1)
0.669
100.0%
1: DAD1 B, 254nm(+/-4) NoRef
UV254
72.9
(1)
0.669
100.0%

21

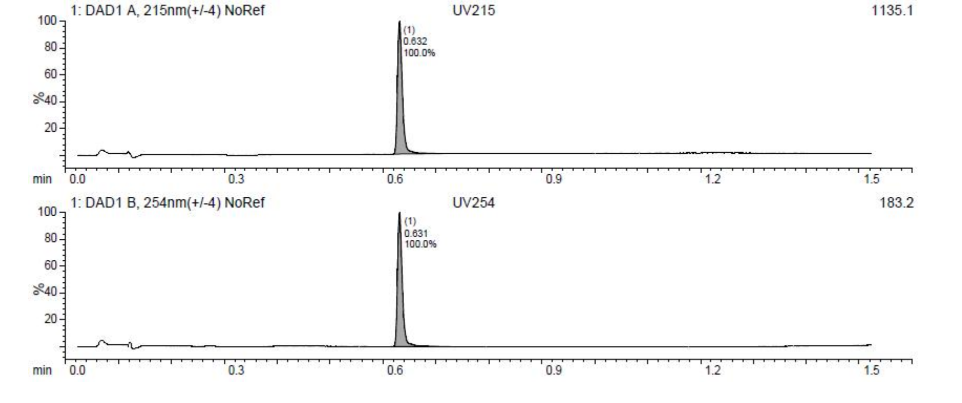
1: DAD1 A, 215nm(+/-4) NoRef
UV215
1135.1
(1)
0.632
100.0%
1: DAD1 B, 254nm(+/-4) NoRef
UV254
183.2
(1)
0.631
100.0%

22

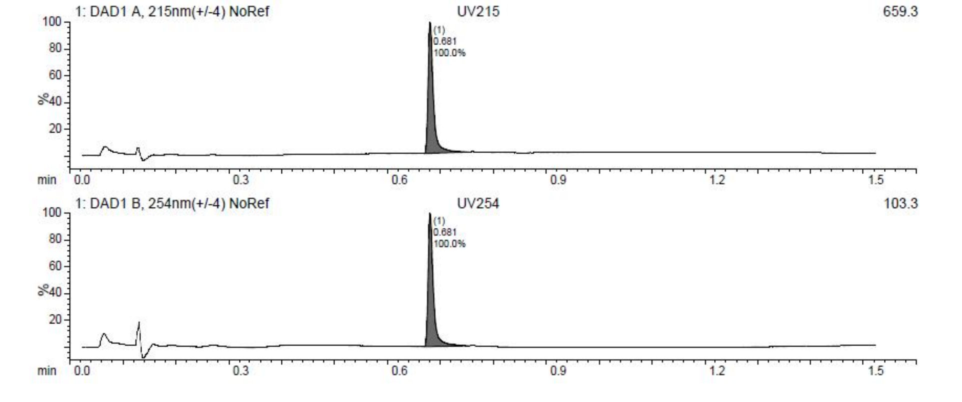
1: DAD1 A, 215nm(+/-4) NoRef
UV215
659.3
(1)
0.681
100.0%
1: DAD1 B, 254nm(+/-4) NoRef
UV254
103.3
(1)
0.681
100.0%

23

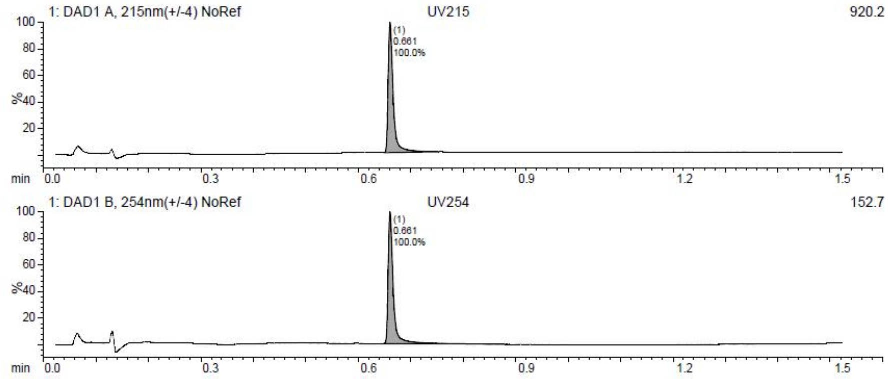
1: DAD1 A, 215nm(+/-4) NoRef
UV215
920.2
(1)
0.661
100.0%
1: DAD1 B, 254nm(+/-4) NoRef
UV254
152.7
(1)
0.661
100.0%

24

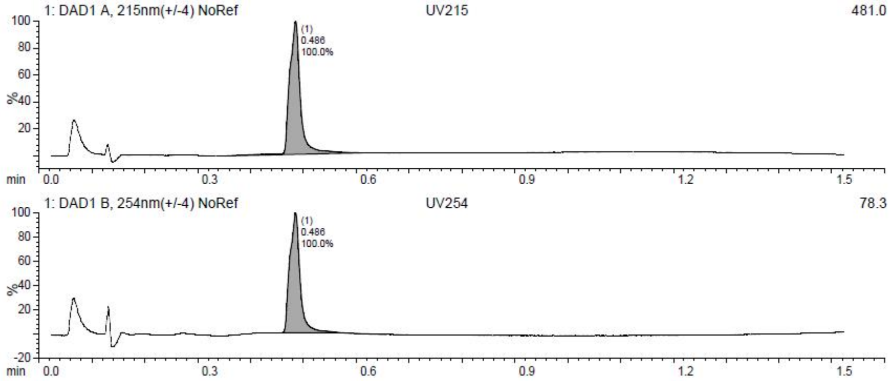
1: DAD1 A, 215nm(+/-4) NoRef
UV215
481.0
(1)
0.486
100.0%
1: DAD1 B, 254nm(+/-4) NoRef
UV254
78.3
(1)
0.486
100.0%

25

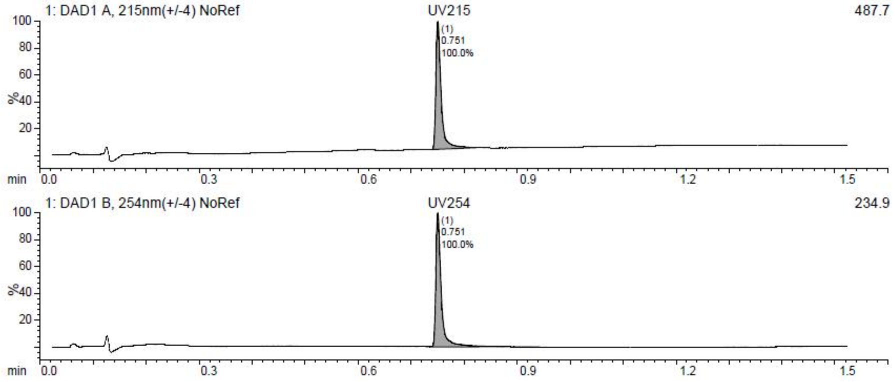
1: DAD1 A, 215nm(+/-4) NoRef
UV215
487.7
(1)
0.751
100.0%
1: DAD1 B, 254nm(+/-4) NoRef
UV254
234.9
(1)
0.751
100.0%

26

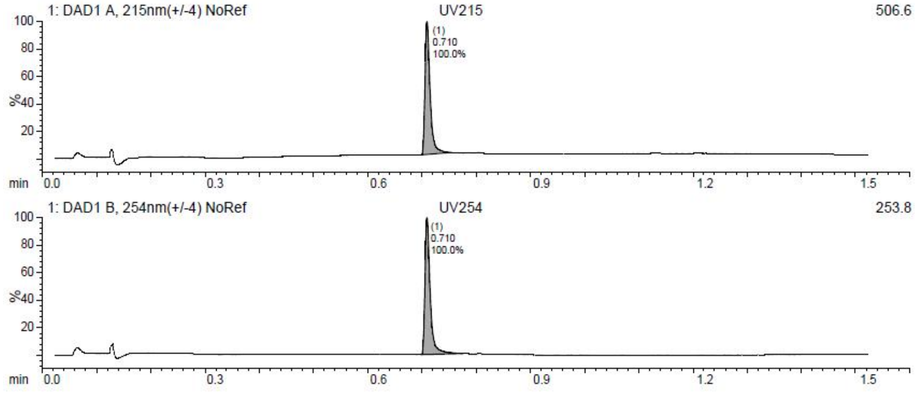
1: DAD1 A, 215nm(+/-4) NoRef
UV215
506.6
(1)
0.710
100.0%
1: DAD1 B, 254nm(+/-4) NoRef
UV254
253.8
(1)
0.710
100.0%

27

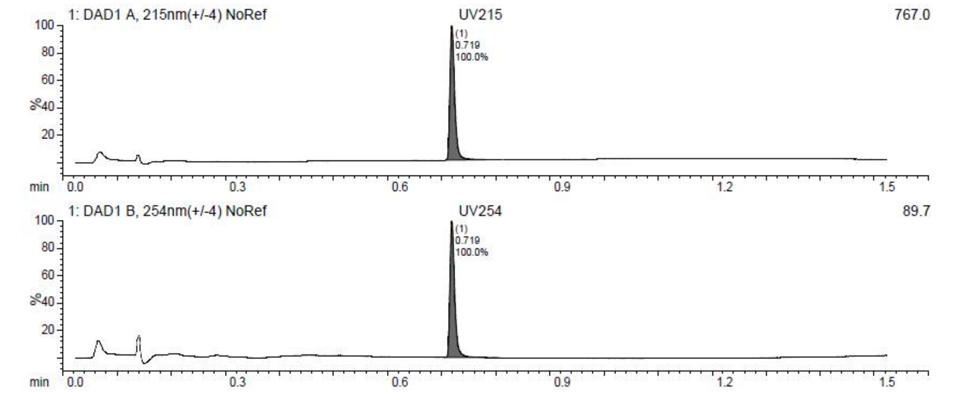
1: DAD1 A, 215nm(+/-4) NoRef
UV215
767.0
(1)
0.719
100.0%
1: DAD1 B, 254nm(+/-4) NoRef
UV254
89.7
(1)
0.719
100.0%
%
min
0.0
0.3
0.6
0.9
1.2
1.5

28

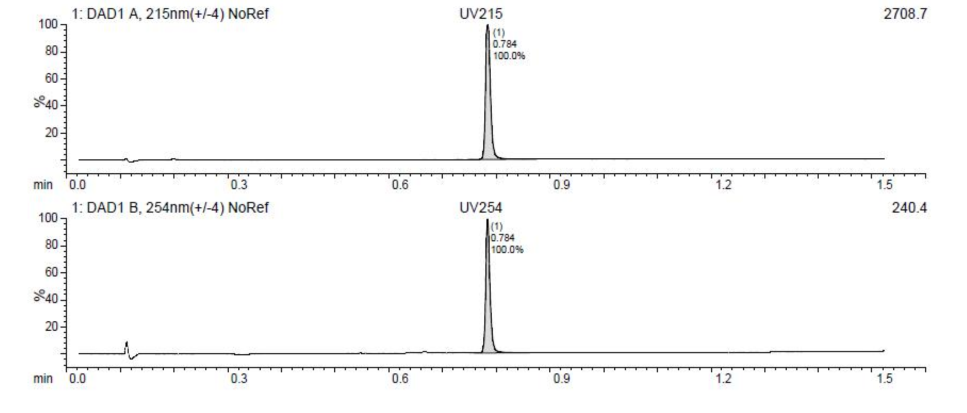
1: DAD1 A, 215nm(+/-4) NoRef
UV215
2708.7
(1)
0.784
100.0%
1: DAD1 B, 254nm(+/-4) NoRef
UV254
240.4
(1)
0.784
100.0%
%
min
0.0
0.3
0.6
0.9
1.2
1.5

29

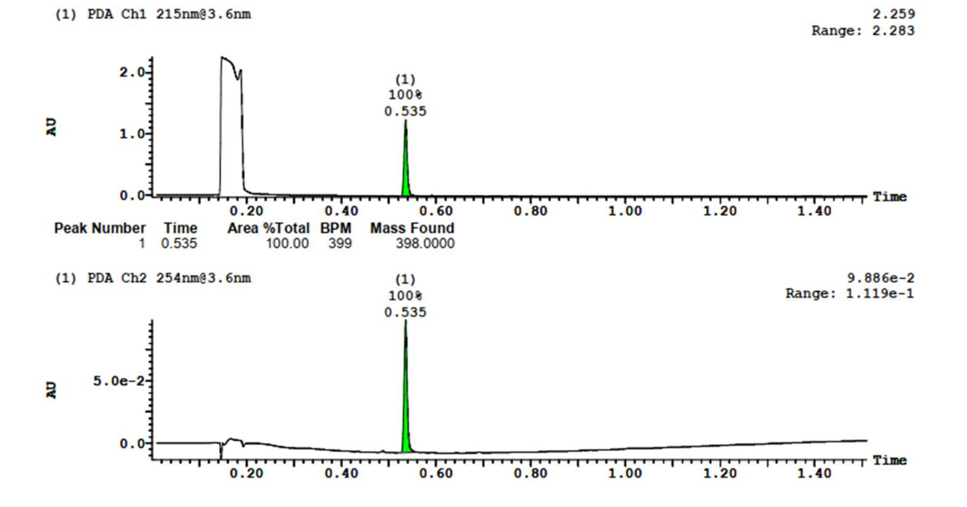
(1) PDA Ch1 215nm@3.6nm
2.259
Range: 2.283
(1)
100%
0.535
AU
Time
Peak Number Time Area %Total BPM Mass Found
1 0.535 100.00 399 398.0000
(1) PDA Ch2 254nm@3.6nm
9.886e-2
Range: 1.119e-1
(1)
100%
0.535
5.0e-2
0.0
0.20
0.40
0.60
0.80
1.00
1.20
1.40

**30**	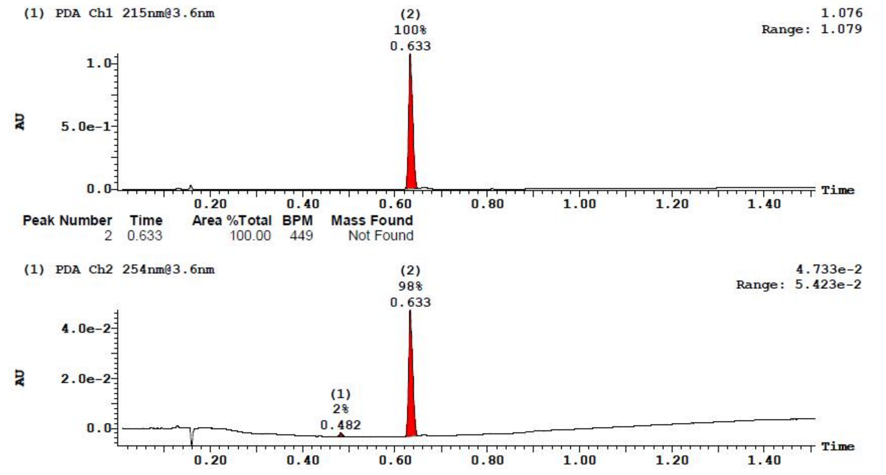 (1) PDA Ch1 215nm@3.6nm<br>(2)<br>100%<br>0.633<br>1.076<br>Range: 1.079<br>AU<br>Time<br>Peak Number Time Area %Total BPM Mass Found<br>2 0.633 100.00 449 Not Found<br>(1) PDA Ch2 254nm@3.6nm<br>(2)<br>98%<br>0.633<br>4.733e-2<br>Range: 5.423e-2<br>(1)<br>2%<br>0.482
**31**	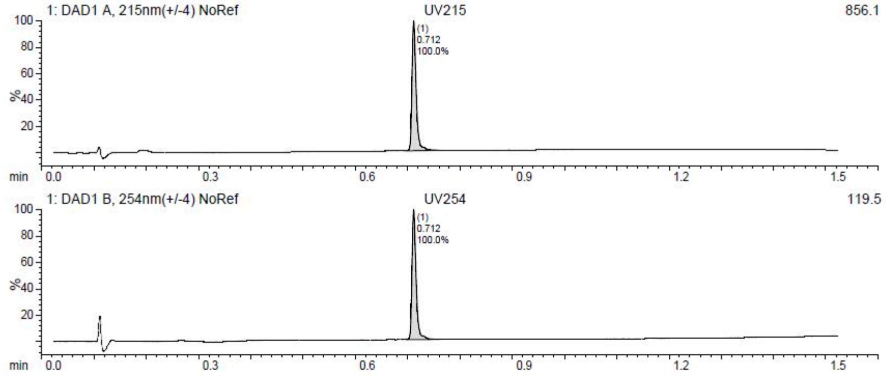 1: DAD1 A, 215nm(+/-4) NoRef<br>UV215<br>856.1<br>(1)<br>0.712<br>100.0%<br>1: DAD1 B, 254nm(+/-4) NoRef<br>UV254<br>119.5<br>(1)<br>0.712<br>100.0%
**32**	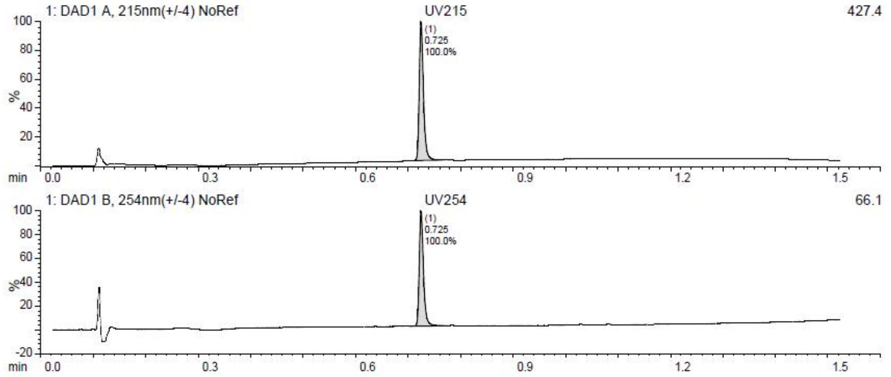 1: DAD1 A, 215nm(+/-4) NoRef<br>UV215<br>427.4<br>(1)<br>0.725<br>100.0%<br>1: DAD1 B, 254nm(+/-4) NoRef<br>UV254<br>66.1<br>(1)<br>0.725<br>100.0%

**33**	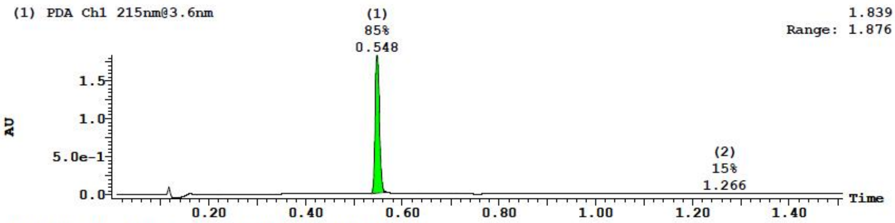<br><br>Peak Number / Time / Area %Total / BPM / Mass Found<br>1 / 0.548 / 85.10 / 411 / 410.0000<br>2 / 1.266 / 14.90 / 253 / Not Found<br>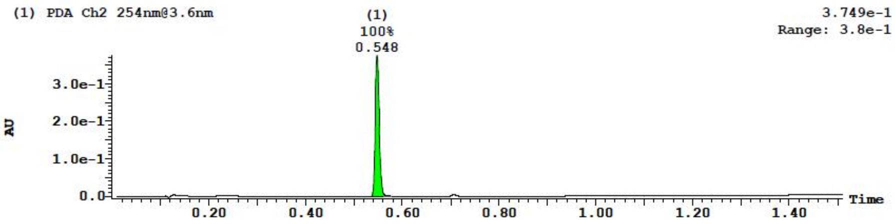
**34**	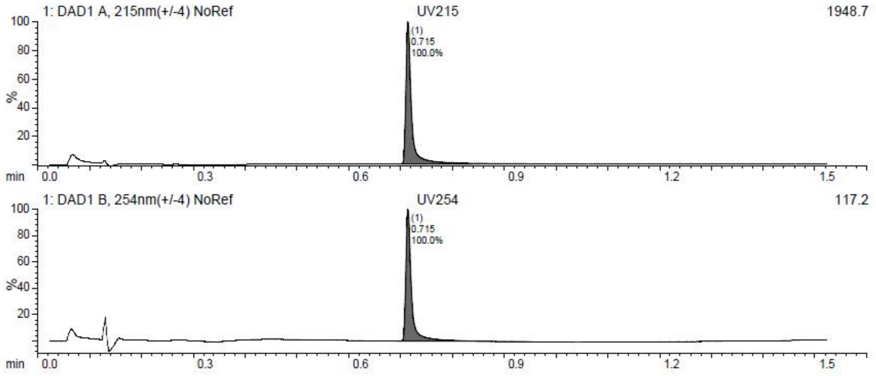
**35**	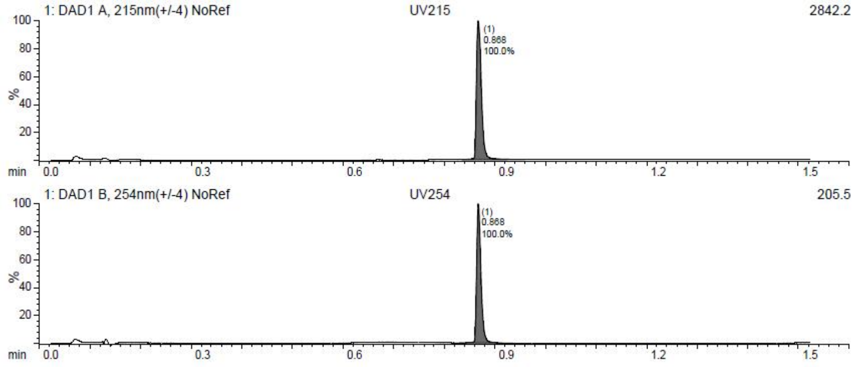

36

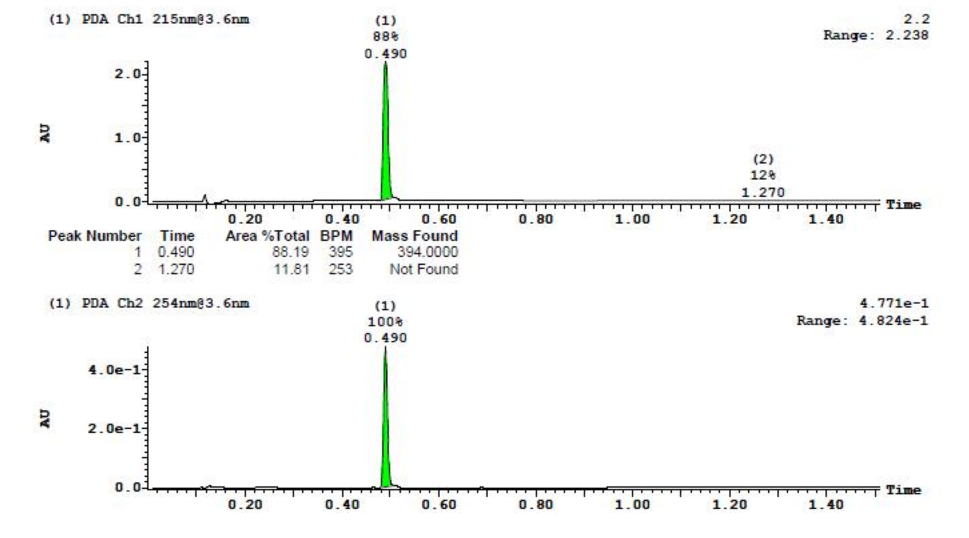
(1) PDA Ch1 215nm@3.6nm
(1)
88%
0.490
2.2
Range: 2.238
AU
(2)
12%
1.270
Time
Peak Number Time Area %Total BPM Mass Found
1 0.490 88.19 395 394.0000
2 1.270 11.81 253 Not Found
(1) PDA Ch2 254nm@3.6nm
(1)
100%
0.490
4.771e-1
Range: 4.824e-1
AU
Time

37

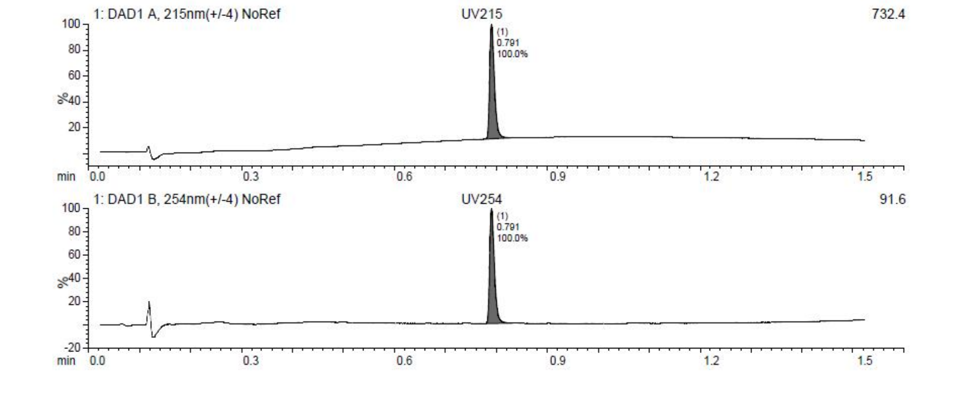
1: DAD1 A, 215nm(+/-4) NoRef
UV215
732.4
(1)
0.791
100.0%
%
min
1: DAD1 B, 254nm(+/-4) NoRef
UV254
91.6
(1)
0.791
100.0%
%
min

38

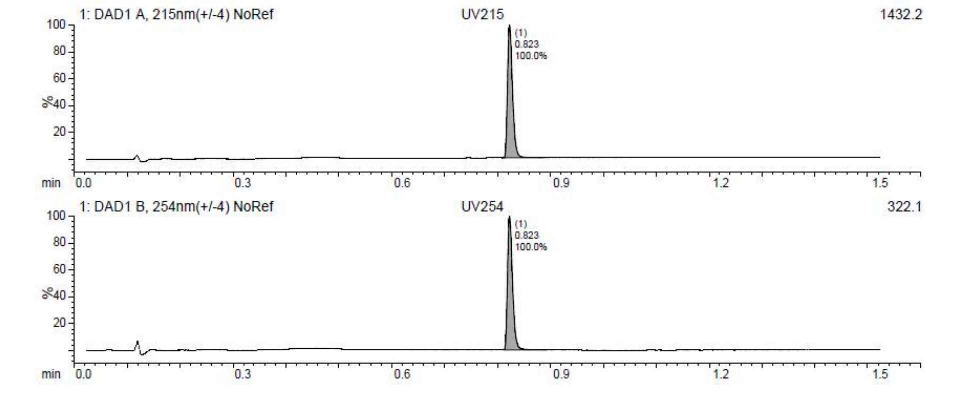
1: DAD1 A, 215nm(+/-4) NoRef
UV215
1432.2
(1)
0.823
100.0%
%
min
1: DAD1 B, 254nm(+/-4) NoRef
UV254
322.1
(1)
0.823
100.0%
%
min

**39**	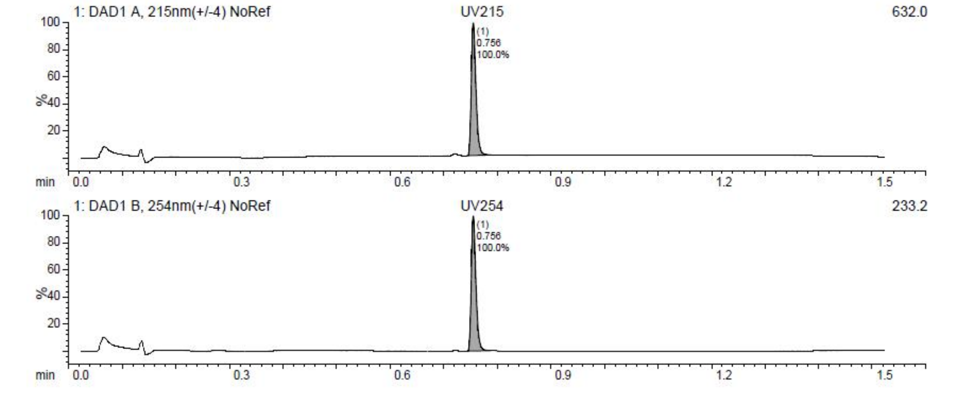1: DAD1 A, 215nm(+/-4) NoRef<br>UV215<br>632.0<br>(1)<br>0.756<br>100.0%<br>1: DAD1 B, 254nm(+/-4) NoRef<br>UV254<br>233.2<br>(1)<br>0.756<br>100.0%
**40**	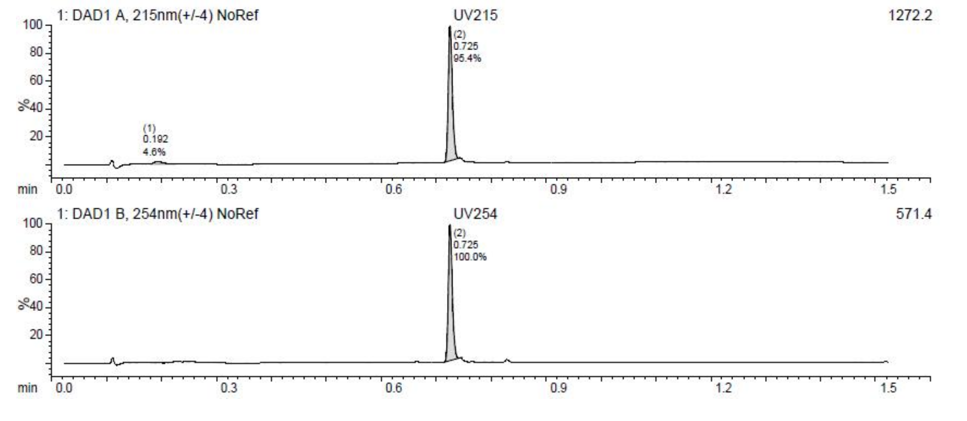1: DAD1 A, 215nm(+/-4) NoRef<br>UV215<br>1272.2<br>(1)<br>0.192<br>4.6%<br>(2)<br>0.725<br>95.4%<br>1: DAD1 B, 254nm(+/-4) NoRef<br>UV254<br>571.4<br>(2)<br>0.725<br>100.0%
**41**	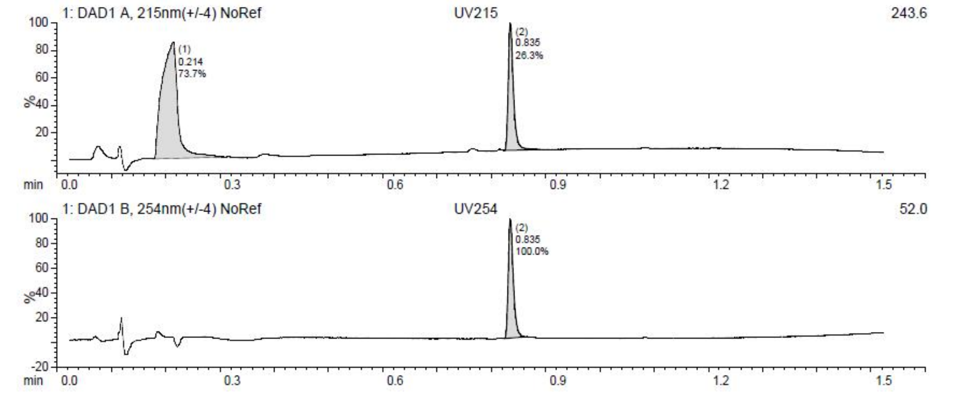1: DAD1 A, 215nm(+/-4) NoRef<br>UV215<br>243.6<br>(1)<br>0.214<br>73.7%<br>(2)<br>0.835<br>26.3%<br>1: DAD1 B, 254nm(+/-4) NoRef<br>UV254<br>52.0<br>(2)<br>0.835<br>100.0%

42

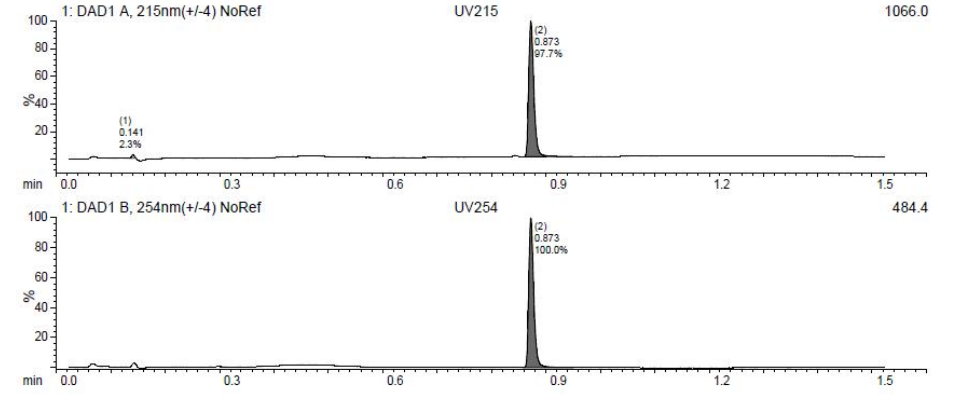
1: DAD1 A, 215nm(+/-4) NoRef
UV215
1066.0
(1) 0.141 2.3%
(2) 0.873 97.7%
1: DAD1 B, 254nm(+/-4) NoRef
UV254
484.4
(2) 0.873 100.0%

43

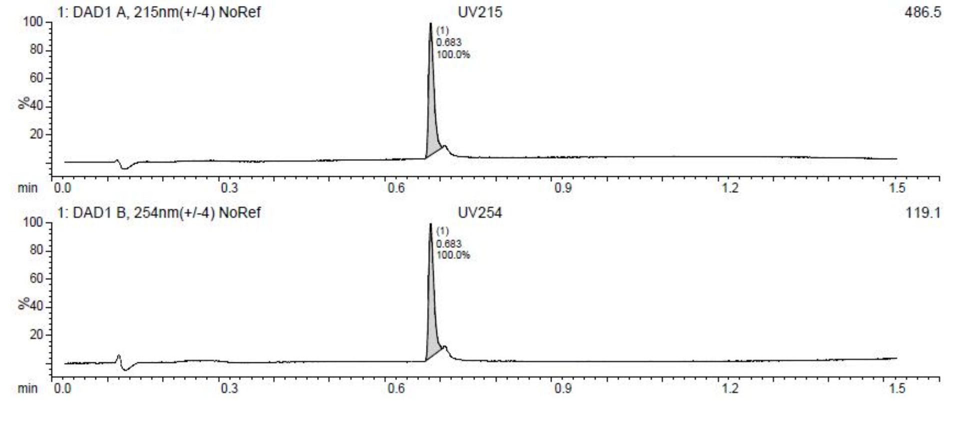
1: DAD1 A, 215nm(+/-4) NoRef
UV215
486.5
(1) 0.683 100.0%
1: DAD1 B, 254nm(+/-4) NoRef
UV254
119.1
(1) 0.683 100.0%

44

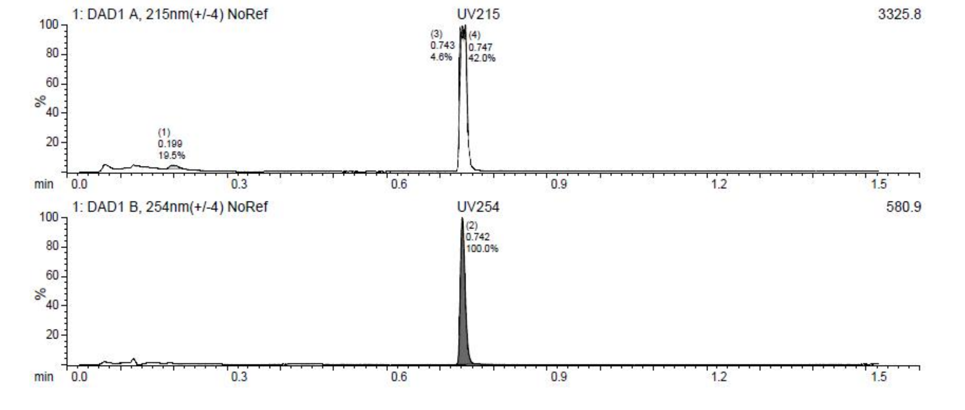
1: DAD1 A, 215nm(+/-4) NoRef
UV215
3325.8
(1) 0.199 19.5%
(3) 0.743 4.6%
(4) 0.747 42.0%
1: DAD1 B, 254nm(+/-4) NoRef
UV254
580.9
(2) 0.742 100.0%

**45**	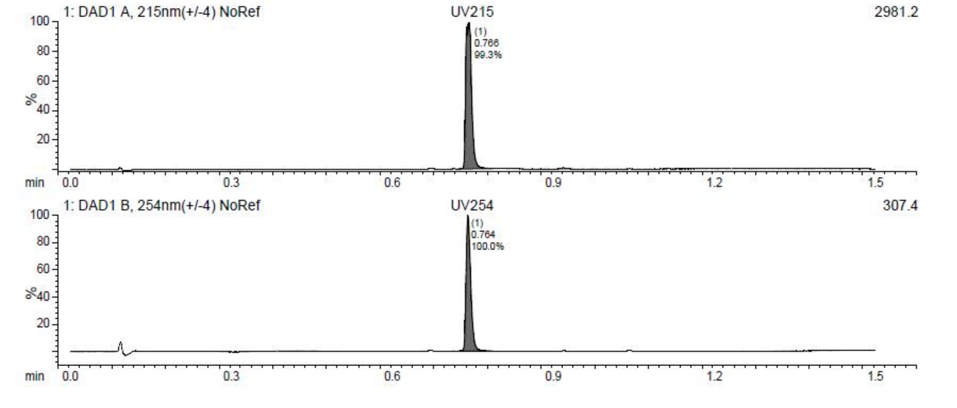

1: DAD1 A, 215nm(+/-4) NoRef
UV215
2981.2
(1)
0.766
99.3%
1: DAD1 B, 254nm(+/-4) NoRef
UV254
307.4
(1)
0.764
100.0%
%
min

References

1. Wang, Q.; Rager, J. D.; Weinstein, K.; Kardos, P. S.; Dobson, G. L.; Li, J.; Hidalgo, I. J., Evaluation of the MDR-MDCK cell line as a permeability screen for the blood-brain barrier. *Int J Pharm* **2005**, *288* (2), 349-359.

2. Lombardo, F.; Shalaeva, M. Y.; Tupper, K. A.; Gao, F., ElogDoct: A tool for lipophilicity determination in drug discovery. 2. Basic and Neutral Compounds. *J. Med. Chem.* **2001**, 44, 2490-2497.

3. Giaginis, C.; Theocharis, S.; Tsantili-Kakoulidou, A., Contribution to the standardization of the chromatographic conditions for the lipophilicity assessment of neutral and basic drugs, *Analytica Chimica Acta* **2006**, 573-574, 311-318.

4. Garrison, A. T.; Orsi, D. L.; Capstick, R. A.; Whomble, D.; Li, J.; Carter, T. R.; Felts, A. S.; Vinson, P. N.; Rodriguez, A. L.; Han, A.; Hajari, K.; Cho, H. P.; Teal, L. B.; Ragland, M. G.; Ghamari-Langroudi, M.; Bubser, M.; Chang, S.; Schnetz-Boutaud, N. C.; Boutaud, O.; Blobaum, A. L.; Foster, D. J.; Niswender, C. M.; Conn, P. J.; Lindsley, C. W.; Jones, C. K.; Han, C. Development of VU6019650: a potent, highly selective, and systemically active orthosteric antagonist of the M_5 muscarinic acetylcholine receptor for the treatment of opioid use disorder. *J Med Chem* **2022**, *65* (8), 6273-6286.
